# Stable Mesoionic N‐Heterocyclic Olefins (mNHOs)

**DOI:** 10.1002/anie.201914571

**Published:** 2020-01-27

**Authors:** Max M. Hansmann, Patrick W. Antoni, Henner Pesch

**Affiliations:** ^1^ Fakultät für Chemie und Chemische Biologie Technische Universität Dortmund Otto-Hahn-Str. 6 44227 Dortmund Germany; ^2^ Georg-August Universität Göttingen Institut für Organische und Biomolekulare Chemie Tammannstr. 2 37077 Göttingen Germany

**Keywords:** carbene homologues, main-group chemistry, N-heterocyclic olefins, ylides

## Abstract

We report a new class of stable mesoionic N‐heterocyclic olefins, featuring a highly polarized (strongly ylidic) double bond. The ground‐state structure cannot be described through an uncharged mesomeric Lewis‐structure, thereby structurally distinguishing them from traditional N‐heterocyclic olefins (NHOs). mNHOs can easily be obtained through deprotonation of the corresponding methylated *N*,*N′*‐diaryl‐1,2,3‐triazolium and *N*,*N′*‐diaryl‐imidazolium salts, respectively. In their reactivity, they represent strong σ‐donor ligands as shown by their coordination complexes of rhodium and boron. Their calculated proton affinities, their experimentally derived basicities (competition experiments), as well as donor abilities (Tolman electronic parameter; TEP) exceed the so far reported class of NHOs.

## Graphical abstract

**Highly charged**: A new class of mesoionic N‐heterocyclic olefins is described featuring a highly ylidic double bond. The compounds can be utilized as ligands in main‐group and transition metal chemistry. Their donor properties exceed previously reported traditional N‐heterocyclic olefins.

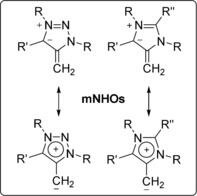

## Introduction

**Scheme 1:**
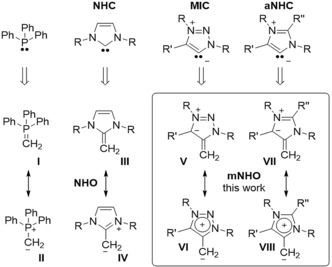
Comparison between methylene phosphoranes (**I/II**), N‐heterocyclic olefins (NHOs) (**III**/**IV**), and mNHOs (**V**–**VIII**) (this work).

Novel bonding modes of carbon, especially strong carbon‐based donor ligands, such as carbenes, have received great interest throughout the last decades and continue to have large impact on the chemical community and beyond. N‐heterocyclic olefins (NHOs, **III**), cyclic derivatives of ene‐1,1‐diamines,^1^ featuring formally an alkylidene moiety appended to a N‐heterocyclic carbene, were first described by Kuhn et al. in 1993,^2^ and popularized by the acronym NHOs by Rivard et al. in 2011.^3^ As initially reported, they represent electron rich “ylidic olefins” which can be described through a neutral (**III**) and zwitterionic mesomeric structure (**IV**) electronically reminiscent to methylene phosphoranes (**I**/**II**) (Scheme 1). As a result of the good stabilization of the positive charge by the aromatic imidazolium moiety, NHOs are highly polarized towards the exocyclic carbon atom leading to strong σ‐donor properties, even exceeding those of NHCs.^4^ So far NHOs and their derivatives, which can be considered as deoxy‐Breslow type intermediates,^5^ have found broad application in several fields such as transition metal and organo catalysis,^6^ main‐group^7^ and coordination chemistry,^8^ or polymer chemistry.^[9,^ ^10]^ Besides Arduengo‐type N‐heterocyclic carbenes,^11^ abnormal N‐heterocyclic carbenes (aNHC),^12^ and mesoionic carbenes (MICs), 1H‐1,2,3‐triazol‐5‐ylidenes^13^ have been shown to be isolable, stable and bottable carbenes with strong σ‐donor properties exceeding traditional NHCs.^14^

While formal methylene extension of phosphines and N‐heterocyclic carbenes leads to methylene phosphoranes (**I**/**II**) and NHOs (**III**/**IV**) respectively, we were curious if formal methylene extension of mesoionic and abnormal carbenes would result in stable compounds (Scheme 1). Note, in contrast to **I**–**IV**, such “olefins” would be mesoionic,^15^ in analogy to MICs, as they cannot be represented by a canonical Lewis resonance structure without charge separation (**V**–**VIII**). Such structural motive is rare, as in general, mesoionic compounds typically feature chalcogen atoms and not carbon in the exocyclic position.^15^ Such an overlooked class of mesoionic N‐heterocyclic olefins (mNHOs) should exhibit strong donor abilities, considering a correlation with the parent carbene entities (see below).

## Results and Discussion

**Scheme 2:**
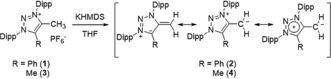
Synthesis of stable mesoionic N‐heterocyclic olefins **2** and **4** (Dipp=2,6‐diisopropylphenyl).

In order to access **V**/**VI** we envisioned deprotonation of the corresponding methylated 1,2,3‐triazolium salts. *N*,*N′*‐Diaryl‐1,2,3‐triazolium salts such as **1** or **3** are easily accessible in two steps starting from commercial starting materials through formal cycloaddition of 1,3‐diaza‐2‐azoniaallene salts with alkynes.^16^ Upon addition of one equivalent potassium bis(trimethylsilyl)amide (KHMDS) to a solution of **1** an instant color change from colorless to deep purple is observed. Upon extraction with pentane, **2** is isolated as deep purple solid in 67 % yield (Scheme 2). **2** is highly sensitive towards oxygen, however, stable as solid or in solution under inert atmosphere over several days.

**Figure 1:**
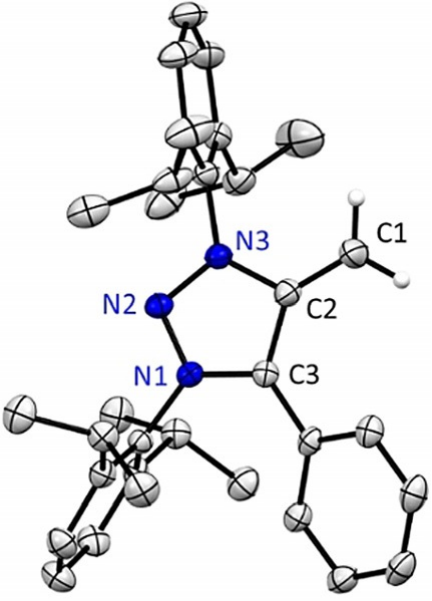
X‐ray solid‐state structure of mNHO **2**. Hydrogen atoms except=CH_2_ moiety (positions refined) omitted for clarity. Ellipsoids set at 50 % probability. Selected bond parameters in [Å] and [°] as well calculated bond distances [BLYP/def2‐TZVPP] in brackets in [Å]: C1−C2 1.361(1) [1.368], C2−C3: 1.435(1) [1.458], C3−N1: 1.356(1) [1.382], N1−N2: 1.324(1) [1.333], N2−N3: 1.369(1) [1.372], N3−C2: 1.399(1) [1.423].

^1^H NMR spectroscopy of **2** in [D_8_]THF at room temperature shows two signals at *δ*=3.03 and 2.19 ppm for the exocyclic olefinic (MIC)=CH_2_ protons. The unusual chemical shift range is in agreement with olefin signals observed for NHOs, being typically in the range of 2.4–3.2 ppm (for a summary of typical NMR shifts, see the Supporting Information). The exocyclic ^13^C signal for **2** is obtained at pronounced high field (*δ*=44.3 ppm), clearly deviating from the normal olefin range, but in line with NHOs (see the Supporting Information).5a Intrigued by the broad shape of the proton signals ^1^H EXSY NMR spectroscopy indicated, at first sight, exchange of the two exocyclic protons indicating rotation of the C−C bond. However, upon addition of small quantities of KHMDS to the NMR sample we observe sharp ^1^H NMR signals for **2** at room temperature (for an NMR discussion see the Supporting Information). We reason that a small amount of H^+^ catalyzes the proton exchange reaction (fast protonation, rotation, deprotonation). Clearly, the unusual chemical ^1^H and ^13^C NMR shifts indicate a high charge density at the exocyclic CH_2_ moiety and a highly polarized zwitterionic structure (**VI**; Scheme 1). Next, we investigated the structurally simple symmetrical diaryl‐1,2,3‐triazolium salt featuring two methyl groups (**3**). In analogy, addition of one equivalent KHMDS afforded, after extraction, **4** as red solid in 69 % yield. From a −35 °C pentane solution we were able to obtain single crystals of **2** suitable for X‐ray diffraction (Figure 1).^17^

Most strikingly, the CH_2_ fragment is positioned coplanar to the five‐membered ring. The C1−C2 distance [1.361(1) Å] is significantly shortened compared to the cationic starting material MIC−CH_3_ (**1**) [1.478(5) Å, see the Supporting Information] and slightly longer compared to Kuhn's reported tetramethyl substituted N‐heterocyclic olefin [1.357(3) Å]2a (for a comparison of bond distances, see the Supporting Information). We also obtained single crystals of **4**, however, X‐ray analysis cannot discriminate between the two −CH_3_/=CH_2_ sites as in the solid‐state both molecule orientations overlay, causing averaged bond distances (see the Supporting Information).

**Figure 2:**
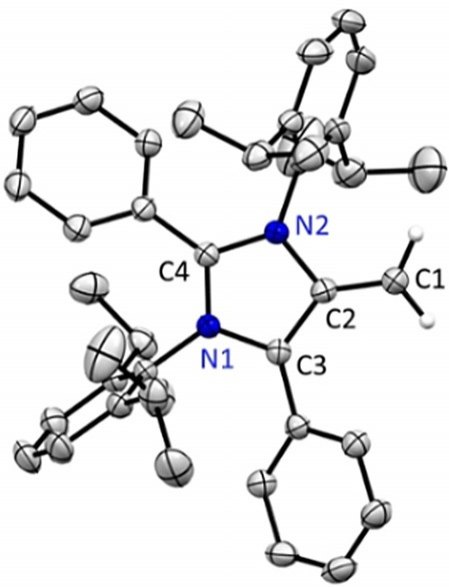
X‐ray solid‐state structure of mNHO **6**. Hydrogen atoms except =CH_2_ moiety (positions refined) omitted for clarity. Ellipsoids set at 50 % probability. Selected bond parameters in [Å] and [°] as well calculated bond distances [BLYP/def2‐TZVPP] in brackets in [Å]: C1−C2 1.363(2) [1.371], C2−C3 1.432(2) [1.446], C3−N1 1.403(1) [1.409], N1−C4 1.341(1) [1.369], C4−N2 1.371(1) [1.391], N2−C2 1.423(1) [1.438].

**Scheme 3:**
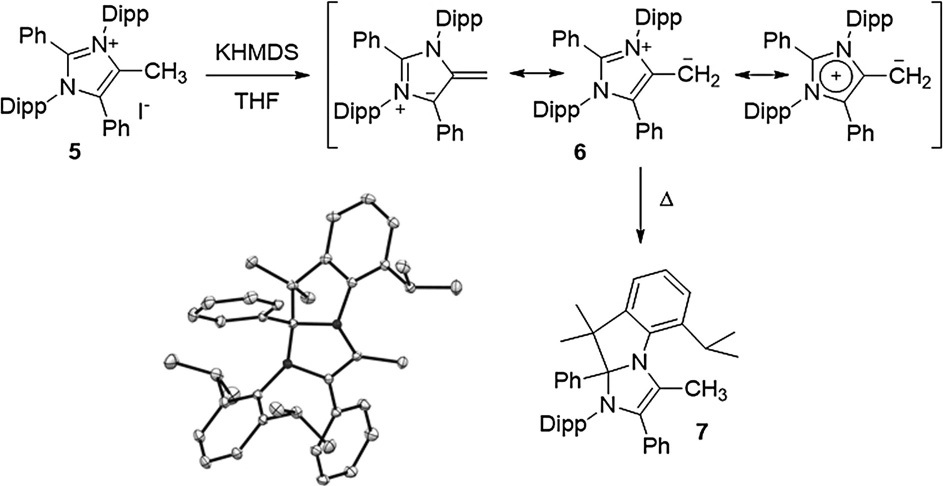
Synthesis of mNHO **6** and its rearrangement product **7**; left: X‐ray solid‐state structure of **7**.

Next, we investigated mNHOs derived from abnormal NHCs. Deprotonation of **5**, easily accessible by methylation of the corresponding stable abnormal carbene, with one equivalent of KHMDS leads to an intense deep green solution (Scheme 3). Extraction with toluene affords **6** in 59 % yield. ^1^H NMR spectroscopy in [D_8_]THF shows for the exocyclic moiety signals at 3.21 ppm and 2.04 ppm and a ^13^C signal at 46.6 ppm. We were able to obtain single crystals of **6** suitable for X‐ray diffraction (Figure 2). The C1−C2 bond distance [1.363(2) Å] is close to **2** but longer than traditional NHOs (see Figure S2, Supporting Information) and is positioned in the range of recently reported lithiated NHOs.7a, 7b Interestingly, **6** is stable in the solid‐state, but in contrast to **2** and **4**, a slow (*t*_1/2_≈5–6 days) rearrangement process takes place at room temperature to give **7**. This species is reminiscent of the decomposition product of abnormal carbenes,^12^ formed through deprotonation of the isopropyl group and intramolecular attack of the resulting anion onto the imidazolium C2‐position. DFT calculations indicate the rearrangement from **6** to **7** being exergonic by Δ*G*≈−3.2 kcal mol^−1^, while the analogous hypothetical rearrangement of **2** would be endergonic by circa 7.2 kcal mol^−1^ (see the Supporting Information). This result hints towards the extraordinarily high basicity of the new mNHO class.

**Figure 3:**
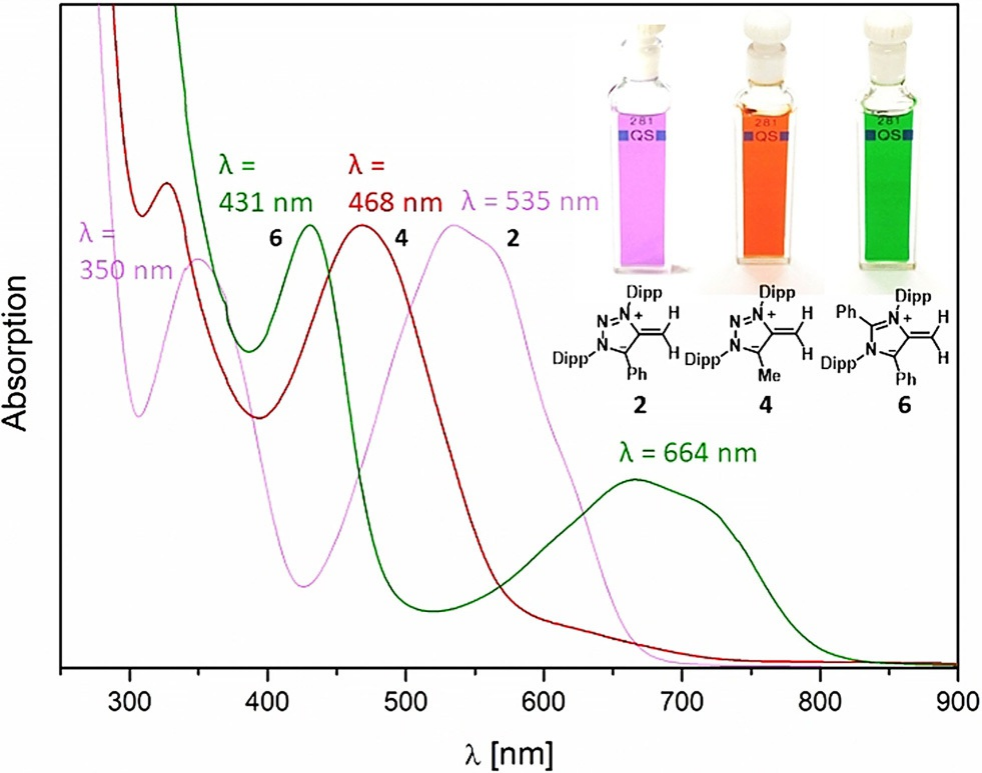
UV/Vis spectrum and optical appearance of mNHOs.

In contrast to N‐heterocyclic olefins, which are typically colorless solids, the mesoionic species investigated here are all intensely colored. Both **2** and **6** feature two absorptions in the visible area [**2**: *λ*=535 nm (*ϵ*: 7062 cm^−1^ m ^−1^), 350 nm (*ϵ*: 6236 cm^−1^ m ^−1^); **6**: 664 nm (*ϵ*: 851 cm^−1^ m ^−1^), 431 nm (*ϵ*: 1880 cm^−1^ m ^−1^)] (Figure 3).

**Figure 4:**
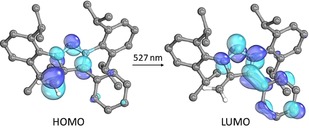
Calculated transition (TD‐DFT B3LYP/def2‐TZVPP) for the main absorption of **2** in the visible area.

Computational analysis at the TD‐DFT [B3LYP/def2‐TZVPP//B3LYP/def2‐TZVP] level of theory is in good agreement with the observed visible transitions (**2**: calc. 527 nm, 337 nm; for **4** and **6** see the Supporting Information). The main bathochromic shifted absorptions are due to HOMO→LUMO charge transfer transitions from the negatively polarized ‐CH_2_ moiety to the cationic heterocyclic moiety (Figure 4).

**Scheme 4:**
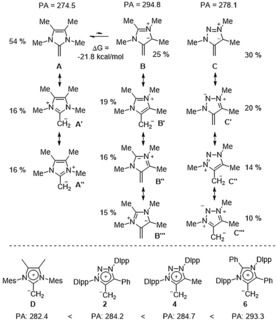
Comparison of the parent NHO (**A**) and the parent mNHOs (**B** and **C**). The % calculated contribution to the resonance structure based on NRT calculation. Proton affinities (PA) [kcal mol^−1^] calculated at the BLYP/def2‐TZVPP level of theory.

In order to rationalize our experimental results, we performed DFT calculations of the parent olefins **A**, **B**, and **C** as well as the full systems **D**, **2**, **4**, and **6** (Scheme 4). While **A** and **B** are isomeric structures, similar to NHC and aNHC, the deprotonation to generate **A** over **B** is thermodynamically favored by 21.8 kcal mol^−1^ (B3LYP/def2‐TZVPP). Natural resonance theory (NRT) calculations implemented into the NBO 6.0 program were performed to evaluate the relative contributions of resonance structures. In line with the definition of the word mesoionic, **B** and **C** cannot be satisfactory described by a single covalent or polar structure but expressed as a resonance hybrid of a series of dipolar canonical structures (for a full list see the Supporting Information).^15^

While **A** is described by a total relative ratio of 64 % C=CH_2_ and 32 % C−CH_2_^−^ contribution, this ratio increases for **B** and **C** (both 57 % C=CH_2_ to 43 % C−CH_2_^−^) indicating a larger ylidic polarization of mNHOs compared to traditional NHOs (see the Supporting Information). In line, calculated proton affinities^18^ at the BLYP/def2‐TZVPP level reflect the basicity trend **A** (274.5) < **C** (278.1) < **B** (294.8), expecting the new mNHO class to be superior bases compared to traditional NHOs. Calculation of the full systems confirm this trend **D** (282.4) <**2** (284.2) <**4** (284.7) <**6** (293.3). Recently Naumann et al. showed computationally that traditional NHO **D** is the most basic NHO with a calculated PA of 282.1 kcal mol^−1^.^18^ Clearly, both new mNHO classes exceed PAs of traditional NHCs (262–275 kcal mol^−1^),^19^ regular NHOs^18^ and are at the limit of the strongest known carbon based PAs.^19^, ^20^ Interestingly, we also calculated at the same level of theory the PAs of the parent carbene entities, which feature an analogous trend (NHC: 283.7; MIC: 284.2; aNHC: 296.8).

**Scheme 5:**
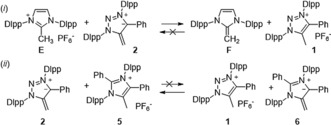
Competition experiments.

Competition experiments between mNHOs and protonated species were performed in [D_8_]THF to test the calculated PA trend (Scheme 5). Upon mixing **E** with mNHO **2**, the clean and quantitative formation of NHO **F** and triazolium salt **1** was observed (see the Supporting Information). Furthermore, addition of mNHO **6** to **1** leads quantitatively and instantaneously to mNHO **2** and salt **5**. Both competition experiments clearly establish the experimental basicity trend NHO (**F**) < mNHO (**2**/**4**) < mNHO (**6**).

**Scheme 6:**
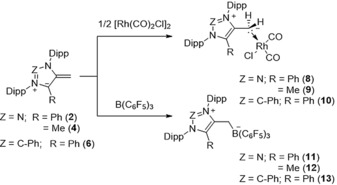
Coordination of mNHOs to rhodium and boron.

In order to study the application of the mNHOs as ligands and to analyze their donor strengths, we prepared the corresponding rhodium and boron complexes (Scheme 6).

**Figure 5:**
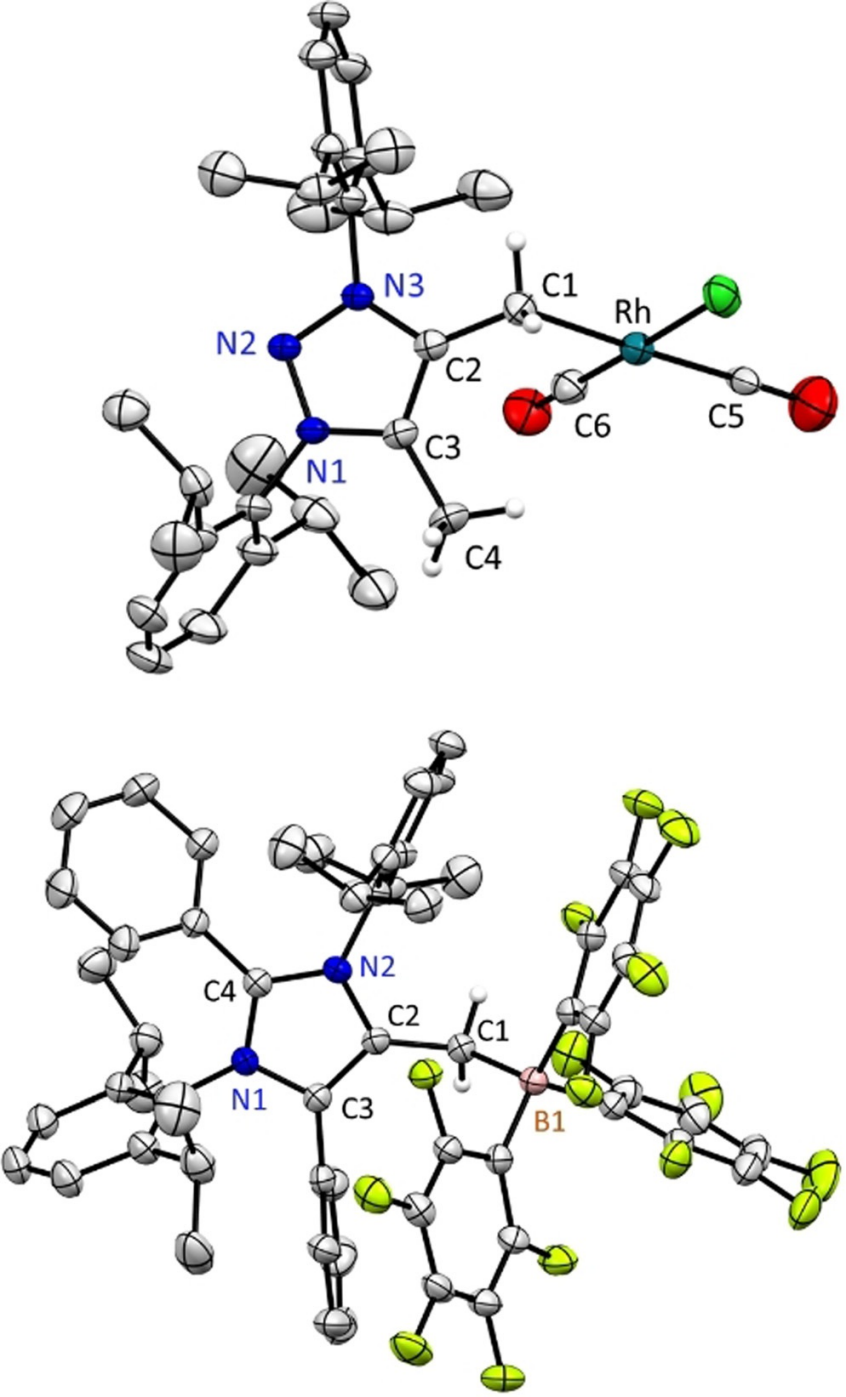
Selected X‐ray solid‐state structures of **9** and **13**. Solvent molecule (**13**: toluene) and hydrogen atoms (except CH_2_/CH_3_) omitted for clarity. Ellipsoids set at 50 % probability probability. Selected bond parameters in [Å] and [°]: **9**: C1‐Rh: 2.137(2), C5−Rh 1.871(2), C6−Rh 1.795(2), C1−C2: 1.458(3), C2−C3: 1.384(3), C3−N1: 1.351(2), N1−N2: 1.331(2), N2−N3: 1.337(2), N3−C2: 1.364(2), C3−C4 1.485(3); **13**: C1−B1 1.661(5), C1−C2 1.482(4), C2−C3 1.368(4), C3−N1 1.393(4), N1−C4 1.349(4), C4−N2 1.356(4), N2−C2 1.400(4).

Slow addition of the colored mNHOs (**2**, **4**, and **6**) to toluene solutions of [Rh(μ‐Cl)(CO)_2_]_2_ as well as B(C_6_F_5_)_3_ gave access to colorless or slightly yellow compounds **8**–**10** and **11**–**13**, respectively. The solid‐state structure of **9** (for **8** and **10** see the Supporting Information)^17^ establishes the end‐on binding mode by the exocyclic α‐carbon atom to square planar rhodium, with CO being *trans* coordinated (Figure 5). The C1−C2 bond distances are strongly elongated [**8**: 1.457(2) Å; **9**: 1.458(3) Å; **10**: 1.461(5) Å] and even longer in the solid‐state structures of boron betains **12** [1.496(2) Å] and **13** [1.482(4) Å]. Note, the C3−C4 bond distance [**9**: 1.485(3) Å; **12**: 1.490(2) Å] can be considered as internal reference for a C_(triazolium)_−CH_3_ single bond. The Rh−C1 distances [**8**: 2.151(2) Å; **9**: 2.137(2) Å; **10**: 2.158(4) Å] are similar to reported NHO complexes (see the Supporting Information).

IR stretching frequencies of [RhCl(CO)_2_L] complexes are generally used to derive the Tolmann electronic parameter (TEP) which relates to the overall donor properties of a ligand L.^21^ As reference we prepared the corresponding IPr and IPr=CH_2_ complexes and measured the IR carbonyl stretching frequencies in CH_2_Cl_2_ (Table 1; for ATR measurements see the Supporting Information). As pointed out previously,^4^ NHOs are overall stronger donors than NHCs (ν_av_ 2038 vs. 2014 cm^−1^; entry 1 and 2), due to negligible π‐backbonding. Importantly, in line with the basicity trends, the IR frequencies follow the trend ν_av_ (NHO) > ν_av_ (**8**) ≈ν_av_ (**9**) > ν_av_ (**10**). Note, **10** (TEP: 2023 cm^−1^) is an exceptional strong donor exceeding common ligand classes such as phosphines, NHCs, NHOs, and even MICs and aNHOs.

**Table 1:** Comparison of carbonyl stretching frequencies measured in CH_2_Cl_2_ in [cm^−1^], averaged ν_av_ and calculated TEP (TEP=0.8001 ν_av_ + 420 cm^−1^).^21^ IPr: 1,3‐(2,6‐diisopropylphenyl)imidazolin‐2‐ylide.

Head 1^[a]^	IR v (CO)	ν_av_ (CO)	TEP [cm^−1^]
**(IPr)RhCl(CO)_2_**	2080.3/1996.9	2038.6	2051.1
**(IPrCH_2_)RhCl(CO)_2_**	2056.6/1971.5	2014.1	2031.4
**8**	2053.4/1971.6	2012.5	2030.2
**9**	2052.5/1971.5	2012.0	2029.8
**10**	2046.9/1960.7	2003.8	2023.2

**Figure 6:**
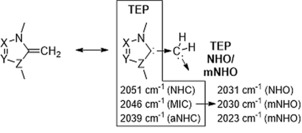
Correlation of TEP of carbene and NHO/mNHO.

**Scheme 7:**
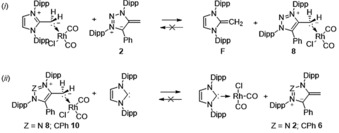
Ligand competition experiments between Rh complexes.

Interestingly, the donor properties of the new mNHOs can be correlated with the overall donor properties of the parent carbene entities. IR stretching frequencies of Rh−NHC (ν_av_: 2038 cm^−1^; TEP: 2051 cm^−1^) > MIC (ν_av_: 2032 cm^−1^; TEP: 2046 cm^−1^) > aNHC (ν_av_: 2023 cm^−1^; TEP: 2039 cm^−1^) complexes (see the Supporting Information), follow the same trend as observed for the corresponding olefins (shifted by Δ≈20 cm^−1^), but being able to participate in π‐back donation. In the extreme picture, this can be rationalized as a transfer of the electronic properties through a methylene spacer (Figure 6). Furthermore, as Tolman electronic parameter relate to the overall donor abilities (combined σ and π‐contributions), but not necessary to the metal‐ligand bond strength, we also investigated through competition experiments which ligand class would form the thermodynamically most robust metal‐ligand bond (Scheme 7).

Upon addition of mNHO **2** to (IPrCH_2_)RhCl(CO)_2_ the clean liberation of free NHO **F** and the formation of **8** was observed. Additionally, mNHO **6** can displace mNHO **2** in the coordination sphere of rhodium (see the Supporting Information). These results are in line with the formation of the thermodynamically most stable Rh‐complexes with the more donating ligands, as in these cases π‐backbonding can be neglected. However, the reaction of free IPr carbene with **8** or **10** leads to (IPr)RhCl(CO)_2_ (Scheme 7 *ii*), indicating that the overall less donating free carbene forms the stronger metal‐ligand bond as previously observed for regular NHOs.4a In this case the interplay of both σ‐donation and π‐backbonding results in a stronger metal‐ligand bond.

## Conclusion

In summary, we report a so far overlooked class of mesoionic N‐heterocyclic olefins (mNHOs), which are best described through an array of dipolar canonical resonance structures. Their basicity and donor abilities outreach traditional NHOs as shown by experiment and theory. The simple synthetic access should allow straightforward tune‐ability of their electronic and steric properties. We could prepare the first rhodium and boron coordination complexes of mNHOs and expect a larger exploration in transition metal and main group chemistry, which is under current investigation.

## Conflict of interest

The authors declare no conflict of interest.

## Supporting information

As a service to our authors and readers, this journal provides supporting information supplied by the authors. Such materials are peer reviewed and may be re‐organized for online delivery, but are not copy‐edited or typeset. Technical support issues arising from supporting information (other than missing files) should be addressed to the authors.

Supplementary
