# Supplementary material for: Stable Mesoionic N‐Heterocyclic Olefins (mNHOs)
Source: Angew Chem Int Ed Engl. 2020 Jan 27;59(14):5782–7. doi: 10.1002/anie.201914571 (PMC7154647; doi:10.1002/anie.201914571)
Supplement: Supplementary file 1 — Supplementary [file ANIE-59-5782-s001.pdf]

Angewandte Chemie
Eine Zeitschrift der Gesellschaft Deutscher Chemiker

Supporting Information

Stable Mesoionic N-Heterocyclic Olefins (mNHOs)

Max M. Hansmann, Patrick W. Antoni, and Henner Pesch*

anie_201914571_sm_miscellaneous_information.pdf

Table of Contents

1. **General procedures**.......... S-2
2. **Comparison of NHOs with mNHOs**.......... S-3
3. **Characterization data**.......... S-4
4. **NMR spectra**.......... S-14
5. **Discussion of olefinic ^{1}H NMR signals**.......... S-50
7. **Competition experiments**.......... S-56
8. **X-ray characterization data**.......... S-63
9. **IR-spectroscopy**.......... S-75
10. **UV-VIS spectroscopy**.......... S-79
11. **Computational details**.......... S-80
12. **References**.......... S-110

1. General procedures

All solvents were purified by distillation over the drying agents indicated. Reactions were carried out either under N_2 or Ar atmosphere. IR spectra were measured on Nicolet FT-7199, JASCO FT-4100 spectrometer or Bruker Vertex 60, wavenumbers in cm^{-1}. Microwave: Biotage Initatior. MS (EI): Finnigan MAT 8200 (70 eV), ESIMS: Finnigan MAT 95, accurate mass determinations: Bruker APEX III FT-MS (7 T magnet). NMR: Spectra were recorded on a Bruker AV 500, 400 or DPX 300; H and chemical shifts (δ) are given in ppm relative to TMS, coupling constants (J) in Hz. The solvent signals were used as references and the chemical shifts converted to the TMS scale. Flash chromatography was performed with Merck 60 silica gel (40-63 μm). Thin-layer chromatography (TLC) analysis was performed using Merck silica gel 60 F254 TLC plates and visualized by UV irradiation and/or ceric ammonium molybdate, $KMnO_4$ or p-anisaldehyde. All commercially available compounds (Acros, ABCR, Alfa Aesar, Aldrich, Fluorochem) were used as received. N-heterocyclic olefin IPr=CH_2 as well as IPr-RhCl$(CO)_2$ and IPrCH_2-RhCl$(CO)_2$ complexes were synthesized according to literature procedures.[1]

2. Comparison of NHOs with mNHOs

Typical reported NMR chemical shifts for *N*-heterocyclic olefins

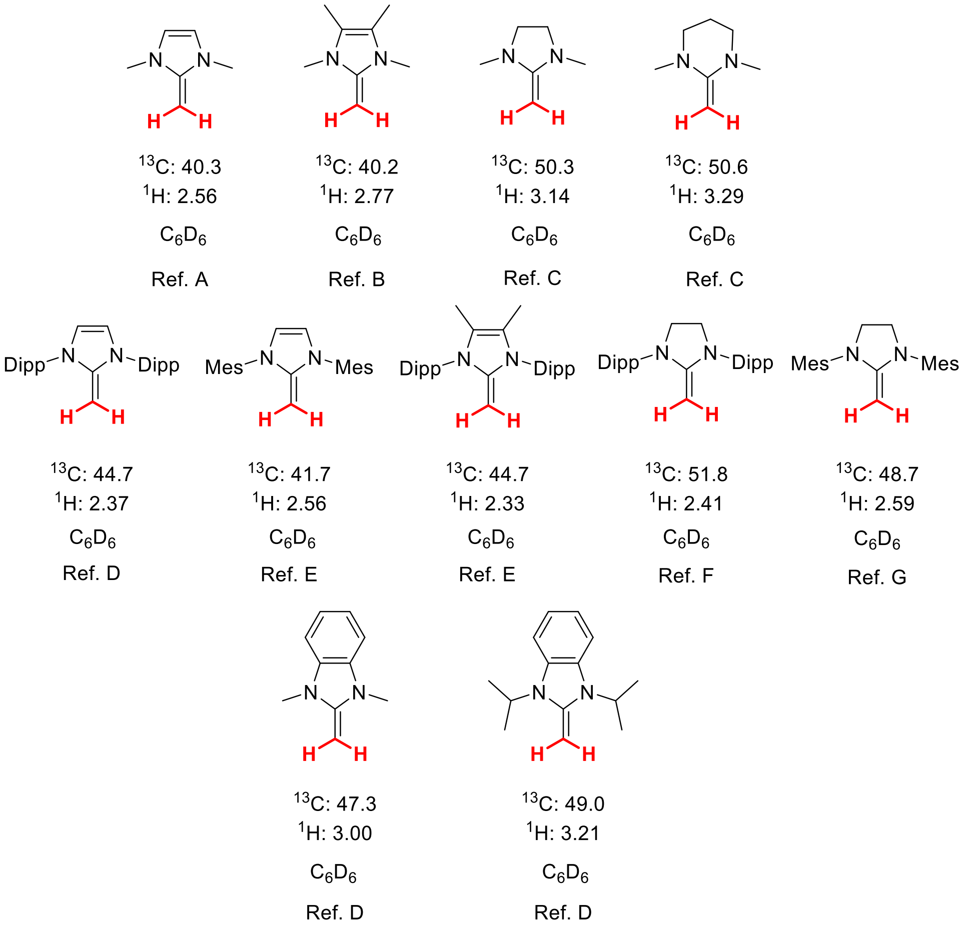

Figure S1. Summary of typical ^{1}H and ^{13}C NMR shifts for *N*-heterocyclic olefins in [ppm]. Ref. A;[2] ref. B;[3] ref. C;[4] ref. D;[5] ref. E;[1] ref. F[6]; ref. G.[7]

Typical C-C bond distances of *N*-heterocyclic olefins based on X-ray diffraction

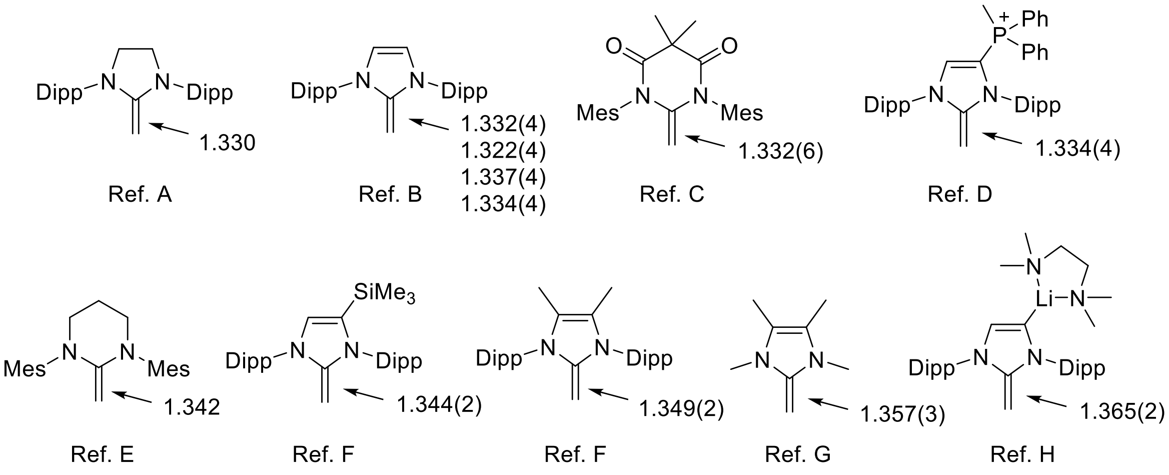

Figure S2. Summary of C-C olefinic bond distances in *N*-heterocyclic olefins based on X-ray diffraction, sorted after increasing C-C bond distance. Ref. A;[7] Ref. B;[8] Ref. C;[9] Ref. D;[10] Ref. E;[11] Ref. F;[1] Ref. G;[3] Ref. H.[12]

3. Characterization data

Synthesis of **1**

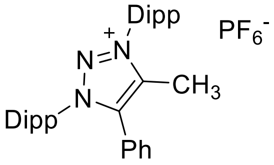

1 was synthesized according to ref.[13]: To a mixture of Dipp-triazene[14] (1.0 g, 2.7 mmol) and potassium hexafluorophosphate (650 mg, 3.53 mmol) in CH_2Cl_2 (20 mL) was added at -78 °C in the dark tertbutylhypochlorite[15] (450 µL, 4.30 mmol). The reaction was stirred in the dark at -78 °C for 30 min and then at this temperature prop-1-yn-1-ylbenzene (537 µL, 5.0 mmol) added. The reaction was slowly warmed up to room temperature over night to give a dark red colored solution. The solution was filtered and the remaining solid washed with CH_2Cl_2 (20 ml). The collected filtrates were evaporated under reduced pressure to nearly complete dryness. Et_2O (100 mL) was added and the suspension triturated under vigorous stirring. The solid is collected by filtration and washed with additional Et_2O (2x 50 mL). The remaining slightly pink solid is again dissolved in little CH_2Cl_2 (5 mL) and precipitated by additional Et_2O (100 mL). After filtration the product is dried under reduced pressure to give the desired product **1** as colorless solid (1.1 g, 1.8 mmol, 65%). Spectral data is in agreement with previous reports.[13]

^{1}H-NMR (CD_3CN, 400MHz, 298K): 7.82-7.76 (m, 1H, Ar-H), 7.70-7.64 (m, 1H, Ar-H), 7.62-7.55 (m, 3H, Ar-H), 7.54-7.48 (m, 2H, Ar-H), 7.48-7.42 (m, 4H, Ar-H), 2.48-2.36 (m, 4H, C*H*(CH_3)$_2$), 2.42 (s, 3H, triaz-C*H*$_3$), 1.32 (d, *J* = 6.8 Hz, 6H, CH(C*H*$_3$)$_2$), 1.17 (d, *J* = 6.8 Hz, 6H, CH(C*H*$_3$)$_2$), 1.13 (d, *J* = 6.8 Hz, 6H, CH(C*H*$_3$)$_2$), 1.04 (d, *J* = 6.7 Hz, 6H, CH(C*H*$_3$)$_2$); **^{13}C-NMR** (CD_3CN, 75MHz, 298K): 146.8 (Ar-*C*), 146.6 (Ar-*C*), 143.7 (Ar-*C*), 142.1 (triaz-C), 134.5 (Ar-*C*H), 134.3 (Ar-*C*H), 132.9 (Ar-*C*H), 130.8 (Ar-*C*H), 130.3 (Ar-*C*H), 130.1 (Ar-*C*), 129.3 (Ar-*C*), 126.6 (Ar-*C*H), 126.3 (Ar-*C*H), 123.0 (triaz-C), 30.0 (*C*H(CH_3)$_2$), 29.8 (*C*H(CH_3)$_2$), 26.0 (CH(*C*H$_3$)$_2$), 25.5 (CH(*C*H$_3$)$_2$), 23.5 (CH(*C*H$_3$)$_2$), 22.6 (CH(*C*H$_3$)$_2$), 10.6 (triaz-CH_3); **IR (ATR)** [cm^{-1}]: $\tilde{v}$ = 2968, 2932, 2873, 1466, 1457, 1389, 1341, 1228, 1062, 1014, 880, 695, 556; **HR-MS-ESI(+)** calc. $C_{33}H_{42}N_3^+$ $[M]^+$ 480.3373; found 480.3376.

Synthesis of **2**

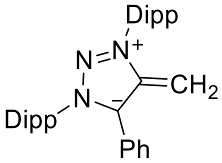

To solid triazolium salt **1** (700 mg, 1.12 mmol) and solid KHMDS (245 mg, 1.23 mmol, 1.1 eq.) cooled to -78 °C was added THF (30 mL). The reaction instantaneously turns dark purple. The

reaction was stirred for 30 min at -78 °C and then warmed up to room temperature and stirred for an additional 2 h. The solvent was removed under reduced pressure and the remaining solid extracted with pentane (2x 20 mL + 10 mL). The solvent was removed, little pentane (5 mL) added and again removed under reduced pressure to afford the product as intense dark purple solid (360 mg, 0.75 mmol, 67%).

m.p. 147 °C; **^{1}H-NMR** (C_6D_6, 500MHz, 298K): 7.45-7.41 (m, 2H, Ar-H), 7.34-7.29 (m, 1H, Ar-H), 7.27-7.24 (m, 2H, Ar-H), 7.17-7.13 (m, 1H, Ar-H), 7.00-6.96 (m, 2H, Ar-H), 6.95-6.90 (m, 2H, Ar-H), 6.81-6.76 (m, 1H, Ar-H), 3.77-3.50 (brs., 1H, triaz=CH_2), 3.56 (sept., J = 6.9 Hz, 2H, C*H*(CH_3)$_2$), 3.16 (sept., J = 6.9 Hz, 2H, C*H*(CH_3)$_2$), 3.03-2.71 (brs., 1H, triaz=CH_2), 1.47 (d, J = 6.9 Hz, 6H, CH(C*H*$_3$)$_2$), 1.30 (d, J = 6.9 Hz, 6H, CH(C*H*$_3$)$_2$), 1.20 (d, J = 6.9 Hz, 6H, CH(C*H*$_3$)$_2$), 0.90 (d, J = 6.9 Hz, 6H, CH(C*H*$_3$)$_2$); **^{1}H-NMR** (d^8-THF, 400MHz, 223K): 7.56-7.51 (m, 1H, Ar-H), 7.50-7.45 (m, 1H, Ar-H), 7.41-7.36 (m, 4H, Ar-H), 7.20-7.13 (m, 4H, Ar-H), 7.08-7.02 (m, 1H, Ar-H), 3.18 (sept., J = 6.7 Hz, 2H, C*H*(CH_3)$_2$), 3.01 (s, 1H, triaz=CH_2), 2.91 (sept., J = 6.7 Hz, 2H, C*H*(CH_3)$_2$), 2.16 (s, 1H, CH_2, triaz=CH_2), 1.32 (d, J = 6.7 Hz, 6H, CH(C*H*$_3$)$_2$), 1.19 (d, J = 6.7 Hz, 12H, CH(C*H*$_3$)$_2$), 0.95 (d, J = 6.7 Hz, 6H, CH(C*H*$_3$)$_2$); **^{13}C-NMR** (C_6D_6, 500MHz, 298K): 148.6 (Ar-C), 147.4 (triaz-C), 146.0 (Ar-C), 133.3 (Ar-C), 133.1 (Ar-C), 131.1 (Ar-CH), 130.5 (Ar-CH), 129.8 (Ar-C), 128.5 (Ar-CH), 126.1 (Ar-CH), 125.7 (Ar-CH), 124.8 (Ar-CH), 124.6 (Ar-CH), 120.0 (triaz-C), 45.9 (triaz=CH_2), 29.2 (*C*H(CH_3)$_2$), 25.9 (CH(*C*H$_3$)$_2$), 24.7 (CH(*C*H$_3$)$_2$), 23.7 (CH(*C*H$_3$)$_2$), 22.7 (CH(*C*H$_3$)$_2$); **^{13}C-NMR** (d^8-THF, 400MHz, 223K): 148.7 (Ar-C), 147.2 (triaz-C), 146.1 (Ar-C), 133.3 (Ar-CH), 133.1 (Ar-CH), 132.1 (Ar-CH), 131.2 (Ar-C), 129.9 (Ar-C), 129.1 (Ar-CH), 126.8 (Ar-C), 126.0 (Ar-CH), 125.6 (Ar-CH), 125.4 (Ar-CH), 120.0 (triaz-C), 44.3 (triaz=CH_2), 29.9 (*C*H(CH_3)$_2$), 29.6 (*C*H(CH_3)$_2$), 26.3 (CH(*C*H$_3$)$_2$), 24.9 (CH(*C*H$_3$)$_2$), 23.7 (CH(*C*H$_3$)$_2$), 23.0 (CH(*C*H$_3$)$_2$); **IR (ATR)** [cm^{-1}]: ṽ = 2961, 2928, 2867, 1649, 1593, 1579, 1506, 1466, 1445, 1384, 1363, 1342, 1284, 1155, 1060, 936, 799, 755, 694, 587; **HR-MS-ESI(+)** calc. $C_{33}H_{42}N_3^+$ [M+H]$^+$ 480.3373; found 480.3377; **UV-VIS** [nm]: in THF: 350 nm (ε = 6236 cm^{-1}M^{-1}); 535 nm (ε = 7062 cm^{-1}M^{-1}).

Synthesis of **3**

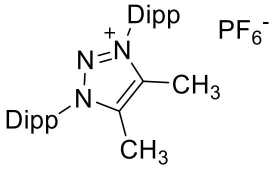

3 was synthesized according to ref.[13]: To a mixture of Dipp-triazene[14] (0.75 g, 2.1 mmol) and potassium hexafluorophosphate (502 mg, 2.73 mmol) in CH_2Cl_2 (15 mL) was added at -78°C in the dark tertbutylhypochlorite[15] (350 µL, 2.7 mmol). The reaction was stirred in the dark at -78°C for 30 min and then at this temperature but-2-yne (537 µL, 5.0 mmol) was added. The reaction was slowly warmed up to room temperature overnight to give a dark red colored solution. The solution was filtered and the remaining solid washed with CH_2Cl_2 (20 ml). The collected filtrates were evaporated

under reduced pressure to nearly complete dryness. Et_2O (100 mL) was added and the suspension triturated under vigorous stirring. The solid was collected by filtration and washed with additional Et_2O (2x 50 mL). The remaining slightly brown solid is again dissolved in little CH_2Cl_2 (5 mL) and precipitated by additional Et_2O (100 mL). After filtration the product is dried under reduced pressure to give the desired product **3** as colorless solid (0.66 g, 1.6 mmol, 77%).

^{1}H-NMR (DMSO-d_6, 400MHz, 298K): 7.70 (t, *J* = 7.8 Hz, 2H, Ar-H), 7.53 (d, *J* = 7.8 Hz, 4H, Ar-H), 2.27 (s, 6H, triaz-CH_3), 2.22 (hept, *J* = 6.8 Hz, 4H, C*H*$(CH_3)_2$), 1.18 (d, *J* = 6.7 Hz, 12H, CH(C*H*$_3$)$_2$), 1.00 (d, *J* = 6.8 Hz, 12H, CH(C*H*$_3$)$_2$); **^{13}C {^{1}H} NMR** (DMSO-d_6, 101 MHz, 298K): 145.3 (Ar-*C*), 141.5 (triaz-C), 133.3 (Ar-*C*H), 128.1 (Ar-C), 125.4 (Ar-*C*H), 28.3 (*C*H$(CH_3)_2$), 24.8 (CH(*C*H$_3$)$_2$), 23.0 (CH(*C*H$_3$)$_2$), 9.3 (triaz-CH_3); **^{19}F-NMR** (DMSO-d_6, 376 MHz, 298K): -70.19 (d, *J* = 711.2 Hz, PF_6^-); **^{31}P-NMR** (162 MHz, DMSO-*d*$_6$, 298K) -144.22 (hept, *J* = 711.3 Hz, PF_6^-); **IR (ATR)** [cm^{-1}]: ṽ = 2970, 2933, 2873, 1598, 1466, 1389, 1366, 1340, 1227, 1061, 1015, 876, 831, 806, 785, 762, 741, 599, 556, 438, 421; **ESI(+)** calc. $C_{28}H_{40}N_3^+$ $[M]^+$ 418.3217 found 418.3217.

Synthesis of **4**

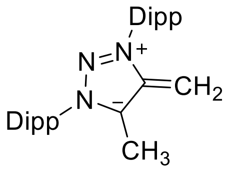

To solid triazolium salt **3** (400 mg, 0.71 mmol) and solid KHMDS (155 mg, 0.78 mmol, 1.1 eq.) cooled to -78°C was added THF (20 mL). The reaction mixture instantly turns orange/red. The reaction was stirred for 15 min at -78 °C and then warmed up to room temperature and stirred for an additional 1 h. The solvent was removed under reduced pressure and the remaining solid extracted with pentane (2x 20 mL). The solvent was removed under reduced pressure, to afford the product as orange solid (205 mg, 0.59 mmol, 69%).

m.p. 149 °C; **^{1}H-NMR** (C_6D_6, 400MHz, 298K): 7.35 – 7.21 (m, 3H, Ar-H), 7.18 - 7.15 (m, 1H, Ar-H), 7.01 (d, *J* = 7.8 Hz, 2H, Ar-H), 3.51 (hept, *J* = 6.9 Hz, 2H, C*H*$(CH_3)_2$), 2.98 (hept, *J* = 6.9 Hz, 2H, C*H*$(CH_3)_2$), 2.92 (d, *J* = 1.5 Hz, 1H, triaz=CH_2), 2.76 (d, *J* = 1.5 Hz, 1H, triaz=CH_2), 1.65 (s, 3H, triaz-CH_3), 1.44 (d, *J* = 6.9 Hz, 6H, CH(C*H*$_3$)$_2$), 1.29 (d, *J* = 6.9 Hz, 6H, CH(C*H*$_3$)$_2$), 1.20 (d, *J* = 6.8 Hz, 6H, CH(C*H*$_3$)$_2$), 1.07 (d, *J* = 6.9 Hz, 6H, CH(C*H*$_3$)$_2$); **^{13}C {^{1}H} NMR** ($CDCl_3$, 101 MHz, 298K): 148.7 (triaz-C), 148.6 (Ar-C), 146.2 (Ar-C), 133.7 (Ar-C), 131.9 (Ar-C), 131.0 (Ar-CH), 130.1 (Ar-CH), 124.6 (Ar-CH), 124.3 (Ar-CH), 117.5 (triaz-C), 42.5 (triaz=CH_2), 29.0 (*C*H$(CH_3)_2$), 28.8 (*C*H$(CH_3)_2$), 25.5 (CH(*C*H$_3$)$_2$), 24.6 (CH(*C*H$_3$)$_2$), 23.5 (CH(*C*H$_3$)$_2$), 23.4 (CH(*C*H$_3$)$_2$), 9.6 (triaz-CH_3); **IR (ATR)** [cm^{-1}]: ṽ = 2960, 2926, 2867, 1581, 1464, 1376, 1361, 1344, 1264, 1134, 1100, 1059, 1009, 936, 800, 790, 768, 742, 597, 512, 439; **HR-MS-ESI(+)** calc. 418.3217; found 418.3219; **UV-VIS** [nm]: in THF: 327 nm (ε = 2302 $cm^{-1}M^{-1}$); 468 nm (ε = 2590 $cm^{-1}M^{-1}$).

Synthesis of **5**

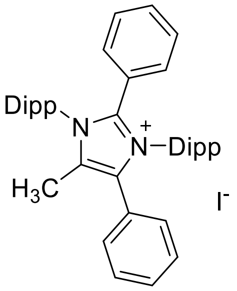

To a mixture of solid **aNHC-H$^+$ HCl$_2^-$**[16] (870 mg, 1.42 mmol, 1.0 eq.) and solid KHMDS (565 mg, 2.84 mmol, 2.0 eq.) was added precooled THF (30 mL) at -78 °C. After stirring at -78 °C for 5 minutes the solution was warmed up to room temperature and stirred for 1 h. The solvent was removed *in vacuo*. The green residue was extracted with pentane (2x 20 mL), the solvent removed under reduced pressure and the resulting green/yellow solid dissolved in 20 mL Et_2O. The green solution was added to a solution of methyl iodide (265 µL, 4.25 mmol, 3.0 eq.) in Et_2O (20 mL) at -30 °C. After stirring the white suspension for 10 minutes at -30 °C and 15 min at room temperature the supernatant was removed by filtration and the colorless solid was washed with Et_2O (3x 15 mL). The remaining solvent was removed under reduced pressure to afford **5** as a colorless solid (750 mg, 1.1 mmol, 78%).

m.p. >300 °C; **^{1}H NMR** (500 MHz, $CDCl_3$, 298K): 7.69 (t, *J* = 7.8 Hz, 1H, Ar-H), 7.57 (t, *J* = 7.8 Hz, 1H, Ar-H), 7.45-7.40 (m, 6H, Ar-H), 7.30-7.17 (m, 6H, Ar-H), 6.95-6.92 (m, 2H, Ar-H), 2.47 (sept., *J* = 6.8 Hz, 2H, $CH(CH_3)_2$), 2.42 (s, 3H, imidazol-CH_3), 2.41 (sept., *J* = 6.8 Hz, 2H, $CH(CH_3)_2$), 1.32 (d, *J* = 6.8 Hz, 6H, $CH(CH_3)_2$), 0.96 (d, *J* = 6.8 Hz, 6H, $CH(CH_3)_2$), 0.85 (d, *J* = 6.8 Hz, 12H, $CH(CH_3)_2$); **^{13}C {^{1}H} NMR** (125 MHz, CD_3CN, 298K): 145.1 (Ar-C), 144.8 (Ar-C), 144.5 (imidazol-C), 133.2 (Ar-CH), 133.1 (Ar-CH), 132.8 (Ar-CH), 130.8 (Ar-CH), 130.7 (Ar-C), 130.2 (Ar-CH), 129.6 (Ar-CH), 129.5 (Ar-CH), 129.3 (Ar-CH), 128.8 (Ar-C), 128.2 (Ar-C), 126.3 (Ar-CH), 126.2 (Ar-CH), 124.4 (imidazol-C), 120.9 (imidazol-C)), 29.4 ($CH(CH_3)_2$), 29.3 ($CH(CH_3)_2$), 25.1 ($CH(CH_3)_2$), 24.1 ($CH(CH_3)_2$), 23.8 ($CH(CH_3)_2$), 23.3 ($CH(CH_3)_2$), 11.1 (imidazole-CH_3); **IR (ATR)** [cm^{-1}]: ṽ = 2956, 2927, 2868, 1472, 1444, 1423, 1386, 1363, 1321, 1176, 1158, 1057, 1042, 1018, 927, 812, 793, 773, 760, 745, 727, 671, 615, 545, 513, 486, 434; **ESI(+)** calc. $C_{40}H_{47}N_2^+$ $[M]^+$ 555.3734 found 555.3737.

Synthesis of **6**

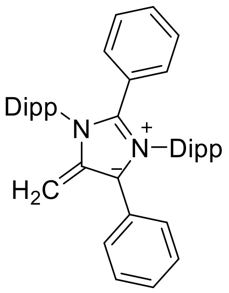

To a mixture of solid **5** (250 mg, 0.37 mmol, 1.0 eq.) and KHMDS (77 mg, 0.38 mmol, 1.05 eq.) was added pre-cooled THF at -78 °C. The reaction mixture turned green instantly. The reaction was stirred for 30 minutes at -78 °C and 1 h at room temperature. The solvent was removed under reduced pressure and the remaining green solid was extracted with toluene (2x 10 ml). After removing the solvent the crude product was washed with cold (-20 °C) pentane (5 mL) and dried under reduced pressure to afford **6** as a dark green solid (120 mg, 0.22 mmol, 59%).

m.p. 132 °C; **^{1}H NMR** (500 MHz, d^8-THF, 233K): 7.55-7.49 (m, 1H, Ar-H), 7.46-7.41 (m, 1H, Ar-H), 7.35-7.28 (m, 4H, Ar-H), 6.97-6.87 (m, 5H, Ar-H), 6.87-6.81 (m, 2H, Ar-H), 6.72-6.67 (m, 1H, Ar-H), 6.63-6.58 (m, 2H, Ar-H), 3.21 (s, 1H, imidazol=CH_2), 3.25-3.16 (m, 2H, C*H*$(CH_3)_2$), 3.05 (sept., *J* = 6.8 Hz, 2H, C*H*$(CH_3)_2$), 2.04 (s, 1H, imidazol=CH_2), 1.34 (d, *J* = 6.4 Hz, 6H, CH(C*H*$_3)_2$), 0.98 (d, *J* = 6.4 Hz, 6H, CH(C*H*$_3)_2$), 0.85 (d, *J* = 6.4 Hz, 6H, CH(C*H*$_3)_2$), 0.80 (d, *J* = 6.4 Hz, 6H, CH(C*H*$_3)_2$); **^{1}H NMR** (400 MHz, C_6D_6, 298K): 7.40-7.35 (m, 2H, Ar-H), 7.28-7.22 (m, 1H, Ar-H), 7.20-7.15 (m, 3H, Ar-H), 7.03-6.97 (m, 4H, Ar-H), 6.83-6.79 (m, 2H, Ar-H), 6.76-6.71 (m, 1H, Ar-H), 6.60-6.55 (m, 2H, Ar-H), 6.51-6.46 (m, 1H, Ar-H), 3.98 (brs., 1H, imidazol=CH_2), 3.57 (sept., *J* = 7.0 Hz, 2H, C*H*$(CH_3)_2$), 3.32 (sept., *J* = 7.0 Hz, 2H, C*H*$(CH_3)_2$), 2.74 (brs., 1H, imidazol =CH_2), 1.51 (d, *J* = 6.8 Hz, 6H, CH(C*H*$_3)_2$), 1.10 (d, *J* = 6.8 Hz, 6H, CH(C*H*$_3)_2$), 0.89 (d, *J* = 6.8 Hz, 6H, CH(C*H*$_3)_2$), 0.88 (d, *J* = 6.8 Hz, 6H, CH(C*H*$_3)_2$); **^{13}C {^{1}H} NMR** (125 MHz, d^8-THF, 233K): 147.7 (Ar-C), 147.0 (imidazo-C), 146.0 (Ar-C), 133.8 (Ar-C), 133.4 (Ar-C), 133.3 (Ar-C), 131.5 (Ar-CH), 130.6 (Ar-CH), 130.3 (Ar-C), 128.5 (Ar-CH), 128.3 (Ar-CH), 128.3 (Ar-CH), 127.4 (Ar-CH), 126.2 (Ar-CH), 126.1 (Ar-CH), 125.5 (Ar-CH), 125.0 (Ar-C), 122.2 (Ar-CH), 107.0 (imidazo-C), 46.4 (imidazol=CH_2), 29.8 (*C*H$(CH_3)_2$), 29.6 (*C*H$(CH_3)_2$), 25.4 (CH(*C*H$_3)_2$), 24.1 (CH(*C*H$_3)_2$), 23.8 (CH(*C*H$_3)_2$), 23.7 (CH(*C*H$_3)_2$); **^{13}C {^{1}H} NMR** (100 MHz, C_6D_6, 298K): 147.6 (Ar-C), 147.2 (imidazo-C), 145.9 (Ar-C), 133.9 (Ar-C), 133.4 (Ar-C), 133.1 (Ar-CH), 130.7 (Ar-CH), 130.0 (Ar-CH), 129.2 (Ar-CH), 127.1 (Ar-CH), 126.0 (Ar-CH), 125.6 (Ar-CH), 125.5 (Ar-CH), 125.3 (Ar-CH), 124.9 (Ar-C), 122.4 (Ar-CH), 107.7 (imidazo-C), 48.0 (imidazol=CH_2), 29.3 (*C*H$(CH_3)_2$), 29.0 (*C*H$(CH_3)_2$), 25.2 (CH(*C*H$_3)_2$), 23.7 (CH(*C*H$_3)_2$), 23.5 (CH(*C*H$_3)_2$); **IR (ATR)** [cm^{-1}]: $\tilde{v}$ = 2956, 2927, 2868, 1472, 1444, 1423, 1386, 1363, 1321, 1176, 1158, 1057, 1042, 1018, 927, 812, 793, 773, 760, 745, 727, 671, 615, 545, 513, 486, 434; **HR-MS-ESI(+)** calc. $C_{40}H_{47}N_2^+$ $[M+H]^+$ 555.3734, found 555.3737. **UV-VIS** [nm]: in THF: 431 nm (ε = 1880 cm^{-1}M^{-1}); 669 nm (ε = 851 cm^{-1}M^{-1}).

Synthesis of **7**

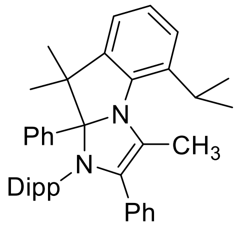

A solution of **6** (50 mg, 0.09 mmol) in THF (25 mL) was heated to 45 °C for 3 days upon which the solution turned blue after one day. The solvent was removed under reduced pressure, the remaining blue solid redissolved in pentane and filtrated over a short silica pad (in a nitrogen filled glovebox). The solution was collected until it started to turn yellow. Removing the solvent under reduced pressure afforded **7** as a colorless solid (18 mg, 0.03 mmol, 36%).

m.p. 169 °C; **^{1}H-NMR** (C_6D_6, 400MHz, 298K): 7.60-7.30 (brs, 1H, Ar-H), 7.16-7.14 (m, 2H, Ar-H), 7.12-6.95 (m, 8H, Ar-H), 6.93-6.88 (m, 2H, Ar-H), 6.87-6.78 (brs., 1H, Ar-H), 6.77-6.72 (m, 1H, Ar-H), 6.70 (dd, *J* = 7.3 Hz, 1.2 Hz, 1H, Ar-H), 4.37 (sept., *J* = 6. 9Hz, 1H, C*H*(CH_3)$_2$)), 3.87-3.77 (m, 2H, C*H*(CH_3)$_2$)), 2.11 (s, 3H, *gem*-CH_3), 1.87 (s, 3H, (imidaz)-CH_3), 1.47 (d, *J* = 6.9 Hz, 3H, CH(C*H*$_3$)$_2$), 1.37 (d, *J* = 7.0 Hz, 3H, CH(C*H*$_3$)$_2$), 1.36 (d, *J* = 7.0 Hz, 3H, CH(C*H*$_3$)$_2$), 1.31 (d, *J* = 6.8 Hz, 3H, CH(C*H*$_3$)$_2$), 1.09 (s, 3H, *gem*-CH_3), 0.96 (d, *J* = 6.8 Hz, 3H, CH(C*H*$_3$)$_2$), 0.38 (d, *J* = 6.6 Hz, 3H, CH(C*H*$_3$)$_2$); **^{13}C-NMR** (C_6D_6, 100MHz, 298K): 151.8 (Ar-C), 151.0 (Ar-C), 144.5 (Ar-C), 144.5 (Ar-C), 141.2 (Ar-C), 139.1 (Ar-C), 137.3 (Ar-C), 134.2 (Ar-C), 130.6 (Ar-CH), 130.3 (imidaz-C), 128.3 (HSQC, Ar-CH), 128.2 (imidaz-C), 128.2 (HSQC, Ar-CH), 128.1 (HSQC, Ar-CH), 127.1 (Ar-CH), 126.6 (Ar-CH), 126.4 (Ar-CH), 125.4 (Ar-CH), 125.2 (Ar-CH), 123.9 (Ar-CH), 120.7 (Ar-CH), 102.7 (N*C*N), 53.9 (Me-C(im)-Me), 30.5 (*gem*-*C*H$_3$), 29.1 (*C*H(CH_3)$_2$), 28.1 (*C*H(CH_3)$_2$), 27.7 (*C*H(CH_3)$_2$), 25.6 (*gem*-*C*H$_3$), 25.5 (CH(*C*H$_3$)$_2$), 25.1 (CH(*C*H$_3$)$_2$), 25.0 (CH(*C*H$_3$)$_2$), 24.4 (CH(*C*H$_3$)$_2$), 24.3 (CH(*C*H$_3$)$_2$), 23.1 (CH(*C*H$_3$)$_2$), 11.7 ((imidaz)-*C*H$_3$); **IR (ATR)** [cm^{-1}]: ṽ = 3056, 2961, 2926, 2866, 1660, 1628, 1598, 1494, 1462, 1443, 1382, 1362, 1299, 1277, 1258, 1238, 1210, 1154, 1131, 1062, 1033, 984, 969, 930, 903, 876, 808, 792, 767, 754, 732, 718, 699, 662, 634, 609, 589, 552, 526, 516, 497, 470, 442; **HR-MS-ESI(+)** calc. $C_{40}H_{47}N_2^+$ [M+H]$^+$ 555.3734, found 555.3716; $C_{40}H_{47}N_2Na^+$ [M+Na]$^+$ 577.3553, found 577.3542.

Synthesis of **8**

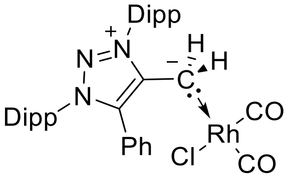

To a solution of [RhCl(CO)$_2$]$_2$ (24 mg, 0.06 mmol, 1.0 eq.) in toluene (3 mL) was added dropwise a solution of **2** (60 mg, 0.13 mmol, 2.0 eq.) in toluene (2 mL). Instant decolorization of the solution of **2** was observed upon addition. After stirring the colorless, slightly yellow solution for 1 h at room temperature, pentane (15 mL) was added to precipitate the crude product. The supernatant was decanted off and the remaining colorless solid was washed with pentane (3 x 5 mL). The solvent was removed under reduced pressure to afford **8** as beige solid (41 mg, 0.06 mmol, 49%).

^{1}H-NMR ($CDCl_3$, 400MHz, 298K): 7.68-7.62 (m, 1H, Ar-H), 7.61-7.53 (m, 3H, Ar-H), 7.47-7.43 (m, 2H, Ar-H), 7.38-7.33 (m, 3H, Ar-H), 7.32-7.28 (m, 2H, Ar-H), 2.29-2.68 (m, 4H, C*H*(CH_3)$_2$), 2.21 (s,

2H, CH_2-Rh, $^3J_{Rh-H}$ < 3 Hz), 1.50 (d, J = 6.5 Hz, 6H, CH(CH_3)$_2$), 1.17 (d, J = 6.5 Hz, 12H, CH(CH_3)$_2$), 1.05 (d, J = 6.5 Hz, 6H, CH(CH_3)$_2$); **^{13}C-NMR** ($CDCl_3$, 100MHz, 298K): 186.8 (d, $^1J_{CRh}$ = 79.3 Hz, CO), 184.6 (d, $^1J_{CRh}$ = 57.0 Hz, CO), 157.7 (triazole-C), 146.2 (Ar-C), 146.0 (Ar-C), 136.9 (triazole-C), 132.3 (Ar-CH), 132.3 (Ar-C), 130.5 (Ar-CH), 130.2 (Ar-CH), 130.0 (Ar-CH), 128.8 (Ar-CH), 125.0 (Ar-CH), 124.8 (Ar-CH), 29.3 (*C*H(CH_3)$_2$), 29.0 (*C*H(CH_3)$_2$), 25.9 (CH(*C*H_3)$_2$), 25.8 (CH(*C*H_3)$_2$), 23.5 (CH(*C*H_3)$_2$), 22.6 (CH(*C*H_3)$_2$), 8.3 (d, $^1J_{CRh}$ = 17.5 Hz, CH_2-Rh); **IR (ATR)** [cm^{-1}]: ṽ = 2968, 2931, 2870, 2046, 1969, 1547, 1493, 1468, 1409, 1389, 1362, 1342, 1278, 1199, 1060, 1035, 1017, 987, 937, 803, 778, 767, 757, 732, 695, 677, 612, 588, 521, 490, 462, 448, 431, 421; **HR-MS-ESI(+)** calc. $C_{35}H_{41}N_3O_2Rh^+$ [M-Cl]$^+$ 638.2248; found 638.2246; $C_{34}H_{41}N_3ORh^+$ [M-Cl-CO]$^+$ 610.2299; found 610.2295; **EA** calc. for [$C_{35}H_{41}ClN_3O_2Rh$] C: 62.36; H: 6.13; N: 6.23; found C: 62.52; H: 6.44; N: 5.89.

Synthesis of **9**

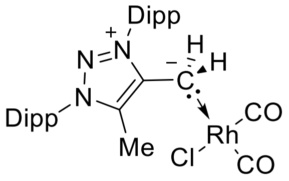

To a stirred solution of [RhCl(CO)$_2$]$_2$ (23 mg, 0.06 mmol, 1.0 eq.) in toluene (1 mL) was added a solution of **4** (50 mg, 0.12 mmol, 2.0 eq.) in toluene (5 mL). After stirring the reaction mixture for 10 min the solvent was removed under reduced pressure. The resulting beige solid was dissolved in pentane (10 mL). The suspension was filtered over a short Celite pad and the solvent was removed under reduced pressure to afford **9** as off-white solid (43 mg, 0.07 mmol, 58%).

^{1}H-NMR ($CDCl_3$, 400MHz, 298K): 7.65 – 7.55 (m, 2H, Ar-H), 7.39 (d, J = 4.3 Hz, 2H, Ar-H), 7.37 (d, J = 4.3 Hz, 2H, Ar-H), 2.57 (hept, J = 6.8 Hz, 2H, C*H*(CH_3)$_2$), 2.49 (hept, J = 6.8 Hz, 2H, C*H*(CH_3)$_2$), 2.32 (s, 3H, imidazole-CH_3), 1.96 (d, $^3J_{Rh-H}$ = 2.6 Hz, 2H, CH_2-Rh), 1.44 (d, J = 6.8 Hz, 6H, CH(CH_3)$_2$), 1.27 (d, J = 6.8 Hz, 6H, CH(CH_3)$_2$), 1.16 (d, J = 6.8 Hz, 6H, CH(CH_3)$_2$), 1.10 (d, J = 6.9 Hz, 6H, CH(CH_3)$_2$); **^{13}C {^{1}H} NMR** ($CDCl_3$, 101 MHz, 298K): 186.5 (d, $^1J_{CRh}$ = 79.3 Hz, CO), 184.7 (d, $^1J_{CRh}$ = 56.6 Hz, CO), 156.6 (d, J = 1.9 Hz, triazole-C), 146.2 (Ar-C), 146.1 (Ar-C), 135.7 (triazole-C), 132.5 (Ar-CH), 132.2 (Ar-CH), 130.0 (Ar-C), 129.5 (Ar-C), 124.9 (Ar-CH), 124.9 (Ar-CH), 29.3 (*C*H(CH_3)$_2$), 28.8 (*C*H(CH_3)$_2$), 25.8 (CH(*C*H_3)$_2$), 25.1 (CH(*C*H_3)$_2$), 23.7 (CH(*C*H_3)$_2$), 23.5 (CH(*C*H_3)$_2$), 11.2 (triazole-CH_3), 7.0 (d, $^1J_{CRh}$ = 17.5 Hz, CH_2-Rh); **IR (ATR)** [cm^{-1}]: ṽ = 2958, 2927, 2869, 2046, 1962, 1572, 1465, 1386, 1372, 1344, 1193, 1161, 1061, 1038, 1011, 937, 803, 771, 747, 608, 590, 522, 470, 434; **HR-MS-ESI(+)** calc. $C_{30}H_{39}N_3O_2Rh^+$ [M-Cl]$^+$ 576.2092; found 576.2095; **EA** calc. for [$C_{30}H_{39}ClN_3O_2Rh$] C: 58.88; H: 6.42; N: 6.87; found 58.54; H: 6.36; N: 6.78.

Synthesis of **10**

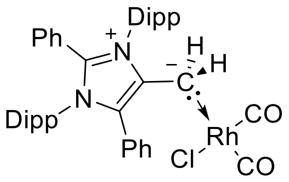

To a stirred solution of $[RhCl(CO)_2]_2$ (18 mg, 0.05 mmol, 1.0 eq.) in toluene (1 mL) was added a solution of **6** (50 mg, 0.09 mmol, 2.0 eq.) in toluene (5 mL). After stirring the reaction mixture for 10 min the solvent was removed under reduced pressure. The resulting brown solid was dissolved in Et_2O (5 mL) and filtered over a short Celite pad. To the resulting yellow solution pentane (15 mL) was added and the mixture cooled to -40 °C to afford **10** as slightly yellow crystals (25 mg, 0.03 mmol, 37%).

^{1}H-NMR ($CDCl_3$, 400MHz, 298K): 7.59 – 7.52 (m, 3H, Ar-H), 7.47 (t, J = 7.8 Hz, 1H, Ar-H), 7.33 (s, 2H, Ar-H), 7.28 – 7.25 (m, 1H, Ar-H), 7.25 – 7.19 (m, 3H, Ar-H), 7.17 (d, J = 7.8 Hz, 2H, Ar-H), 7.05 (t, J = 8.4, 2H, Ar-H), 6.81 (d, J = 7.5 Hz, 2H, Ar-H), 2.87 (hept, J = 6.8 Hz, 2H, CH(CH_3)$_2$), 2.77 (hept, J = 6.7 Hz, 2H, CH(CH_3)$_2$), 2.27 (d, $^3J_{Rh\text{-}H}$ = 2.8 Hz, 2H, CH_2-Rh), 1.48 (d, J = 6.7 Hz, 6H, CH(CH_3)$_2$), 0.81 (d, J = 6.7 Hz, 6H, CH(CH_3)$_2$), 0.78 – 0.72 (m, 12H, CH(CH_3)$_2$); **^{13}C {^{1}H} NMR** ($CDCl_3$, 101 MHz, 298K): 188.09 (d, J = 81.7 Hz, CO), 185.00 (d, J = 55.4 Hz, CO), 150.1 (triazole-C), 146.0 (Ar-C), 145.4 (Ar-C), 140.0 (Ar-C), 131.6 (Ar-CH), 131.6 (Ar-CH), 130.9 (Ar-C), 130.5 (Ar-CH), 130.3 (Ar-CH), 130.0 (Ar-CH), 129.8 (Ar-C), 128.5 (Ar-CH), 128.2 (Ar-CH), 128. (Ar-CH), 127.8 (Ar-C), 125.8 (Ar-CH), 125.6 (Ar-CH), 125.1 (triazole-C), 123.1 (Ar-C), 28.8 (CH(CH_3)$_2$), 28.6 (CH(CH_3)$_2$), 25.9 (CH(CH$_3$)$_2$), 23.8 (CH(CH$_3$)$_2$), 23.7 (CH(CH$_3$)$_2$), 23.5 (CH(CH$_3$)$_2$), 12.77 (d, $^1J_{CRh}$ = 18.2 Hz, CH_2-Rh); **IR (ATR)** [cm^{-1}]: $\tilde{\nu}$ = 2968, 2931, 2870, 2046, 1969, 1547, 1493, 1468, 1409, 1389, 1362, 1342, 1278, 1199, 1060, 1035, 1017, 987, 937, 803, 778, 767, 757, 732, 695, 677, 612, 588, 521, 490, 462, 448, 431, 421; **HR-MS-ESI(+)** calc. $C_{40}H_{45}ClN_2Rh^+$ [M-2CO-H]$^+$ 691.2321; found 691.2320; **EA** calc. for [$C_{42}H_{46}ClN_2O_2Rh$] C: 67.33; H: 6.19; N: 3.74; found C: 67.55; H: 6.33; N: 3.57.

Synthesis of **11**

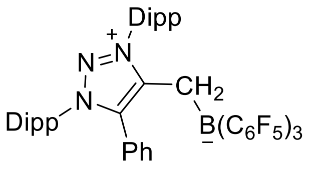

To a stirred solution of $B(C_6F_5)_3$ (43 mg, 0.08 mmol, 1.0 eq.) in toluene (1 mL) was added a solution of **2** (40 mg, 0.08 mmol, 1.0 eq.) in toluene (1 mL). A colorless precipitate formed immediately. After 1 h pentane was added (5 mL) and the colorless precipitate was filtered off and washed with pentane

(5 mL). The product was dried under reduced pressure to afford **11** as colorless solid (59 mg, 0.06 mmol, 71%).

m.p. 280 °C (decomposition); **^{1}H-NMR** ($CDCl_3$, 400MHz, 298K): 7.71 (t, J = 7.8 Hz, 1H, Ar-H), 7.54 (t, J = 7.8 Hz, 1H, Ar-H), 7.39 (d, J = 7.8 Hz, 2H, Ar-H), 7.36-7.30 (m, 1H, Ar-H), 7.29-7.21 (m, 4H, Ar-H), 6.97-6.92 (m, 2H, Ar-H), 3.50 (brs, 2H, CH_2-BR_3), 2.50 (sept., J = 6.7 Hz, 2H, C*H*(CH_3)$_2$), 2.25 (sept., J = 6.7 Hz, 2H, C*H*(CH_3)$_2$), 1.21-1.15 (m, 12H, CH(CH_3)$_2$), 1.11 (d, J = 6.8 Hz, 6H, CH(CH_3)$_2$), 1.04 (d, J = 6.8 Hz, 6H, CH(CH_3)$_2$); **^{19}F-NMR** ($CDCl_3$, 282 MHz, 298K): -131.8 (d, J = 20.6 Hz, 6F, *o*-C_6F_5), -161.8 (t, J = 20.6 Hz, 3F, *p*-C_6F_5), -165.3 (t, J = 21.6 Hz, 6F, *m*-C_6F_5); **^{11}B-NMR** ($CDCl_3$, 96 MHz, 298K): -13.6 (s); **^{13}C {^{1}H} NMR** ($CDCl_3$, 125 MHz, 298K): 151.9 (triazole-C), 148.1 (d, J_{CF} = 241 Hz, C_6F_5), 145.7 (Ar-C), 139.9 (triazole-C), 138.6 (d, J_{CF} = 245 Hz, C_6F_5), 136.6 (d, J_{CF} = 240 Hz, C_6F_5), 133.0 (Ar-CH), 132.8 (Ar-CH), 130.7 (Ar-CH), 129.8 (Ar-C), 129.2 (Ar-C), 129.0 (Ar-C), 128.7 (Ar-CH), 128.4 (Ar-C), 128.1 (Ar-CH), 124.9 (Ar-CH), 124.9 (Ar-CH), 122.8 (Ar-C), 29.6 (*C*H(CH_3)$_2$), 27.7 (CH(*C*H_3)$_2$), 26.2 (CH(*C*H_3)$_2$), 22.7 (CH(*C*H_3)$_2$), 21.3 (CH(CH_3)$_2$), 19.6 (q, J_{CB} = 40 Hz, CH_2-BR_3); **IR (ATR)** [cm^{-1}]: ṽ = 2975, 1644, 1558, 1541, 1510, 1456, 1456, 1390, 1339, 1273, 1204, 1081, 983, 974, 920, 827, 752, 697, 675; **HR-MS-ESI(+)** calc. $C_{51}H_{41}BF_{15}N_3Na^+$ $[M+Na]^+$ 1014.3046; found 1014.3055; **EA** calc. for [$C_{51}H_{41}BF_{15}N_3$] C: 61.77; H: 4.17; N: 4.24; found C: 61.61; H: 4.18; N: 4.21.

Synthesis of **12**

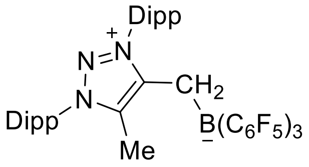

To a stirred solution of $B(C_6F_5)_3$ (25 mg, 0.05 mmol, 1.0 eq.) in 5 mL pentane was slowly added a solution of **4** (20 mg, 0.05 mmol, 1.0 eq.) in pentane (10 mL) at room temperature. The reaction mixture was stirred for 15 minutes. A slightly pink solid was collected by filtration and washed with pentane (2 x 10 mL) and Et_2O (10 mL). After eluting the crude product with THF (10 mL) the solvent was removed under reduced pressure to afford **12** as a colorless solid (21 mg, 0.02 mmol, 47%).

m.p. 280 °C (decomposition); **^{1}H NMR** (400 MHz, $CDCl_3$, 298K): 7.65-7.58 (m, 2H, Ar-H), 7.38 (d, J = 7.7 Hz, 4H, Ar-H), 2.84 (brs, J = 7.7 Hz, 2H, CH_2-BR_3), 2.20 (hept, J = 6.7 Hz, 2H, C*H*(CH_3)$_2$), 1.94 (hept, J = 6.6 Hz, 2H, C*H*(CH_3)$_2$), 1.75 (s, 3H, triazol-CH_3), 1.33 (d, J = 6.7 Hz, 6H, CH(CH_3)$_2$), 1.18 (d, J = 6.8 Hz, 6H, CH(CH_3)$_2$), 1.13 (d, J = 6.8 Hz, 6H, CH(CH_3)$_2$), 1.08 (d, J = 6.8 Hz, 6H, CH(CH_3)$_2$); **^{13}C {1H} NMR** ($CDCl_3$, 125 MHz, 298K): 150.3 (triazole-C), 149.6 (C_6F_5), 147.2 (C_6F_5), 145.7 (Ar-C), 145.3 (Ar-C), 140.0 (C_6F_5), 138.1 (triazole-C), 137.5 (C_6F_5), 135.6 (C_6F_5), 133.1 (Ar-CH), 132.9 (Ar-CH), 129.2 (Ar-C), 128.9 (Ar-C), 125.3 (Ar-CH), 125.0 (Ar-CH), 29.6 (*C*H(*C*H_3)$_2$), 29.2 (*C*H(*C*H_3)$_2$), 26.7 (CH(CH_3)$_2$), 25.1 (CH(CH_3)$_2$), 22.9 (CH(CH_3)$_2$), 22.1 (CH(CH_3)$_2$),

20.0 (q, J_{CB} = 39.5 Hz, CH_2-BR_3), 10.1 (triazol-CH_3); **^{19}F NMR** ($CDCl_3$, 377 MHz, 298K): -132.29 (d, *J* = 23.3 Hz, 6F, *o*-C_6F_5), -160.78 (d, *J* = 20.6 Hz, 3F, *p*-C_6F_5), -165.07 (t, *J* = 19.4 Hz, 6F, *m*-C_6F_5); **^{11}B NMR** ($CDCl_3$, 96 MHz, 298K): -14.86; **IR (ATR)** [cm^{-1}]: ṽ = 2974, 2934, 2876, 1643, 1513, 1454, 1389, 1368, 1339, 1276, 1233, 1080, 1061, 1044, 1015, 980, 952, 939, 809, 772, 754, 738, 692, 673, 648, 638, 605, 571, 451, 440; **HR-MS-ESI(+)** calc. $C_{46}H_{39}BF_{15}N_3Na^+$ $[M+Na]^+$ 952.2898, found 952.2885; **EA** calc. for [$C_{46}H_{39}BF_{15}N_3$] C: 59.43; H: 4.23; N: 4.52; found: C: 59.50; H: 4.13; N: 4.46.

Synthesis of **13**

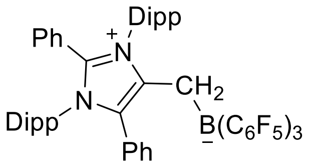

To a stirred solution of $B(C_6F_5)_3$ (23 mg, 0.05 mmol, 1.0 eq.) in pentane (5 mL) was slowly added a solution of **6** (25 mg, 0.05 mmol, 1.0 eq.) in pentane (10 mL) at room temperature. The reaction mixture was stirred for 15 min. A slightly pink solid was collected by filtration and washed with pentane (2 x 10 mL) and Et_2O (10 mL). After eluting the crude product with THF (10 mL) the solvent was removed under educed pressure to afford **13** as a colorless solid (20 mg, 0.02 mmol, 42%).

m.p. 200 °C (decomposition); **^{1}H NMR** (400 MHz, $CDCl_3$, 298K): 7.60 (t, *J* = 7.8 Hz, 1H, Ar-H), 7.39 (t, *J* = 7.8 Hz, 1H, Ar-H), 7.31 (d, *J* = 7.9 Hz, 2H, Ar-H), 7.20 (dt, *J* = 11.3, 7.5 Hz, 2H, Ar-H), 7.11 (d, *J* = 7.8 Hz, 2H, Ar-H), 7.10 – 6.96 (m, 4H, Ar-H), 6.86 (d, *J* = 1.4 Hz, 2H, Ar-H), 6.76 (d, *J* = 7.0 Hz, 2H, Ar-H), 3.07 (bs, 2H, CH_2-BR_3), 2.63 (hept, *J* = 6.3, 5.8 Hz, 2H, C*H*(CH_3)$_2$), 2.49 (hept, *J* = 6.0 Hz, 2H, C*H*(CH_3)$_2$), 1.15 (d, *J* = 6.6 Hz, 6H, CH(CH_3)$_2$), 0.97 (d, *J* = 6.7 Hz, 6H, CH(CH_3)$_2$), 0.81 (d, *J* = 6.7 Hz, 6H, CH(CH_3)$_2$), 0.78 (d, *J* = 6.7 Hz, 6H, CH(CH_3)$_2$); **^{13}C {^{1}H} NMR** ($CDCl_3$, 101 MHz, 298K): 145.6 (imidazole-C), 145.5 (Ar-C), 143.4 (Ar-C), 142.2 (Ar-C), 132.1 (Ar-CH), 131.9 (Ar-CH), 131.7 (Ar-C), 131.6 (Ar-CH), 130.4 (Ar-C), 130.4 (Ar-C), 130.0 (Ar-CH), 129.8 (Ar-CH), 129.6, 128.4 (Ar-CH), 128.3 (Ar-CH), 126.2 (Ar-C), 126.0 (Ar-CH), 125.5 (Ar-CH), 122.5 (imidazole-C), 29.2 (*C*H(CH_3)$_2$), 29.1 (*C*H(CH_3)$_2$), 24.2 (CH(*C*H_3)$_2$), 24.0 (CH(*C*H_3)$_2$), 23.8 (CH(*C*H_3)$_2$), 23.8 (CH(*C*H_3)$_2$), 19.4 (HSQC, *C*H_2-BR_3), boron and fluorine coupled signals could not be detected due to the low solubility of **13**; **^{19}F NMR** ($CDCl_3$, 377 MHz, 298K) -131.64 (d, *J* = 23.5 Hz, 6F, *o*-C_6F_5), -162.58 (t, *J* = 20.7 Hz, 3F, *p*-C_6F_5), -166.09 (t, *J* = 19.6 Hz, 6F, *m*-C_6F_5); **^{11}B NMR** ($CDCl_3$, 128 MHz, 298K): -14.0; **IR (ATR)** [cm^{-1}]: ṽ = 2969, 2934, 2874, 2357, 1640, 1512, 1453, 1389, 1368, 1325, 1272, 1079, 1059, 1033, 972, 944, 922, 830, 798, 768, 756, 721, 697, 671, 625, 530, 510, 461, 445; **HR-MS-ESI(+)** calc. $C_{58}H_{46}BF_{15}N_2Na^+$ $[M+Na]^+$ 1089.3416, found 1089.3409; **EA** calc. for [$C_{58}H_{46}BF_{15}N_2$] C: 65.30; H: 4.35; N: 2.63; found: C: 65.07; H: 4.14; N: 2.53.

4. NMR spectra

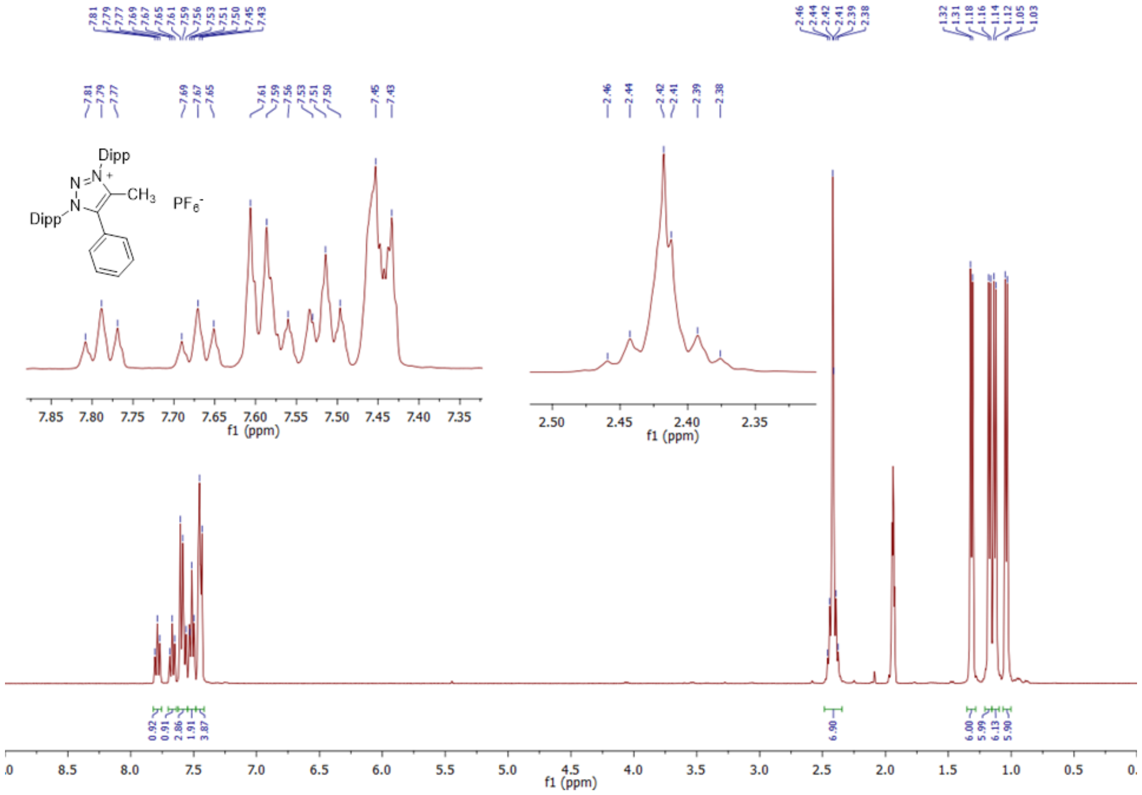

^{1}H NMR (400 MHz, CD_3CN, 295K) of **1**

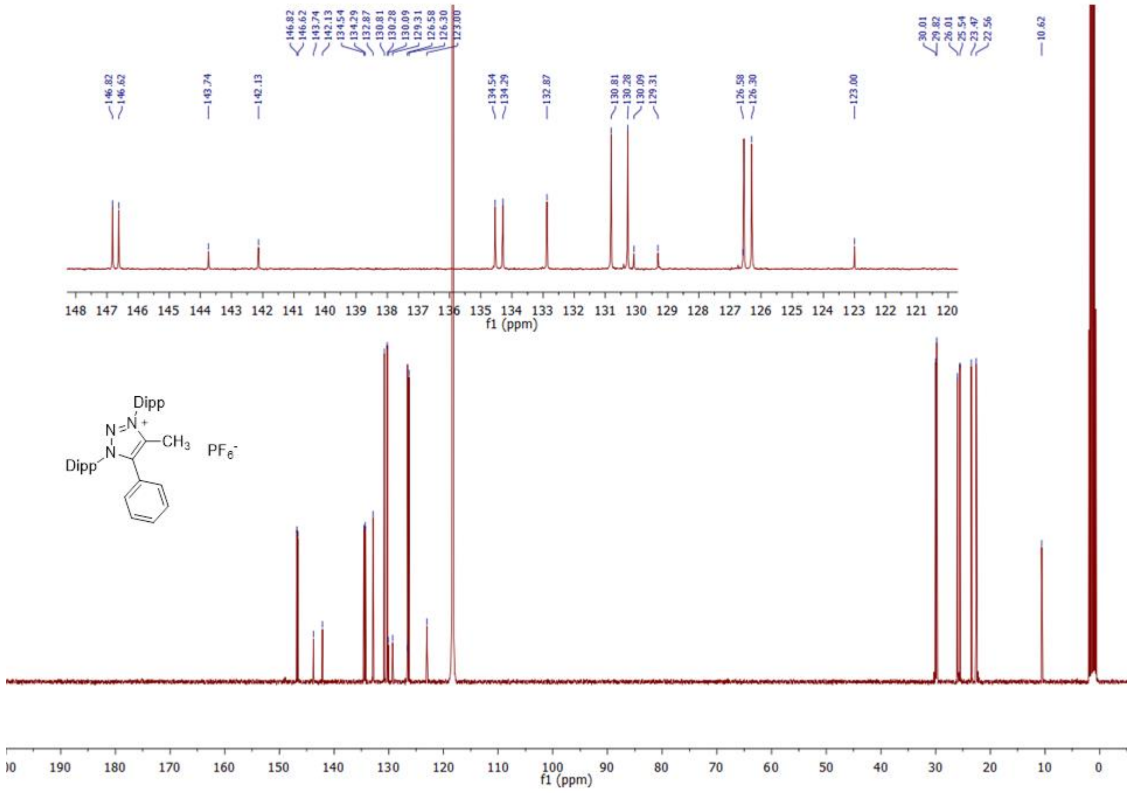

^{13}C {^{1}H} NMR (100 MHz, CD_3CN, 295K) of **1**

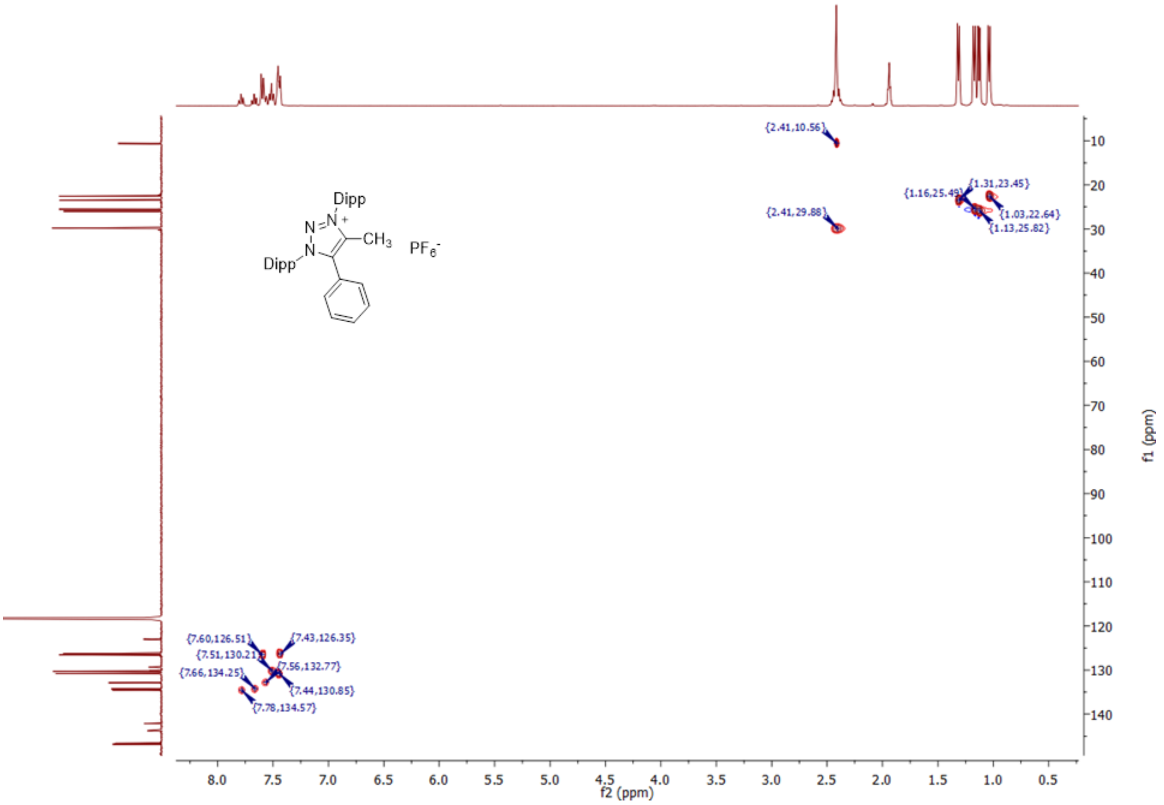

$^{1}H/^{13}C$ HSQC (400/100 MHz, CD_3CN, 298K) of **1**

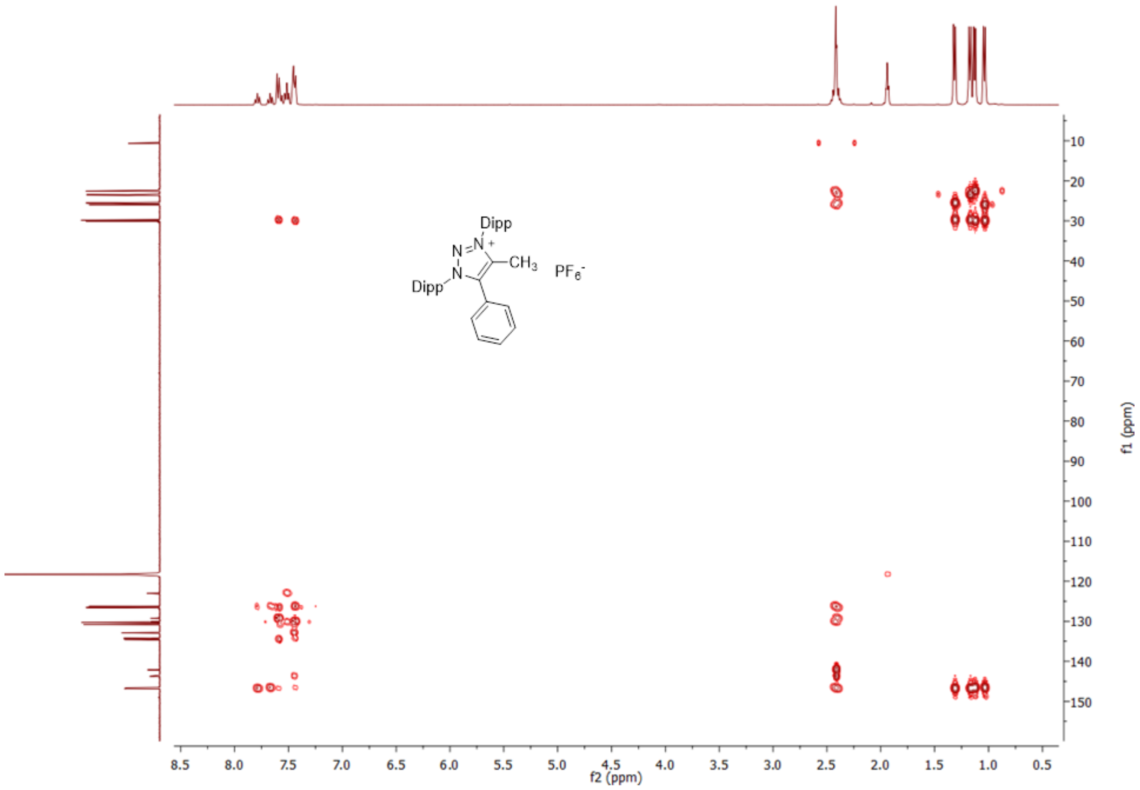

$^{1}H/^{13}C$ HMBC (400/100 MHz, CD_3CN, 298K) of **1**

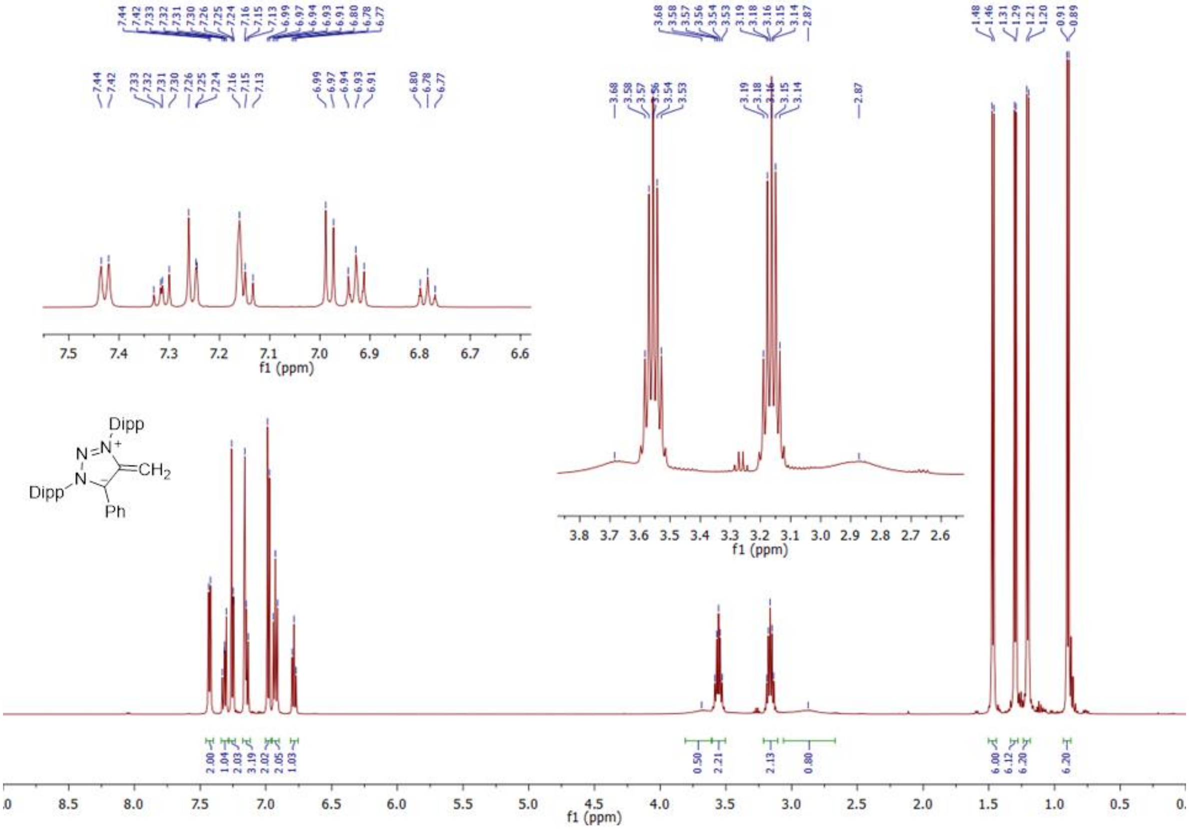

^{1}H NMR (500 MHz, C_6D_6, 295K) of **2**

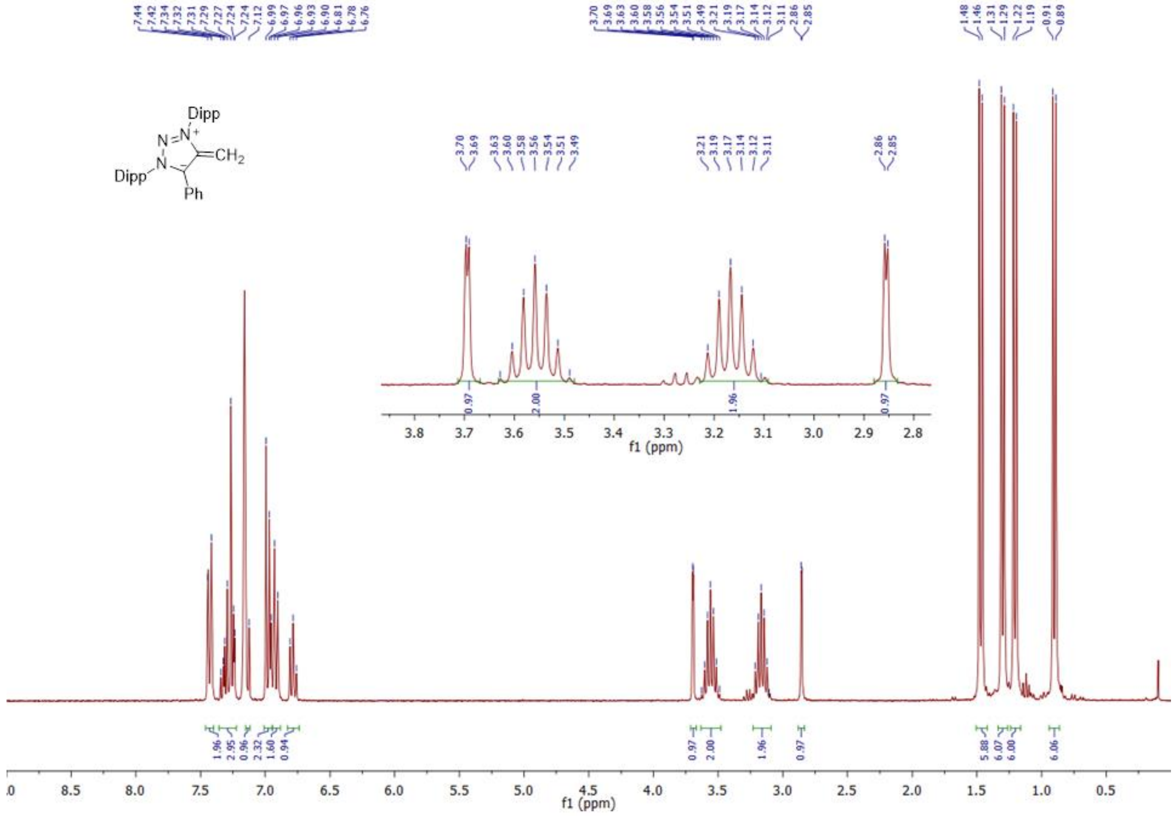

^{1}H NMR (500 MHz, C_6D_6, 295K) of **2** (slight KHMDS signal)

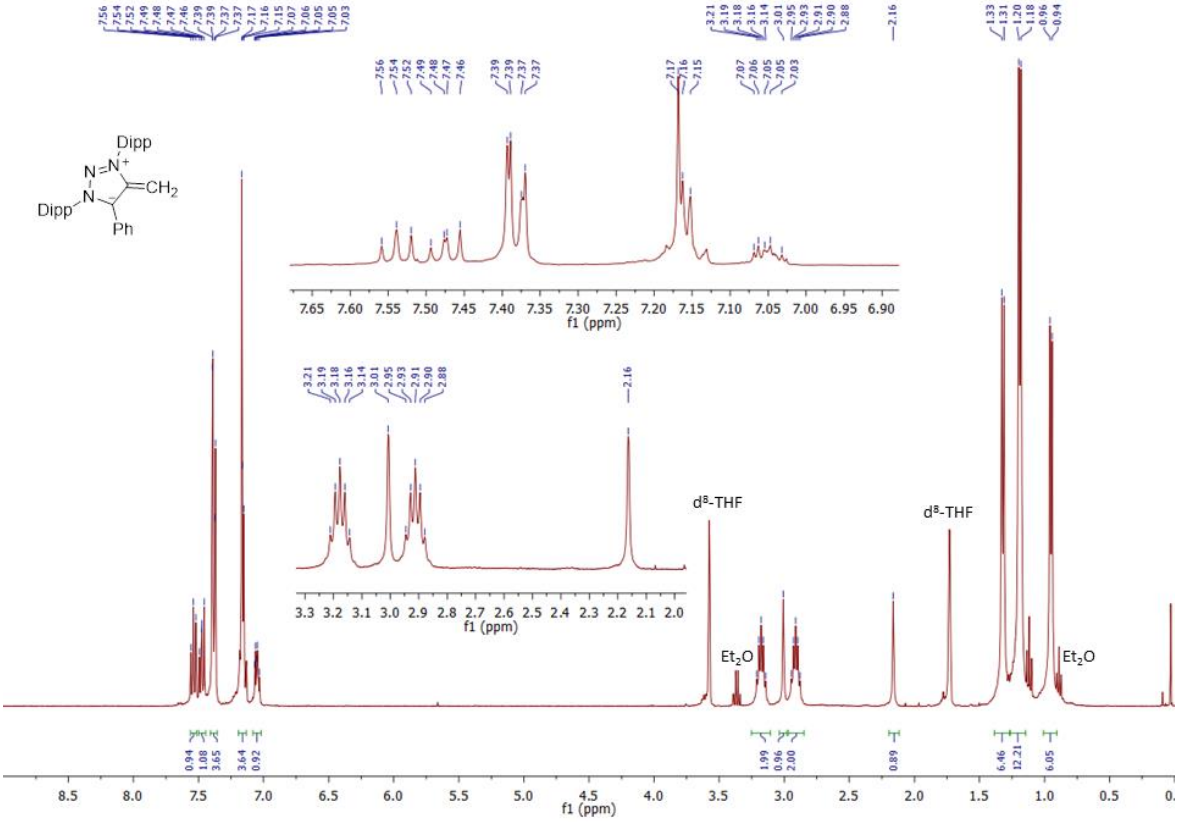

^{1}H NMR (400 MHz, d^8-THF, 223K) of **2**

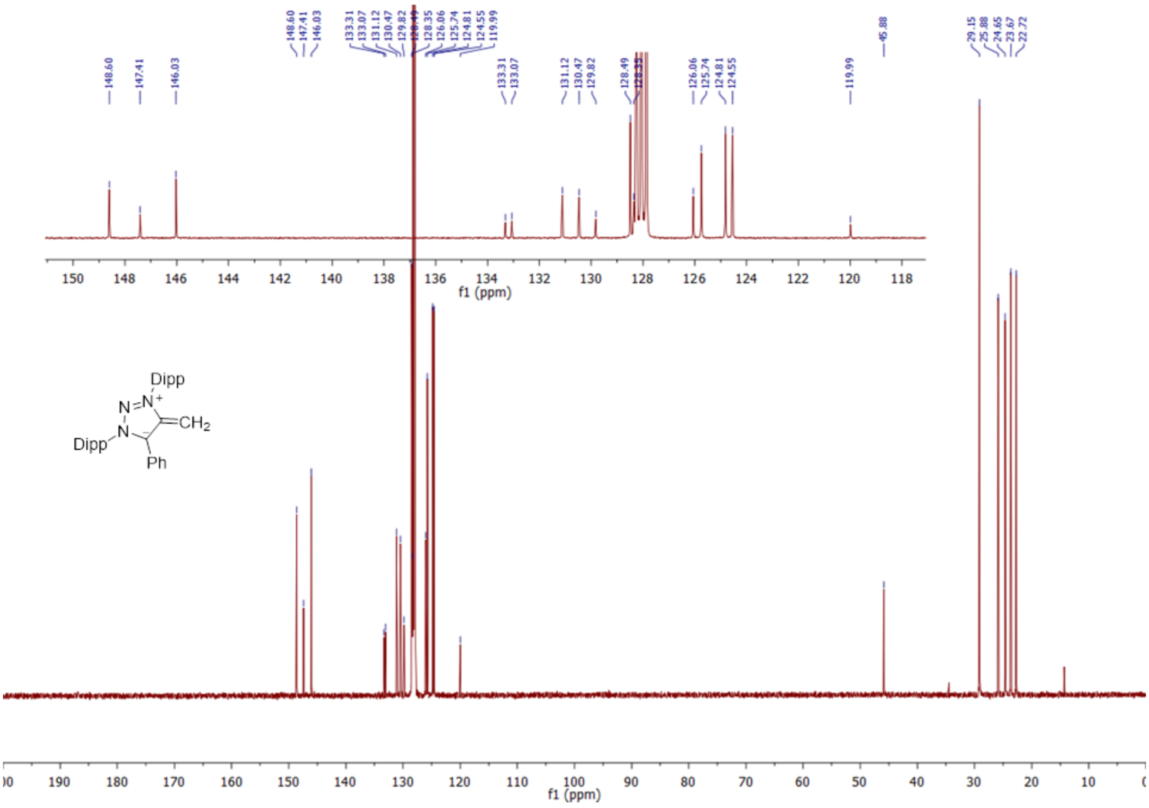

^{13}C {^{1}H} NMR (125 MHz, C_6D_6, 295K) of **2**

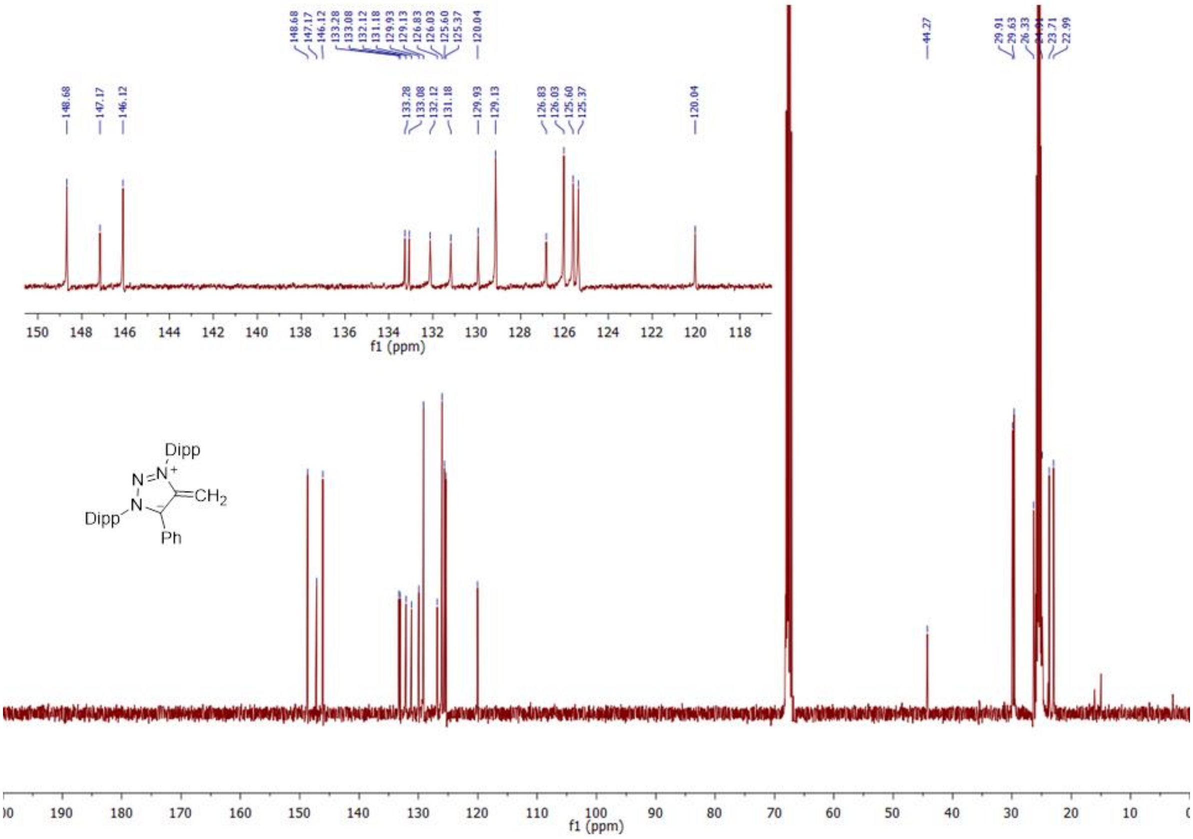

^{13}C {^1H} **NMR** (100 MHz, d^8-THF, 223K) of **2**

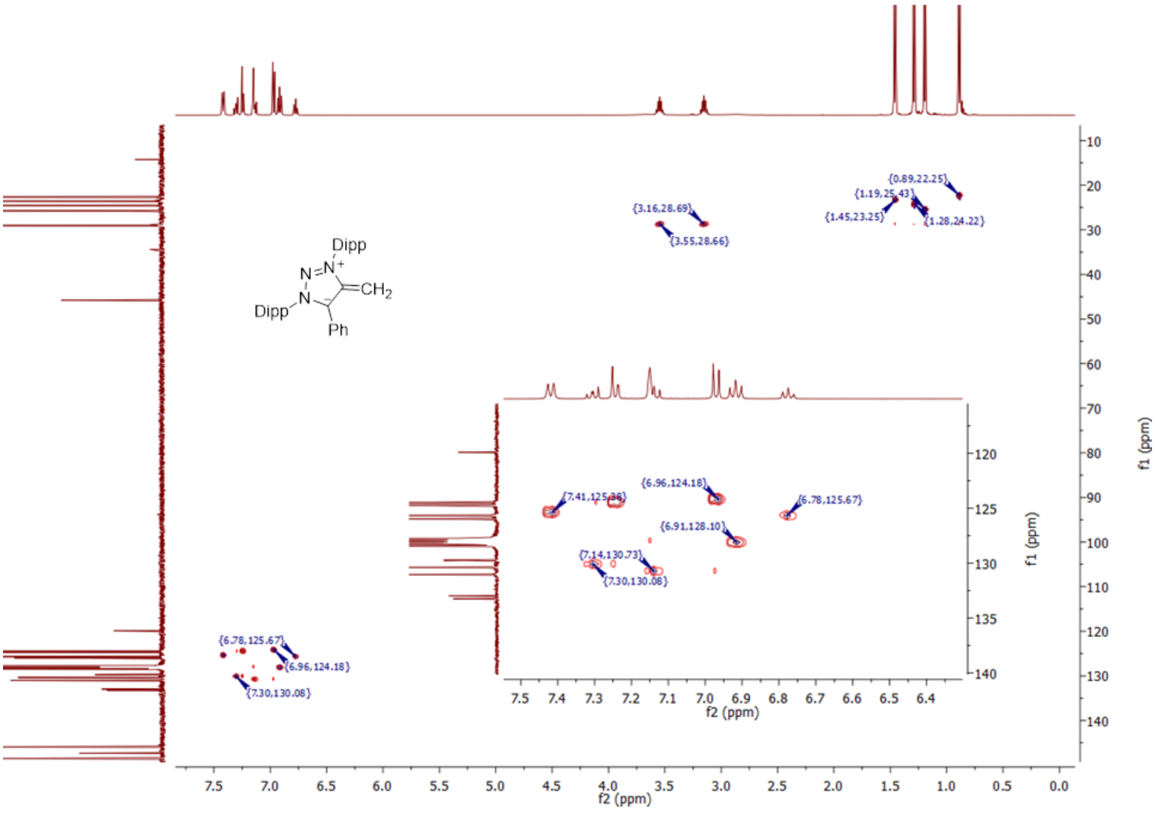

$^1H/^{13}C$ **HSQC** (500/125 MHz, C_6D_6, 295K) of **2**

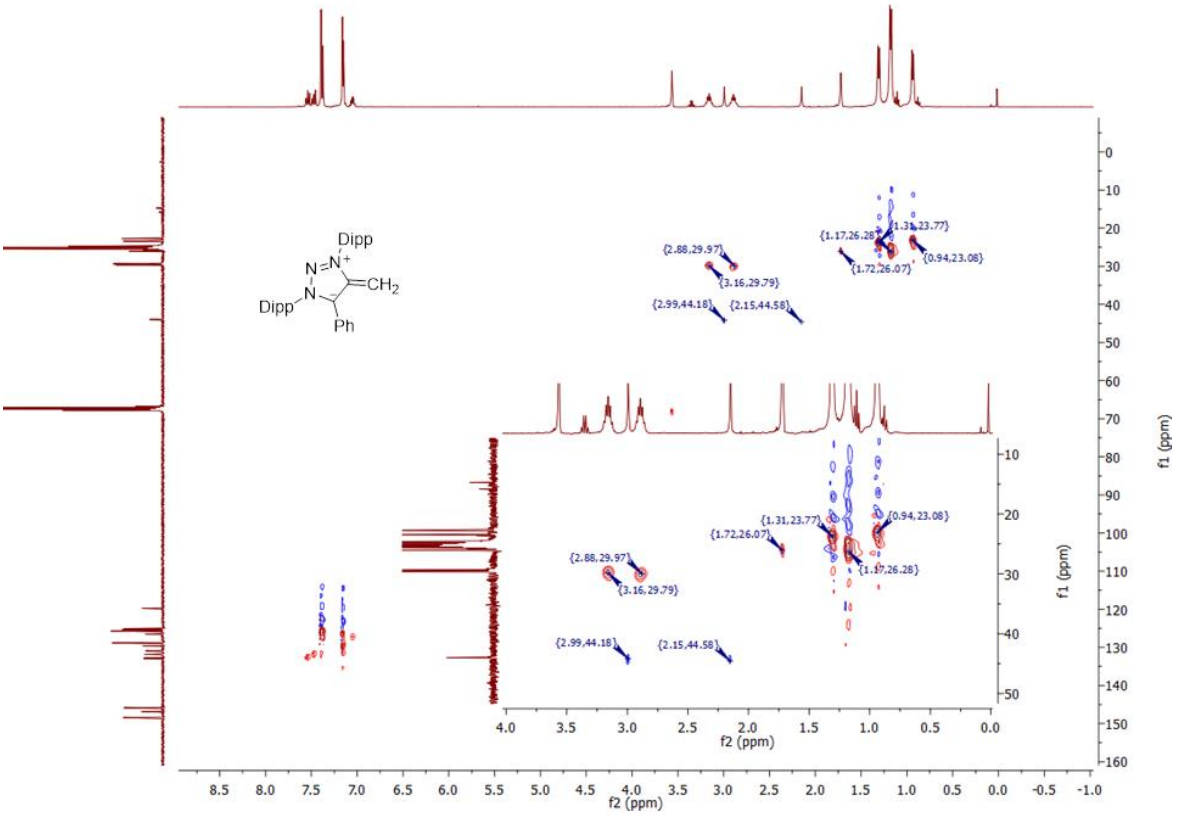

^{1}H/^{13}C HSQC (400/100 MHz, d^8-THF, 223K) of **2**

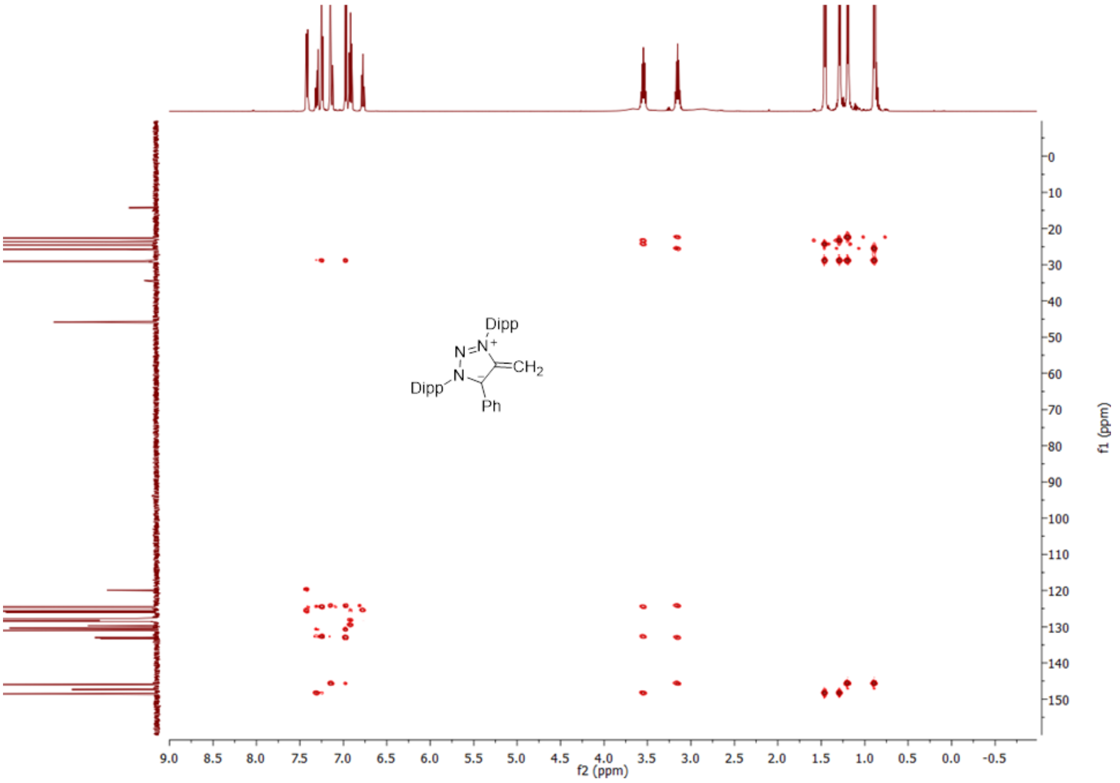

^{1}H/^{13}C HMBC (400/100 MHz, d^8-THF, 223K) of **2**

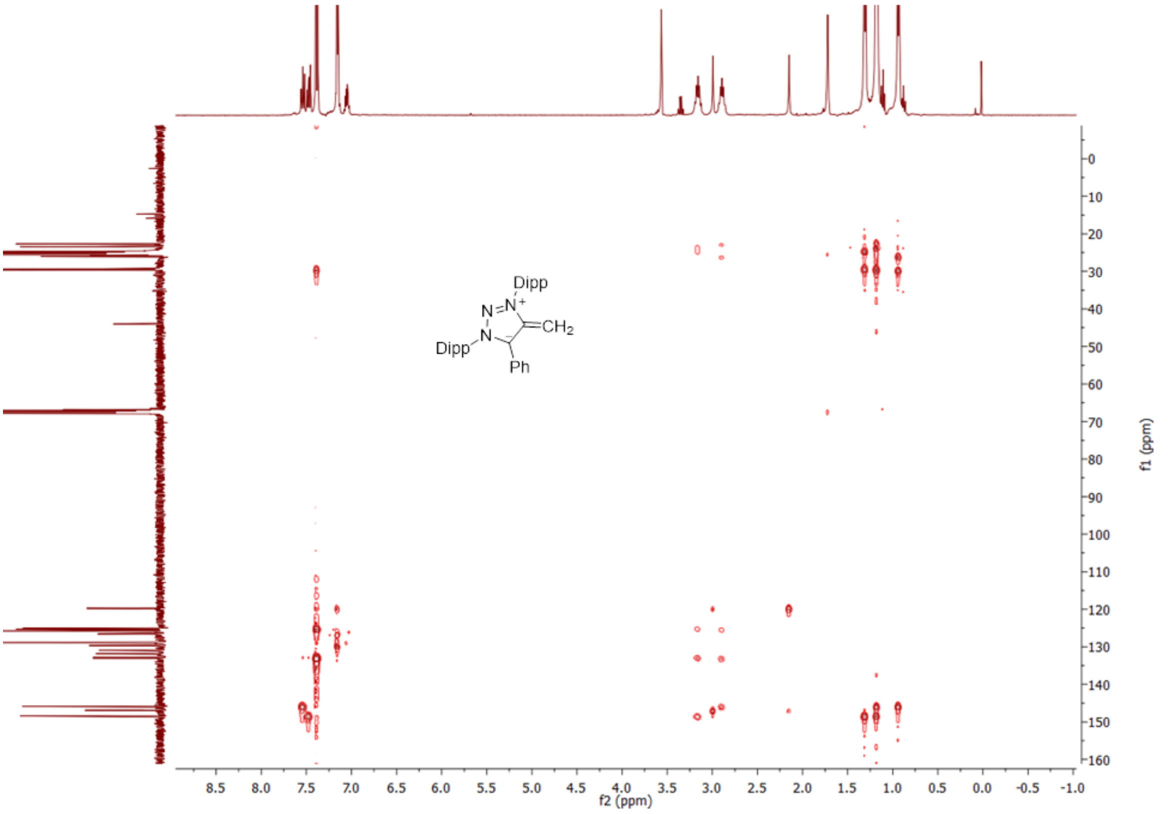

$^{1}H/^{13}C$ HMBC (400/100 MHz, d^{8}-THF, 223K) of **2**

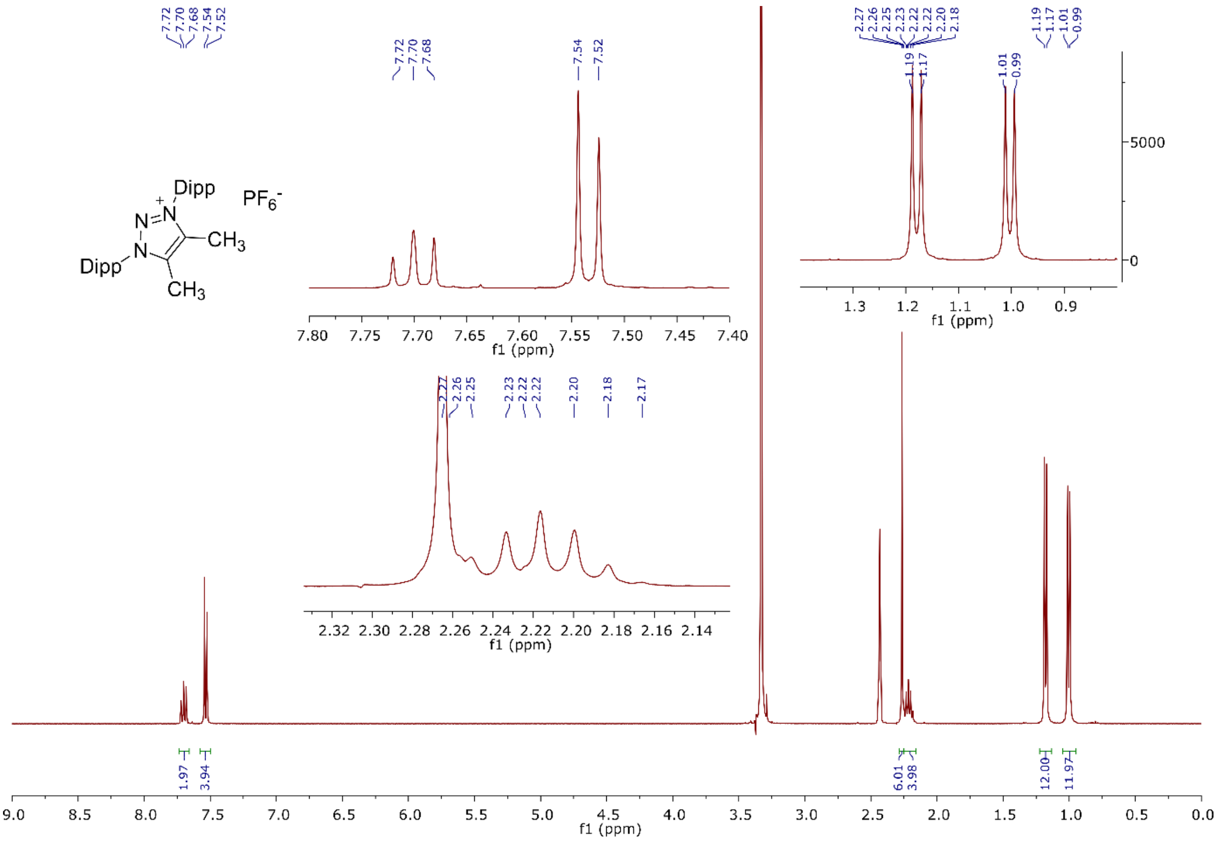

^{1}H NMR (400 MHz, DMSO-d_6, 298K) of **3**

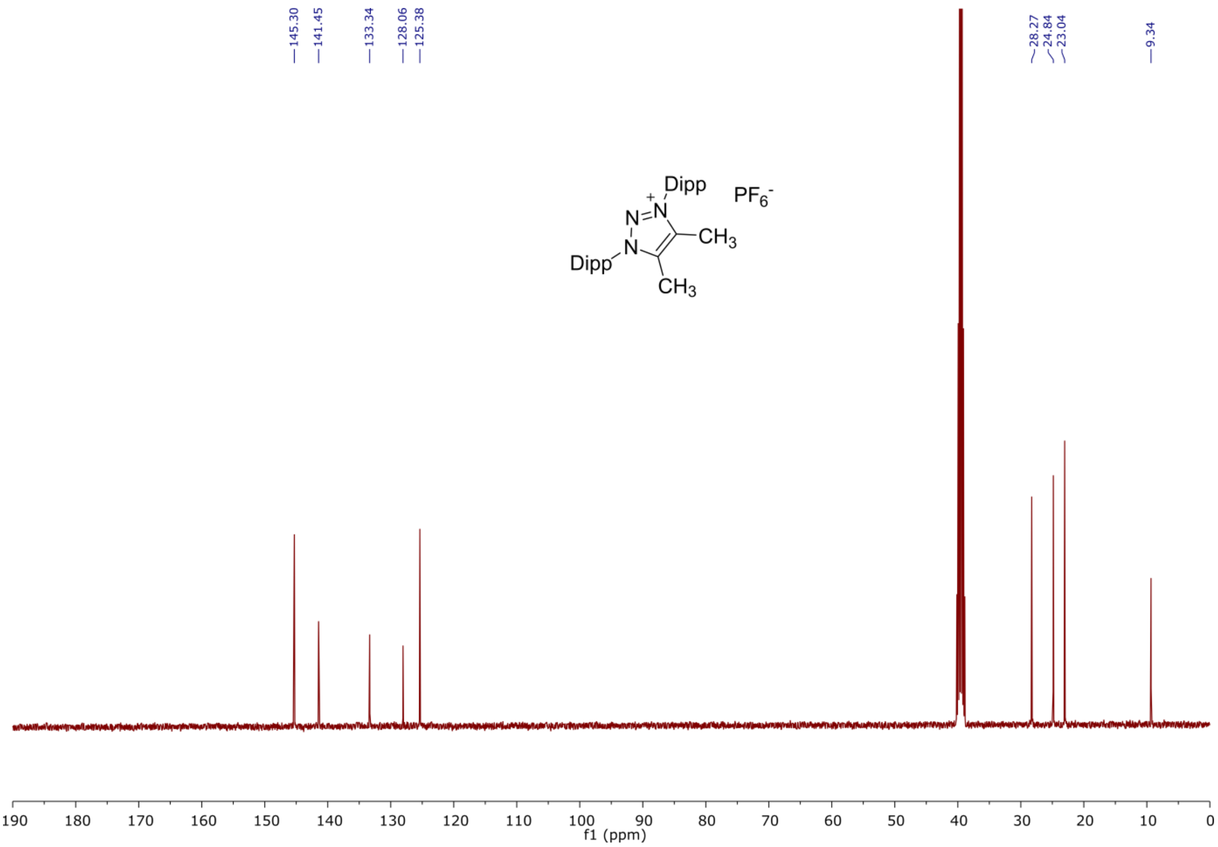

^{13}C {^{1}H} NMR (100 MHz, DMSO-d_6, 298K) of **3**

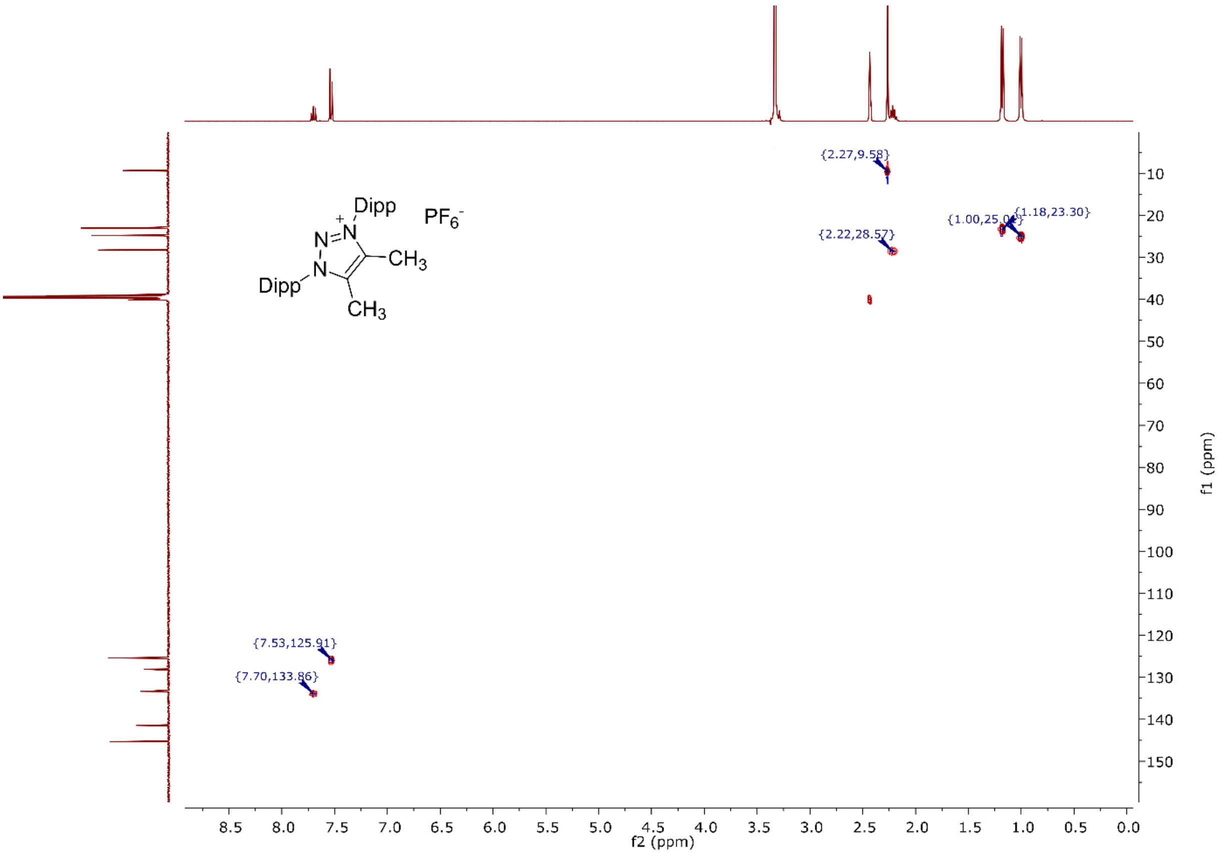

^{1}H/^{13}C HSQC (400/100 MHz, DMSO-d_6, 298K) of **3**

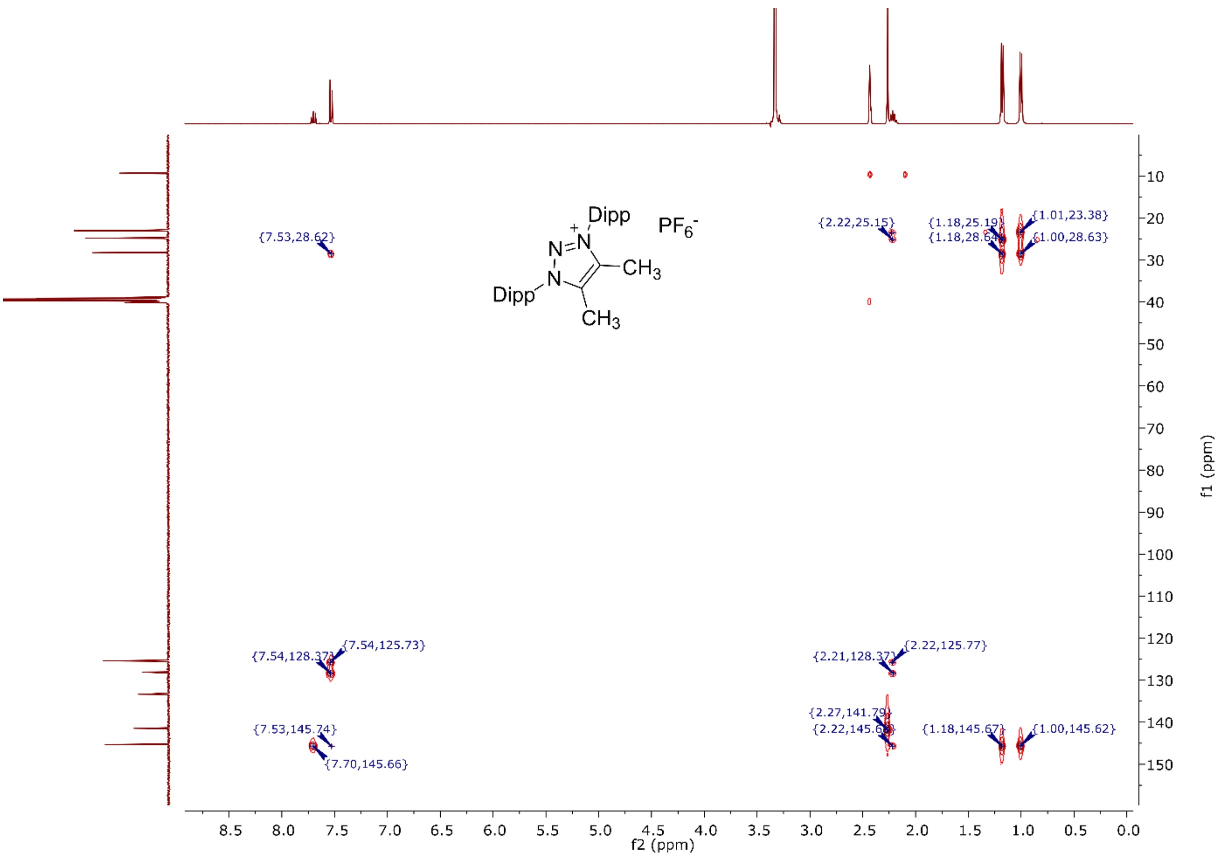

$^{1}H/^{13}C$ HMBC (400/100 MHz, DMSO-d_6, 298K) of **3**

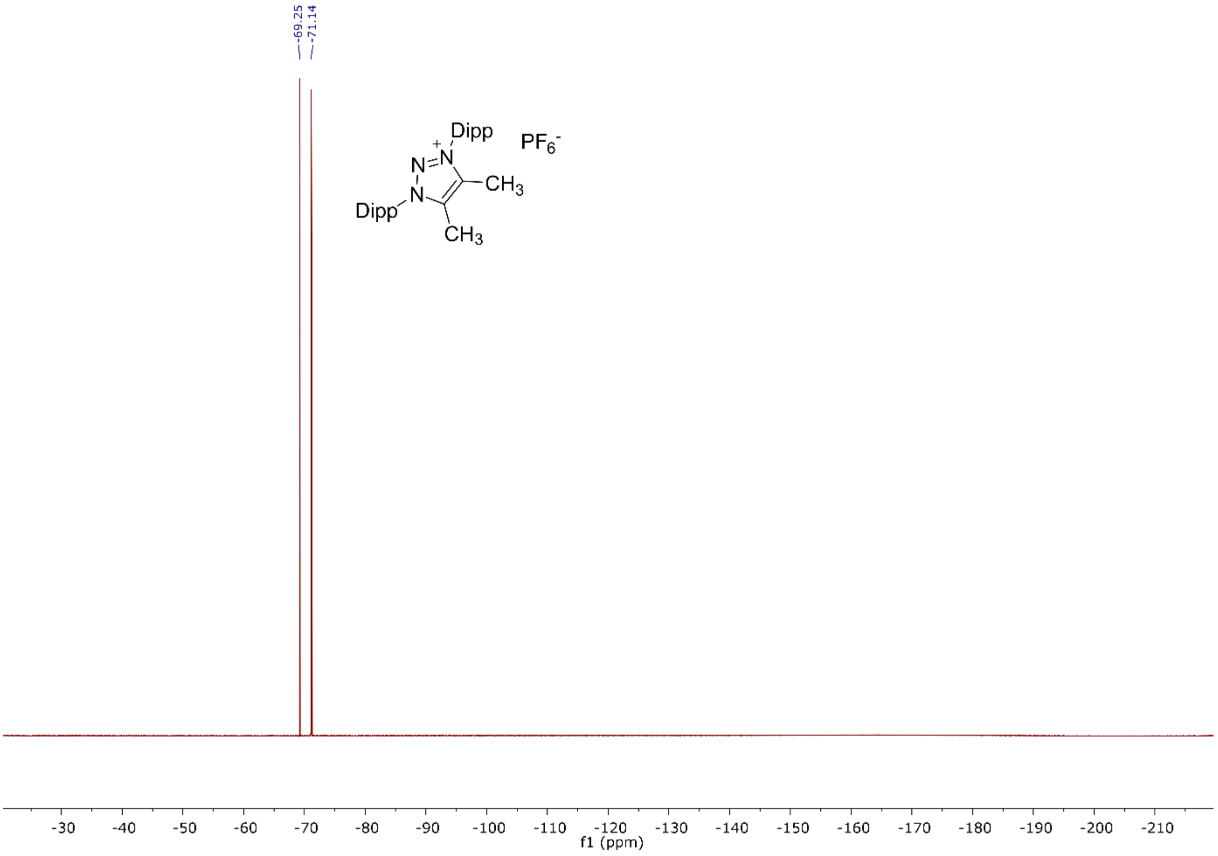

^{19}F NMR (376 MHz, DMSO-d_6, 298K) of **3**

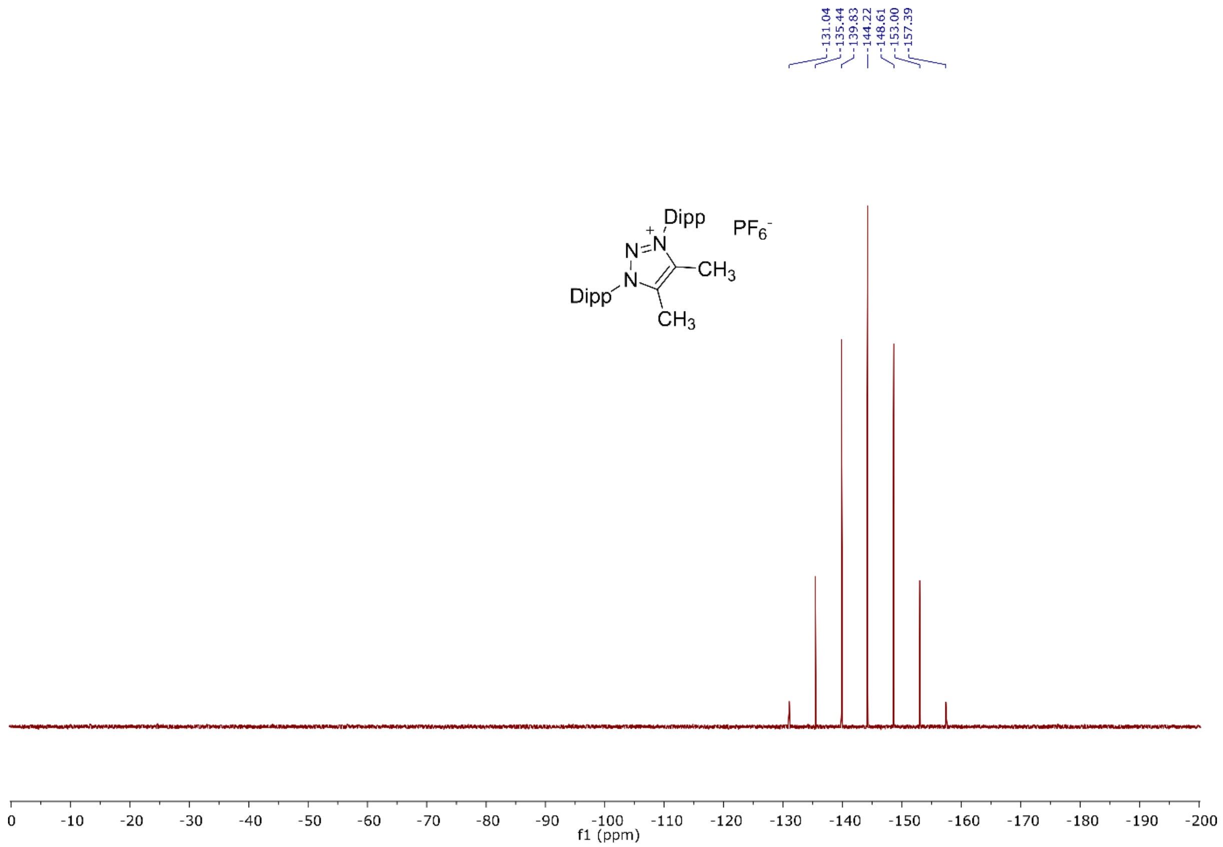

^{31}P NMR (162 MHz, DMSO-d_6, 298K) of **3**

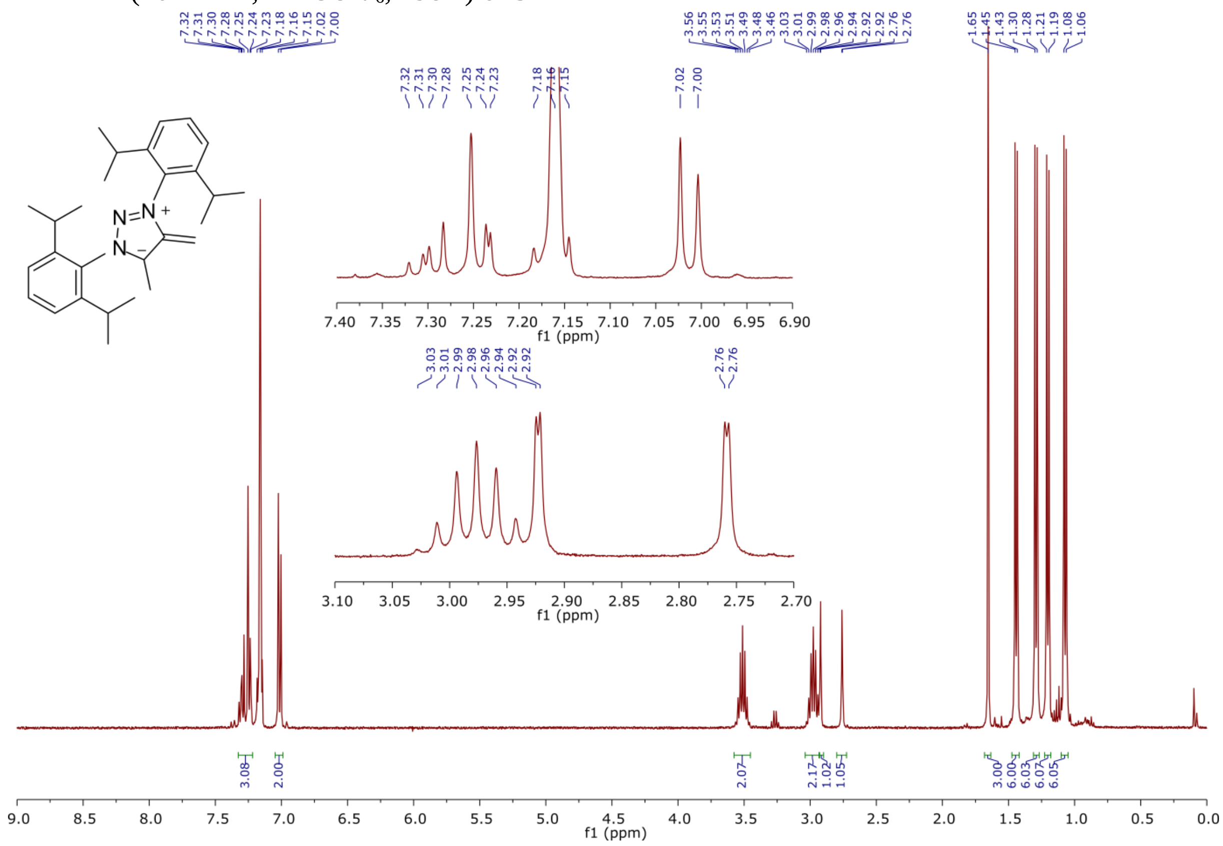

^{1}H NMR (400 MHz, C_6D_6, 298K) of **4**

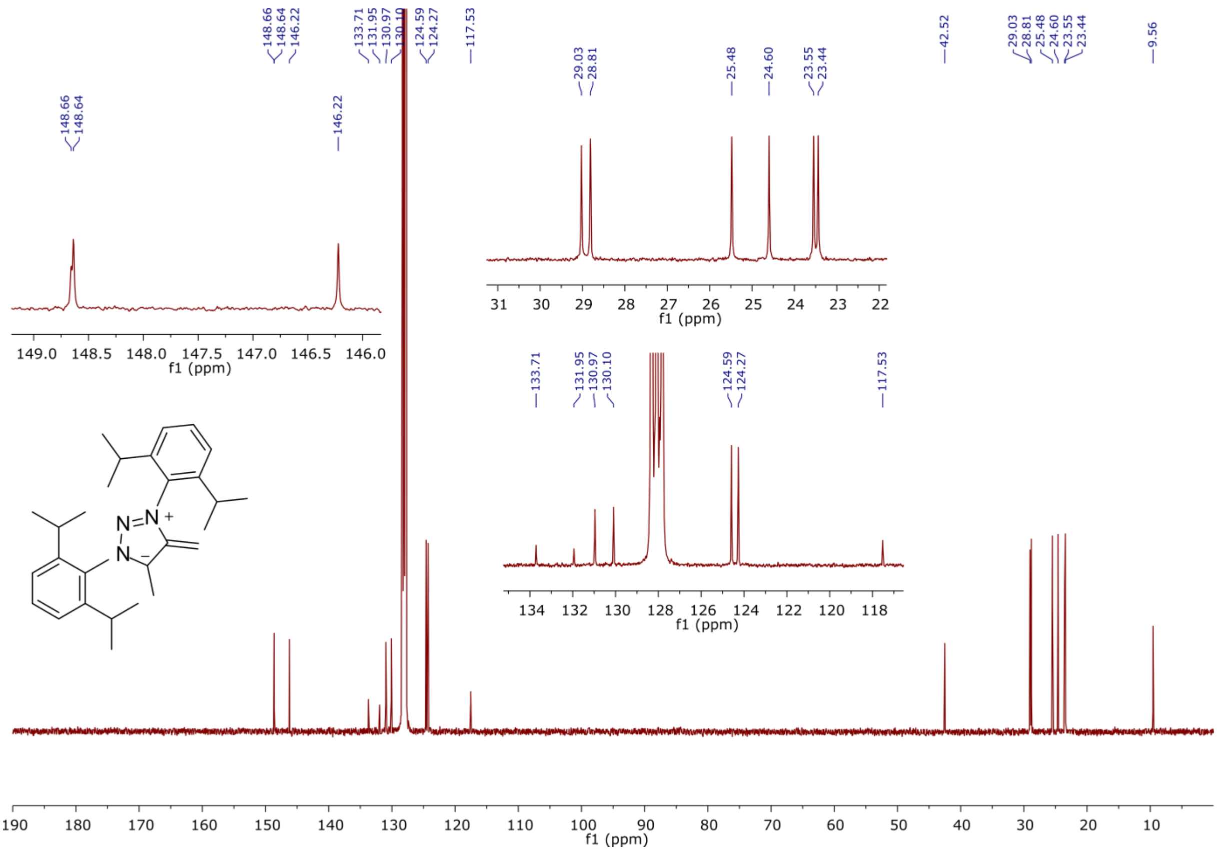

^{13}C {^{1}H} NMR (100 MHz, C_6D_6, 298K) of **4**

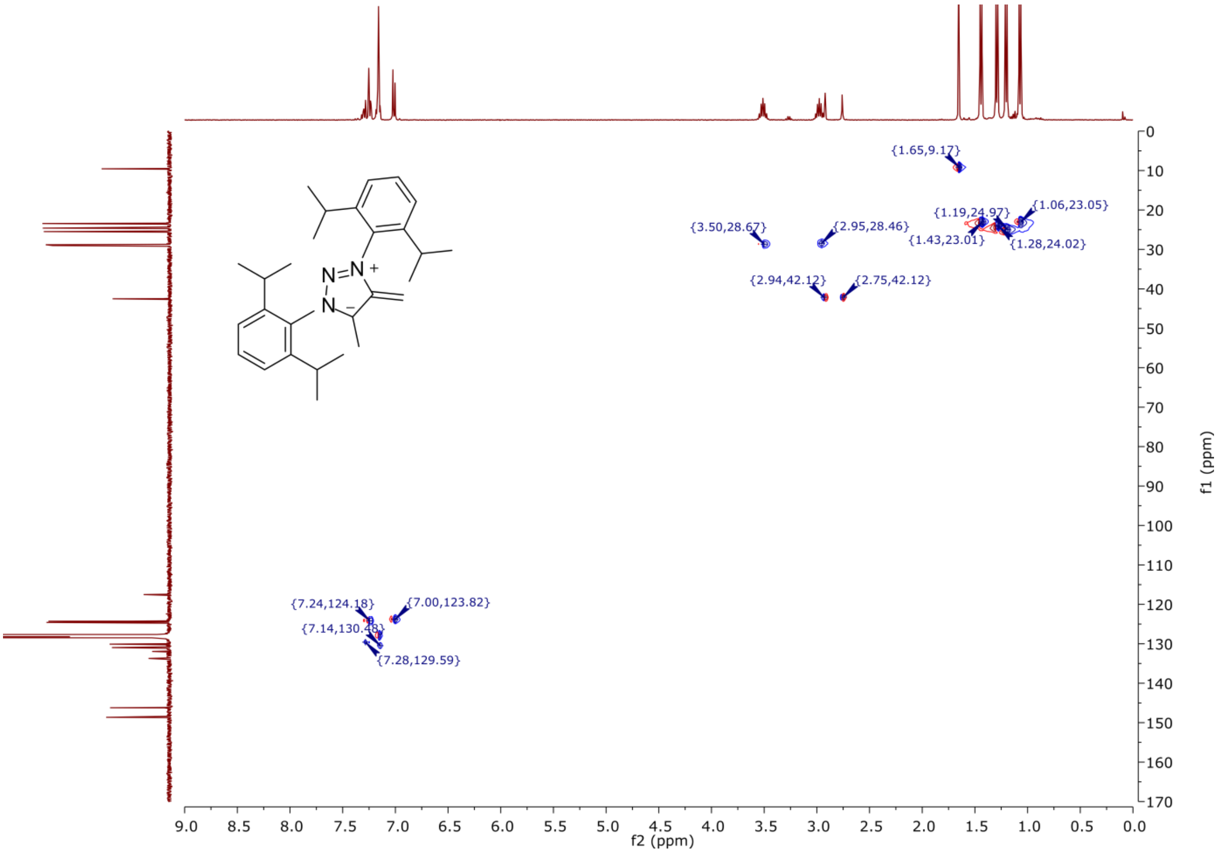

^{1}H/^{13}C HSQC (400/100 MHz, C_6D_6, 298K) of **4**

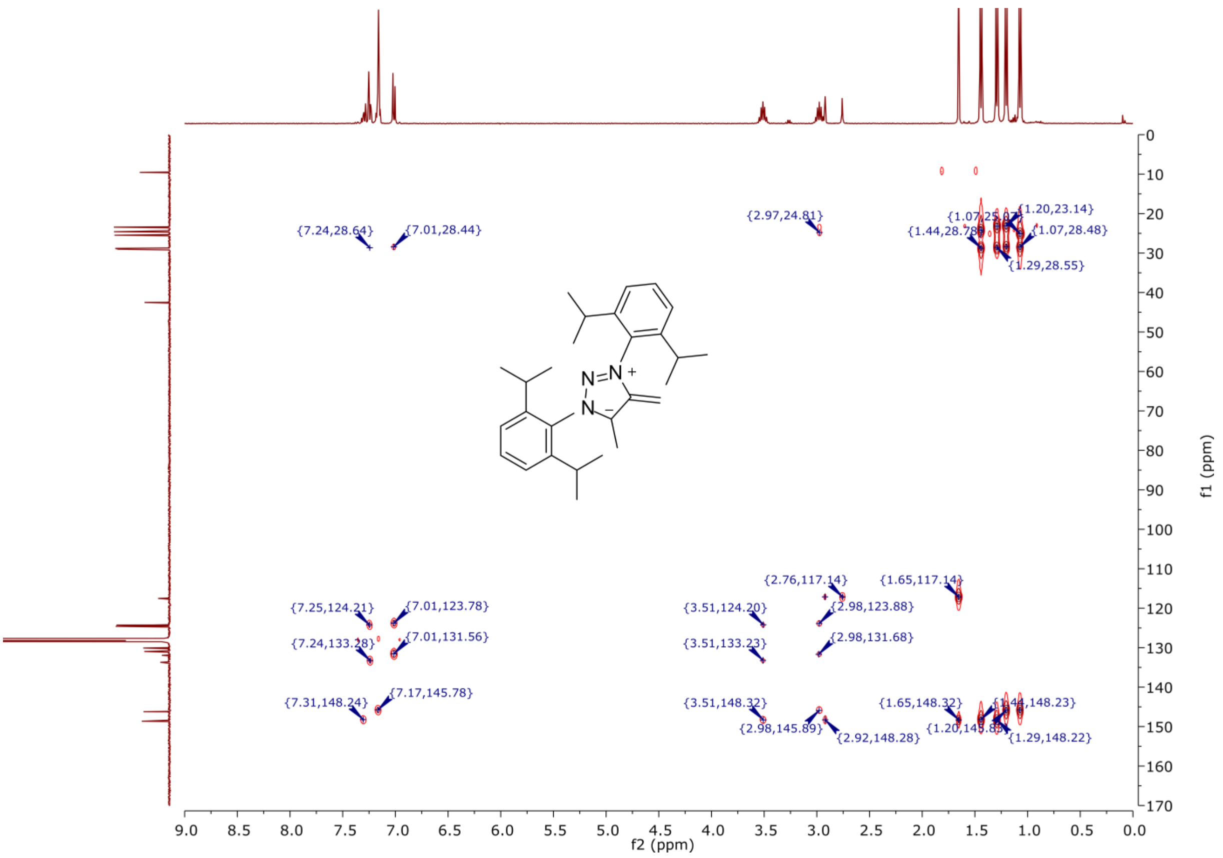

$^{1}H/^{13}C$ HMBC (400/100 MHz, C_6D_6, 298K) of **4**

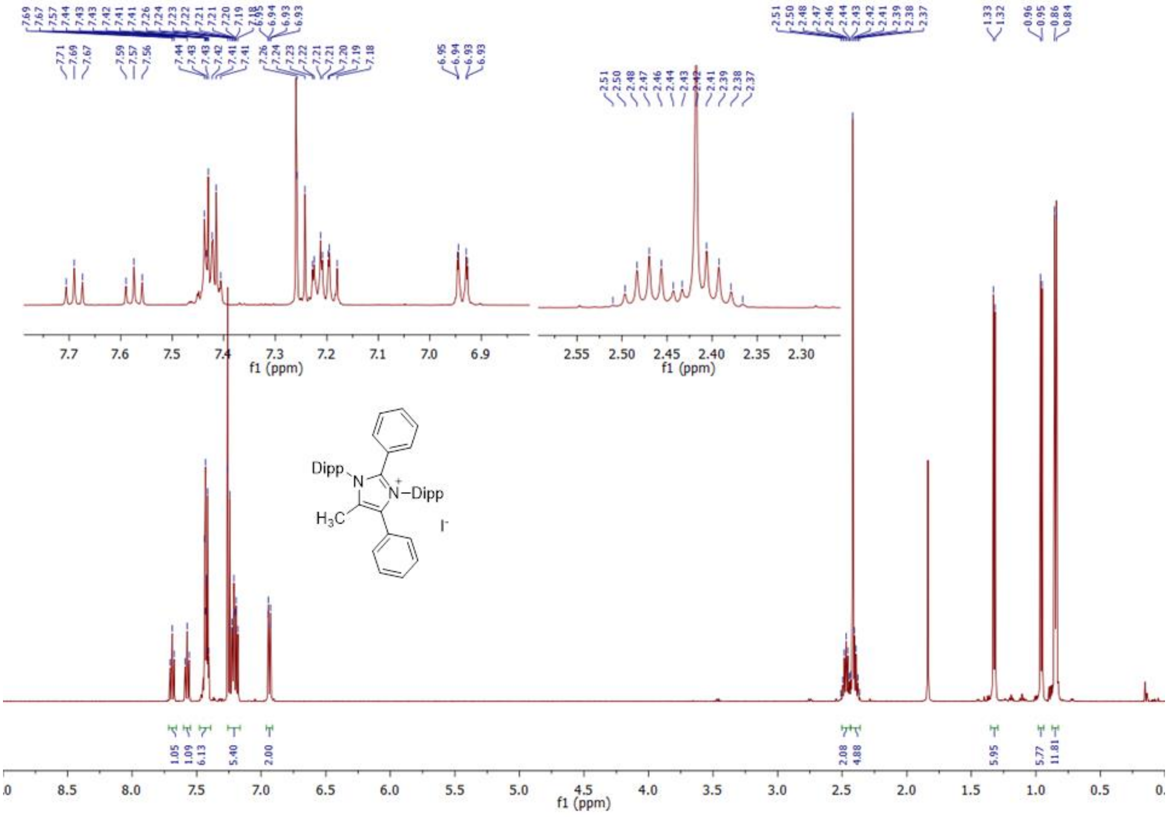

^{1}H NMR (500 MHz, $CDCl_3$, 295K) of **5**

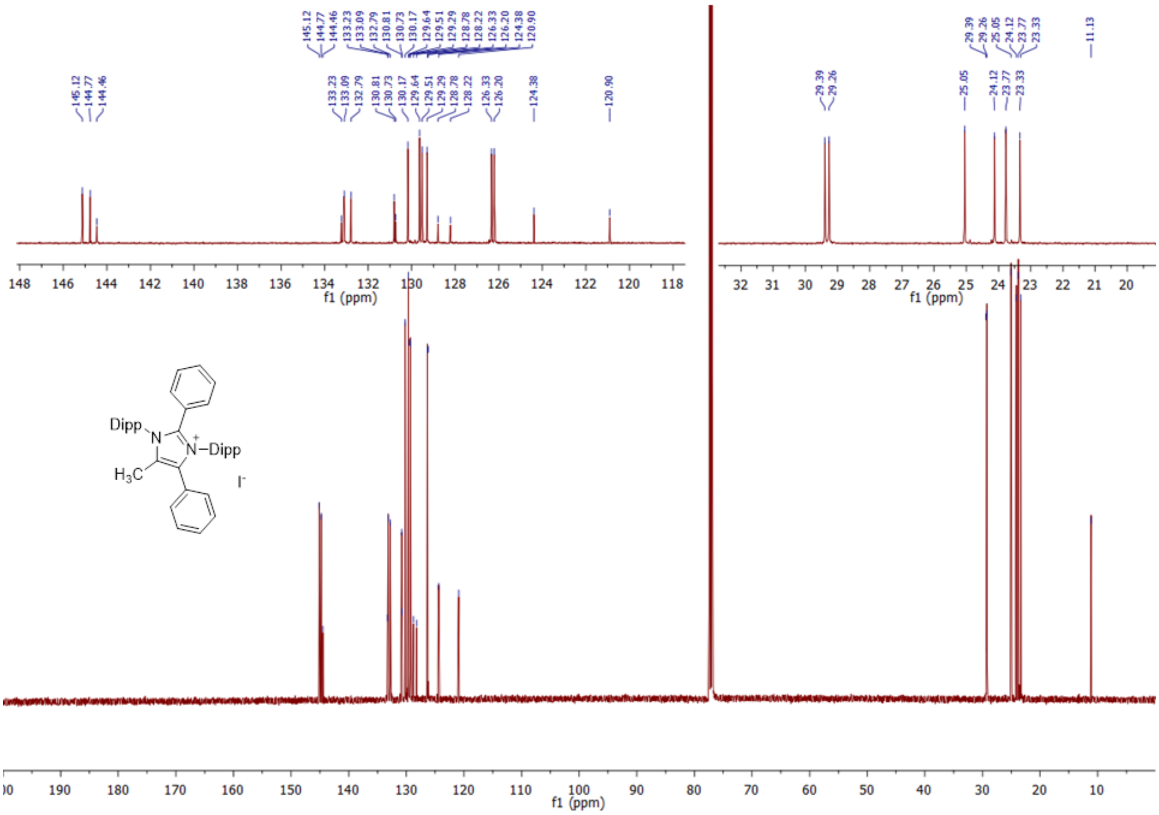

^{13}**C {^{1}H} NMR** (125 MHz, $CDCl_3$, 298K) of **5**

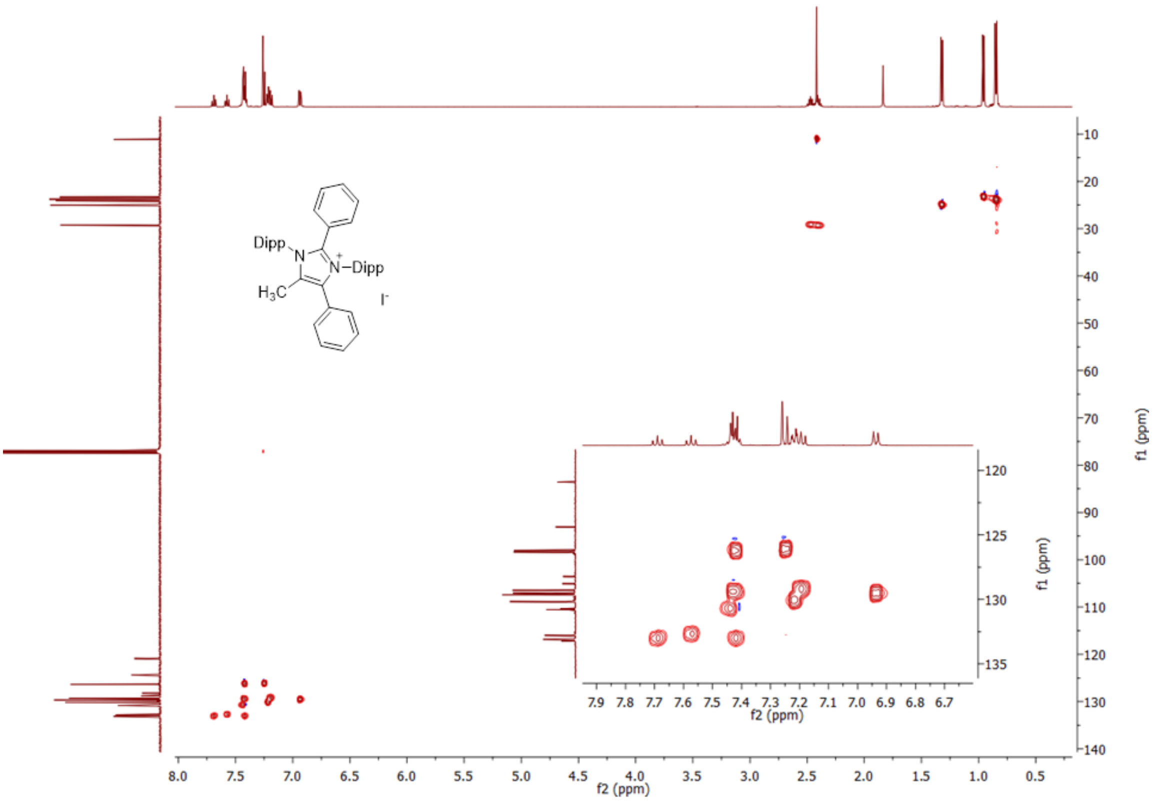

^{1}**H/**^{13}**C HSQC** (500/125 MHz, $CDCl_3$, 298K) of **5**

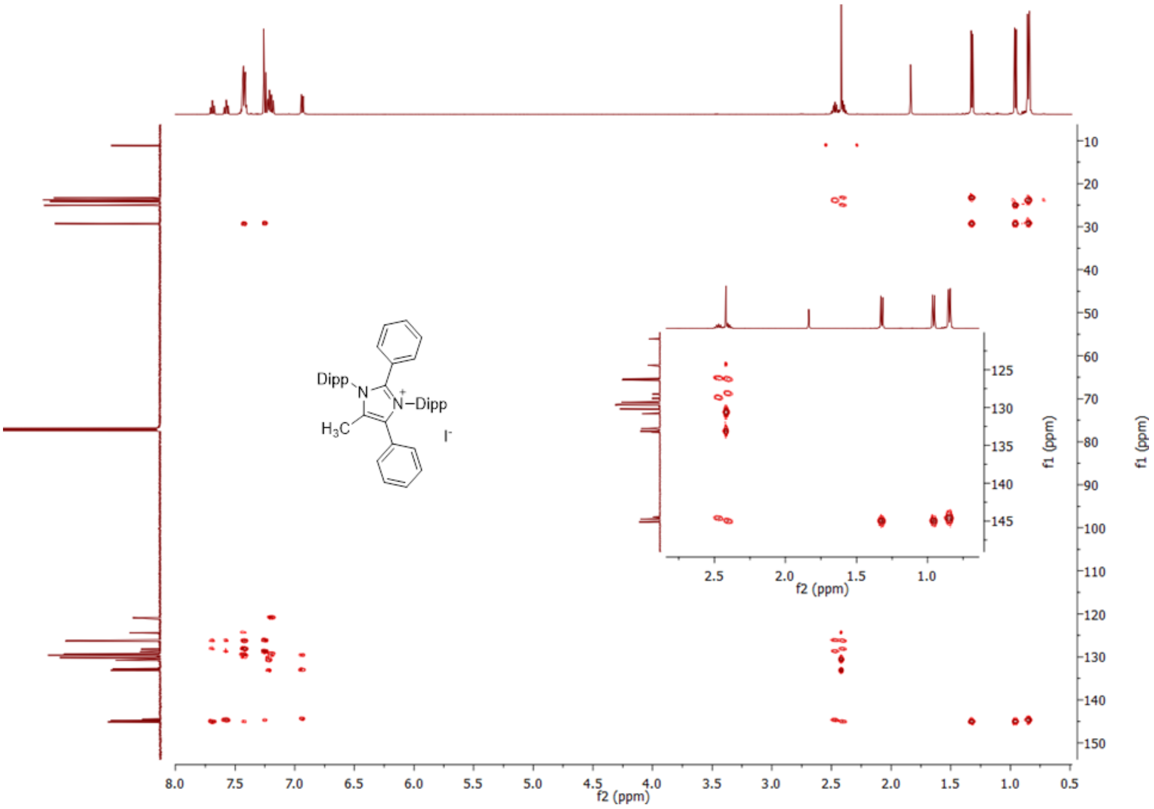

^{1}**H/^{13}C HMBC** (500/125 MHz, $CDCl_3$, 298K) of **5**

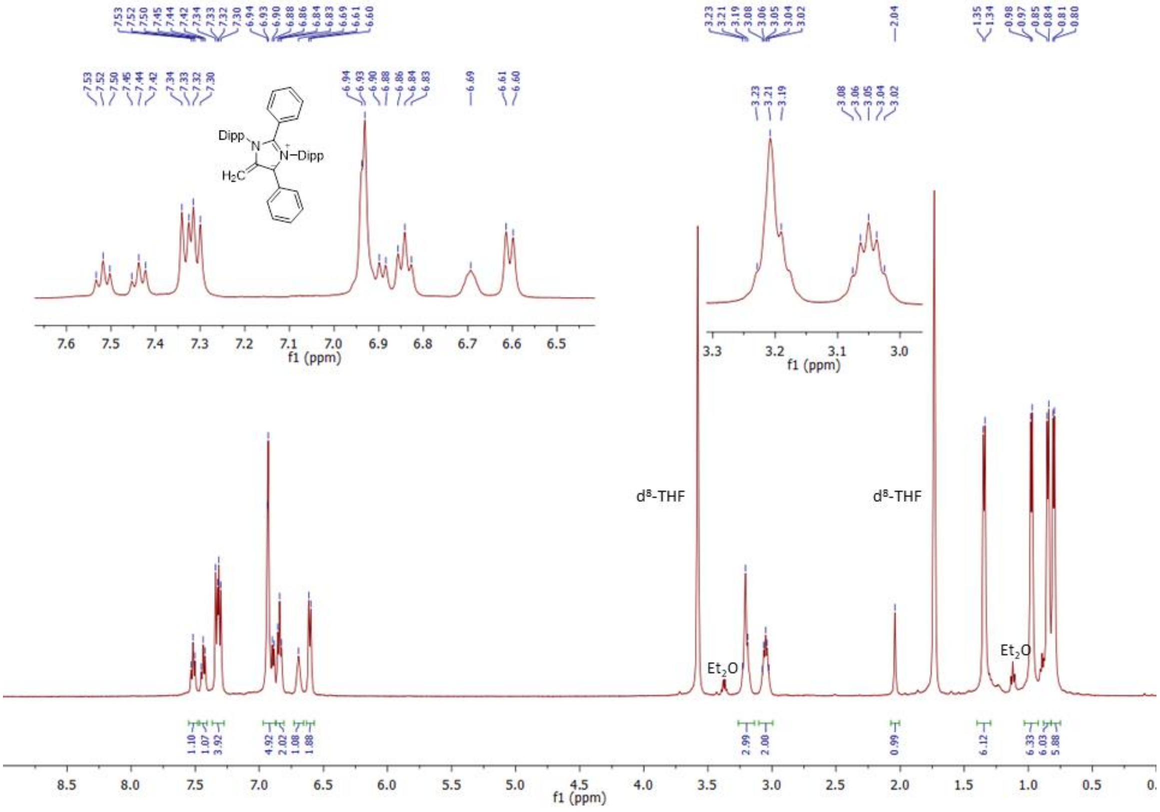

^{1}**H NMR** (500 MHz, d^8-THF, 233K) of **6**

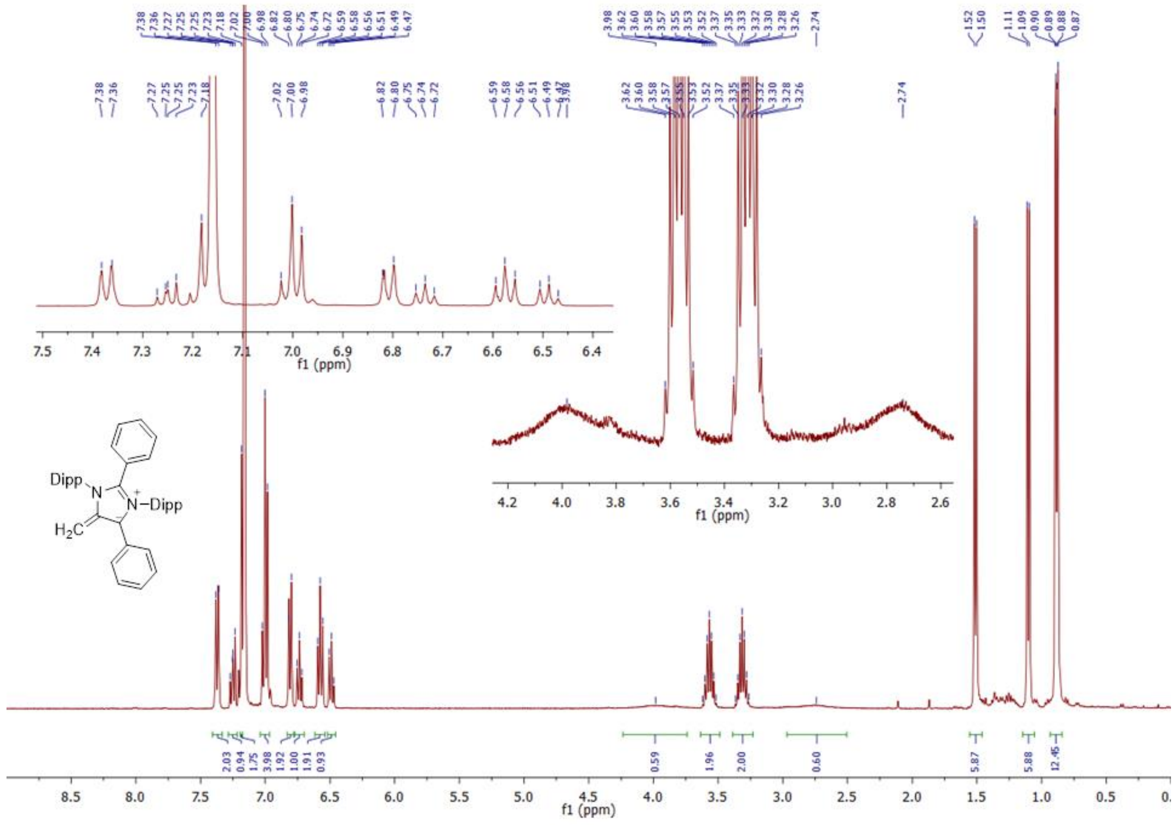

^{1}H NMR (400 MHz, C_6D_6, 295K) of **6**

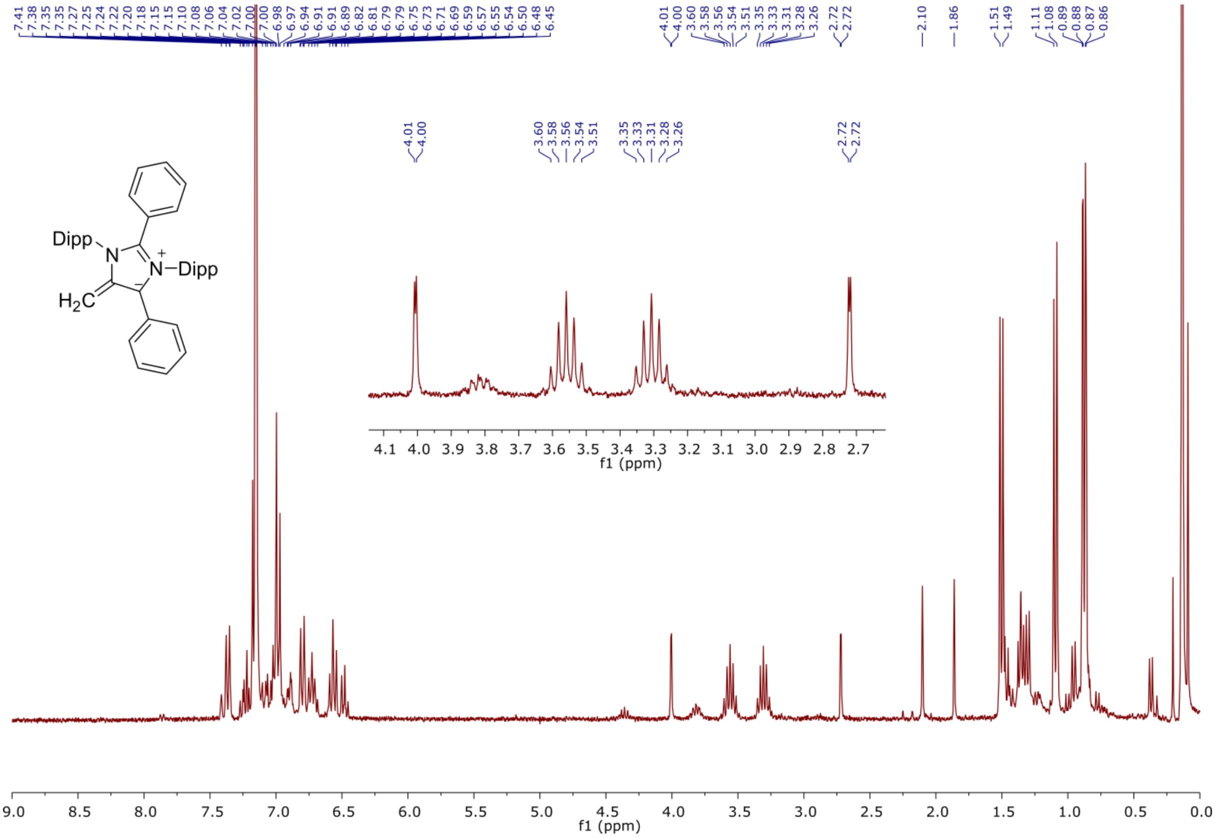

^{1}H NMR (400 MHz, C_6D_6, 298K) of **6** (with additional KHMDS)

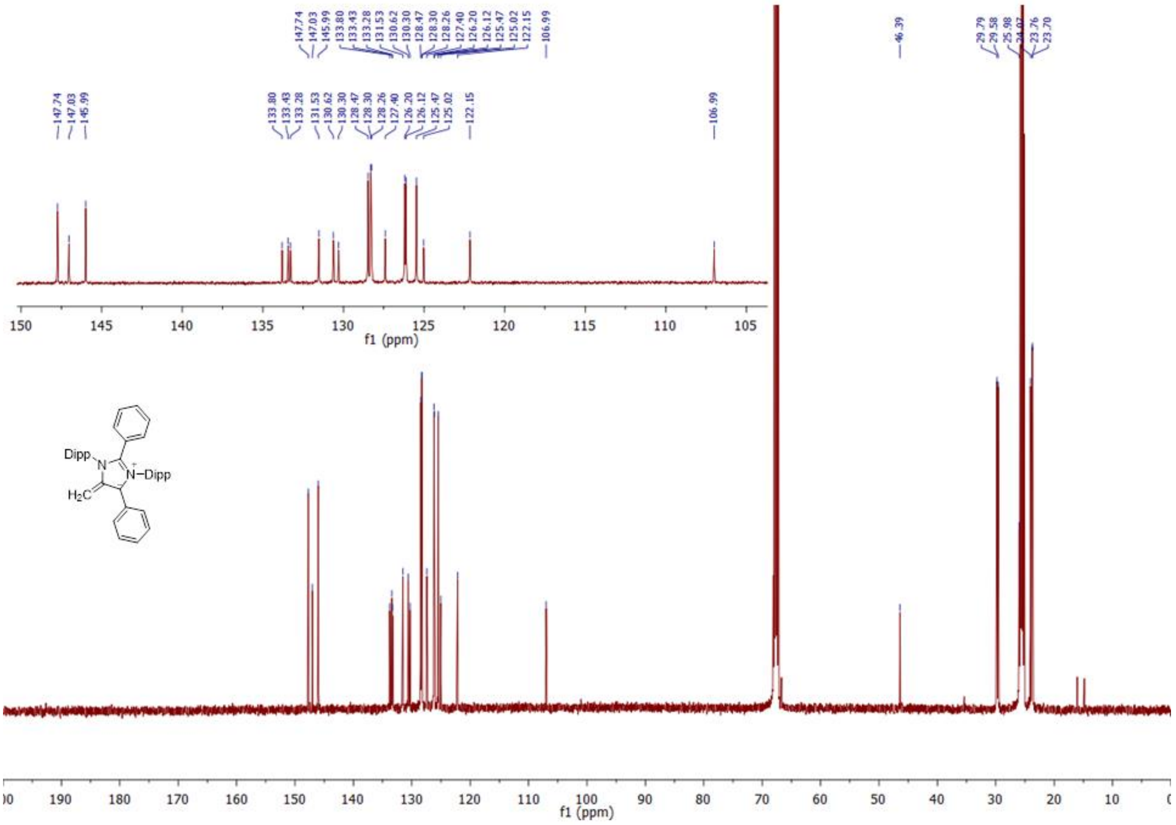

^{13}C {^{1}H} NMR (125 MHz, d^{8}-THF, 233K) of **6**

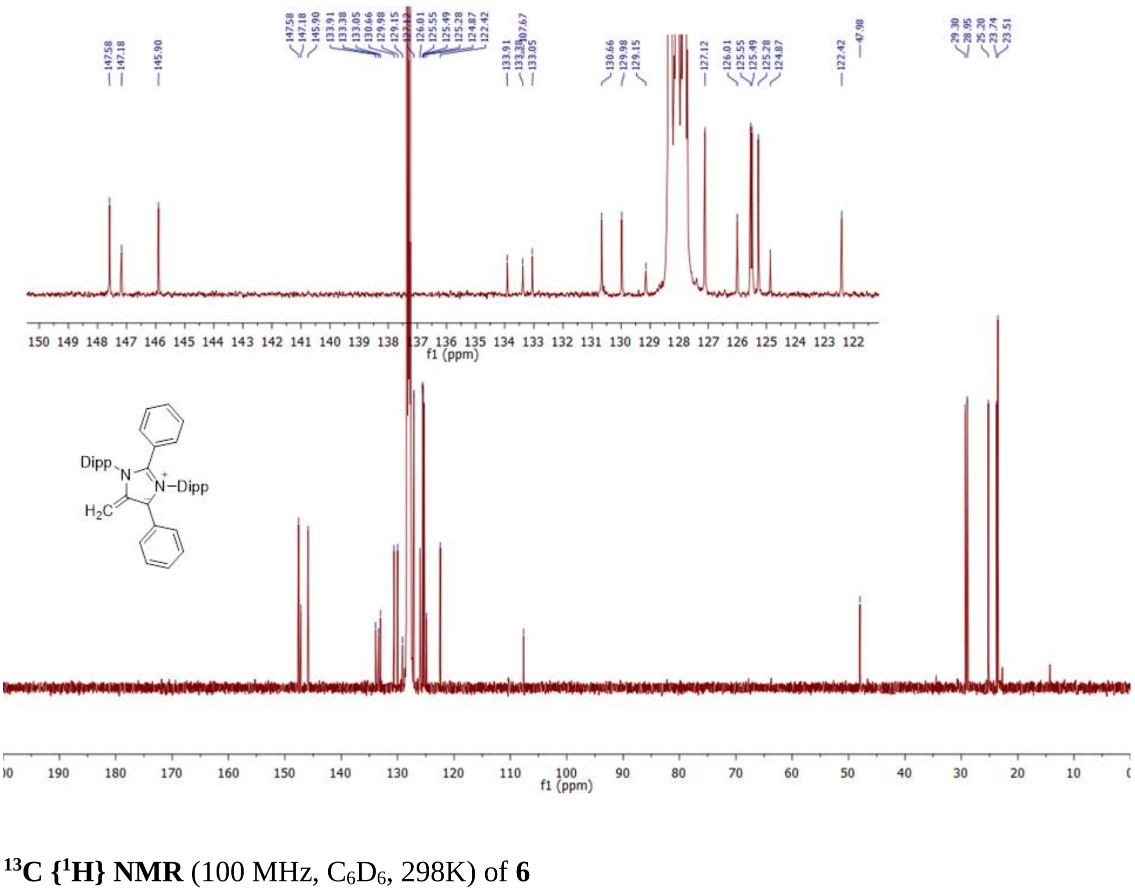

^{13}C {^{1}H} NMR (100 MHz, C_6D_6, 298K) of **6**

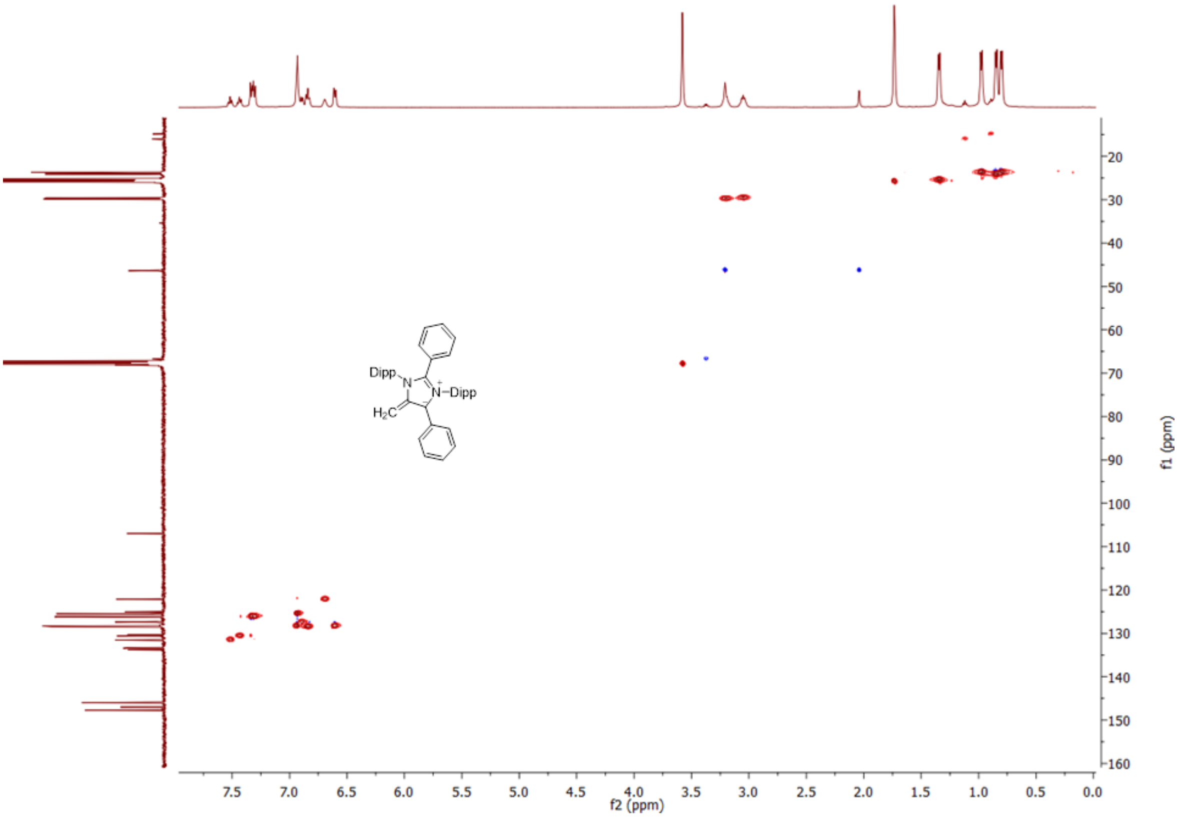

$^{1}H/^{13}C$ HSQC (500/125 MHz, d^8-THF, 233K) of **6**

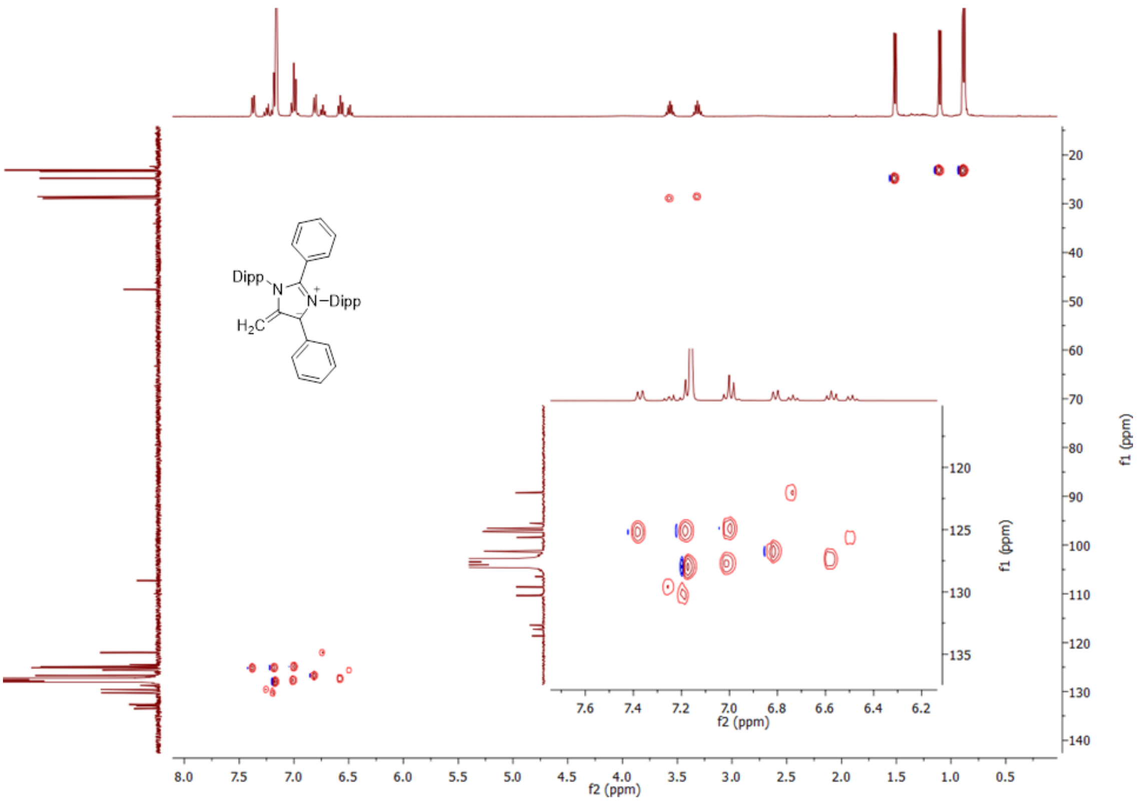

$^{1}H/^{13}C$ HSQC (400/100 MHz, C_6D_6, 298K) of **6**

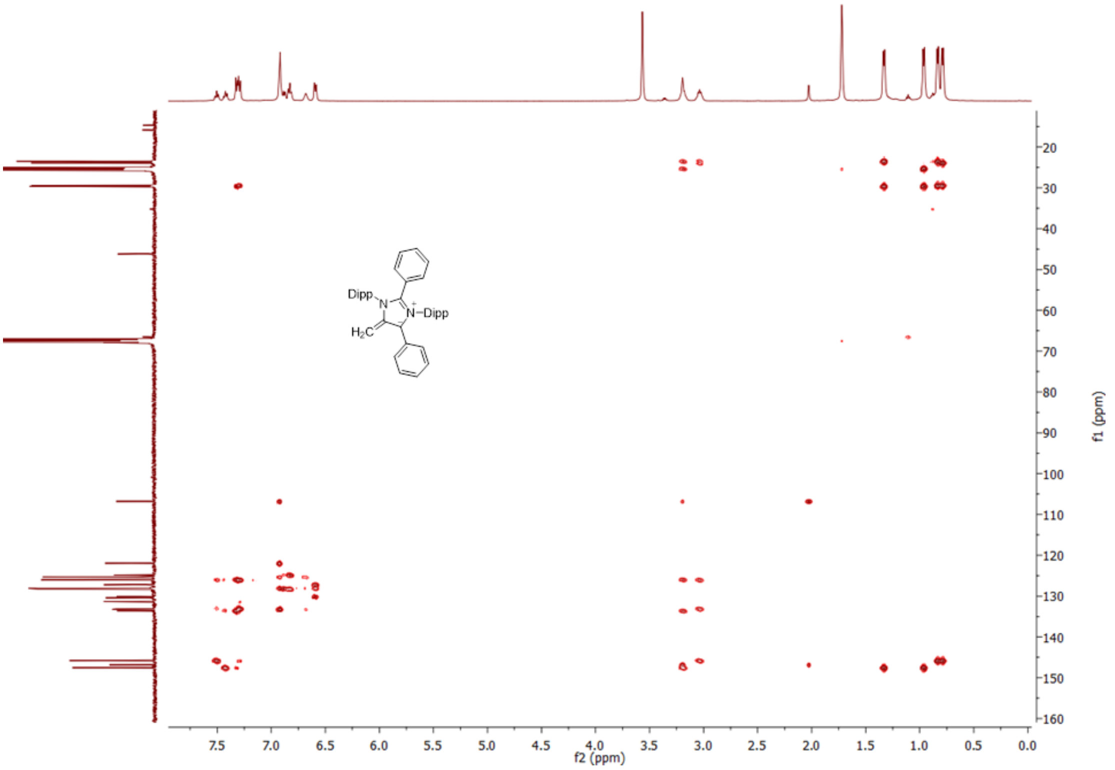

$^{1}H/^{13}C$ HMBC (500/125 MHz, d^{8}-THF, 233K) of **6**

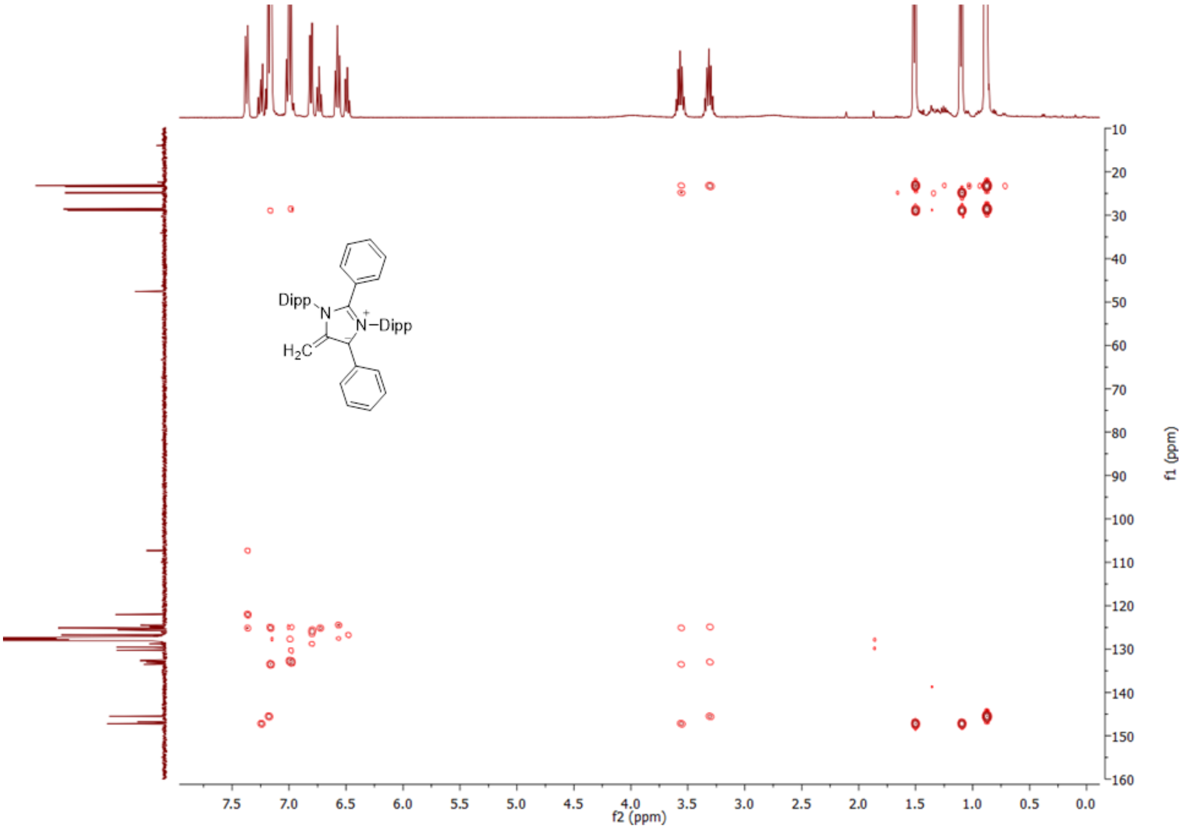

$^{1}H/^{13}C$ HMBC (400/100 MHz, C_6D_6, 298K) of **6**

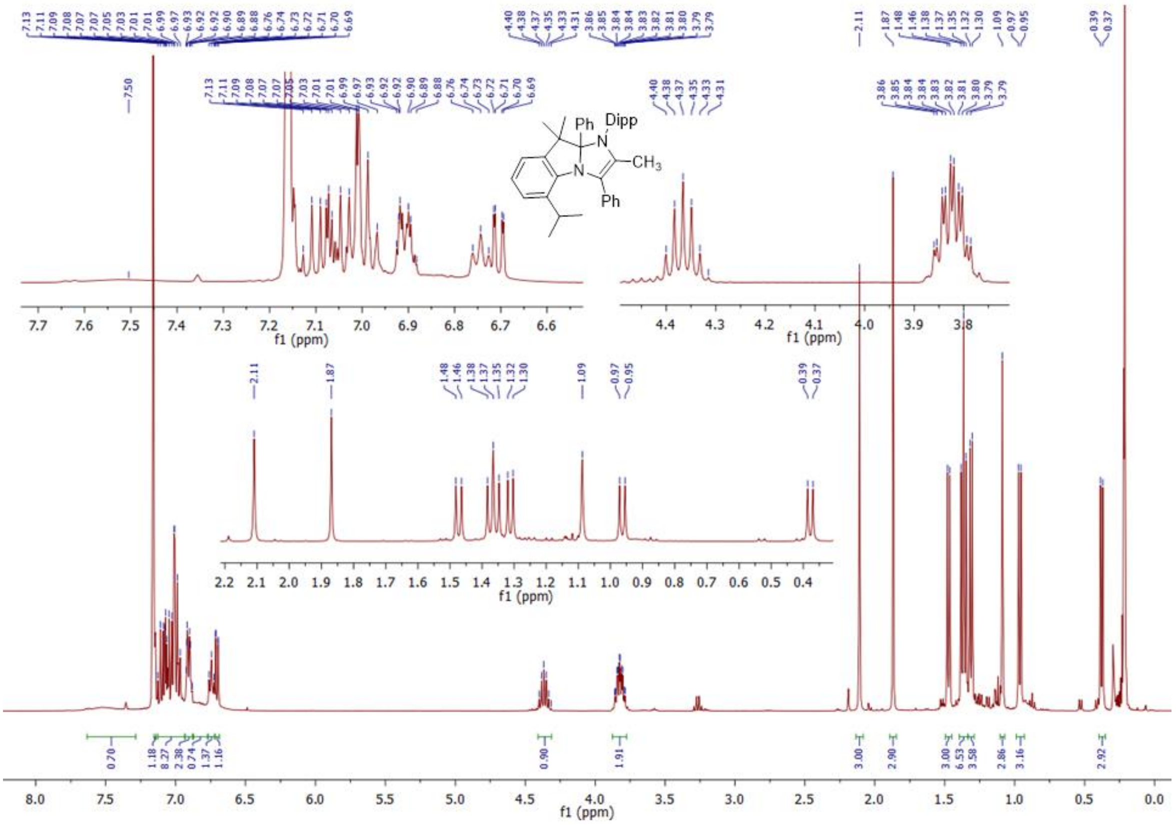

^{1}H NMR (400 MHz, C_6D_6, 298K) of **7**

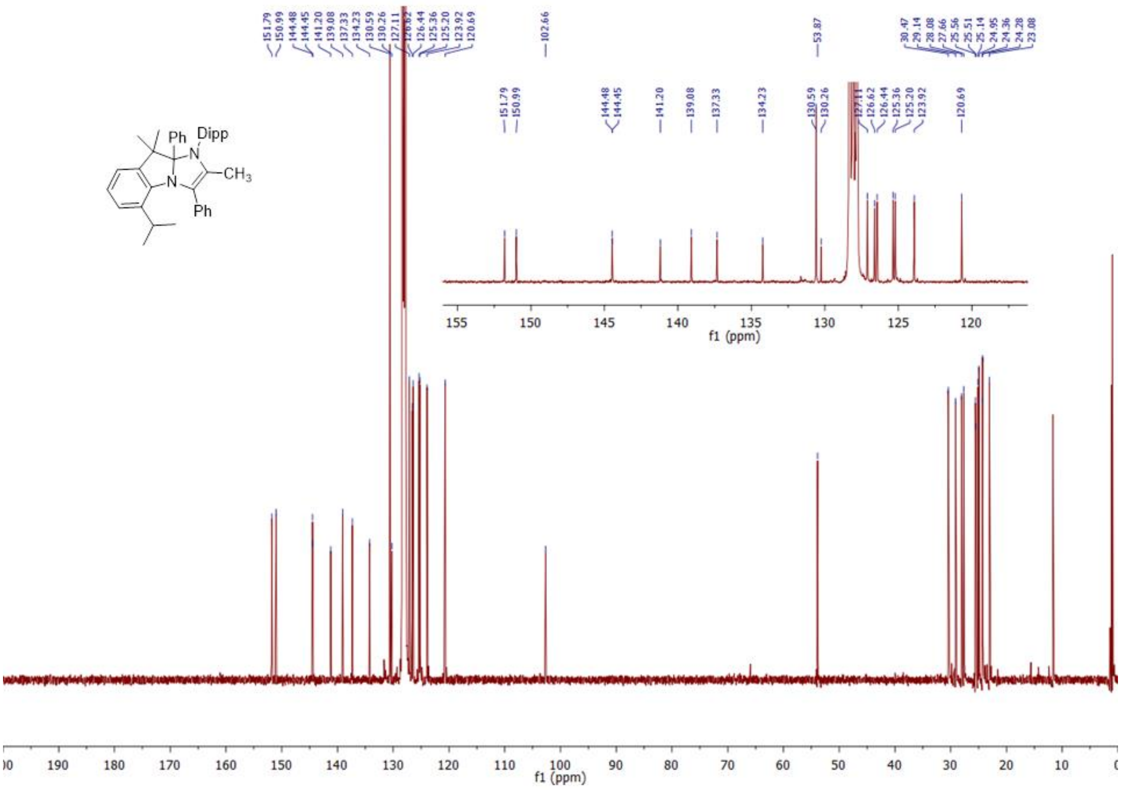

^{13}C {^{1}H} NMR (100 MHz, C_6D_6, 298K) of **7**

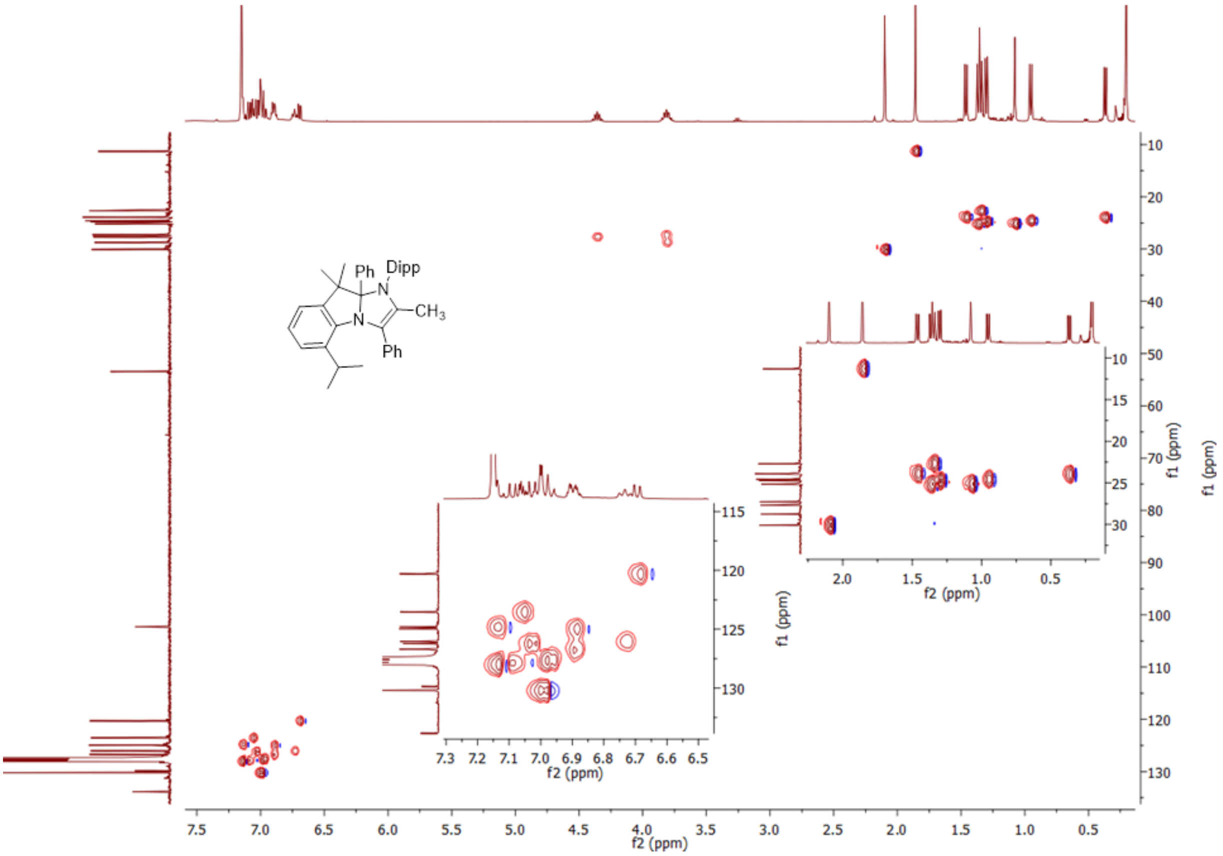

$^{1}H/^{13}C$ **HSQC** (400/100 MHz, C_6D_6, 298K) of **7**

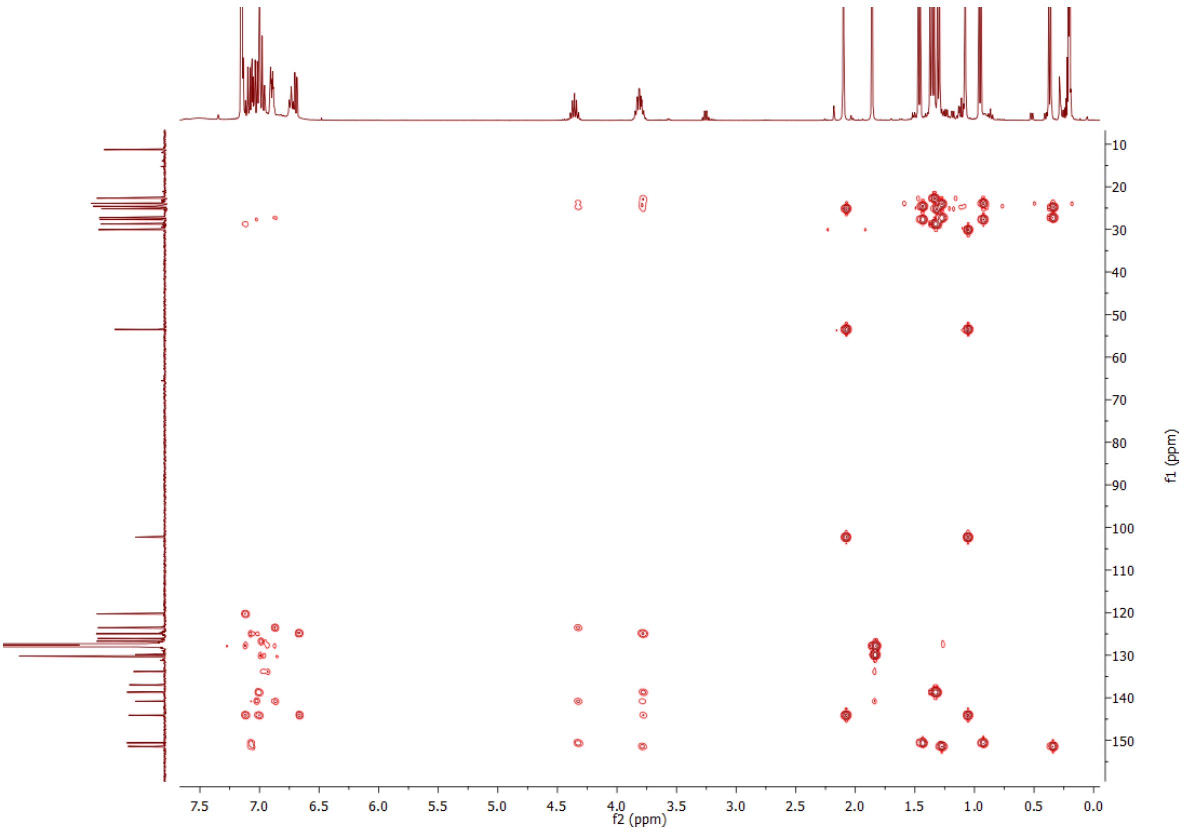

$^{1}H/^{13}C$ **HMBC** (400/100 MHz, C_6D_6, 298K) of **7**

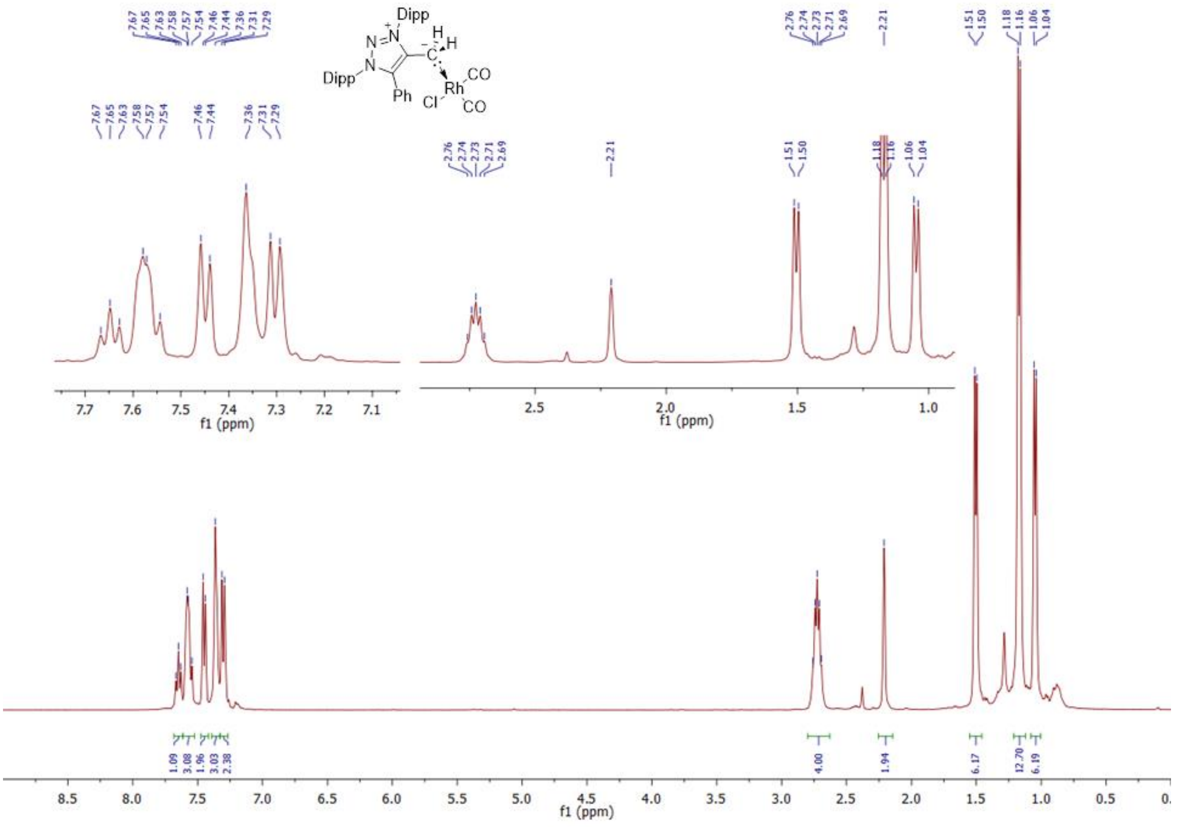

¹H NMR (400 MHz, $CDCl_3$, 295K) of **8**

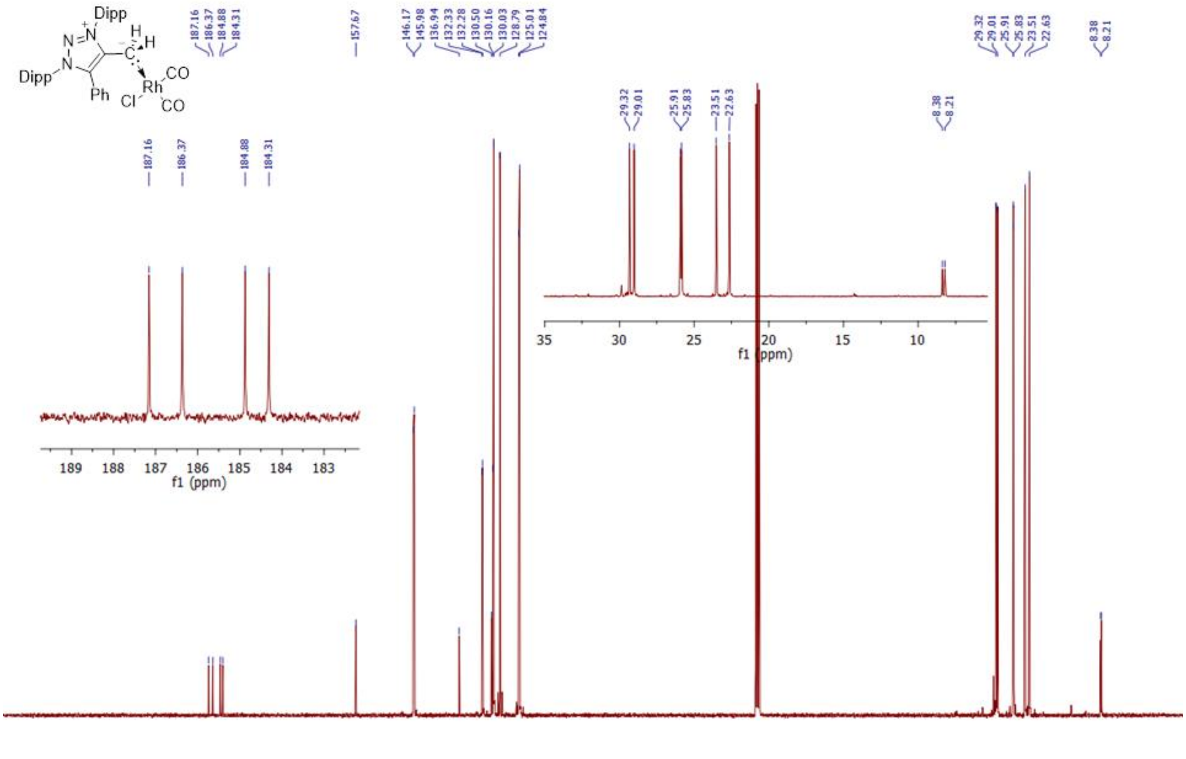

¹³C {¹H} NMR (100 MHz, $CDCl_3$, 295K) of **8**

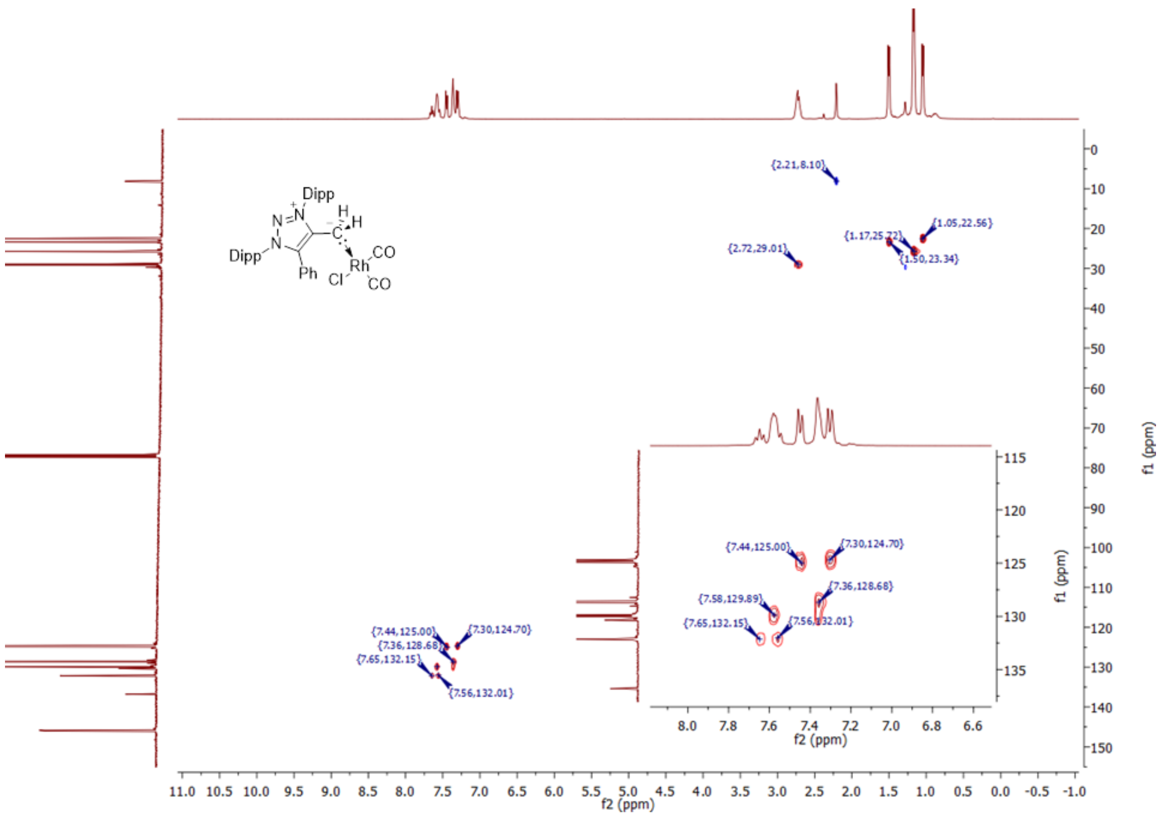

^{1}H/^{13}C HSQC (400/100 MHz, $CDCl_3$, 295K) of **8**

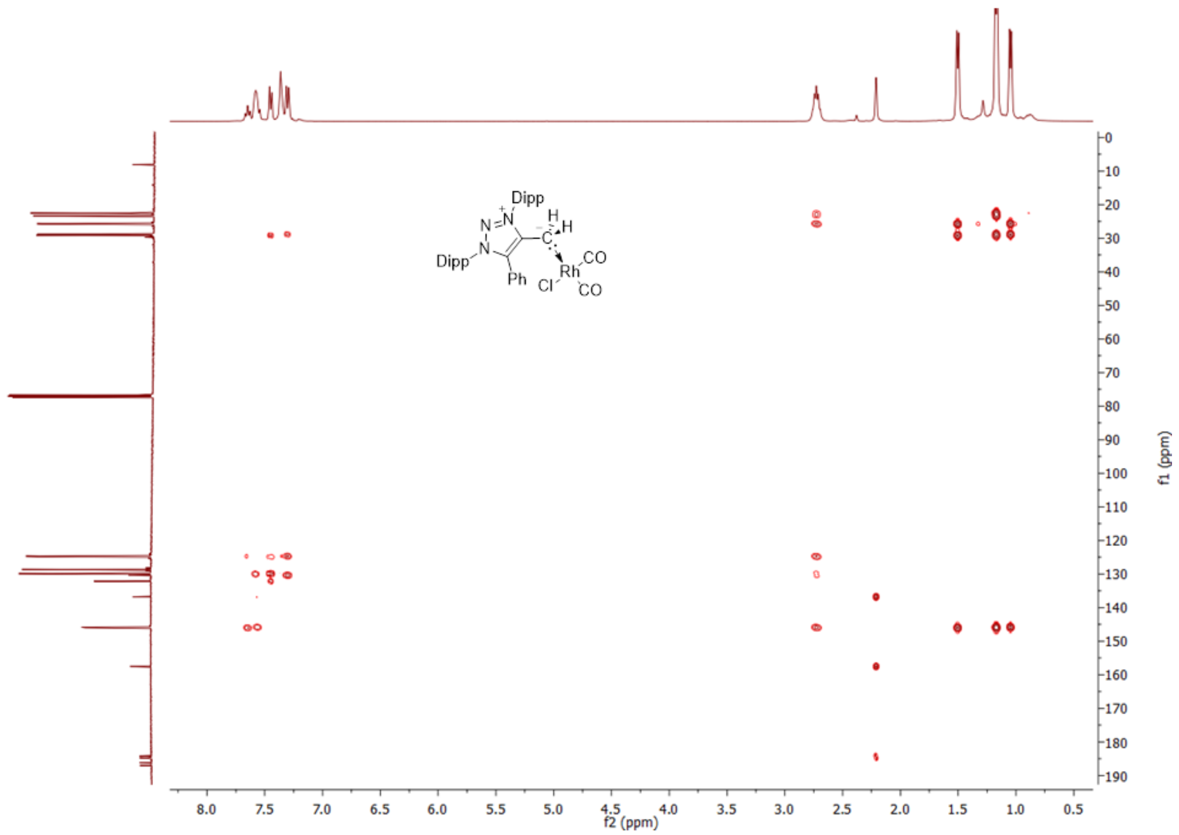

^{1}H/^{13}C HMBC (400/100 MHz, $CDCl_3$, 295K) of **8**

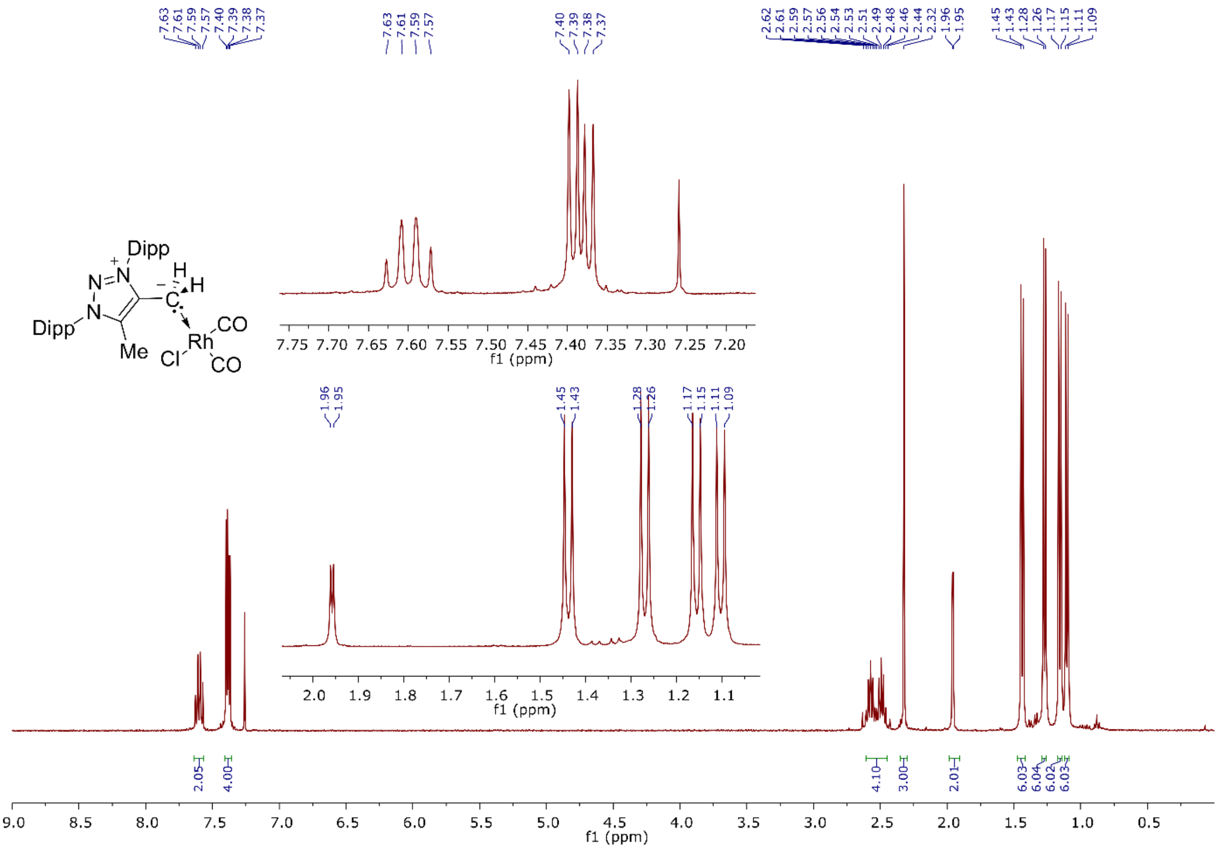

^{1}**H NMR** (400 MHz, CDCl$_3$, 298 K) of **9**

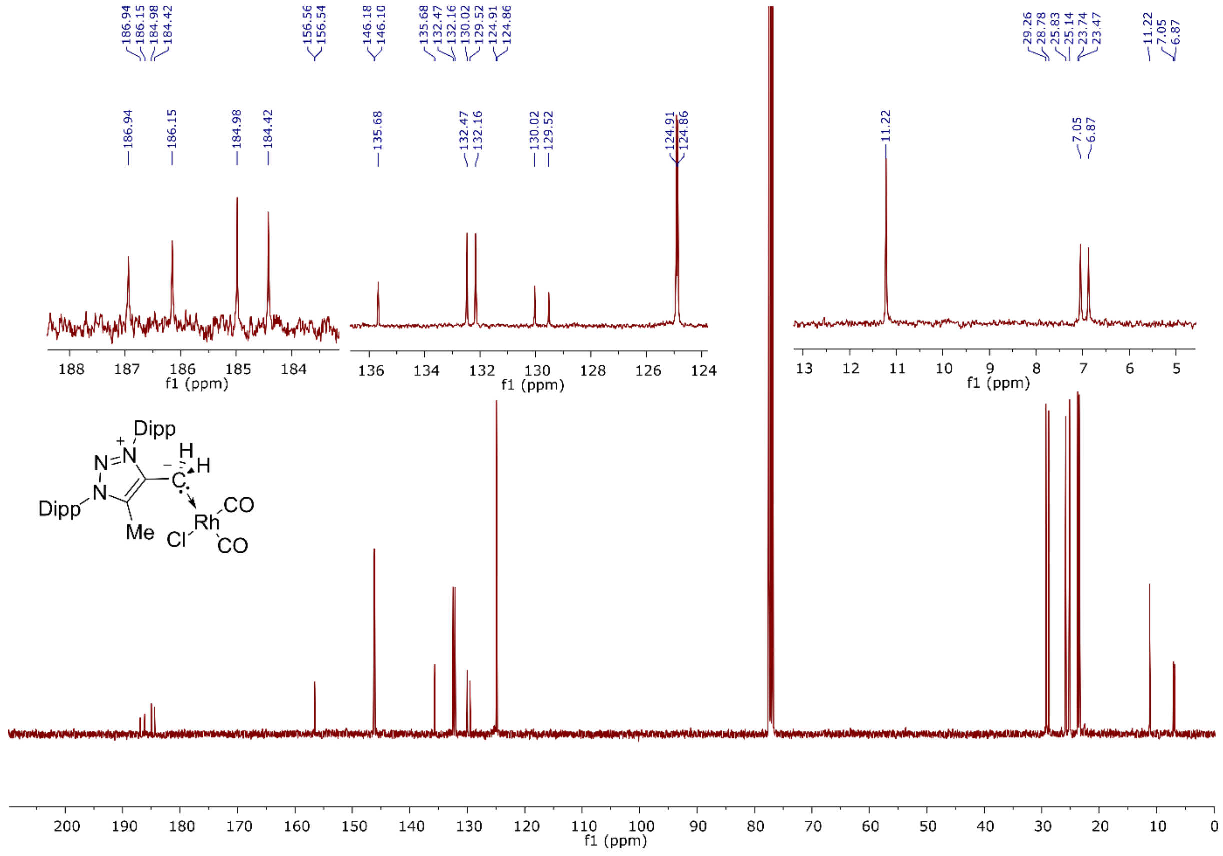

^{13}**C {^{1}H} NMR** (100 MHz, CDCl$_3$, 298 K) of **9**

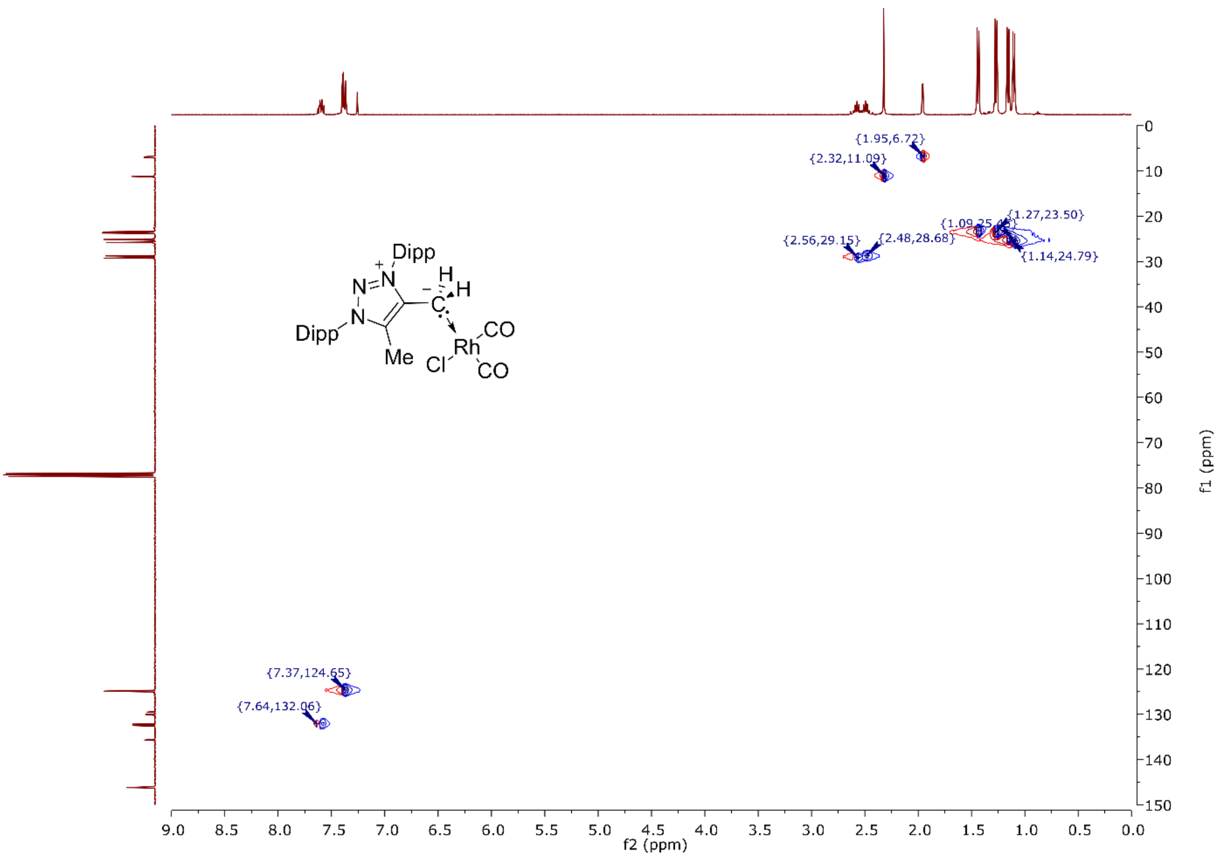

$^{1}H/^{13}C$ **HSQC** (400/100 MHz, $CDCl_3$, 298K) of **9**

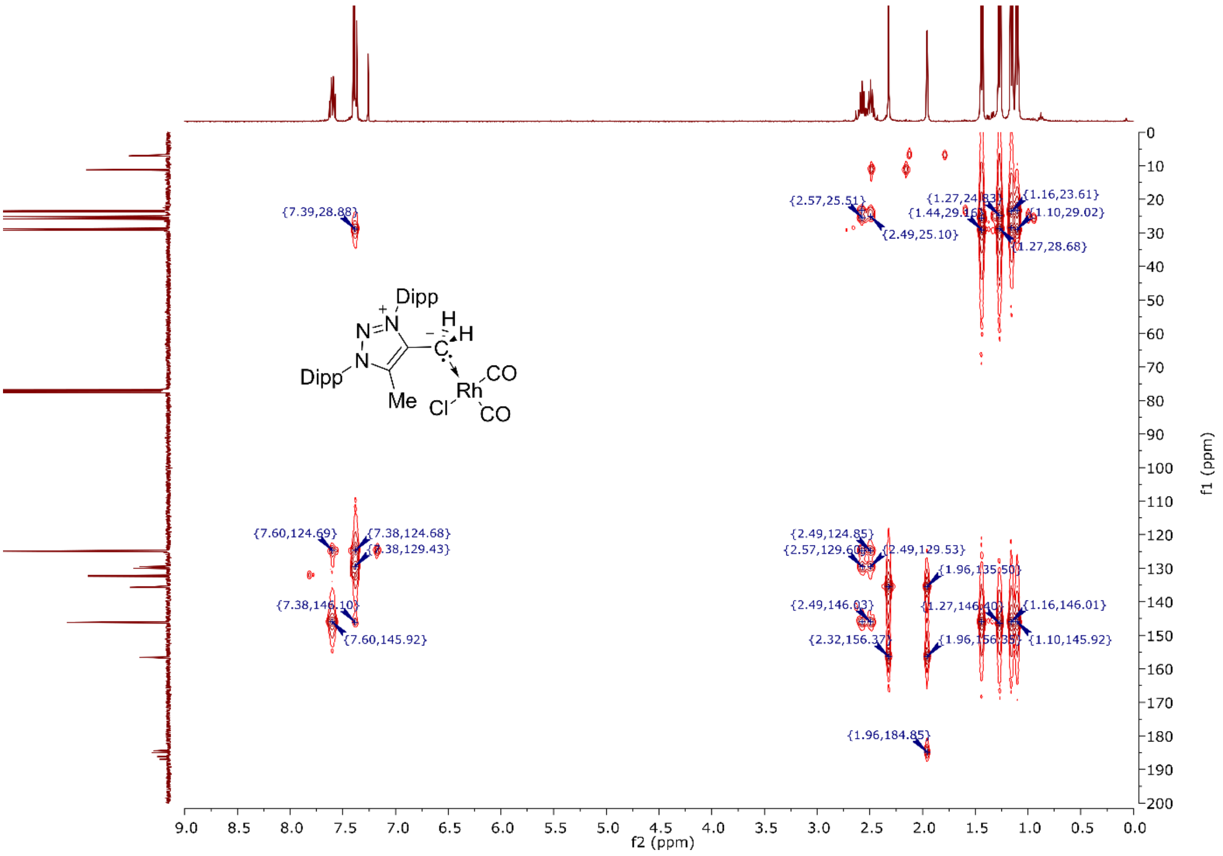

$^{1}H/^{13}C$ **HMBC** (400/100 MHz, $CDCl_3$, 298K) of **9**

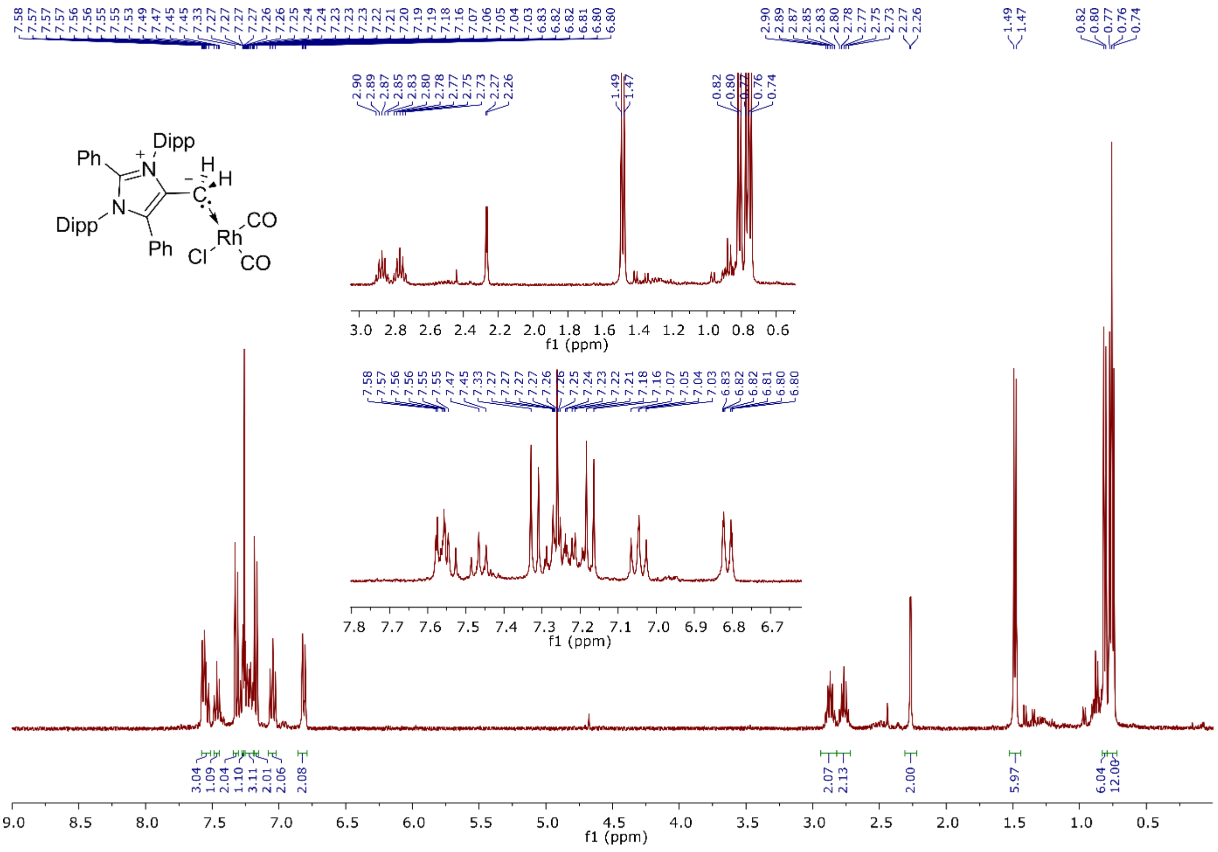

^{1}H NMR (400 MHz, $CDCl_3$, 298K) of **10**

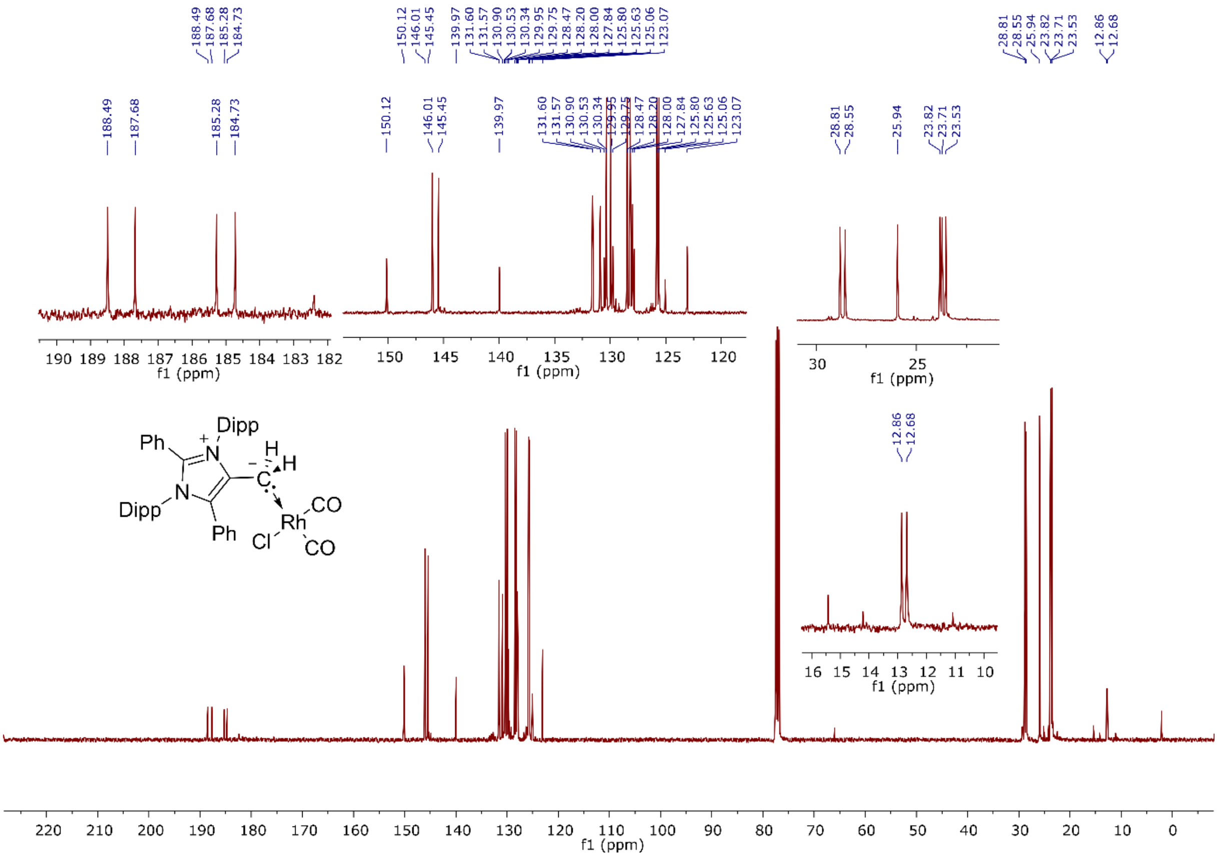

^{1}H NMR (400 MHz, $CDCl_3$, 298K) of **10**

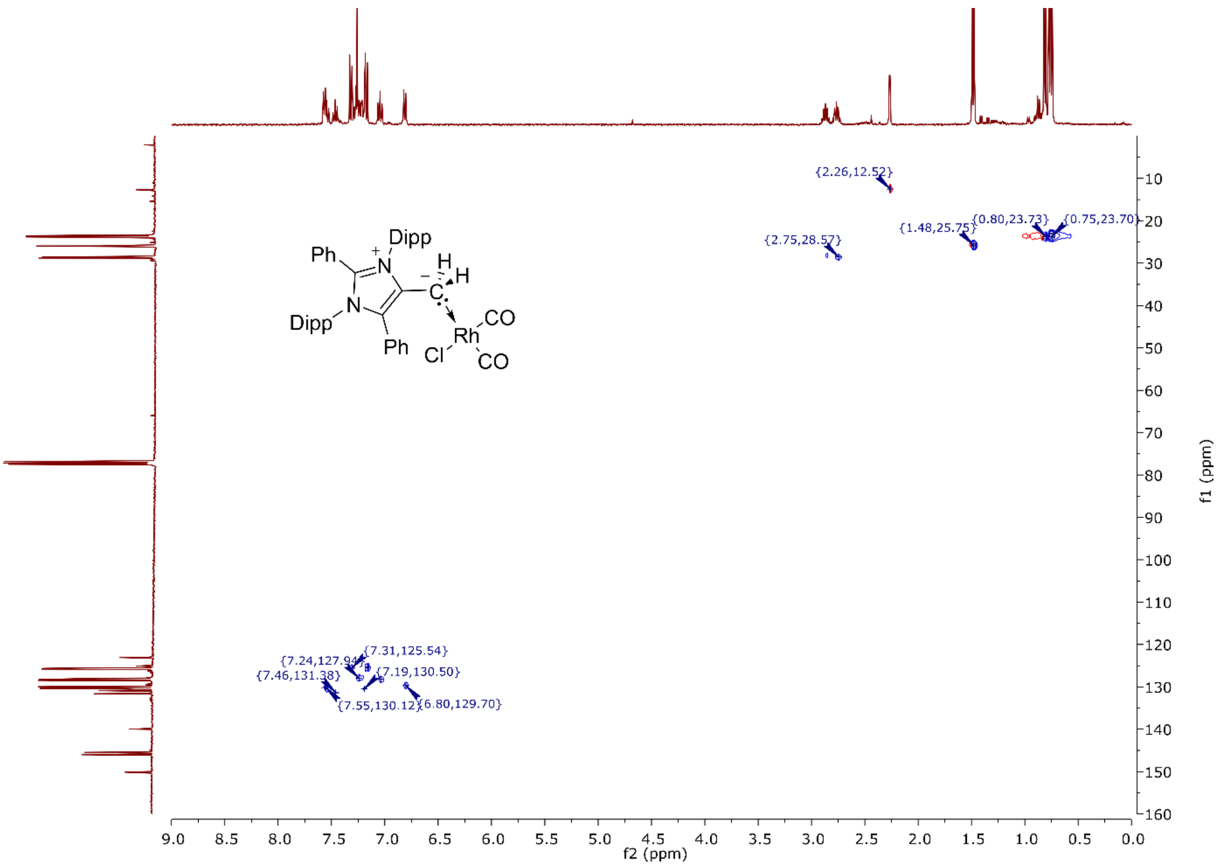

$^{1}H/^{13}C$ HSQC (400/100 MHz, $CDCl_3$, 298K) of **10**

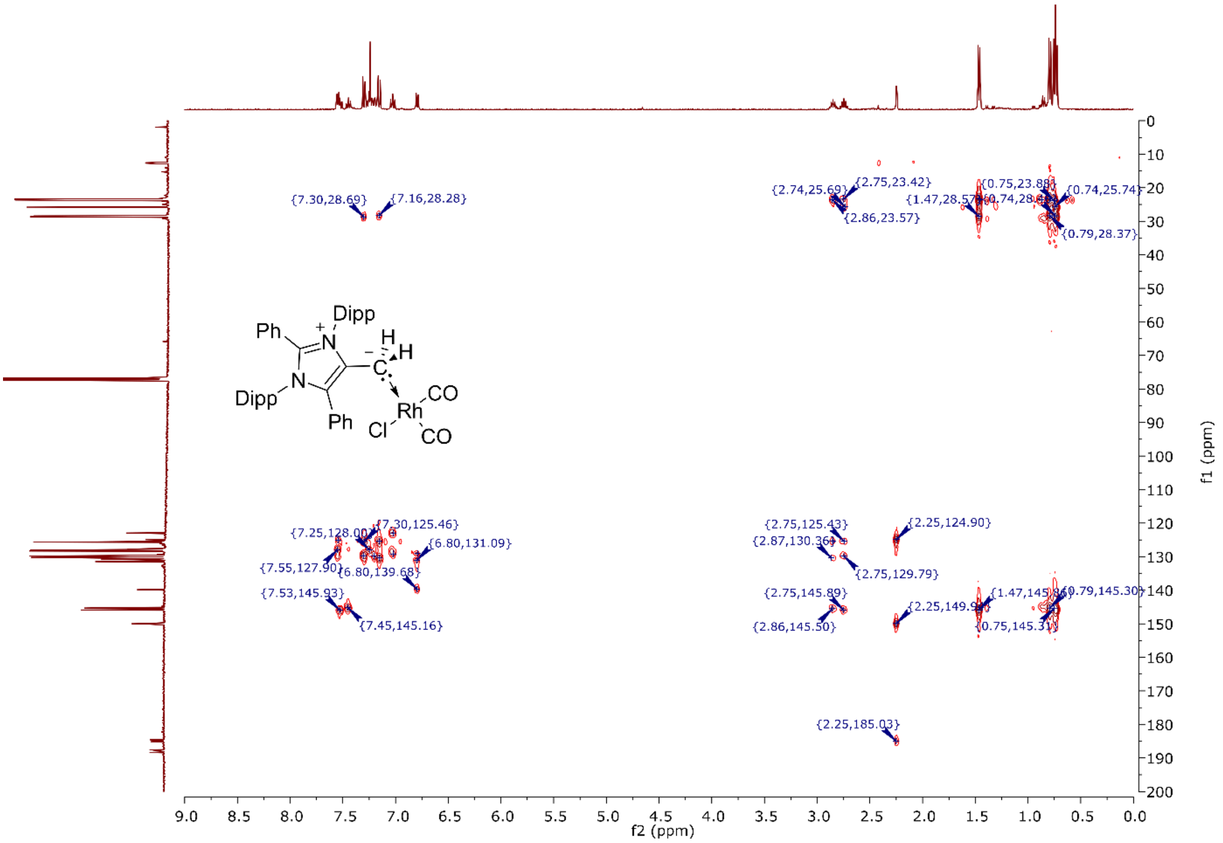

$^{1}H/^{13}C$ HMBC (400/100 MHz, $CDCl_3$, 298K) of **10**

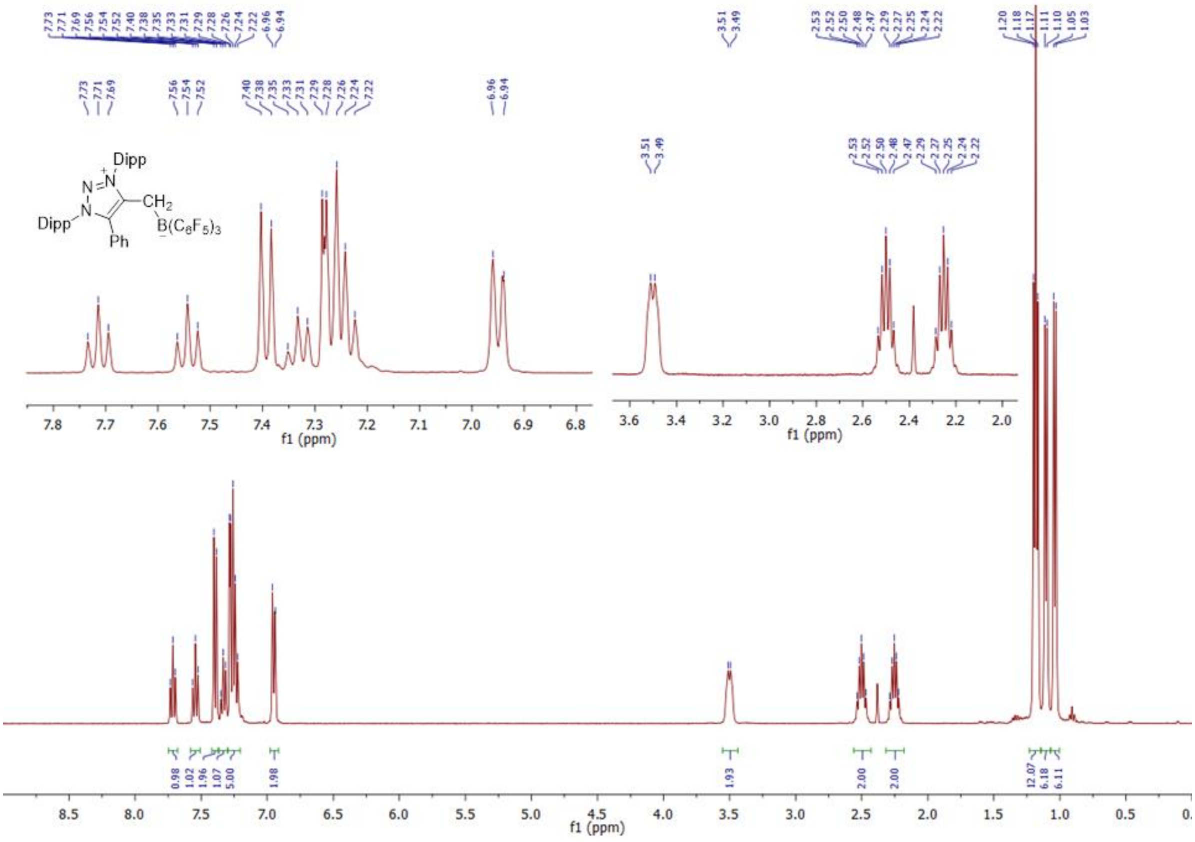

^{1}H NMR (400 MHz, $CDCl_3$, 295K) of **11**

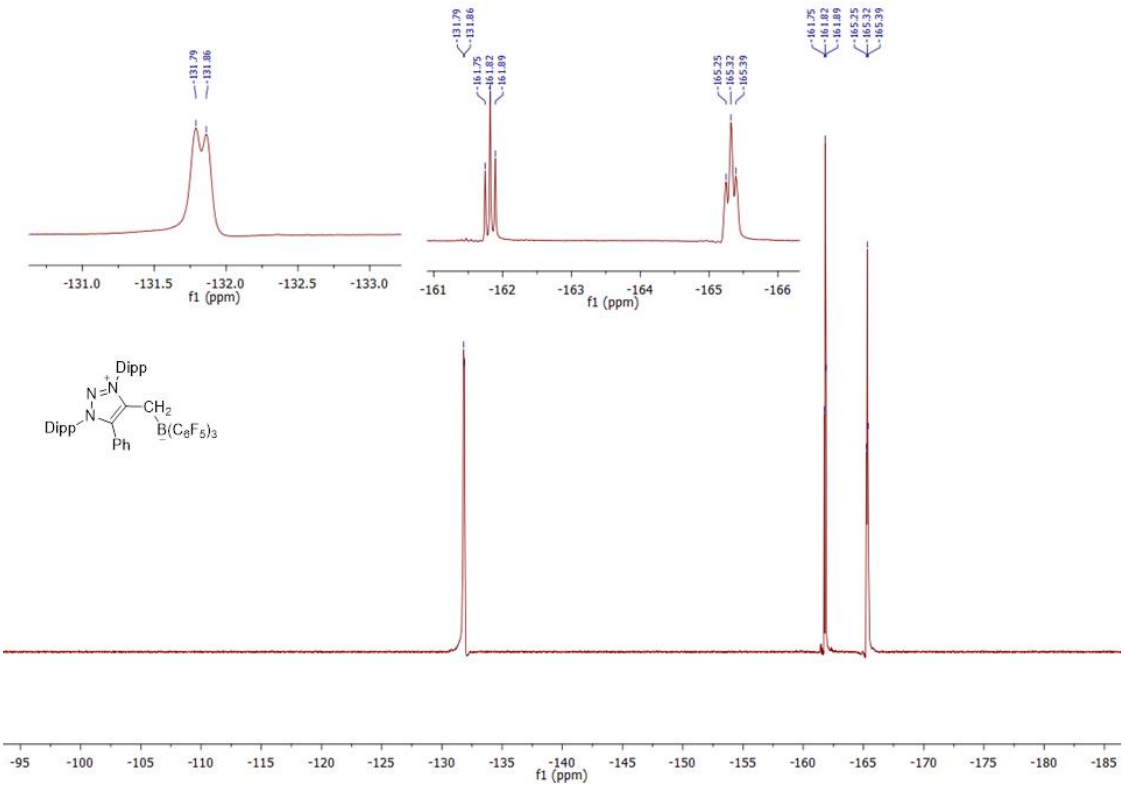

^{19}F NMR (282 MHz, $CDCl_3$, 295K) of **11**

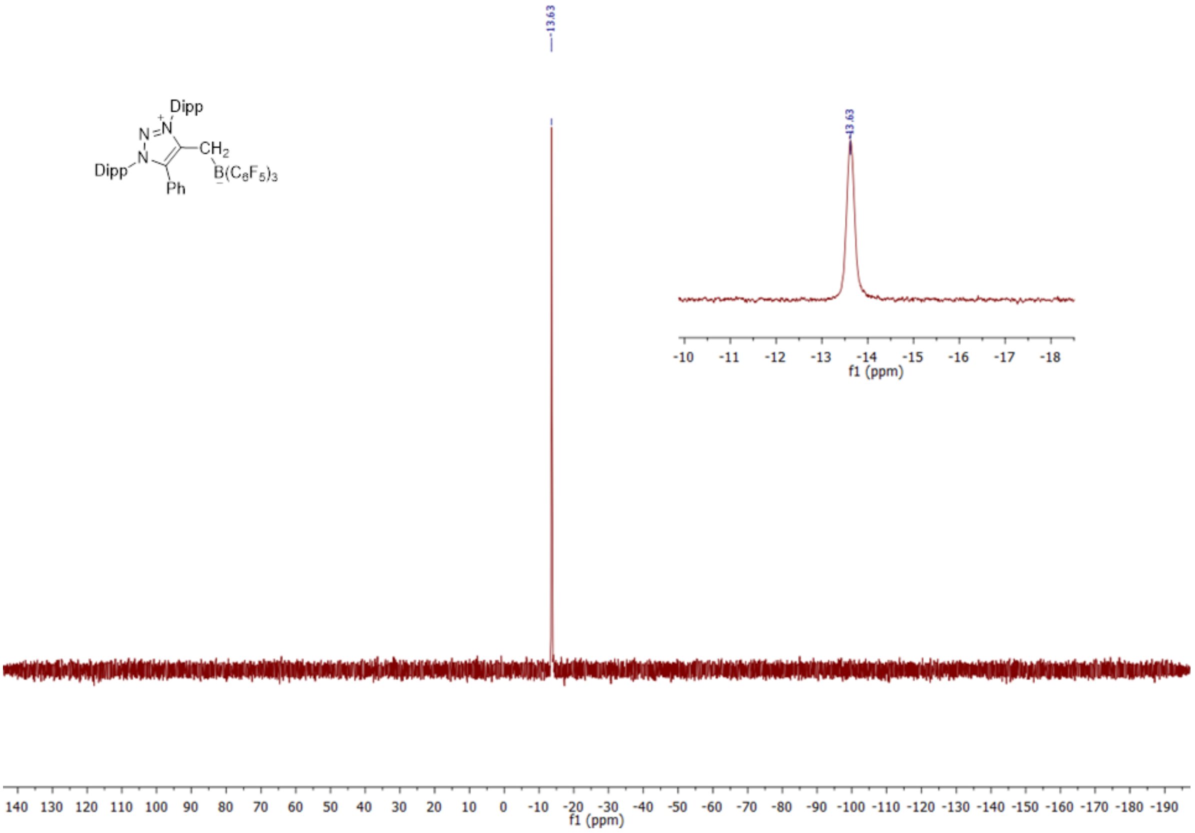

^{11}B NMR (96 MHz, $CDCl_3$, 295K) of **11**

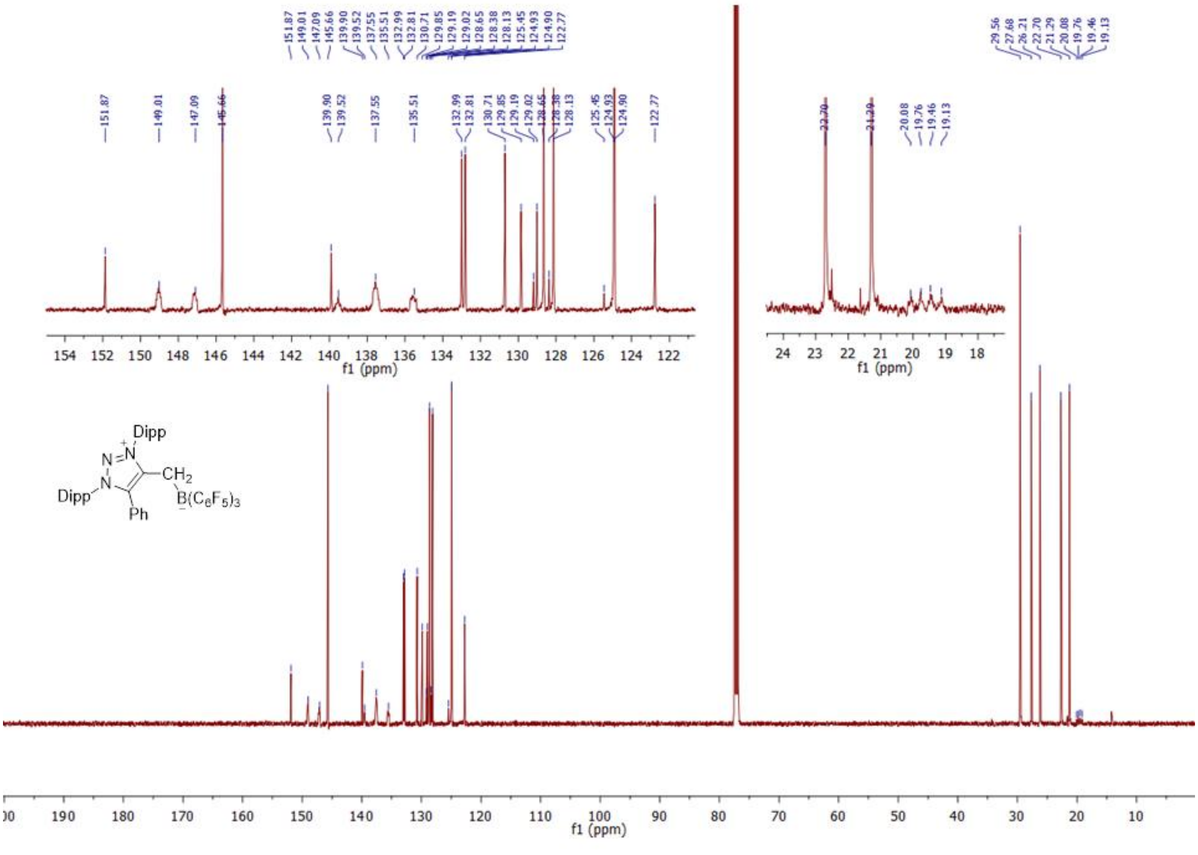

^{13}C {^{1}H} NMR (125 MHz, $CDCl_3$, 295K) of **11**

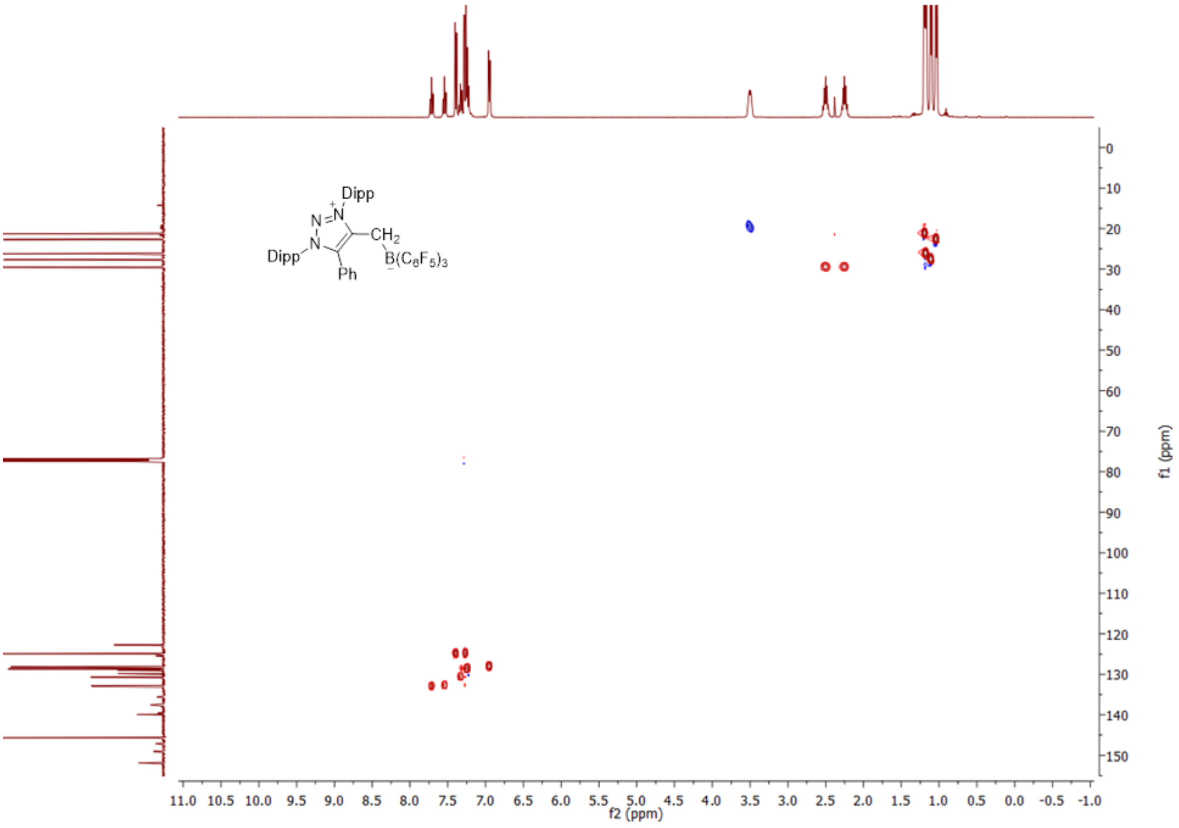

$^{1}H/^{13}C$ HSQC (400/100 MHz, $CDCl_3$, 295K) of **11**

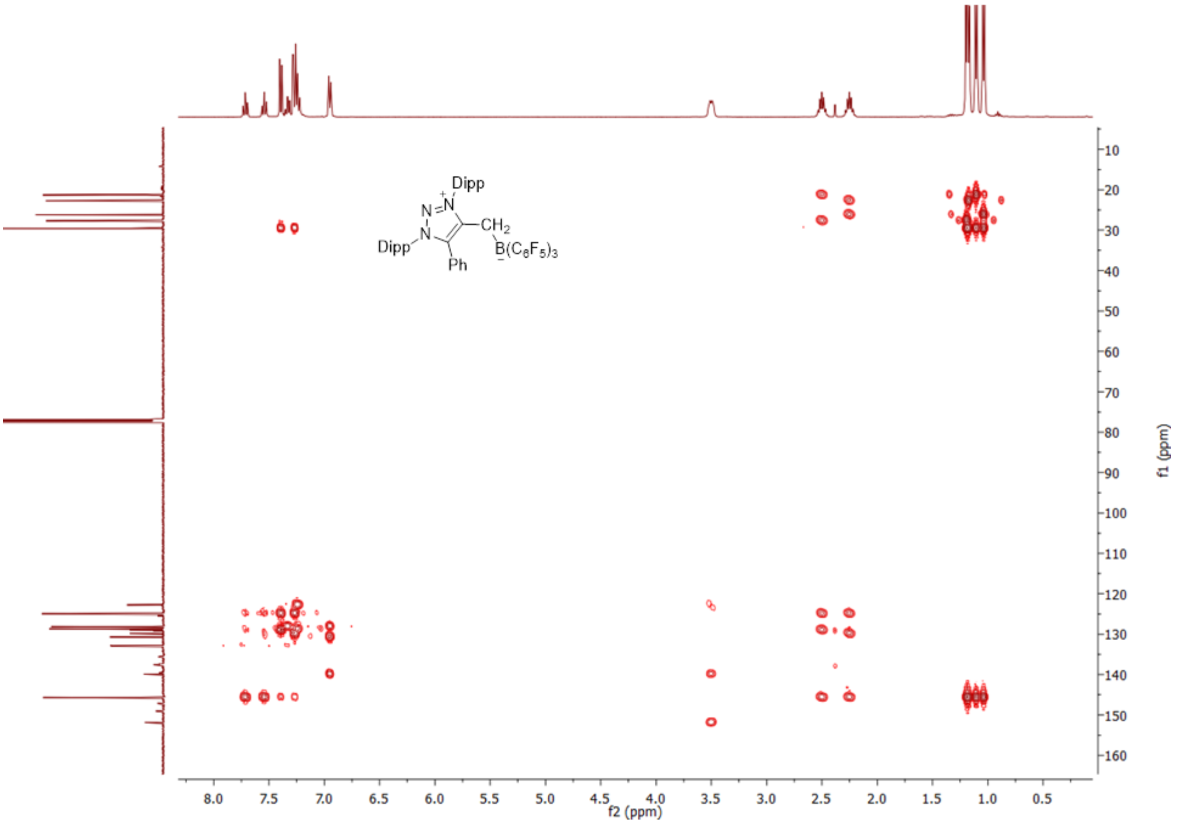

$^{1}H/^{13}C$ HMBC (400/100 MHz, $CDCl_3$, 295K) of **11**

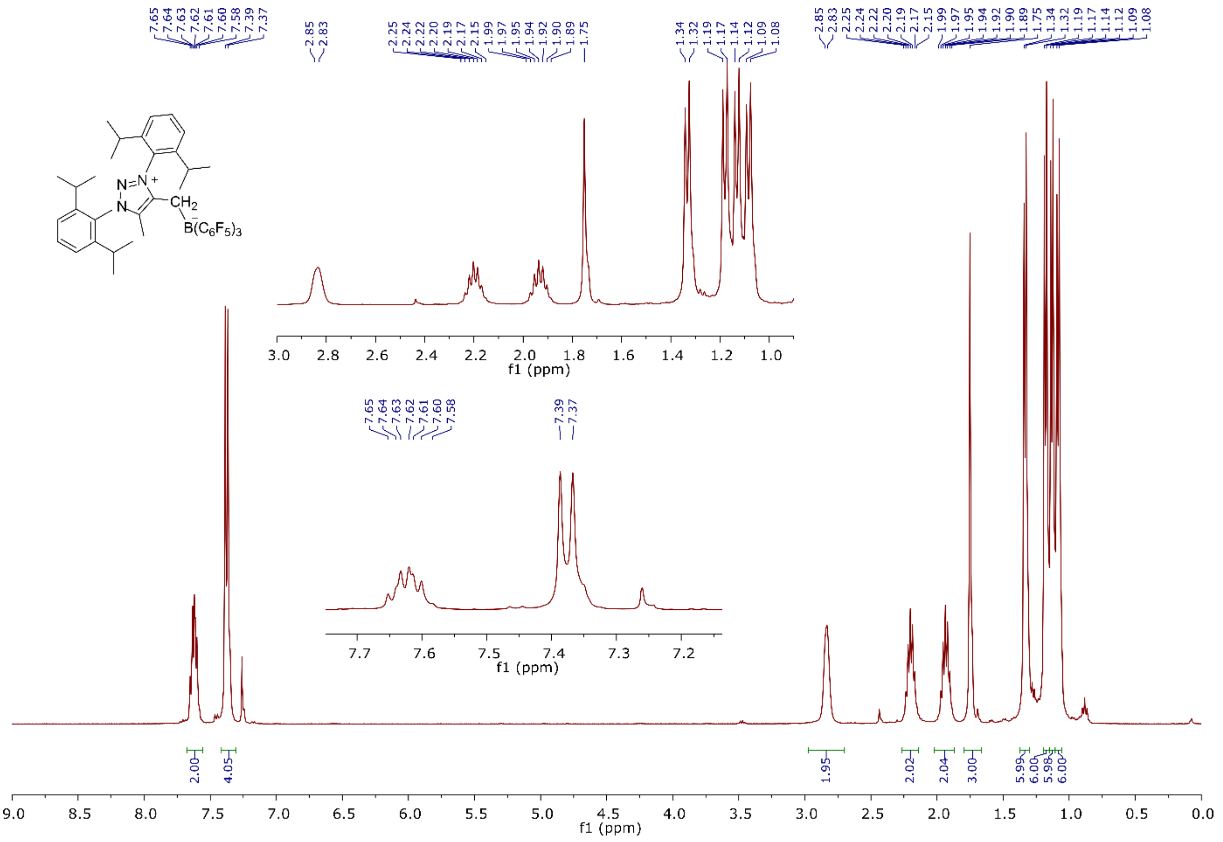

¹H NMR (600 MHz, C_6D_6, 295K) of **12**

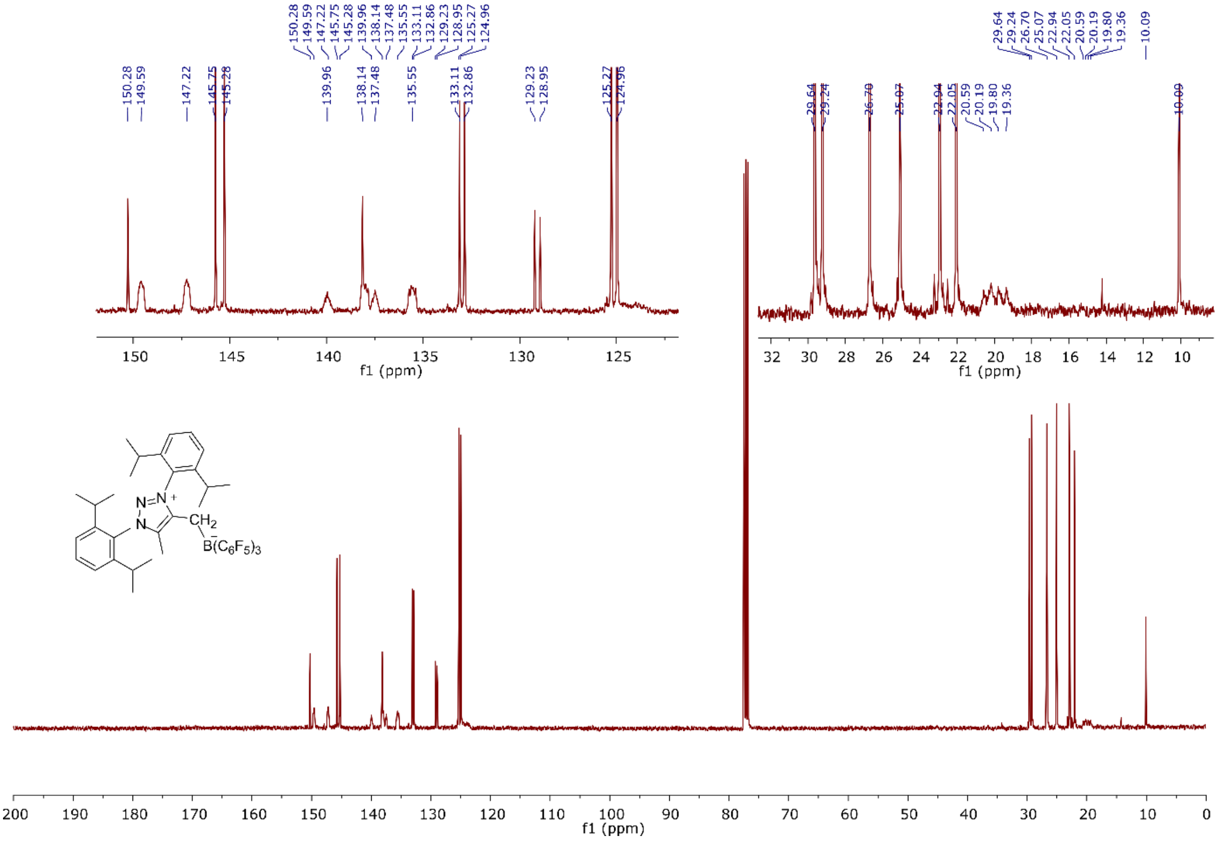

¹³C {¹H} NMR (100 MHz, CD_3CN, 208K) of **12**

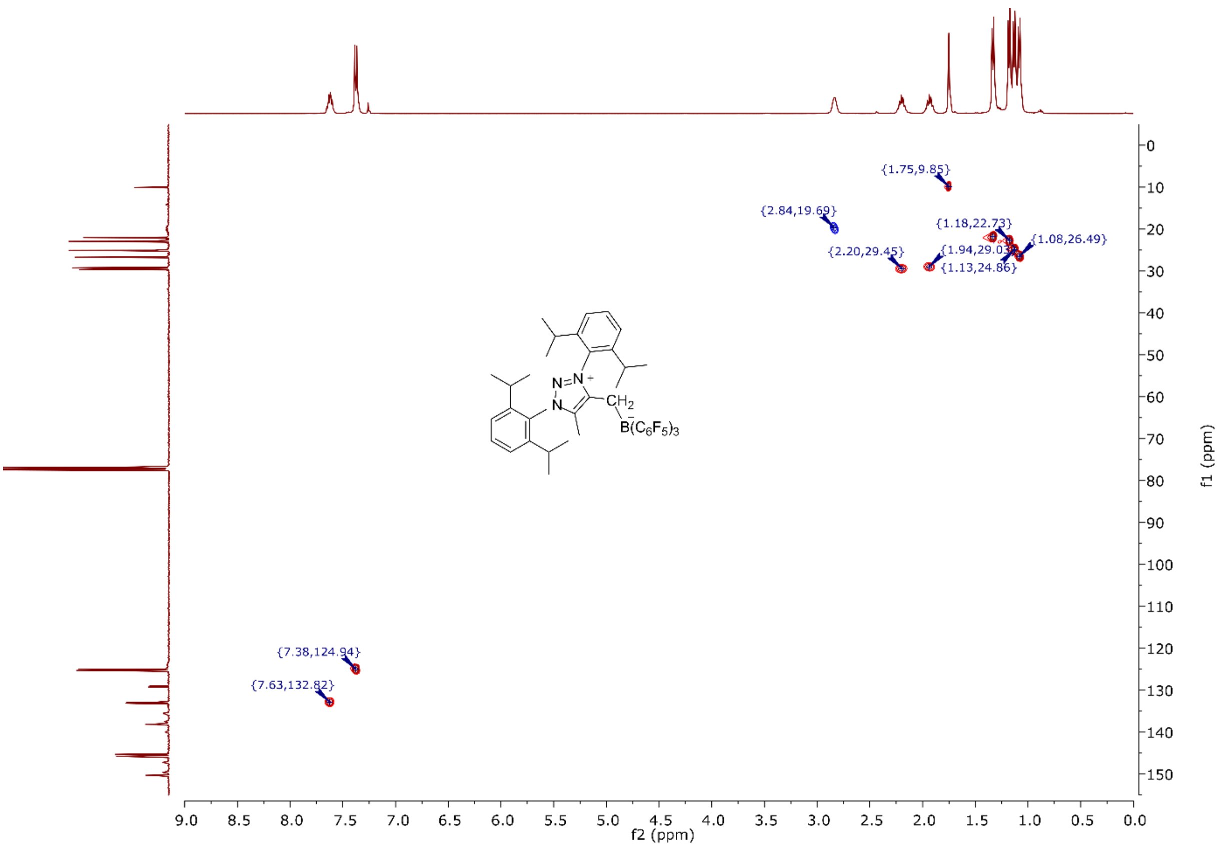

^{1}H/^{13}C HSQC (400/100 MHz, CD_3CN, 298K) of **12**

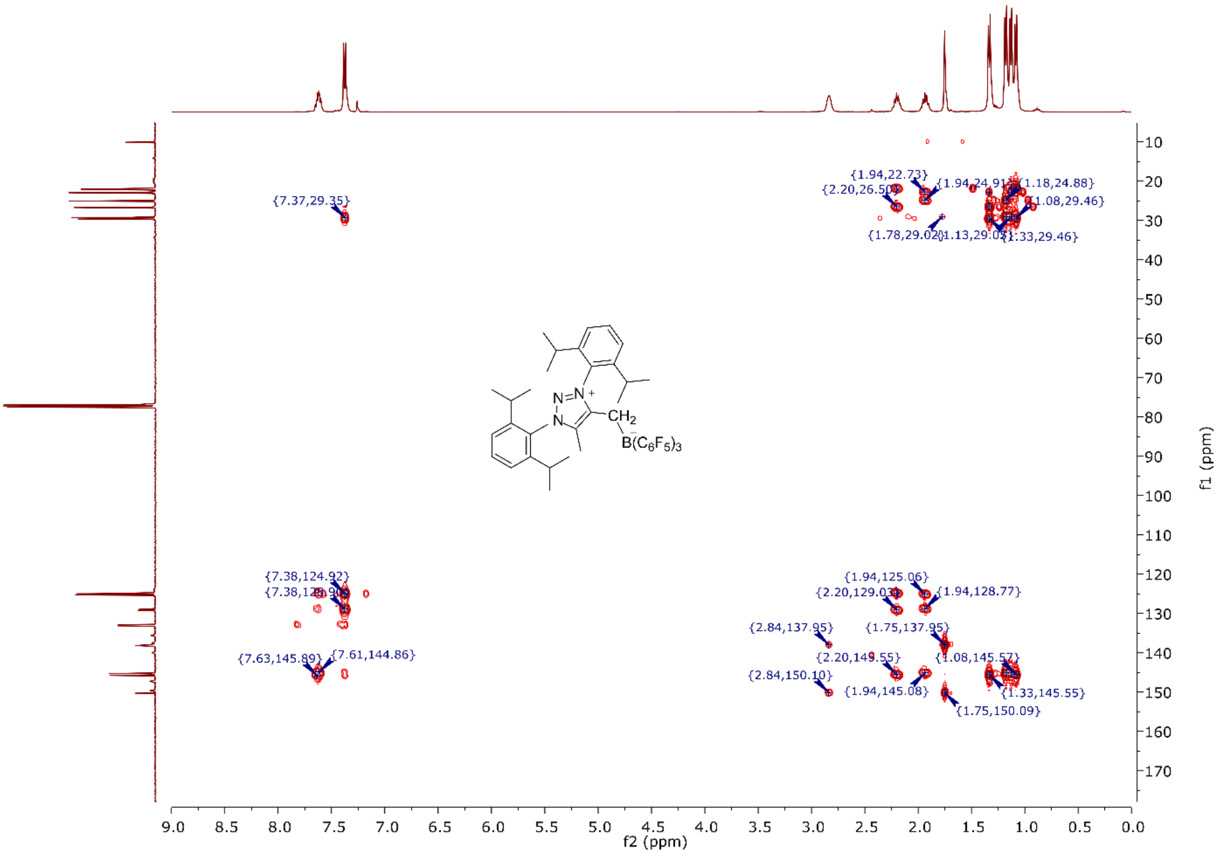

^{1}H/^{13}C HMBC (400/100 MHz, CD_3CN, 298K) of **12**

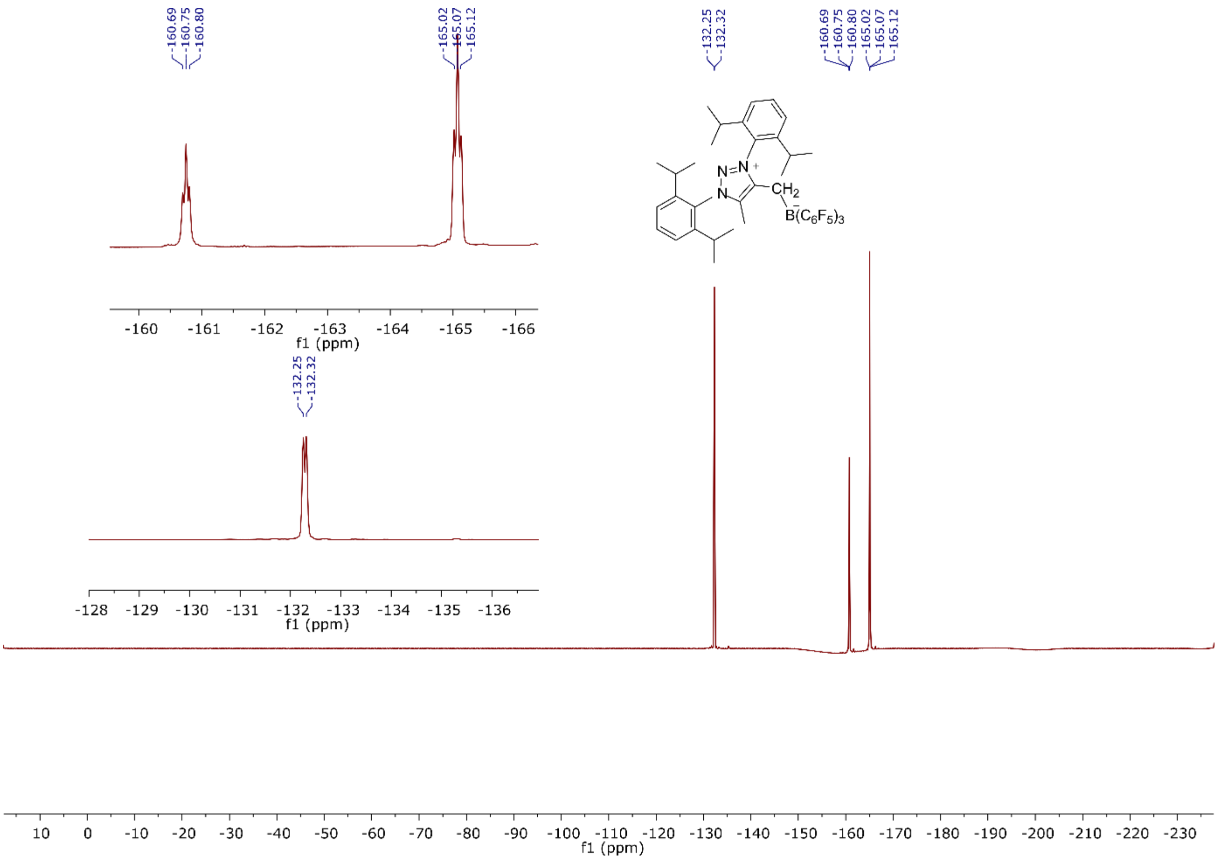

^{19}F NMR (377 MHz, $CDCl_3$, 295K) of **12**

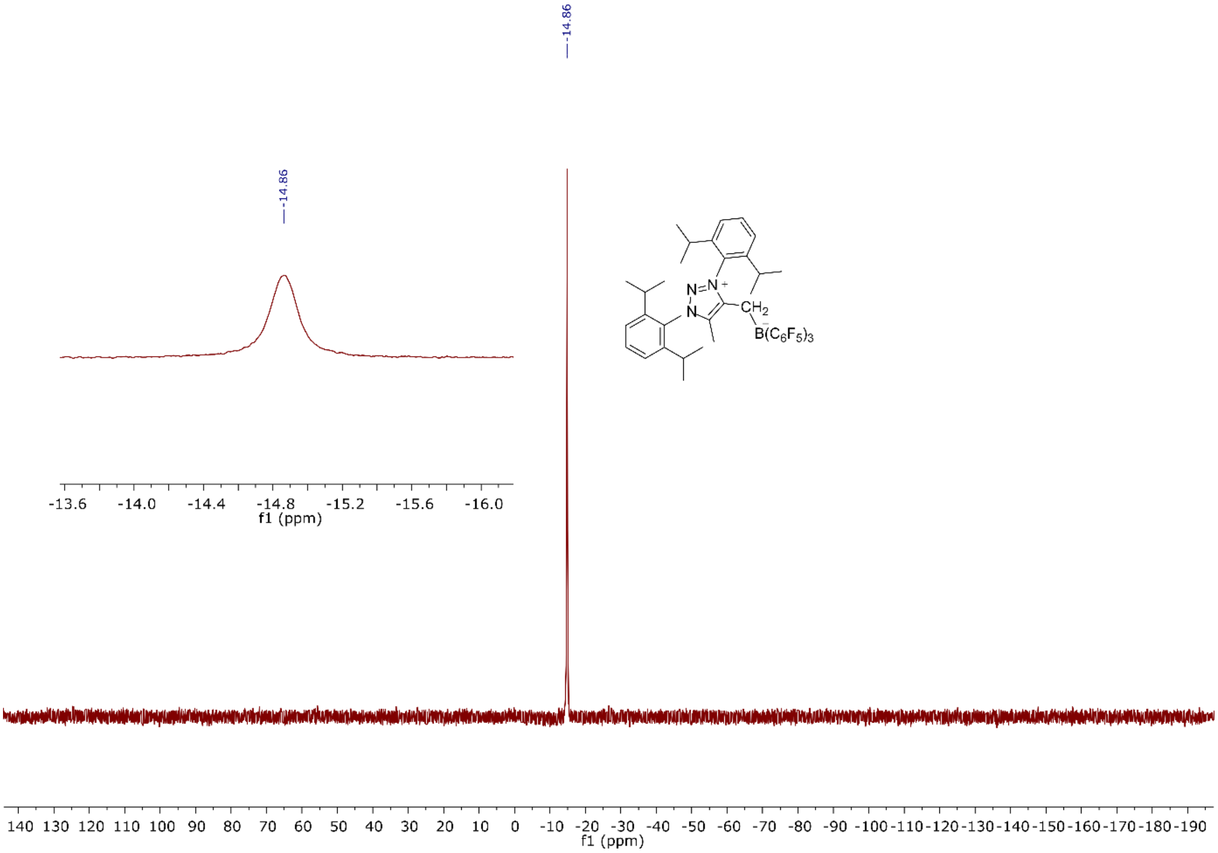

^{11}B NMR (96 MHz, $CDCl_3$, 295K) of **12**

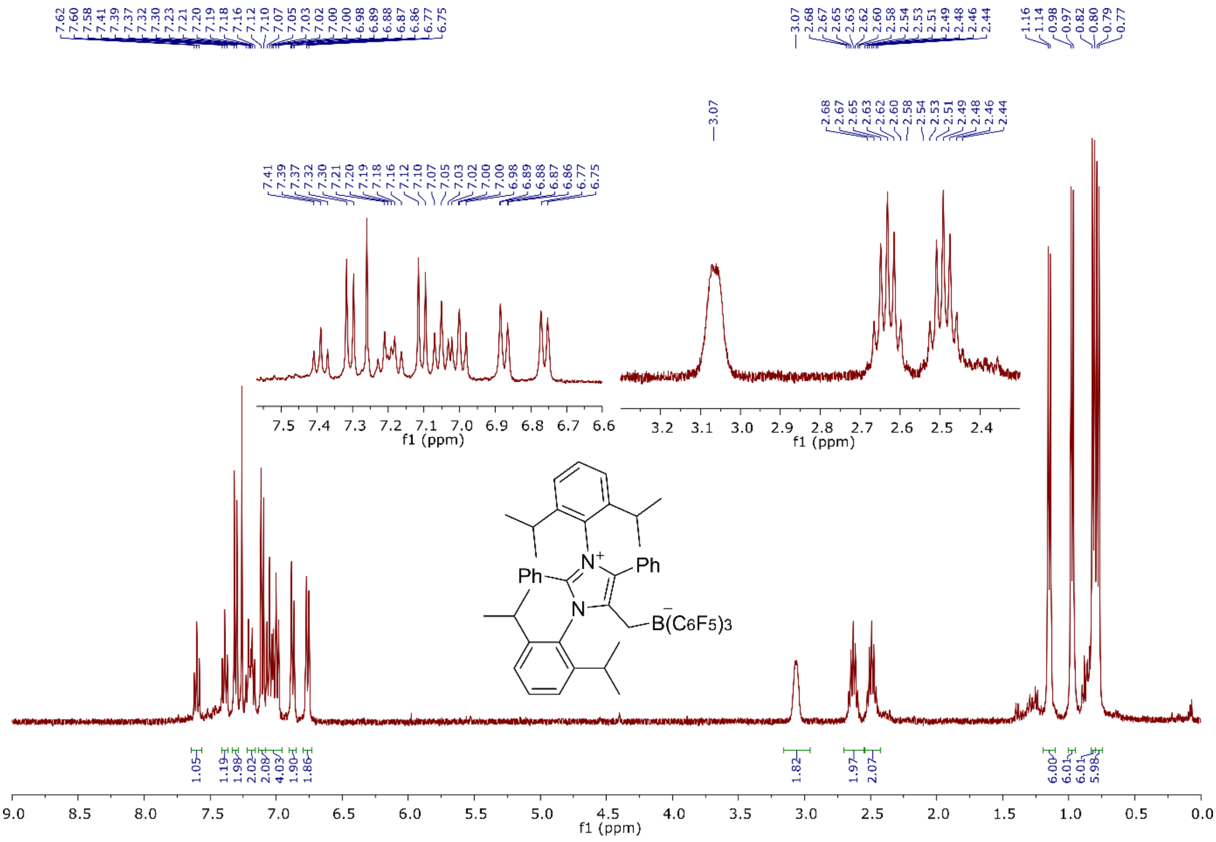

^{1}H NMR (400 MHz, $CDCl_3$, 295K) of **13**

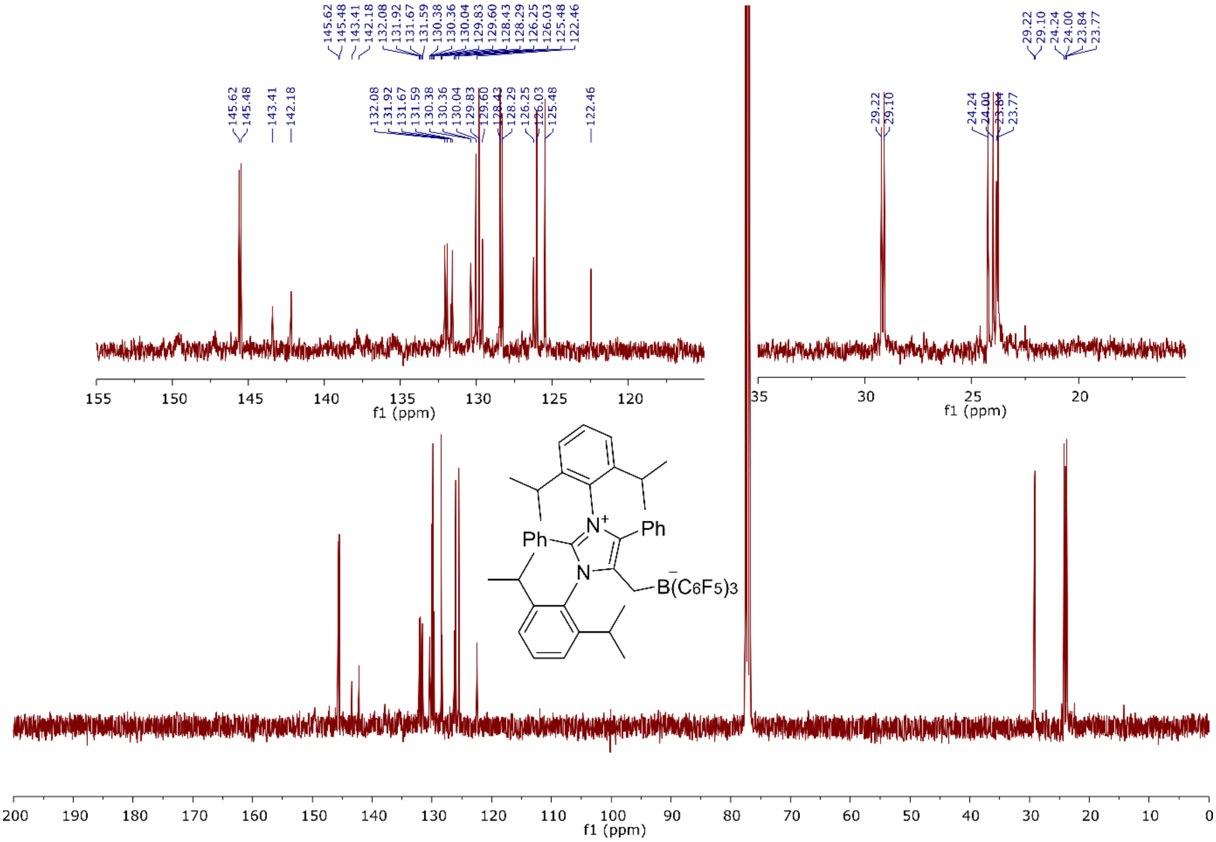

^{13}C {^{1}H} NMR (100 MHz, $CDCl_3$, 295K) of **13**

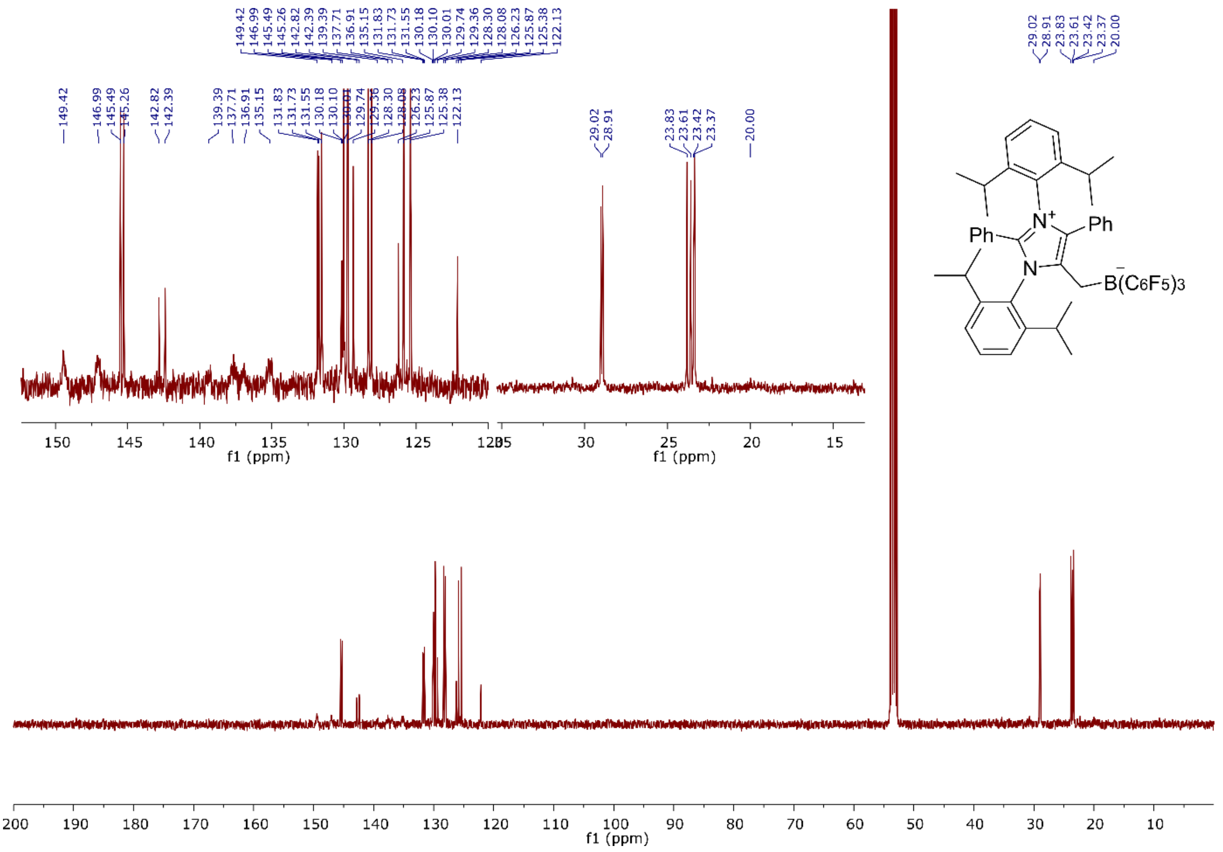

^{13}C {^{1}H} **NMR** (100 MHz, CD_2Cl_2, 295K) of **13**

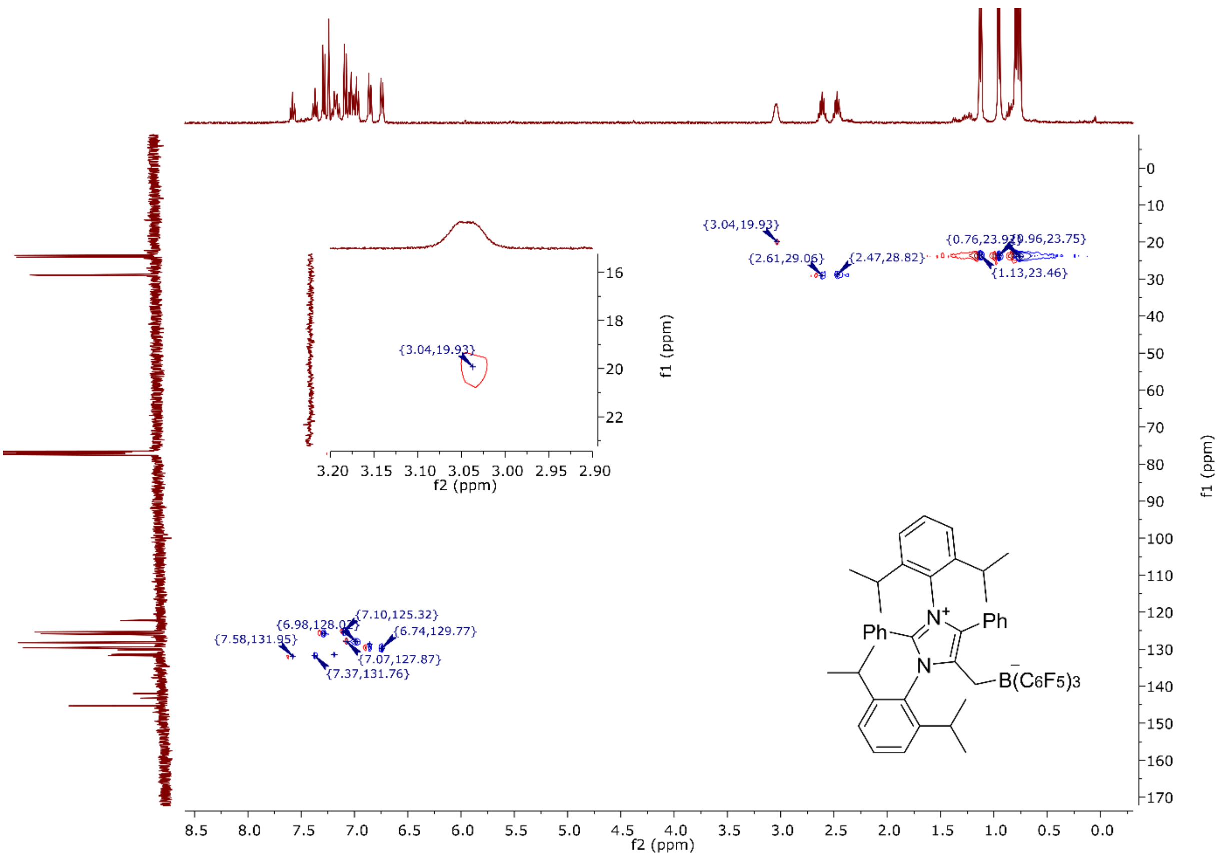

^{1}H/^{13}C **HSQC** (400/100 MHz, $CDCl_3$, 295K) of **13**

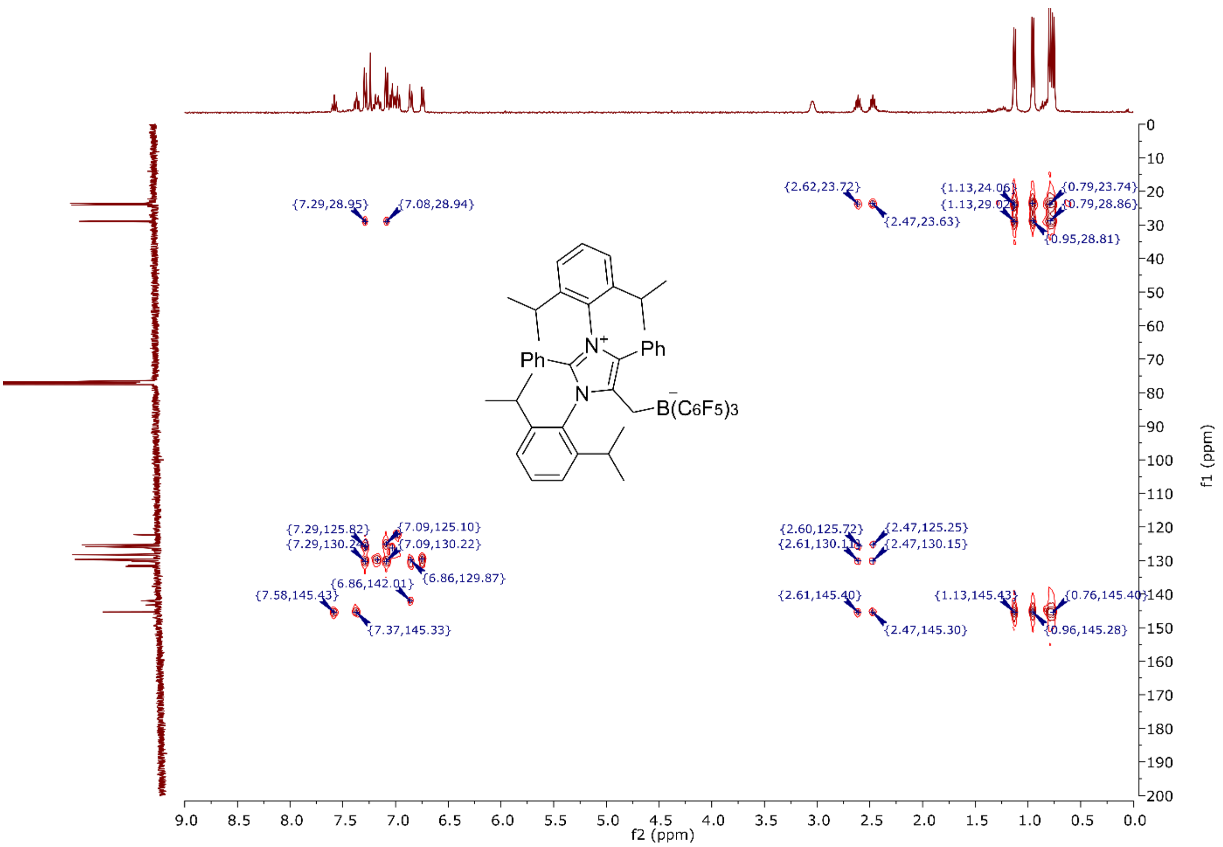

$^{1}H/^{13}C$ **HMBC** (400/100 MHz, $CDCl_3$, 295K) of **13**

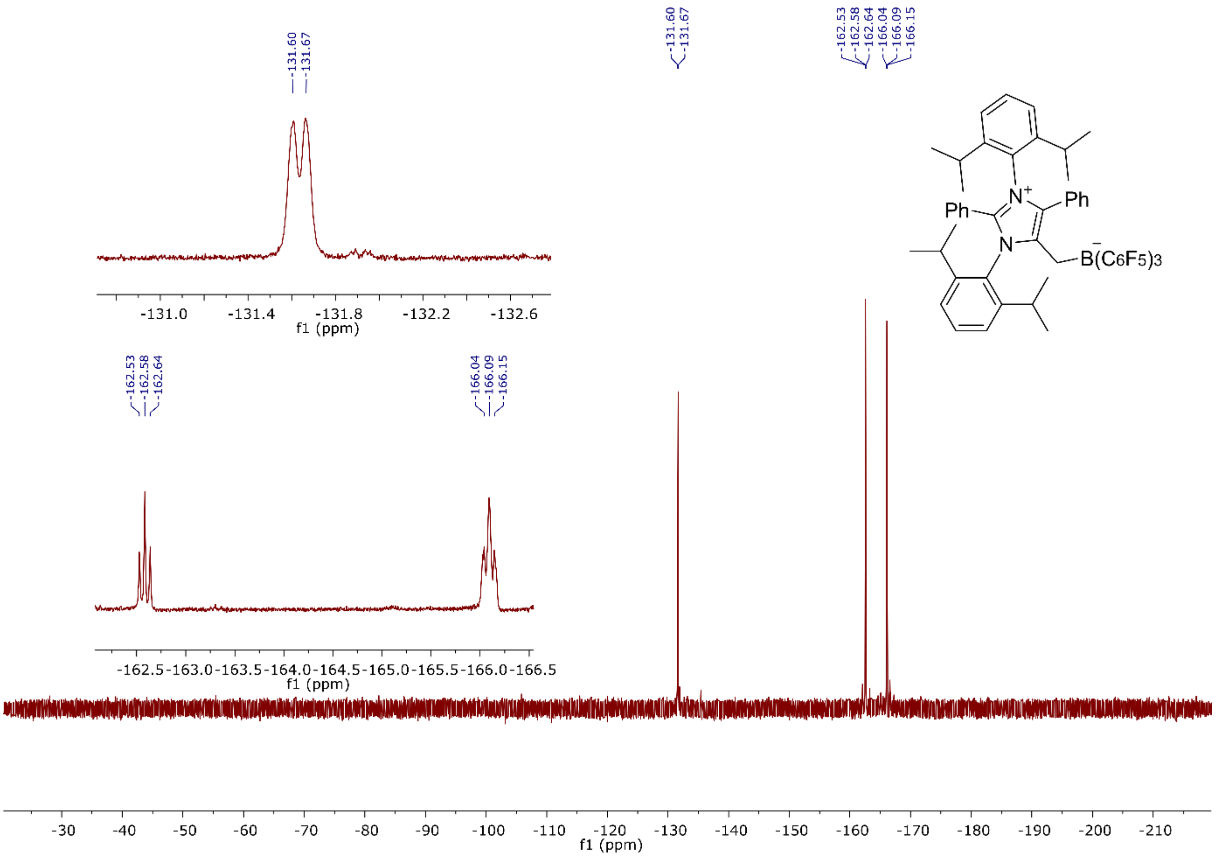

^{19}F **NMR** (377 MHz, $CDCl_3$, 295K) of **13**

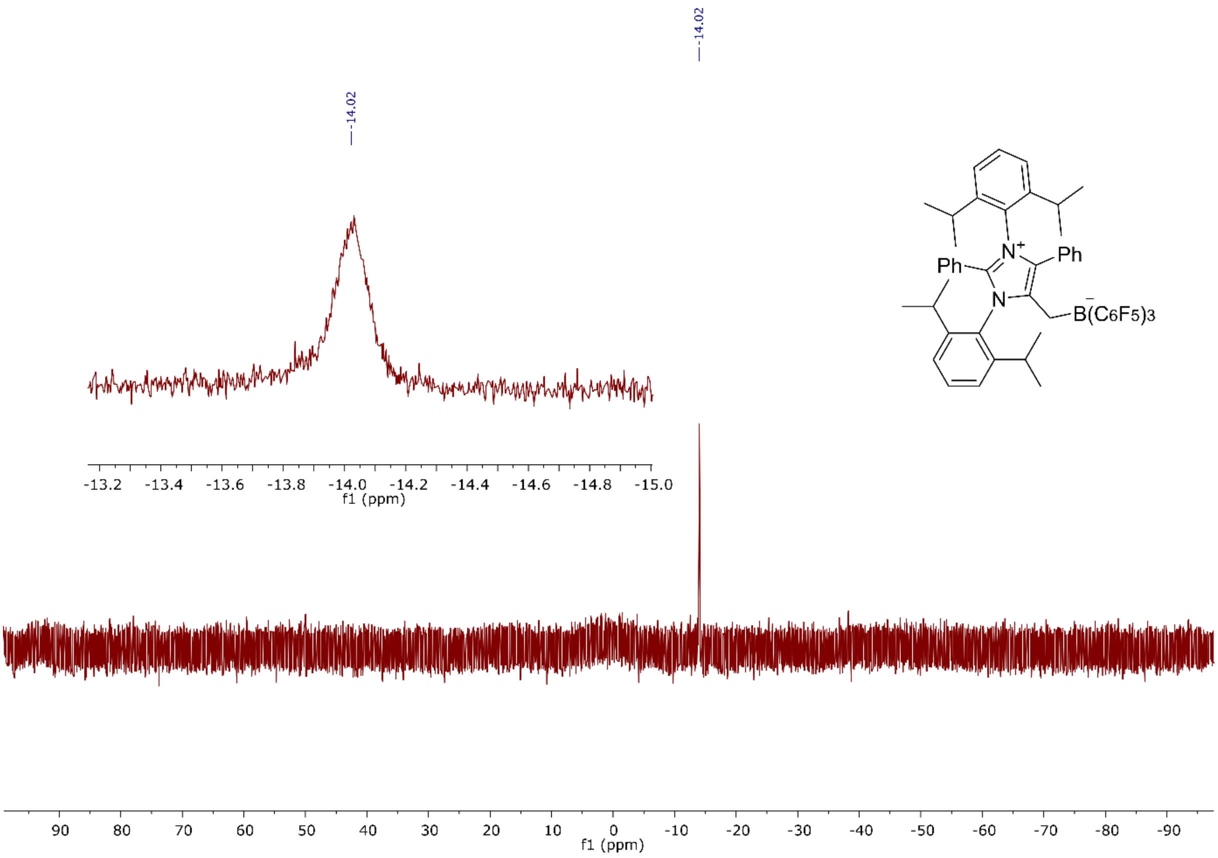

^{11}B NMR (125 MHz, $CDCl_3$, 295K) of **13**

5. Discussion of olefinic ^{1}H NMR signals

<u>Comment on the shape of ^{1}H NMR olefinic signals</u>:

In case of olefins **2**, **4** and **6** we observed for the olefinic signals either sharp or broad peaks based on time and base (KHMDS) present in the NMR tube. We reason that small amounts of H^+ efficiently catalyze the olefinic proton exchange reaction leading to broad signals. In case of broad signals ^{1}H EXSY NMR measurements clearly indicate a proton exchange reaction taking place (Figure S7-S9). In case of **4** ^{1}H EXSY spectra not only show exchange of the olefinic proton signals but also between the $=CH_2$ and $-CH_3$ moieties (Figure S9). In all cases (**2**, **4** and **6**) addition of minor quantities of KHMDS to the NMR sample leads to sharp olefinic signals and stops the exchange reaction based on ^{1}H EXSY NMR experiments. In a sample without additional base, we observe in all cases sharpening of the ^{1}H NMR signals upon cooling (see Figures S3-S6). In order to rule out an influence of potassium ions on the rotation barrier, addition of excess [2,2,2]-cryptand did not lead to sharpening of the NMR signals and had no influence on a sample with small KHMDS quantities. Furthermore, addition of LiHMDS also leads to identical spectra (as with KHMDS) with no difference in chemical shifts. Interestingly, dissolving single crystals of **2** in dry C_6D_6 (distilled over sodium) initially gives sharp ^{1}H NMR olefinic signals, which broaden over time (Figure S10). We assume that a slow minor deprotonation takes part leading to an exchange reaction.

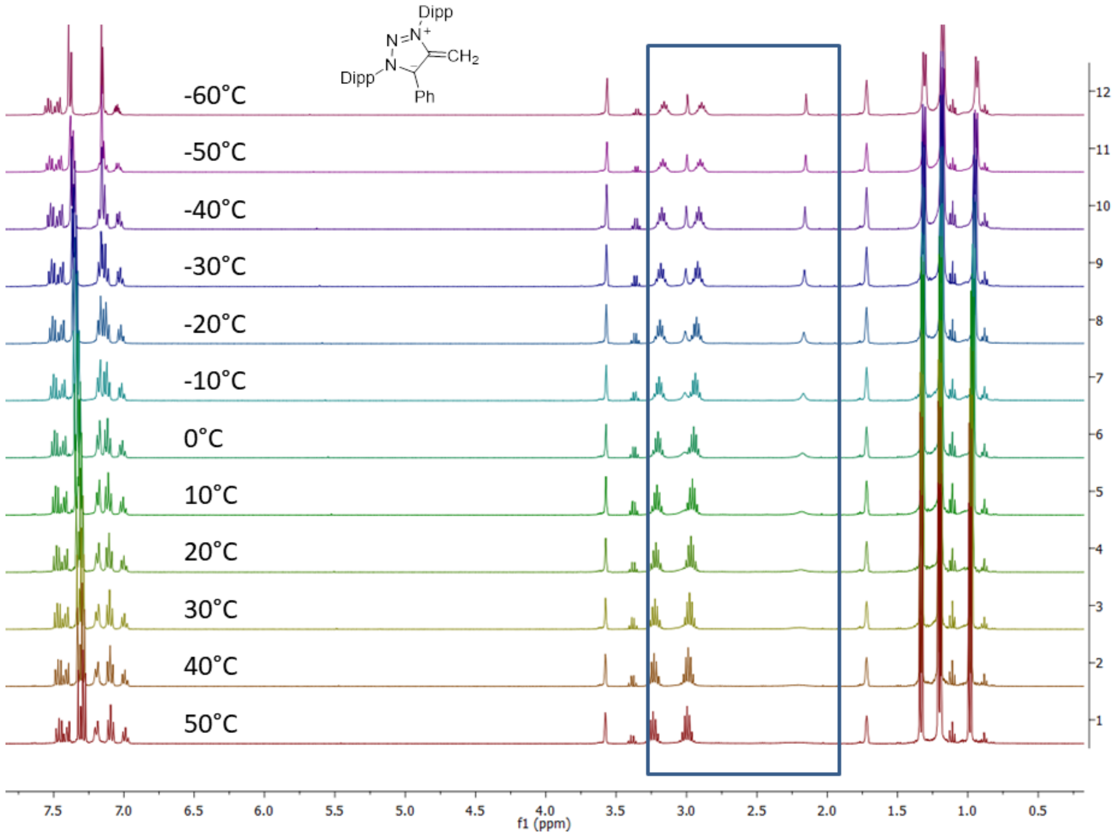

Figure S3. **^{1}H NMR** (400 MHz, d^8-THF, different temperatures) of **2**

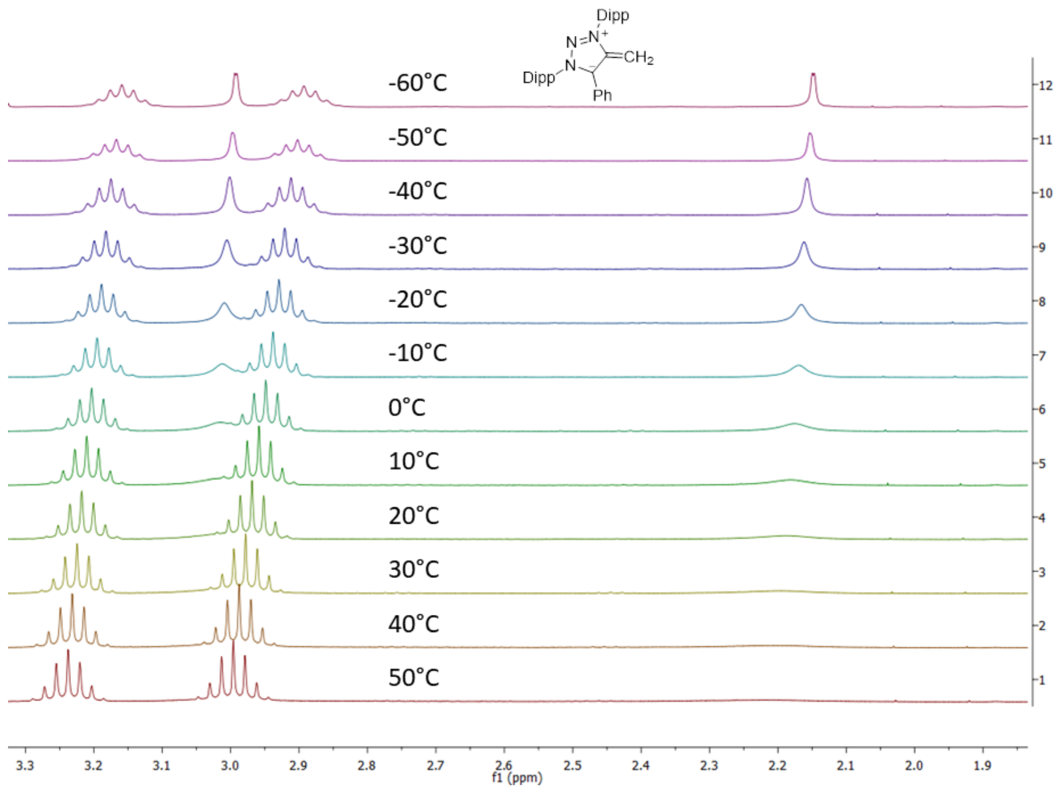

Figure S4. **^{1}H NMR** (400 MHz, d^8-THF, different temperatures within inset) of **2**

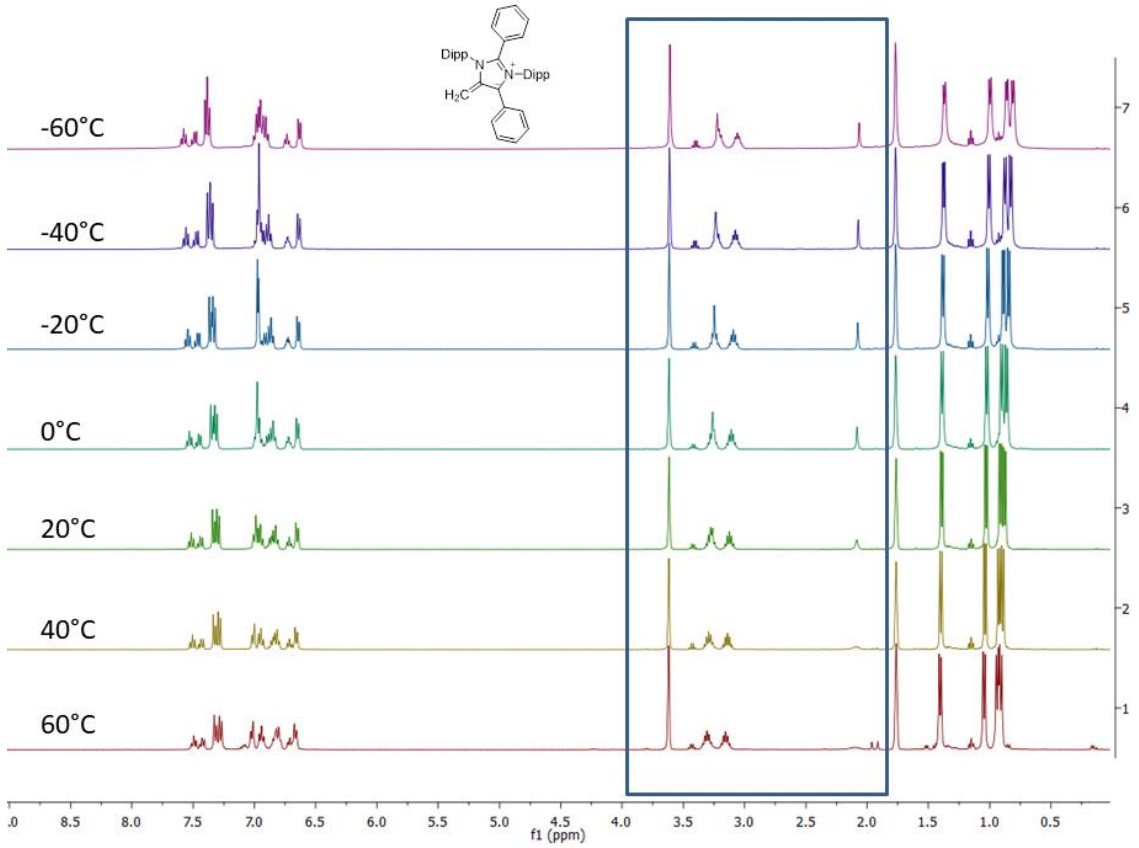

Figure S5. **^{1}H NMR** (400 MHz, d^8-THF, different temperatures) of **6**

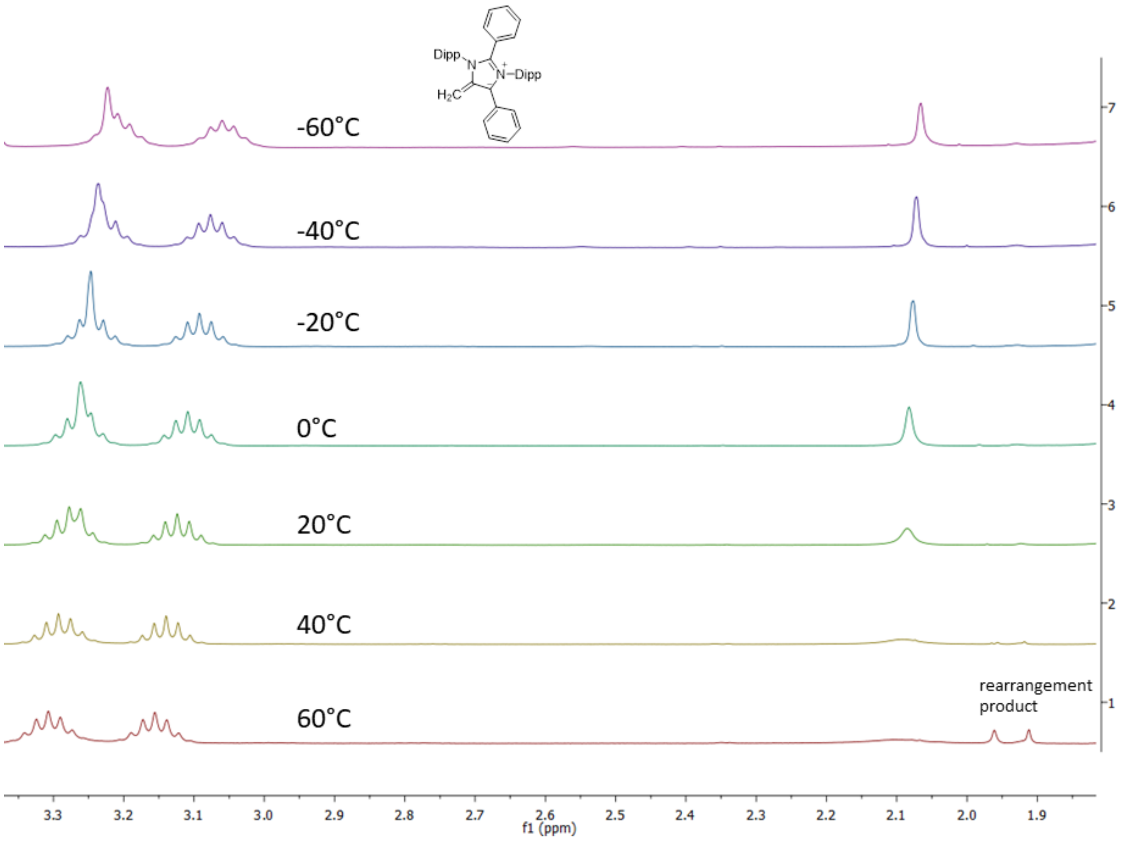

Figure S6. **^{1}H NMR** (400 MHz, d^{8}-THF, different temperatures within inset) of **6**

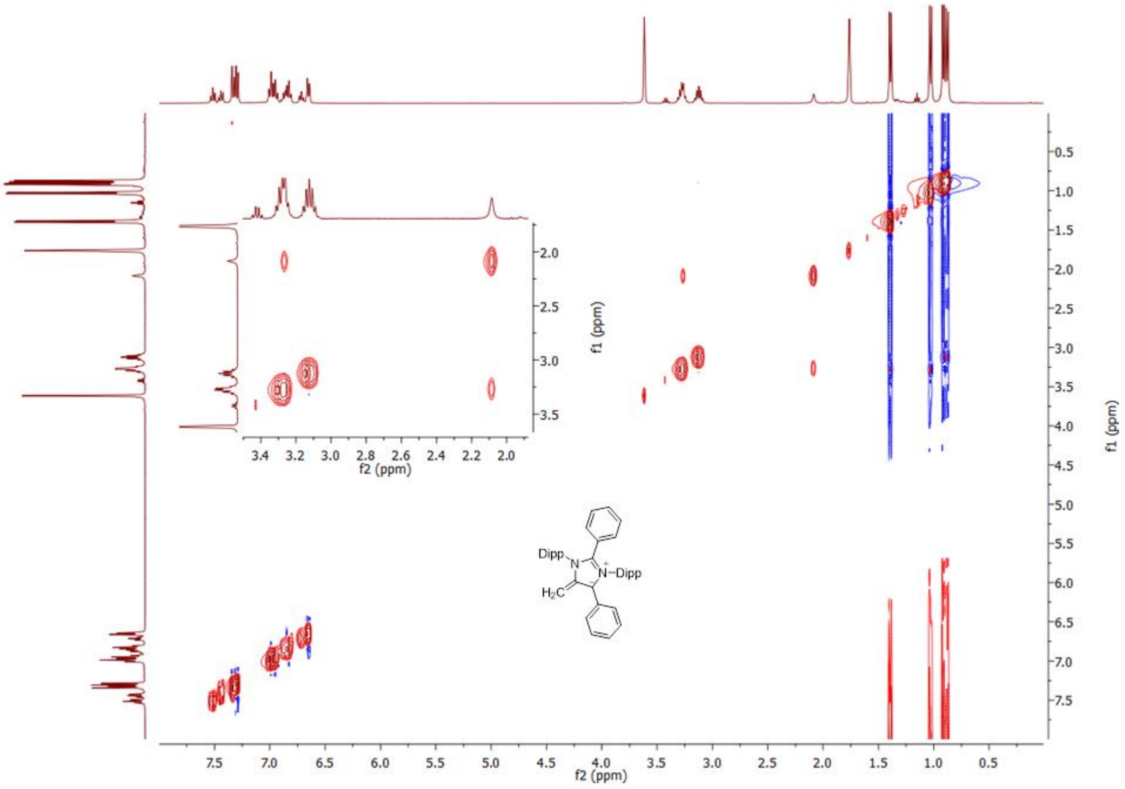

Figure S7. **^{1}H/^{1}H EXSY** (400/400 MHz, d^{8}-THF, 293K, d8 = 0.02 s) of **6**

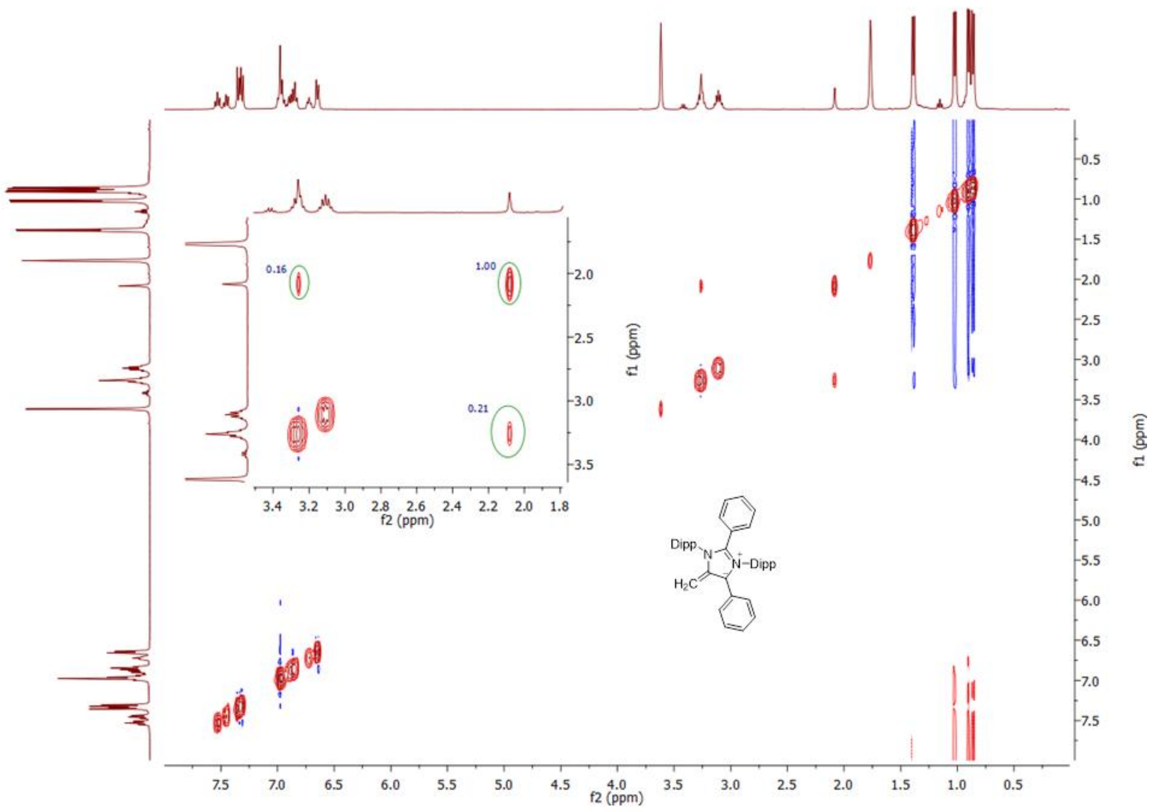

Figure S8. ^{1}H/^{1}H EXSY (400/400 MHz, d^8-THF, 273K, d8 = 0.1 s) of **6**

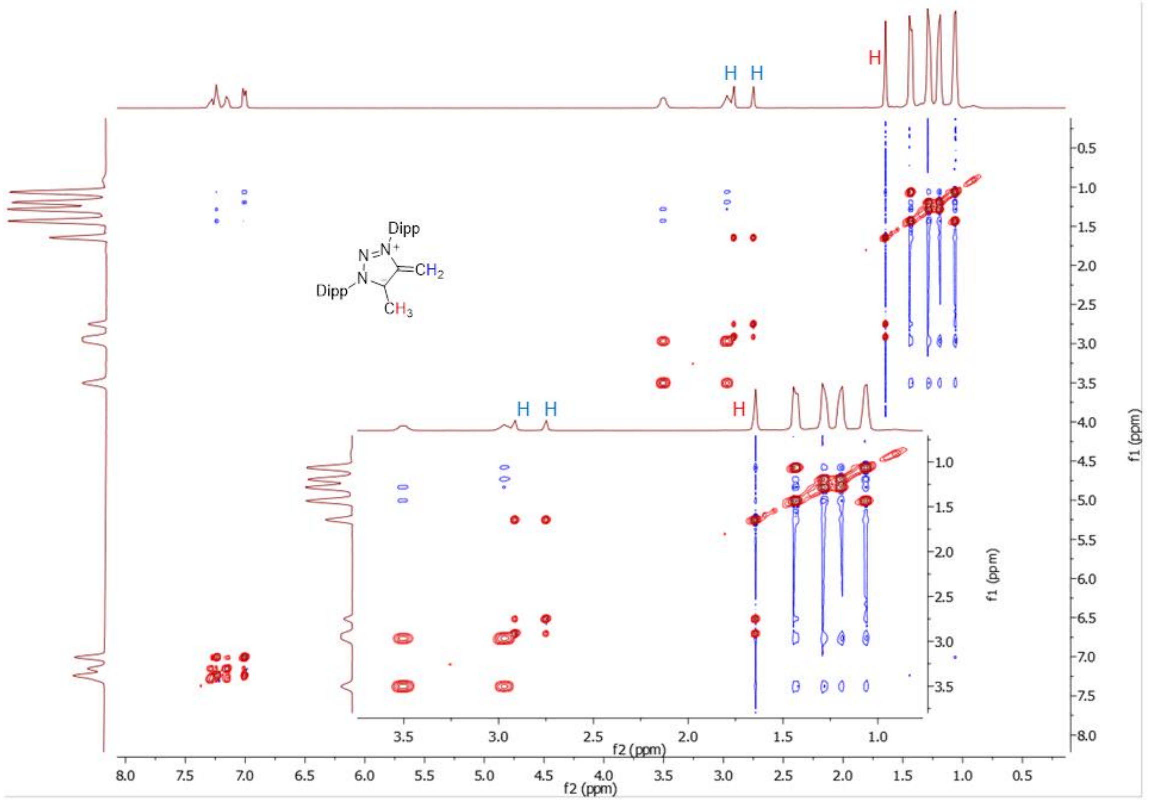

Figure S9. ^{1}H/^{1}H EXSY (400/400 MHz, d^8-THF, 298K) of **4**

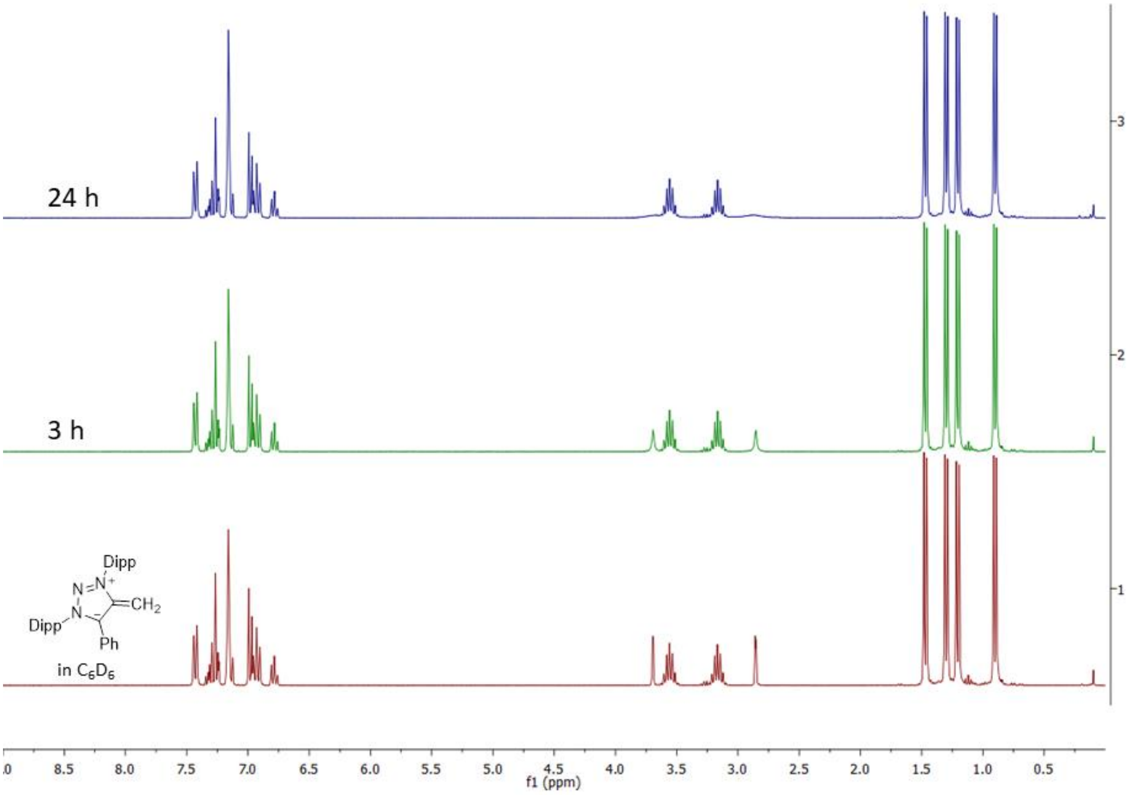

Figure S10. Time-dependent measurement of **2** in C_6D_6.

6. Rearrangement of mNHO 6

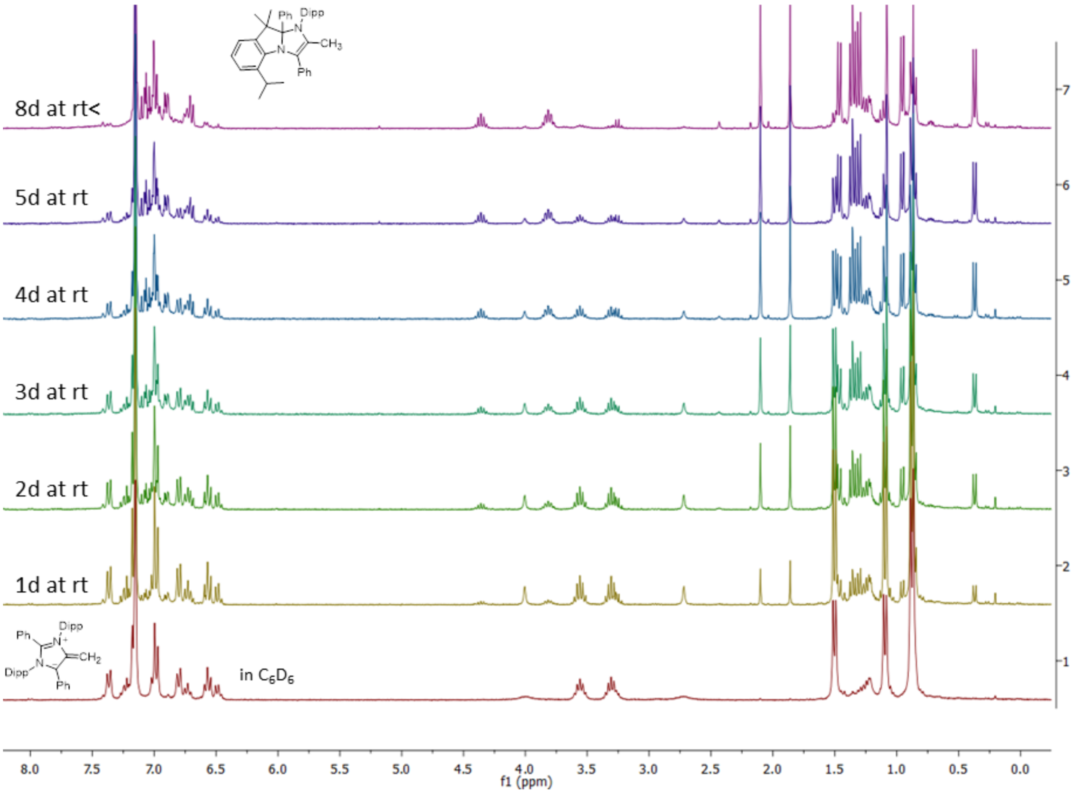

Figure S11. Rearrangement of **6** at room temperature to **7** in C_6D_6.

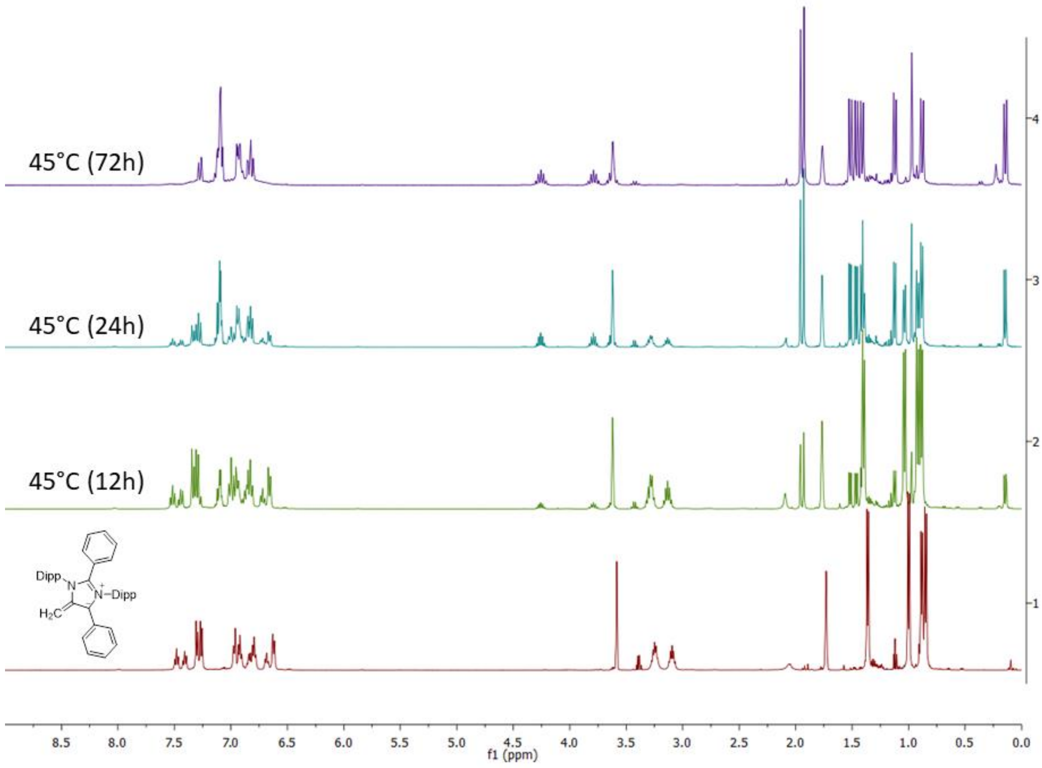

Figure S12. Thermal rearrangement of **6** over time to **7** at 45 °C in d^8-THF.

7. Competition experiments

In order to determine the basicity trends competition experiments were performed (Figure S13). In each case both the forward (*i* and *iii*) and backward (*ii* and *iv*) reaction were performed to guarantee that a thermodynamic and not kinetic regime is present. Indeed, forward and backward reaction gave in each two cases (*i*/*ii*) and (*iii*/*iv*) the same result (Figures S14-S17). In order to rule out an influence of the counter-anion, in each case the corresponding PF_6^- salts were prepared by salt metathesis. d^8-THF was chosen as solvent to guarantee the best solubility of the salts.

General procedures:

In a J-Young NMR tube one of the reagents (typically 10-30 mg) was dissolved in d^8-THF and a ^{1}H NMR measured. To the tube was added the other reagent (1 equivalent), vigorously shaken and an NMR spectrum measured after ca. 20 min. Later measurements had no influence on the spectra.

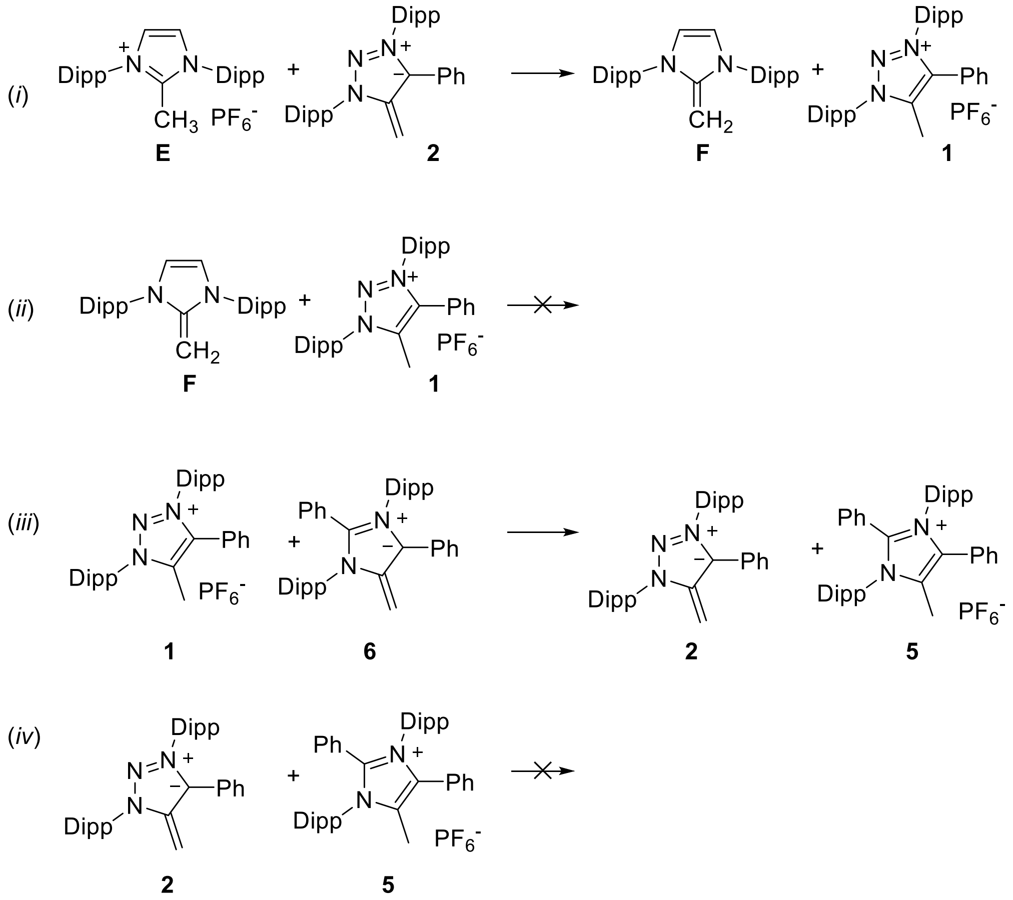

Figure S13. Competition experiments performed (see Figures S14-S17 for results).

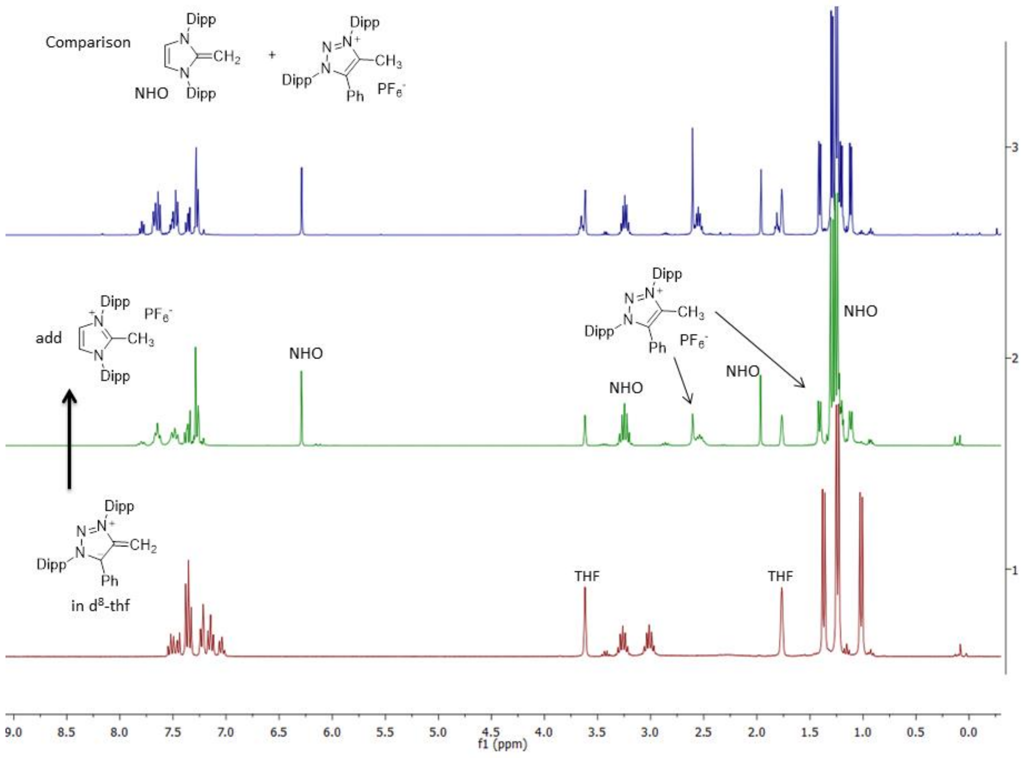

Figure S14. Deprotonation experiment of (NHC)-CH_3 PF_6^- (**E**) with mNHO (**2**).

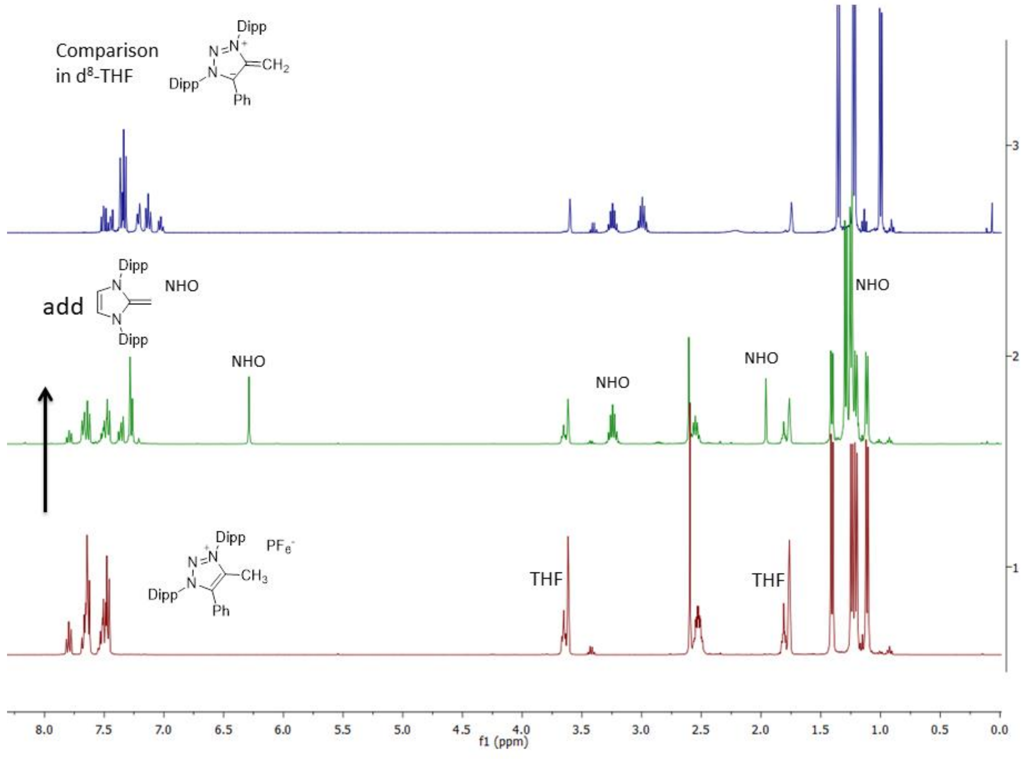

Figure S15. Deprotonation experiment of MIC-CH_3 PF_6^- (**1**) with NHO (**F**).

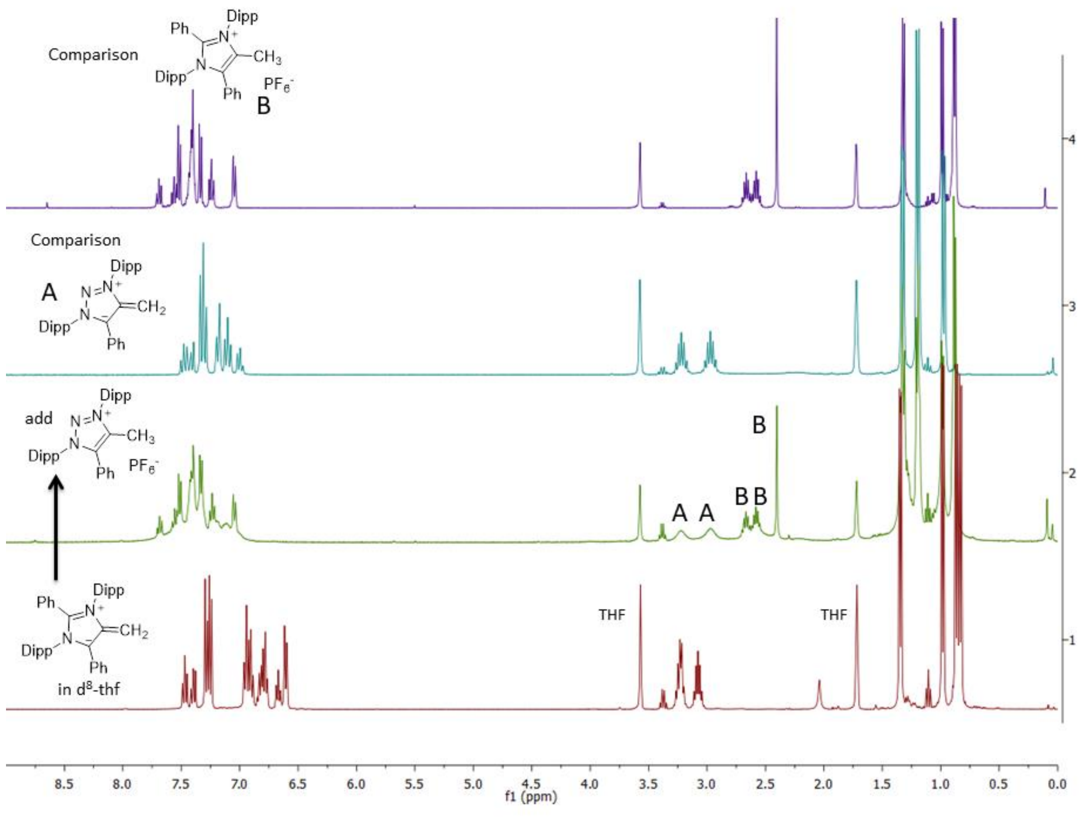

Figure S16. Deprotonation experiment of mNHO (**6**) with (MIC)-CH_3 PF_6^- (**1**).

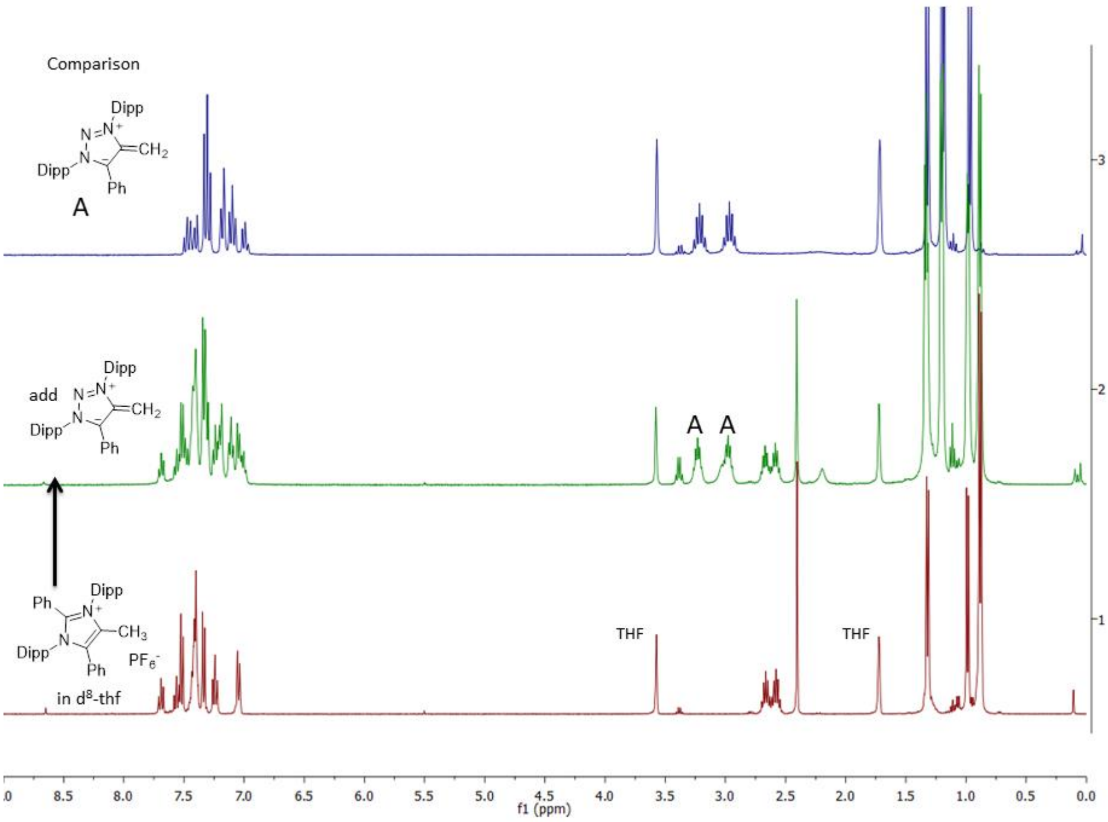

Figure S17. Deprotonation experiment of (aNHC)-CH_3 PF_6^- (**5**) with mNHO (**2**).

Rhodium competition experiments

Procedure for *i*: (mNHO)RhCl(CO)$_2$ **8** (10 mg, 15 µmol) was dissolved in d_8-THF (0.6 mL) in a J-Young NMR and a ^{1}H NMR measured. To this solution IPrCH$_2$ (6 mg, 15 µmol) was added and a ^{1}H-NMR measured after ca. 30 min to show no reaction (Figure S19). No spectral changed could be detected upon standing for 3 days at room temperature.

Procedure for *ii*: (IPrCH$_2$)RhCl(CO)$_2$ (11.3 mg, 19 µmol) was dissolved in d_8-THF (0.6 mL) in a J-Young NMR. A ^{1}H NMR of the slightly yellow solution was taken and mNHO **2** (9.0 mg, 19 µmol) added. The initial purple color fades over ca. 5 min. A ^{1}H-NMR was taken after ca. 20 min. The ^{1}H NMR spectrum shows clean liberation of the IPrCH$_2$ ligand and formation of **8** (Figure S20).

Procedure for *iii*: (mNHO)RhCl(CO)$_2$ **8** (10 mg, 15 µmol) was dissolved in d_8-THF (0.6 mL) in a J-Young NMR and a ^{1}H NMR measured. To this solution IPr carbene (5.8 mg, 15 µmol) was added and a ^{1}H NMR measured after ca. 30 min. The ^{1}H NMR spectrum shows formation of IPrRhCl(CO)$_2$ (Figure S21).

Procedure for *iv*: IPrRhCl(CO)$_2$ (9 mg, 15 µmol) was dissolved in d_8-THF (0.6 mL) in a J-Young NMR and a ^{1}H NMR measured. To this solution mNHO **2** (7 mg, 15 µmol) was added and a ^{1}H-NMR measured after ca. 30 min. No spectral change of IPrRhCl(CO)$_2$ was detected (Figure S22).

Procedure for *v*: (mNHO)RhCl(CO)$_2$ **8** (10 mg, 15 µmol) was dissolved in d_8-THF (0.6 mL) in a J-Young NMR and a ^{1}H NMR measured. To this solution mNHO **6** (8 mg, 15 µmol) was added. The solution instantaneously changes color from green to violet. A ^{1}H-NMR was taken after ca. 20 min. The ^{1}H NMR spectrum shows clean liberation of the mNHO ligand **2** and formation of **10** (Figure S23).

Procedure for *vi*: (mNHO)RhCl(CO)$_2$ **10** (10 mg, 13 µmol) was dissolved in d_8-THF (0.6 mL) in a J-Young NMR and a ^{1}H NMR measured. To this solution IPr carbene (5.2 mg, 13 µmol) was added. The solution instantaneously changes color to intense green. A ^{1}H-NMR was taken after ca. 20 min. The ^{1}H NMR spectrum shows formation of the IPrRhCl(CO)$_2$ complex.

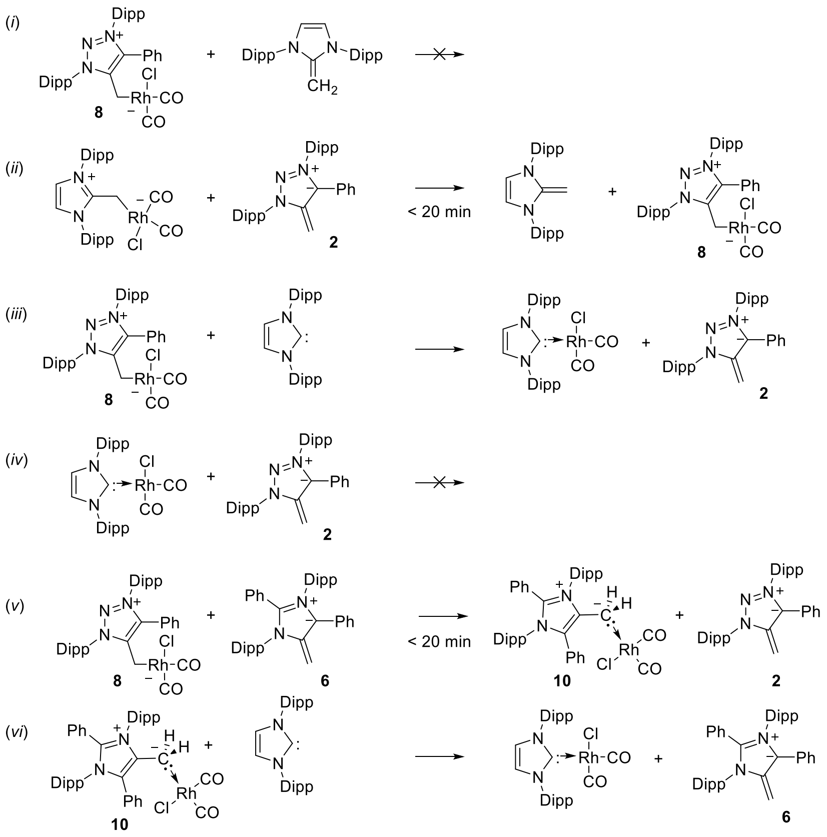

Figure S18. Rhodium competition experiments (see Figures S19-S24 for results).

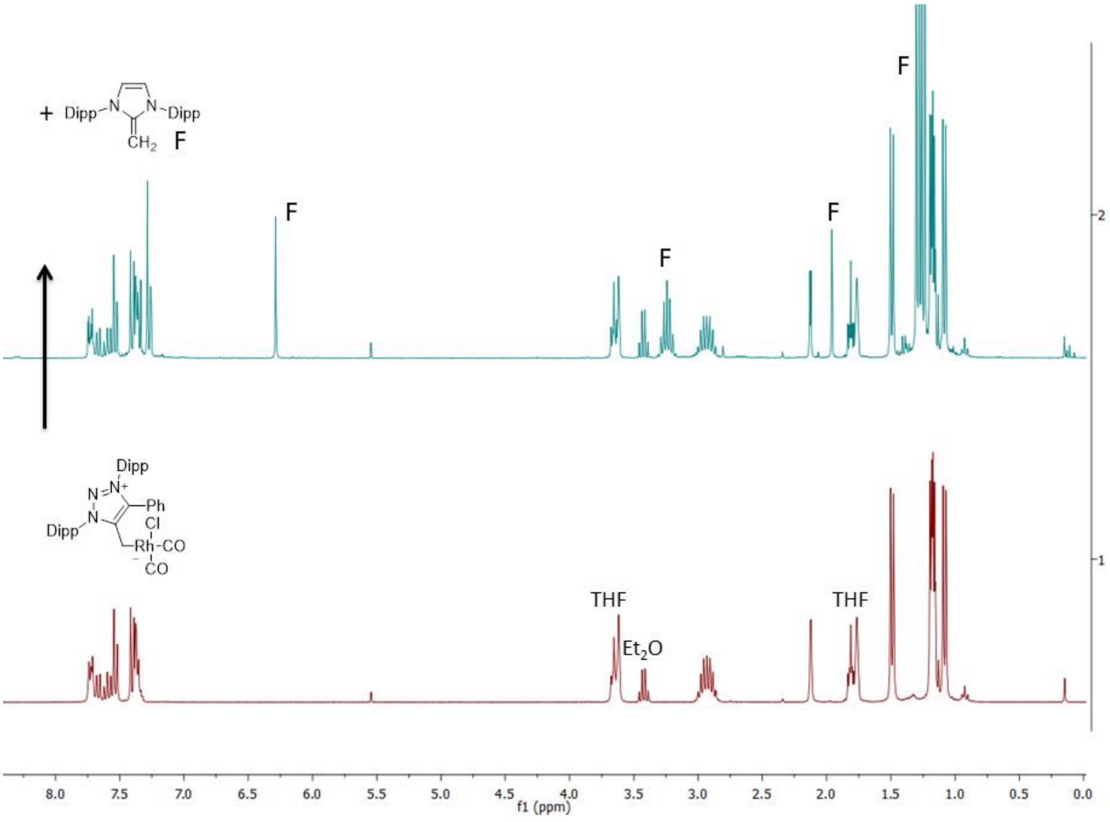

Figure S19. Addition of NHO (**F**) to (mNHO)RhCl(CO)$_2$ **8** in d$_8$-THF.

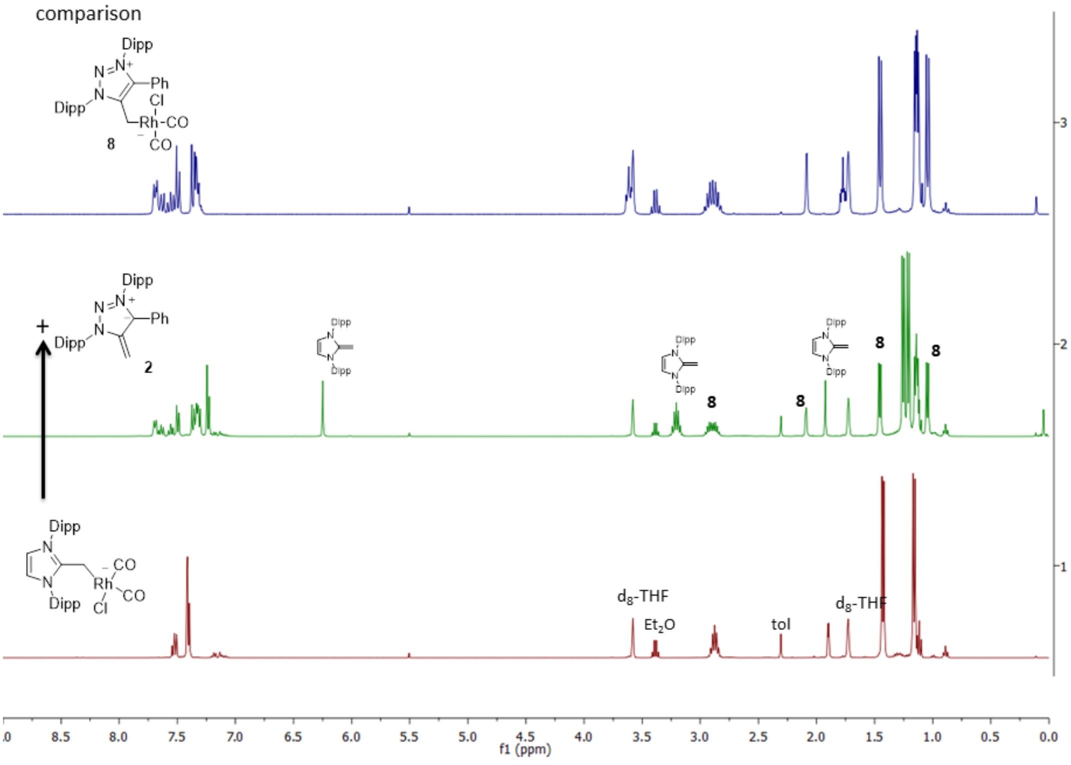

Figure S20. Addition of mNHO (**2**) to (IPrCH$_2$)RhCl(CO)$_2$ **8** in d$_8$-THF.

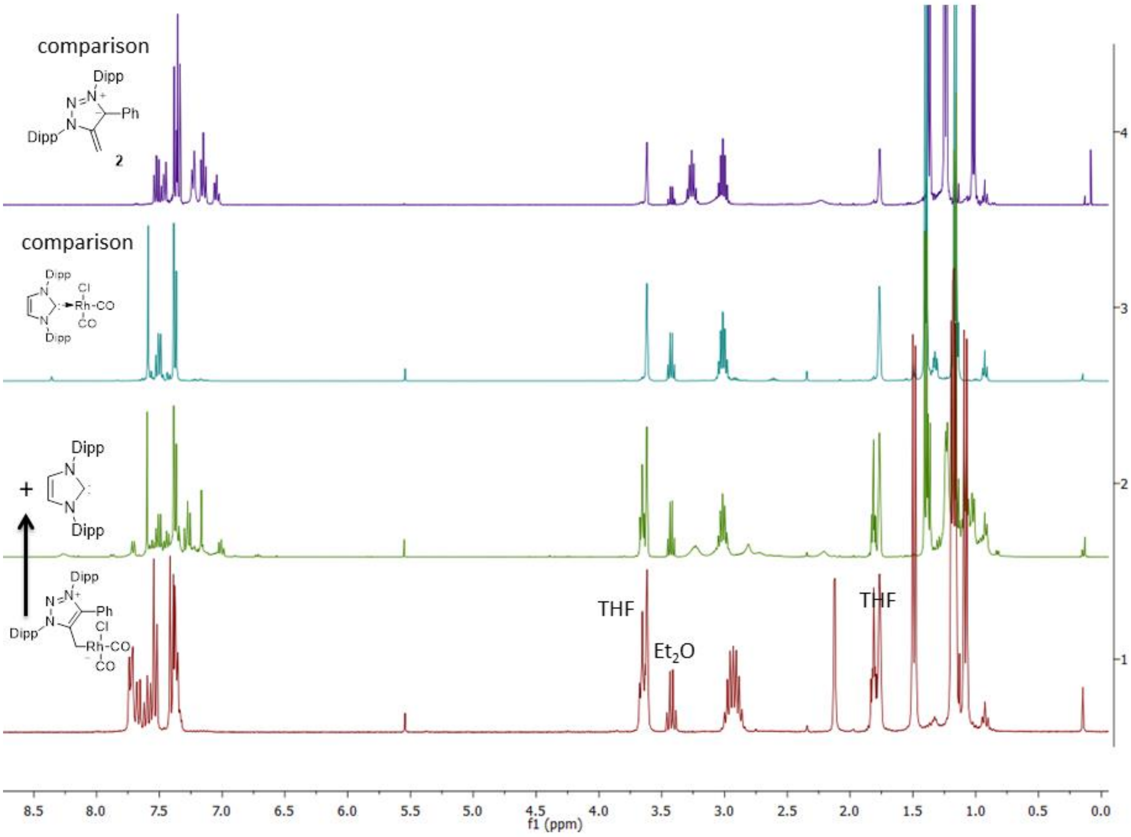

Figure S21. Addition of IPr carbene to (mNHO)RhCl$(CO)_2$ **8** in d_8-THF.

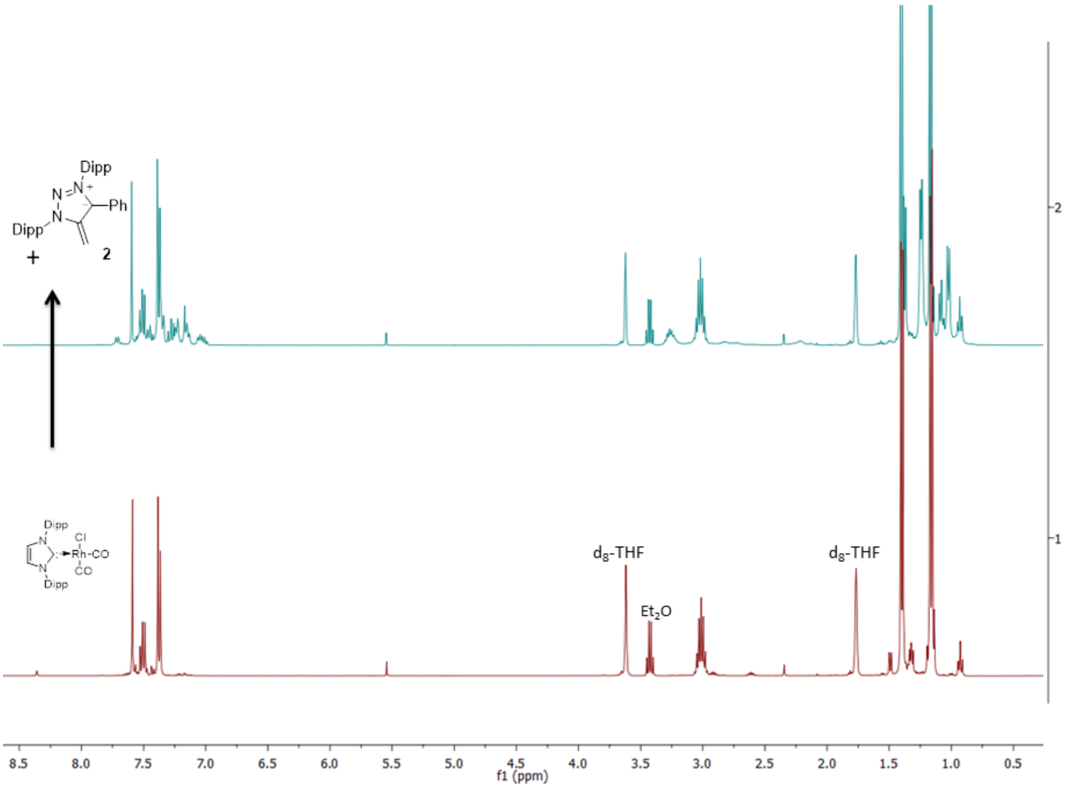

Figure S22. Addition of mNHO **2** to IPrRhCl$(CO)_2$ in d_8-THF.

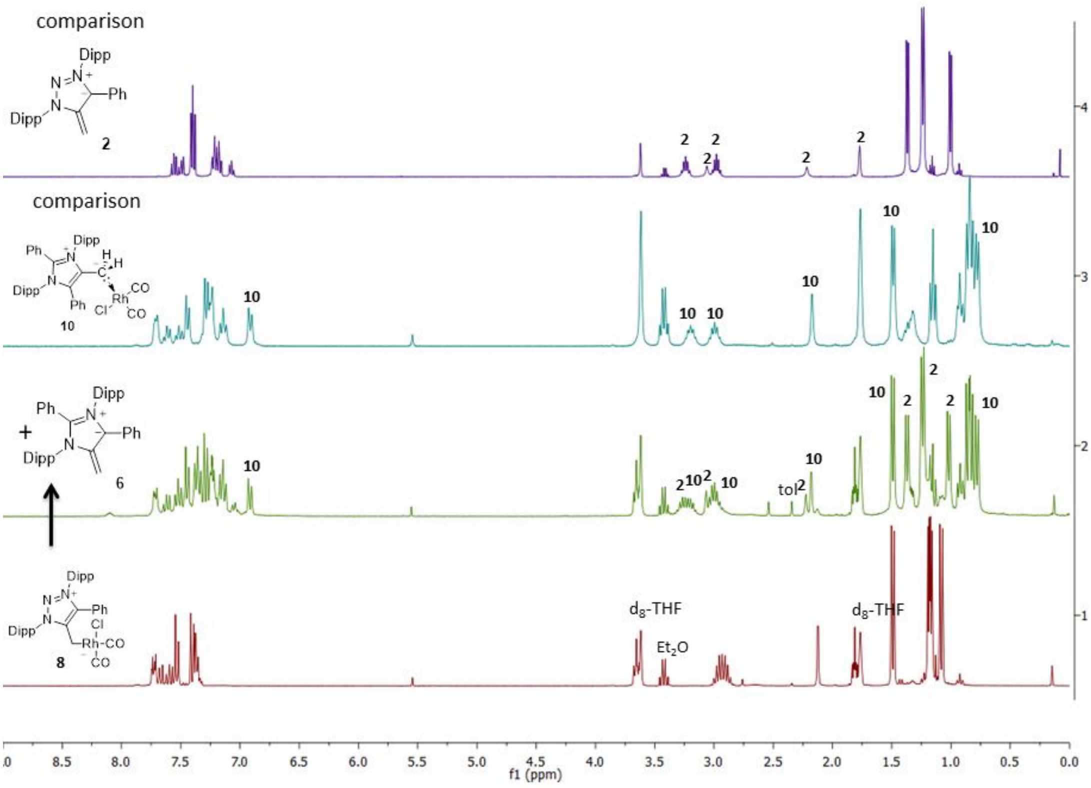

Figure S23. Addition of mNHO **6** to (mNHO)RhCl$(CO)_2$ **8** in d_8-THF.

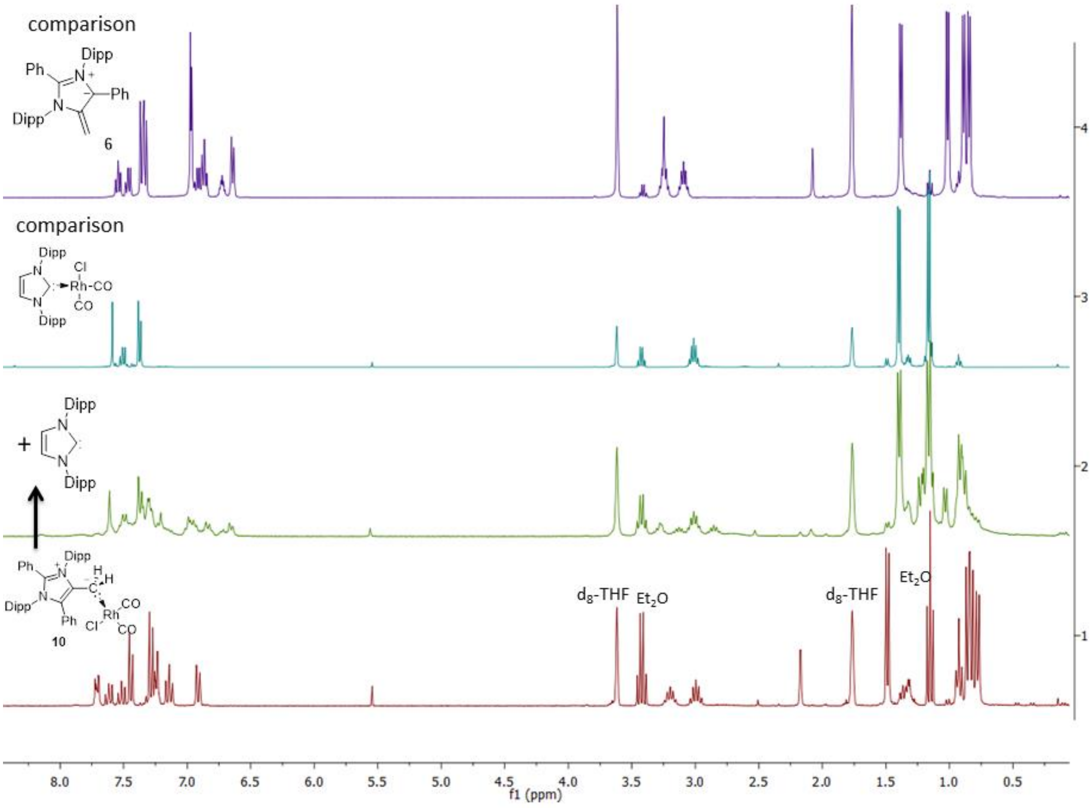

Figure S24. Addition of IPr carbene to (mNHO)RhCl$(CO)_2$ **10** in d_8-THF.

8. X-ray characterization data

General part

Single crystal X-ray diffraction analysis

Data collection was done on a *Bruker D8 Venture* four-circle-diffractometer from *Bruker AXS GmbH*; used detector: *Photon II* from *Bruker AXS GmbH*; used X-ray sources: microfocus *IµS* Cu/Mo from *Incoatec GmbH* with mirror optics *HELIOS* and single-hole collimator from *Bruker AXS GmbH*.

Used programs: *APEX3 Suite* (v2017.3-0) and therein integrated programs *SAINT* (Integration) und *SADABS* (Absorption correction) from *Bruker AXS GmbH*; structure solution was done with *SHELXT*, refinement with *SHELXS*;[17] OLEX was used for data finalization.[18]

Special Utilities: *SMZ1270* stereomicroscope from *Nikon Metrology GmbH* was used for sample preparation; crystals were mounted on *MicroMounts* or *MicroLoops* from *MiTeGen*; for sensitive samples the *X-TEMP 2 System* was used for picking of crystals;[19] crystals were cooled to given temperature with *Cryostream 800* from *Oxford Cryosystems.*

X-ray structures

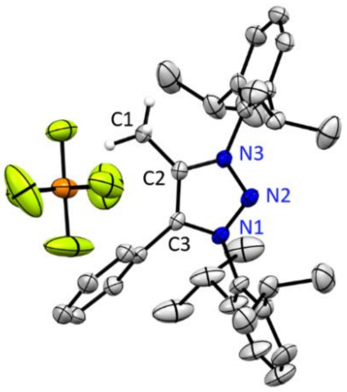

Figure S25. X-ray solid-state structure of **1** (CCDC: 1965566). Ellipsoids shown with 50% probability. Two molecules are positioned in the asymmetric unit cell. Solvent molecule (CH_2Cl_2) and hydrogen atoms (except $-CH_3$) omitted for clarity. Selected bond parameters in [Å] and [°]: first molecule: C1-C2 1.478(5), C2-C3 1.373(5), C3-N1 1.371(5), N1-N2 1.320(5), N2-N3 1.323(4), N3-C2 1.363(5); second molecule: C1-C2 1.474(5), C2-C3 1.384(5), C3-N1 1.371(5), N1-N2 1.319(5), N2-N3 1.324(4), N3-C2 1.359(5).

Table S1. Crystal data and structure refinement for **1**.

Empirical formula	$C_{67}H_{86}Cl_2F_{12}N_6P_2$
Formula weight	1336.25
Temperature/K	100.0
Crystal system	orthorhombic
Space group	$P2_12_12_1$
a/Å	11.982(6)
b/Å	12.227(5)
c/Å	47.340(19)
α/°	90
β/°	90
γ/°	90
Volume/Å^3	6935(5)
Z	4
ρ_{calc}g/cm^3	1.280
μ/mm^{-1}	0.215
F(000)	2808.0
Crystal size/mm^3	0.432 × 0.369 × 0.018
Radiation	MoKα (λ = 0.71073)
2Θ range for data collection/°	4.76 to 52.866
Index ranges	$-14 \leq h \leq 14$, $-15 \leq k \leq 14$, $-50 \leq l \leq 59$
Reflections collected	56619
Independent reflections	14209 [R_{int} = 0.0386, R_{sigma} = 0.0406]
Data/restraints/parameters	14209/139/914
Goodness-of-fit on F^2	1.035
Final R indexes [I>=2σ (I)]	R_1 = 0.0583, wR_2 = 0.1407
Final R indexes [all data]	R_1 = 0.0727, wR_2 = 0.1496
Largest diff. peak/hole / e Å^{-3}	0.45/-0.41
Flack parameter	-0.01(2)

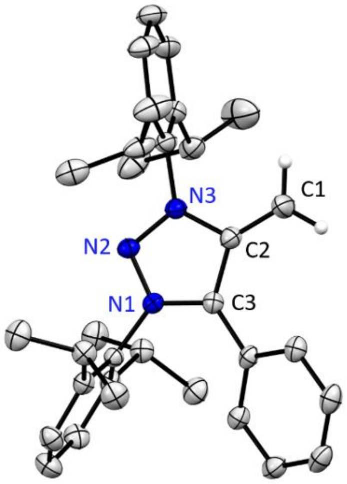

Figure S26. X-ray solid-state structure of **2** (CCDC: 1965562). Ellipsoids shown with 50% probability. Hydrogen atoms (except $=CH_2$) omitted for clarity. Selected bond parameters in [Å] and [°]: C1-C2 1.361(1), C2-C3: 1.435(1), C3-N1: 1.356(1), N1-N2: 1.324(1), N2-N3: 1.369(1), N3-C2: 1.399(1).

Table S2. Crystal data and structure refinement for **2**.

Empirical formula	$C_{33}H_{41}N_3$
Formula weight	479.69
Temperature/K	100.0
Crystal system	triclinic
Space group	P-1
a/Å	9.703(2)
b/Å	10.420(3)
c/Å	15.402(4)
α/°	106.804(7)
β/°	94.452(7)
γ/°	104.890(8)
Volume/Å^3	1421.1(6)
Z	2
ρ_{calc}g/cm^3	1.121
μ/mm^{-1}	0.065
F(000)	520.0
Crystal size/mm^3	0.234 × 0.134 × 0.076
Radiation	MoKα (λ = 0.71073)
2Θ range for data collection/°	4.27 to 59.272
Index ranges	$-13 \leq h \leq 13$, $-14 \leq k \leq 14$, $-21 \leq l \leq 21$
Reflections collected	54076
Independent reflections	7985 [R_{int} = 0.0382, R_{sigma} = 0.0234]
Data/restraints/parameters	7985/0/341
Goodness-of-fit on F^2	1.026
Final R indexes [I>=2σ (I)]	R_1 = 0.0422, wR_2 = 0.1028
Final R indexes [all data]	R_1 = 0.0505, wR_2 = 0.1093
Largest diff. peak/hole / e Å^{-3}	0.33/-0.20

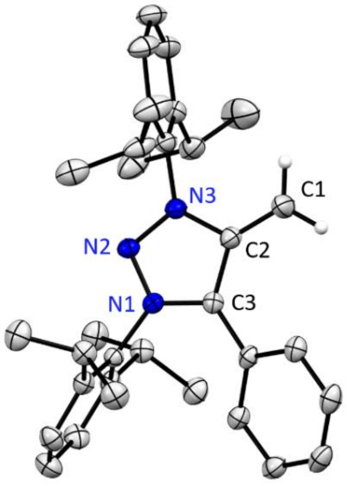

Figure S27. X-ray solid-state structure of **2** (CCDC: 1965570). Second polymorph. Ellipsoids shown with 50% probability. Hydrogen atoms (except $=CH_2$) omitted for clarity. Selected bond parameters in [Å] and [°]: C1-C2 1.351(2), C2-C3: 1.442(2), C3-N1: 1.366(2), N1-N2: 1.318(2), N2-N3: 1.357(2), N3-C2: 1.407(2).

Table S3. Crystal data and structure refinement for **2** (different polymorph).

Empirical formula	$C_{33}H_{41}N_3$
Formula weight	479.69
Temperature/K	100.0
Crystal system	monoclinic
Space group	C2/c
a/Å	20.246(8)
b/Å	14.520(5)
c/Å	19.066(7)
α/°	90
β/°	91.222(10)
γ/°	90
Volume/Å^3	5603(4)
Z	8
ρ_{calc}g/cm^3	1.137
μ/mm^{-1}	0.066
F(000)	2080.0
Crystal size/mm^3	0.169 × 0.162 × 0.033
Radiation	MoKα (λ = 0.71073)
2Θ range for data collection/°	5.462 to 55.828
Index ranges	$-26 \leq h \leq 26, 0 \leq k \leq 19, 0 \leq l \leq 25$
Reflections collected	6711
Independent reflections	6711 [R_{int} = ?, R_{sigma} = 0.0301]
Data/restraints/parameters	6711/0/341
Goodness-of-fit on F^2	1.018
Final R indexes [I>=2σ (I)]	R_1 = 0.0463, wR_2 = 0.1046
Final R indexes [all data]	R_1 = 0.0665, wR_2 = 0.1157
Largest diff. peak/hole / e Å^{-3}	0.26/-0.21

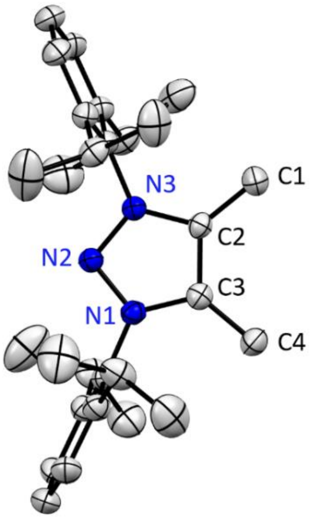

Figure S28. X-ray solid-state structure of **4** (CCDC: 1965564). Ellipsoids shown with 50% probability. Hydrogen atoms omitted for clarity. Stacking of molecules with $C^1=C^2H_2$, $C^3-C^4H_3$ and $C^1-C^2H_3$, $C^3=C^4H_2$. Averaged bond distances as a result. Selected bond parameters in [Å] and [°]: C1-C2 1.420(1), C2-C3 1.424(1), C3-C4 1.428(1), C3-N1 1.367(1), N1-N2 1.352(1), N2-N3 1.353(1), N3-C2 1.372(1).

Table S4. Crystal data and structure refinement for **4**.

Empirical formula	$C_{28}H_{39}N_3$
Formula weight	417.62
Temperature/K	150.0
Crystal system	triclinic
Space group	P-1
a/Å	8.8906(14)
b/Å	12.3017(15)
c/Å	13.3614(14)
α/°	66.525(3)
β/°	81.955(5)
γ/°	72.775(5)
Volume/Å^3	1279.9(3)
Z	2
ρ_{calc}g/cm^3	1.084
μ/mm^{-1}	0.063
F(000)	456.0
Crystal size/mm^3	0.433 × 0.362 × 0.126
Radiation	MoKα (λ = 0.71073)
2Θ range for data collection/°	5.248 to 57.604
Index ranges	$-12 \le h \le 12$, $-16 \le k \le 16$, $-18 \le l \le 18$
Reflections collected	48109
Independent reflections	6634 [R_{int} = 0.0246, R_{sigma} = 0.0156]
Data/restraints/parameters	6634/4/338
Goodness-of-fit on F^2	1.046
Final R indexes [I>=2σ (I)]	R_1 = 0.0449, wR_2 = 0.1193
Final R indexes [all data]	R_1 = 0.0506, wR_2 = 0.1243
Largest diff. peak/hole / e Å^{-3}	0.28/-0.18

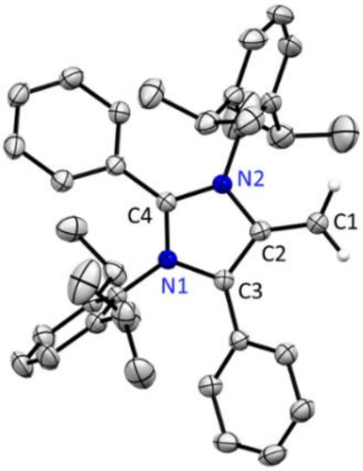

Figure S29. X-ray solid-state structure of **6** (CCDC: 1965561). Ellipsoids shown with 50% probability. Hydrogen atoms (except $=CH_2$) omitted for clarity. Selected bond parameters in [Å] and [°]: C1-C2 1.363(2), C2-C3 1.432(2), C3-N1 1.403(1), N1-C4 1.341(1), C4-N2 1.371(2), N2-C2 1.423(1).

Table S5. Crystal data and structure refinement for **6**.

Empirical formula	$C_{40}H_{46}N_2$
Formula weight	554.79
Temperature/K	150.0
Crystal system	monoclinic
Space group	$P2_1/n$
a/Å	17.5245(12)
b/Å	10.0335(7)
c/Å	19.5719(14)
α/°	90
β/°	107.959(3)
γ/°	90
Volume/Å^3	3273.7(4)
Z	4
ρ_{calc}g/cm^3	1.126
μ/mm^{-1}	0.485
F(000)	1200.0
Crystal size/mm^3	0.257 × 0.043 × 0.041
Radiation	CuKα (λ = 1.54178)
2Θ range for data collection/°	5.926 to 140.958
Index ranges	$-18 \le h \le 18$, $-13 \le k \le 13$, $-10 \le l \le 10$
Reflections collected	6233
Independent reflections	6233 [R_{int} = ?, R_{sigma} = 0.0245]
Data/restraints/parameters	6233/0/520
Goodness-of-fit on F^2	1.079
Final R indexes [I>=2σ (I)]	R_1 = 0.0396, wR_2 = 0.1032
Final R indexes [all data]	R_1 = 0.0447, wR_2 = 0.1067
Largest diff. peak/hole / e Å^{-3}	0.23/-0.21

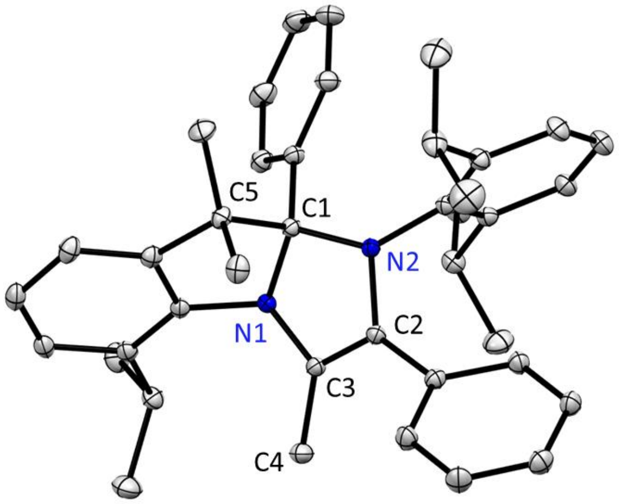

Figure S30. X-ray solid-state structure of **7** (CCDC: 1965563). Ellipsoids shown with 50% probability. Hydrogen atoms omitted for clarity. Selected bond parameters in [Å] and [°]: C1-N1 1.513(1), C1-N2 1.482(1), C1-C5 1.608(1), N2-C2 1.437(1), C2-C3 1.341(1), C3-N1 1.442(1), C3-C4 1.489(1).

Table S6. Crystal data and structure refinement for mo_0839_PA_0m.

Empirical formula	$C_{40}H_{46}N_2$
Formula weight	554.79
Temperature/K	100.0
Crystal system	triclinic
Space group	P-1
a/Å	9.5292(15)
b/Å	11.8160(18)
c/Å	14.788(3)
α/°	98.415(7)
β/°	93.288(6)
γ/°	101.439(6)
Volume/Å^3	1608.1(5)
Z	2
ρ_{calc}g/cm^3	1.146
μ/mm^{-1}	0.066
F(000)	600.0
Crystal size/mm^3	0.22 × 0.12 × 0.07
Radiation	MoKα (λ = 0.71073)
2Θ range for data collection/°	4.872 to 59.292
Index ranges	$-13 \leq h \leq 13$, $-16 \leq k \leq 16$, $-20 \leq l \leq 20$
Reflections collected	97890
Independent reflections	9062 [R_{int} = 0.0360, R_{sigma} = 0.0199]
Data/restraints/parameters	9062/0/388
Goodness-of-fit on F^2	1.065
Final R indexes [I>=2σ (I)]	R_1 = 0.0427, wR_2 = 0.1096
Final R indexes [all data]	R_1 = 0.0486, wR_2 = 0.1146
Largest diff. peak/hole / e Å^{-3}	0.46/-0.24

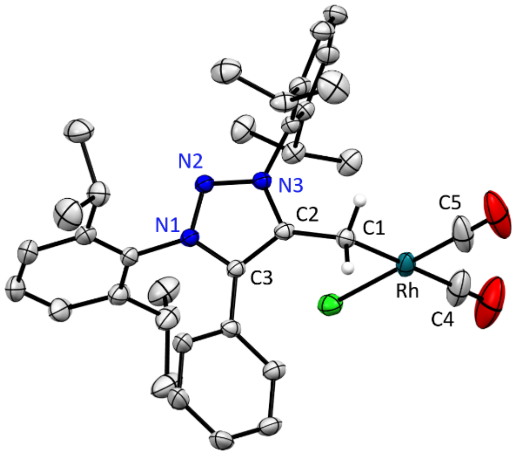

Figure S31. X-ray solid-state structure of **8** (CCDC: 1965571). Ellipsoids shown with 50% probability. Hydrogen atoms (except $-CH_2$) and a molecule of toluene omitted for clarity. Selected bond parameters in [Å] and [°]: C1-Rh: 2.151(2), C4-Rh 1.882(2), C5-Rh 1.826(2), Rh-Cl 2.362(1), C1-C2: 1.457(3), C2-C3: 1.393(2), C3-N1: 1.368(2), N1-N2: 1.324(2), N2-N3: 1.336(2), N3-C2: 1.367(2).

Table S7. Crystal data and structure refinement for **8**.

Empirical formula	$C_{38.5}H_{45}ClN_3O_2Rh$
Formula weight	720.13
Temperature/K	100.0
Crystal system	monoclinic
Space group	$P2_1/c$
a/Å	11.3435(16)
b/Å	16.599(2)
c/Å	19.490(3)
α/°	90
β/°	100.649(3)
γ/°	90
Volume/Å^3	3606.5(9)
Z	4
ρ_{calc}g/cm^3	1.326
μ/mm^{-1}	0.584
F(000)	1500.0
Crystal size/mm^3	0.557 × 0.134 × 0.108
Radiation	MoKα (λ = 0.71073)
2Θ range for data collection/°	4.586 to 57.504
Index ranges	$-15 \le h \le 14$, $-19 \le k \le 22$, $-25 \le l \le 26$
Reflections collected	43711
Independent reflections	9245 [R_{int} = 0.0313, R_{sigma} = 0.0253]
Data/restraints/parameters	9245/122/451
Goodness-of-fit on F^2	1.028
Final R indexes [I>=2σ (I)]	R_1 = 0.0316, wR_2 = 0.0696
Final R indexes [all data]	R_1 = 0.0415, wR_2 = 0.0752
Largest diff. peak/hole / e Å^{-3}	0.65/-0.66

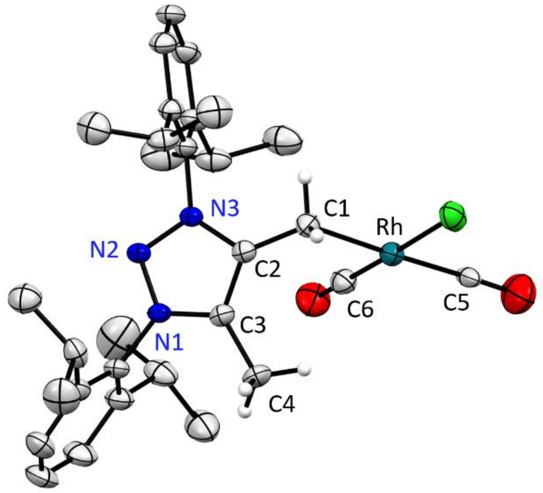

Figure S32. X-ray solid-state structure of **9** (CCDC: 1965569). Ellipsoids shown with 50% probability. Hydrogen atoms (except -CH_2 and –CH_3) omitted for clarity. Selected bond parameters in [Å] and [°]: C1-Rh: 2.137(2), C5-Rh 1.871(2), C6-Rh 1.795(2), Rh-Cl 2.3572(6), C1-C2: 1.458(3), C2-C3: 1.384(3), C3-N1: 1.351(2), N1-N2: 1.331(2), N2-N3: 1.337(2), N3-C2: 1.364(2), C3-C4 1.485(3).

Table S8. Crystal data and structure refinement for **9**.

Empirical formula	$C_{30}H_{39}ClN_3O_2Rh$
Formula weight	612.00
Temperature/K	150.0
Crystal system	monoclinic
Space group	$P2_1/n$
a/Å	13.8016(12)
b/Å	17.0420(11)
c/Å	14.2129(12)
α/°	90
β/°	108.880(3)
γ/°	90
Volume/Å^3	3163.1(4)
Z	4
ρ_{calc}g/cm^3	1.285
μ/mm^{-1}	0.653
F(000)	1272.0
Crystal size/mm^3	0.53 × 0.351 × 0.224
Radiation	MoKα (λ = 0.71073)
2Θ range for data collection/°	5.002 to 57.648
Index ranges	$-18 \leq h \leq 18$, $-23 \leq k \leq 23$, $-19 \leq l \leq 19$
Reflections collected	65174
Independent reflections	8207 [R_{int} = 0.0299, R_{sigma} = 0.0170]
Data/restraints/parameters	8207/0/343
Goodness-of-fit on F^2	1.056
Final R indexes [I>=2σ (I)]	R_1 = 0.0321, wR_2 = 0.0876
Final R indexes [all data]	R_1 = 0.0395, wR_2 = 0.0931
Largest diff. peak/hole / e Å^{-3}	0.81/-0.83

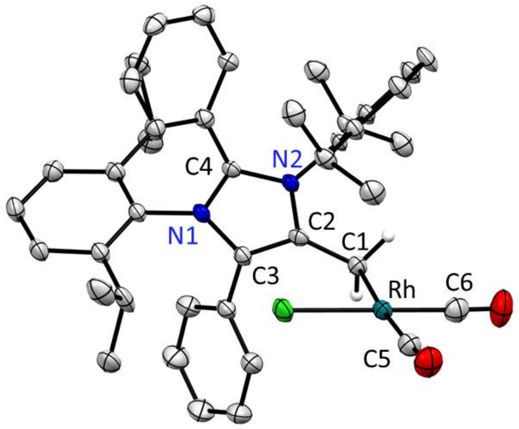

Figure S33. X-ray solid-state structure of **10** (CCDC: 1965565). Ellipsoids shown with 50% probability. Hydrogen atoms (except $-CH_2$ and $–CH_3$) omitted for clarity. Selected bond parameters in [Å] and [°]: C1-Rh: 2.158(4), C5-Rh 1.892(2), C6-Rh 1.816(5), Rh-Cl 2.388(1), C1-C2: 1.461(5), C2-C3: 1.375(5), C3-N1: 1.406(5), N1-C4 1.350(4):, C4-N2: 1.353(4), N2-C2: 1.394(5).

Table S9. Crystal data and structure refinement for **10**.

Empirical formula	$C_{42}H_{46}ClN_2O_2Rh$
Formula weight	749.17
Temperature/K	100.0
Crystal system	monoclinic
Space group	$P2_1/n$
a/Å	12.702(4)
b/Å	15.528(6)
c/Å	19.063(7)
α/°	90
β/°	95.010(7)
γ/°	90
Volume/Å^3	3745(2)
Z	4
ρ_{calc}g/cm^3	1.329
μ/mm^{-1}	0.564
F(000)	1560.0
Crystal size/mm^3	0.309 × 0.276 × 0.09
Radiation	MoKα (λ = 0.71073)
2Θ range for data collection/°	4.02 to 57.544
Index ranges	$-17 \leq h \leq 16$, $-20 \leq k \leq 20$, $-25 \leq l \leq 25$
Reflections collected	91405
Independent reflections	9680 [R_{int} = 0.0612, R_{sigma} = 0.0352]
Data/restraints/parameters	9680/0/441
Goodness-of-fit on F^2	1.219
Final R indexes [I>=2σ (I)]	R_1 = 0.0561, wR_2 = 0.1190
Final R indexes [all data]	R_1 = 0.0739, wR_2 = 0.1269
Largest diff. peak/hole / e Å^{-3}	0.81/-1.10

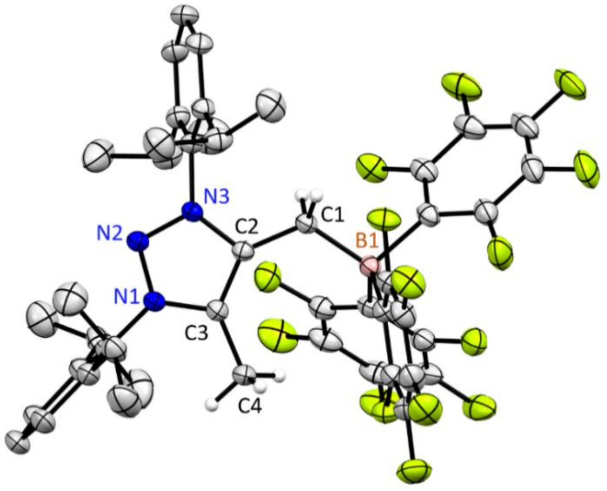

Figure S34. X-ray solid-state structure of **12** (CCDC: 1965567). Ellipsoids shown with 50% probability. Hydrogen atoms (except -CH_2 and –CH_3) omitted for clarity. Selected bond parameters in [Å] and [°]: B1-C1 1.660(2), C1-C2 1.496(2), C2-C3 1.384(2), C3-N1 1.366(2), N1-N2 1.327(1), N2-N3 1.328(1), N3-C2 1.374(2), C3-C4 1.490(2).

Table S10. Crystal data and structure refinement for **12**.

Empirical formula	$C_{46}H_{39}BF_{15}N_3$
Formula weight	929.61
Temperature/K	150.0
Crystal system	triclinic
Space group	P-1
a/Å	12.7053(10)
b/Å	13.5550(11)
c/Å	13.9569(12)
α/°	74.088(2)
β/°	89.090(2)
γ/°	68.468(2)
Volume/Å^3	2140.6(3)
Z	2
ρ_{calc}g/cm^3	1.442
μ/mm^{-1}	0.130
F(000)	952.0
Crystal size/mm^3	0.53 × 0.351 × 0.224
Radiation	MoKα (λ = 0.71073)
2Θ range for data collection/°	4.824 to 57.512
Index ranges	$-17 \le h \le 17$, $-17 \le k \le 18$, $-18 \le l \le 18$
Reflections collected	48018
Independent reflections	11049 [R_{int} = 0.0272, R_{sigma} = 0.0219]
Data/restraints/parameters	11049/0/595
Goodness-of-fit on F^2	1.038
Final R indexes [I>=2σ (I)]	R_1 = 0.0429, wR_2 = 0.1072
Final R indexes [all data]	R_1 = 0.0521, wR_2 = 0.1152
Largest diff. peak/hole / e Å^{-3}	0.72/-0.21

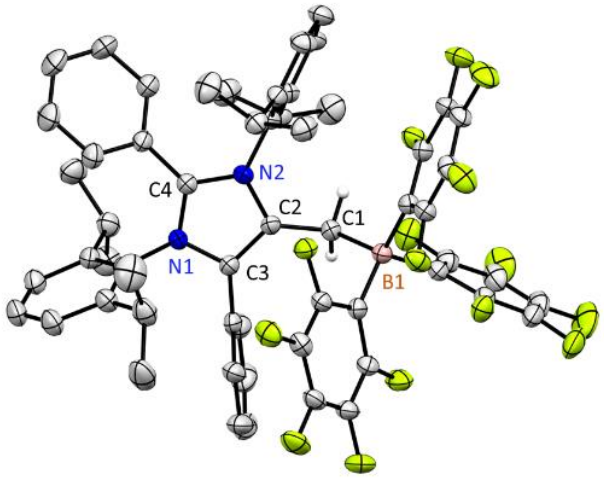

Figure S35. X-ray solid-state structure of **13** (CCDC: 1965568). Ellipsoids shown with 50% probability. Hydrogen atoms (except -CH_2) and a molecule of toluene omitted for clarity. Selected bond parameters in [Å] and [°]: C1-B1 1.661(5), C1-C2 1.482(4), C2-C3 1.368(4), C3-N1 1.393(4), N1-C4 1.349(4), C4-N2 1.356(4), N2-C2 1.400(4).

Table S11. Crystal data and structure refinement for **13**.

Empirical formula	$C_{65}H_{54}BF_{15}N_2$
Formula weight	1158.91
Temperature/K	150.0
Crystal system	monoclinic
Space group	$P2_1$
a/Å	11.582(2)
b/Å	13.655(2)
c/Å	17.241(3)
α/°	90
β/°	95.562(5)
γ/°	90
Volume/Å^3	2713.9(8)
Z	2
ρ_{calc}g/cm^3	1.418
μ/mm^{-1}	0.118
F(000)	1196.0
Crystal size/mm^3	0.394 × 0.258 × 0.152
Radiation	MoKα (λ = 0.71073)
2Θ range for data collection/°	4.444 to 57.662
Index ranges	$-15 \leq h \leq 15$, $-18 \leq k \leq 18$, $-23 \leq l \leq 22$
Reflections collected	36579
Independent reflections	13279 [R_{int} = 0.0273, R_{sigma} = 0.0347]
Data/restraints/parameters	13279/123/757
Goodness-of-fit on F^2	1.047
Final R indexes [I>=2σ (I)]	R_1 = 0.0495, wR_2 = 0.1279
Final R indexes [all data]	R_1 = 0.0649, wR_2 = 0.1398
Largest diff. peak/hole / e Å^{-3}	0.60/-0.48
Flack parameter	0.5

9. IR-spectroscopy

Comparison with reported [(NHO)RhCl(CO)₂] complexes:

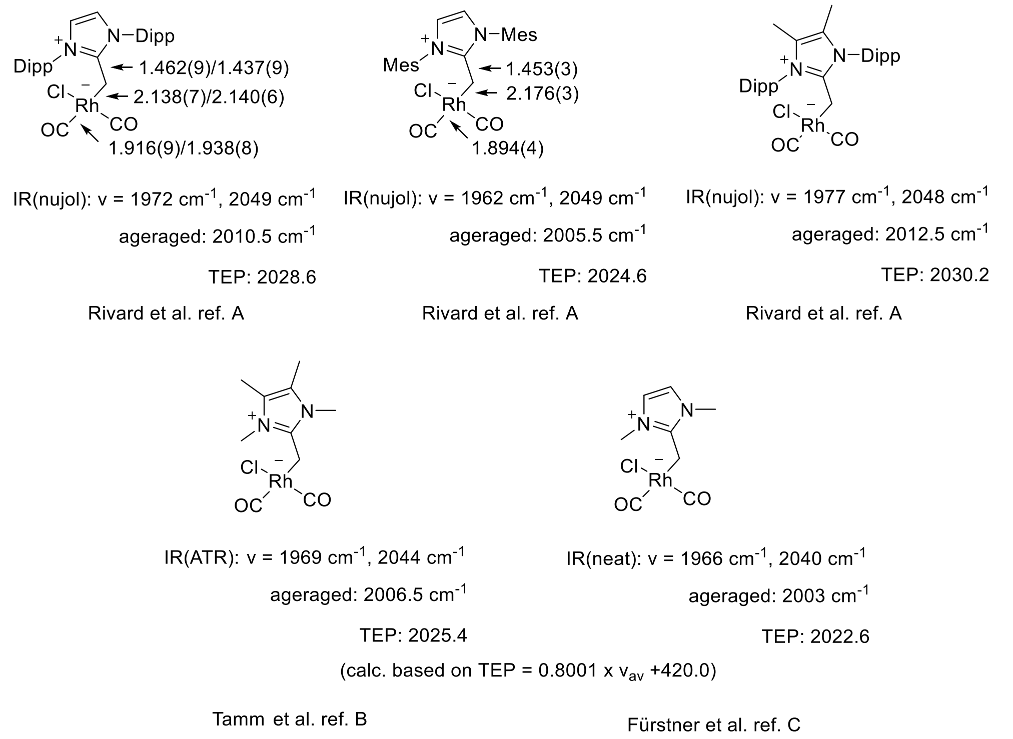

Figure S36. Summary of all reported [(NHO)RhCl(CO)₂] complexes with their frequencies and bonding parameters. Ref: A,[1] Ref. B,[20] Ref. C.[2]

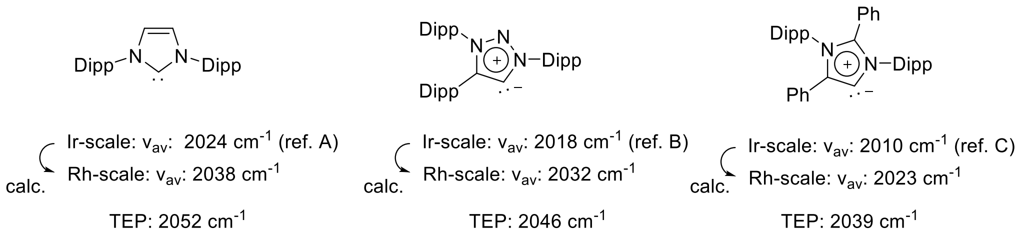

Figure S37. Comparison with reported Rh-complexes. Calculation of Ir into Rh-scale based on $\nu_{av}(Rh) = 1.035\ \nu_{av}(Ir) - 56.9\ cm^{-1}$.[21] Ref A.[22]; Ref. B[13]; Ref. C[23].

We noticed that the IR values reported in literature were measured by different techniques (nujol, ATR etc., Figure S36) leading to problems to compare. In general, typical TEP parameter measurements are performed in solution in CH_2Cl_2.[24] A clear difference between solution and ATR measurement has been reported, in which ATR measurements typically give the smallest IR stretching frequencies.[24]

In order to check the influence of the IR technique onto the carbonyl stretching frequencies we also measured the compounds as solids by ATR (Table S12; Figures S39-S43). As expected ATR measured ν_{av} values are by 10 to 5 cm^{-1} lower than the corresponding solution phase values (Table S12; Figure S38). ATR measurements were performed on a Jasco FT/IR 4600 as solid between a

diamond stamp. Solution phase (CH_2Cl_2) IRs were measured on a Bruker Vertex 70 spectrometer in a Specac Omni cell (Figure S38).

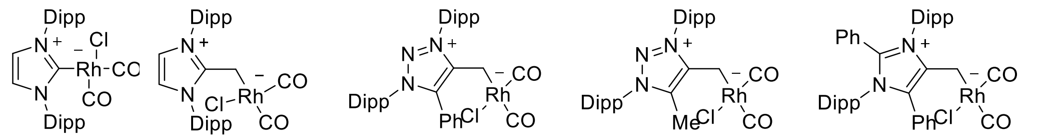

ATR					
v [cm^{-1}]	2071.2	2045.1	2046.1	2046.1	2040.3
v [cm^{-1}]	1986.3	1966.1	1968.9	1962.2	1955.5
v_{av}	**2028.8**	**2005.6**	**2007.5**	**2004.2**	**1997.9**
TEP	2043.2	2024.7	2026.2	2023.5	2018.5
**Solution CH_2Cl_2**					
v [cm^{-1}]	2080.3	2056.6	2053.4	2052.5	2046.9
v [cm^{-1}]	1996.9	1971.5	1971.6	1971.5	1960.7
v_{av}	**2038.6**	**2014.1**	**2012.5**	**2012.0**	**2003.8**
TEP	2051.1	2031.4	2030.2	2029.8	2023.2
Δv [cm^{-1}]	9.8	8.4	5.0	7.8	5.9

Table S12. Comparison of IR bands measured with ATR and in solution (CH_2Cl_2).

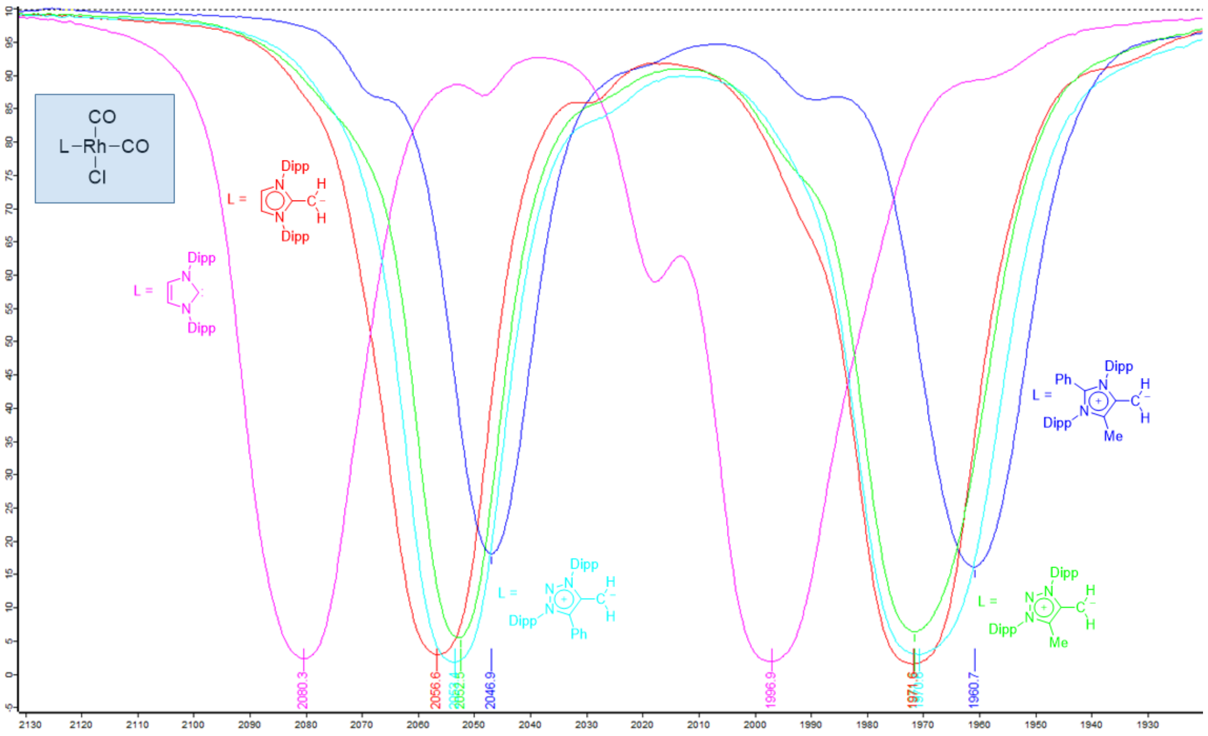

Figure S38. Overlay of measured IR spectra (in CH_2Cl_2); cut-out of the carbonyl group area.

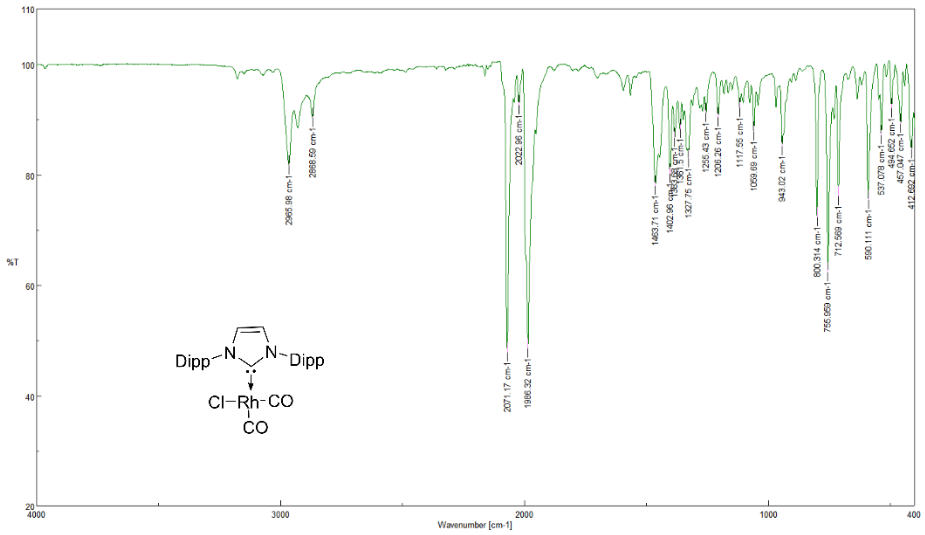

Figure S39. ATR-IR spectrum of (IPr)RhCl$(CO)_2$ (measured as solid).

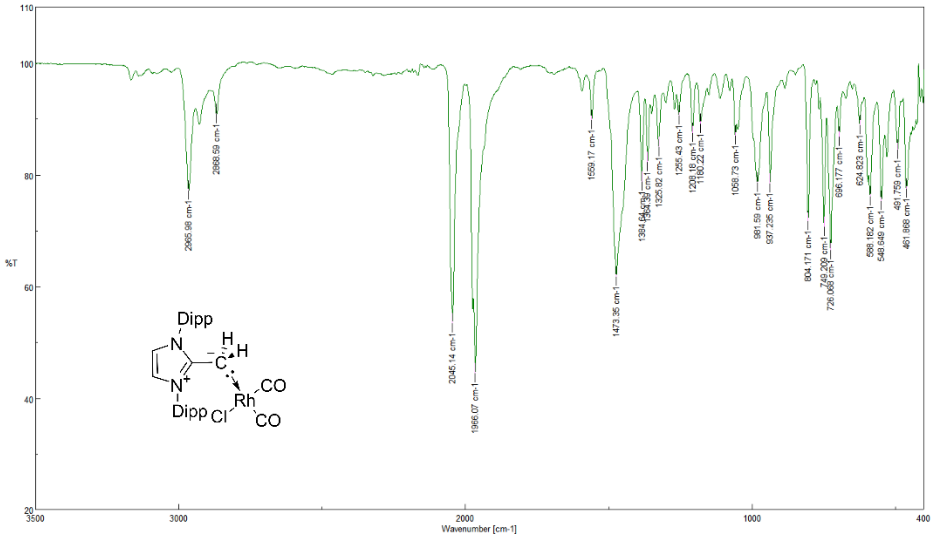

Figure S40. ATR-IR spectrum of ($IPrCH_2$)RhCl$(CO)_2$ (measured as solid).

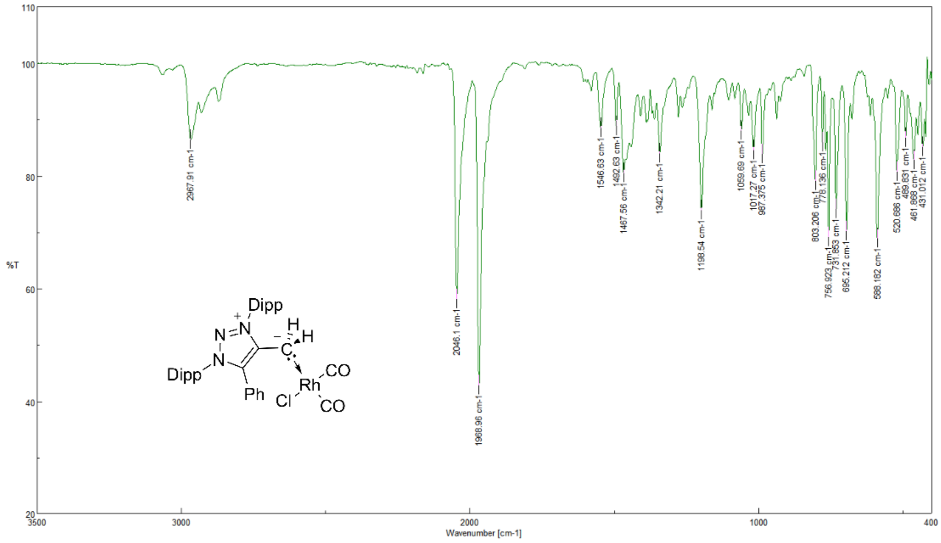

Figure S41. ATR-IR spectrum of (**2**)RhCl$(CO)_2$ (measured as solid).

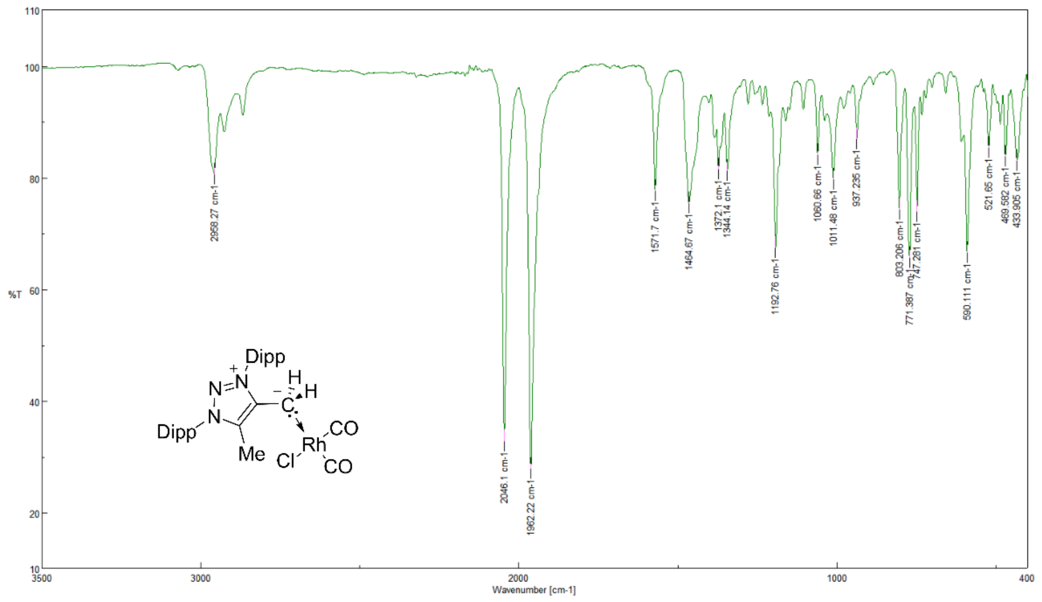

Figure S42. ATR-IR spectrum of (**4**)RhCl(CO)$_2$ (measured as solid).

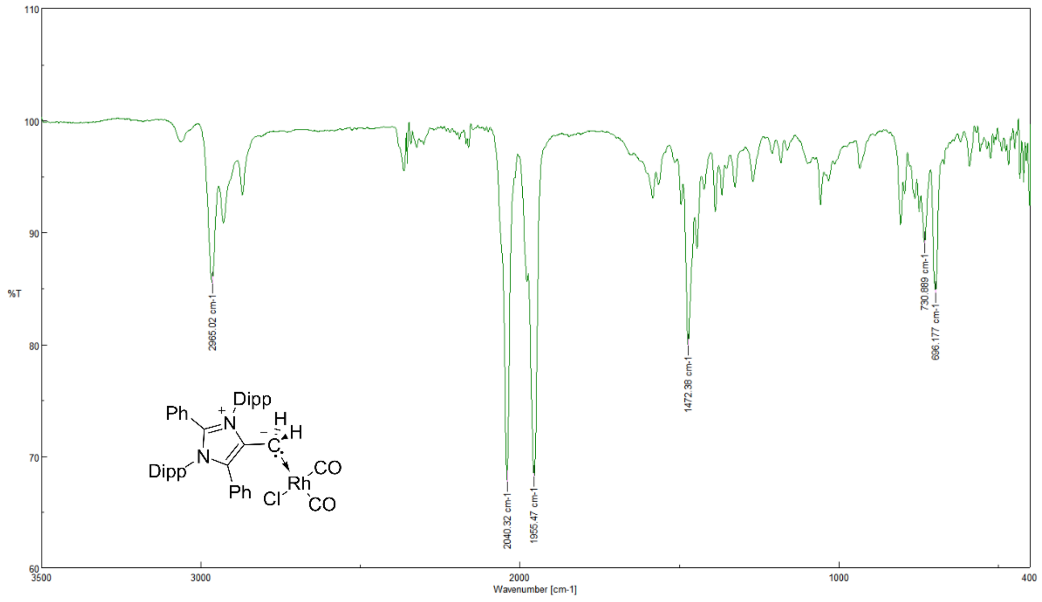

Figure S43. ATR-IR spectrum of (**6**)RhCl(CO)$_2$ (measured as solid).

10. UV-VIS spectroscopy

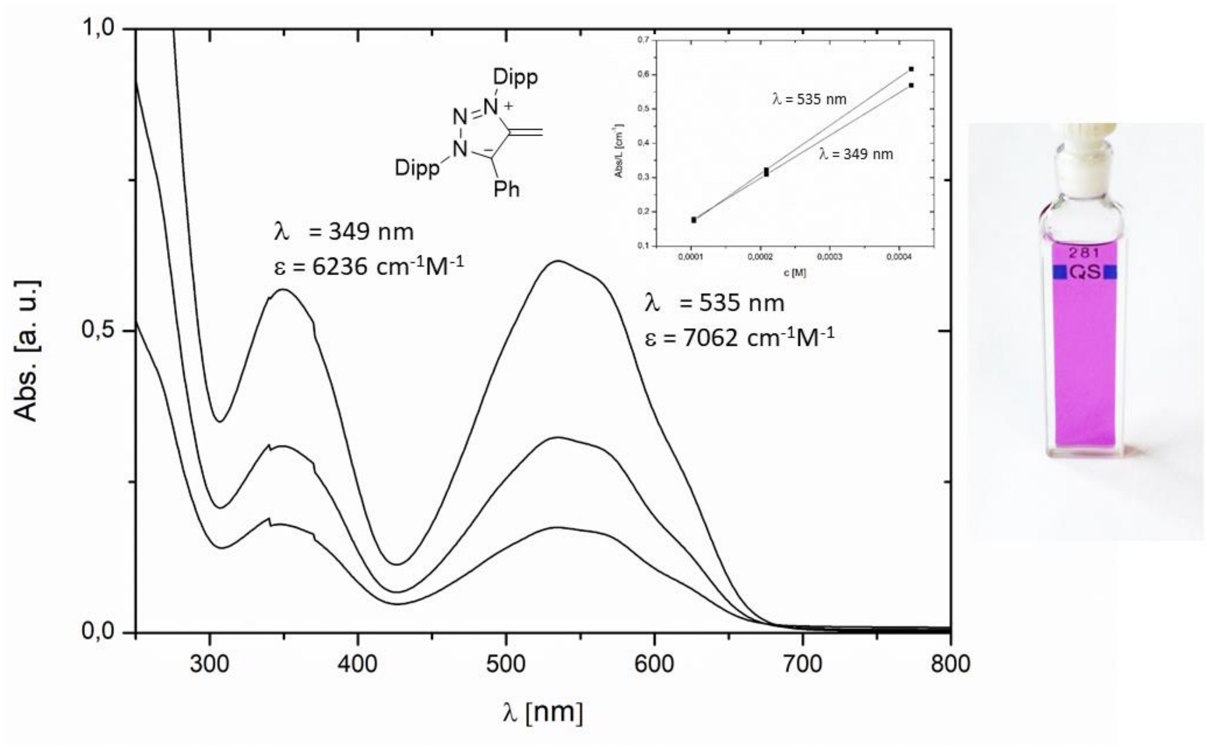

Figure S44. UV-VIS spectra of compound **2**.

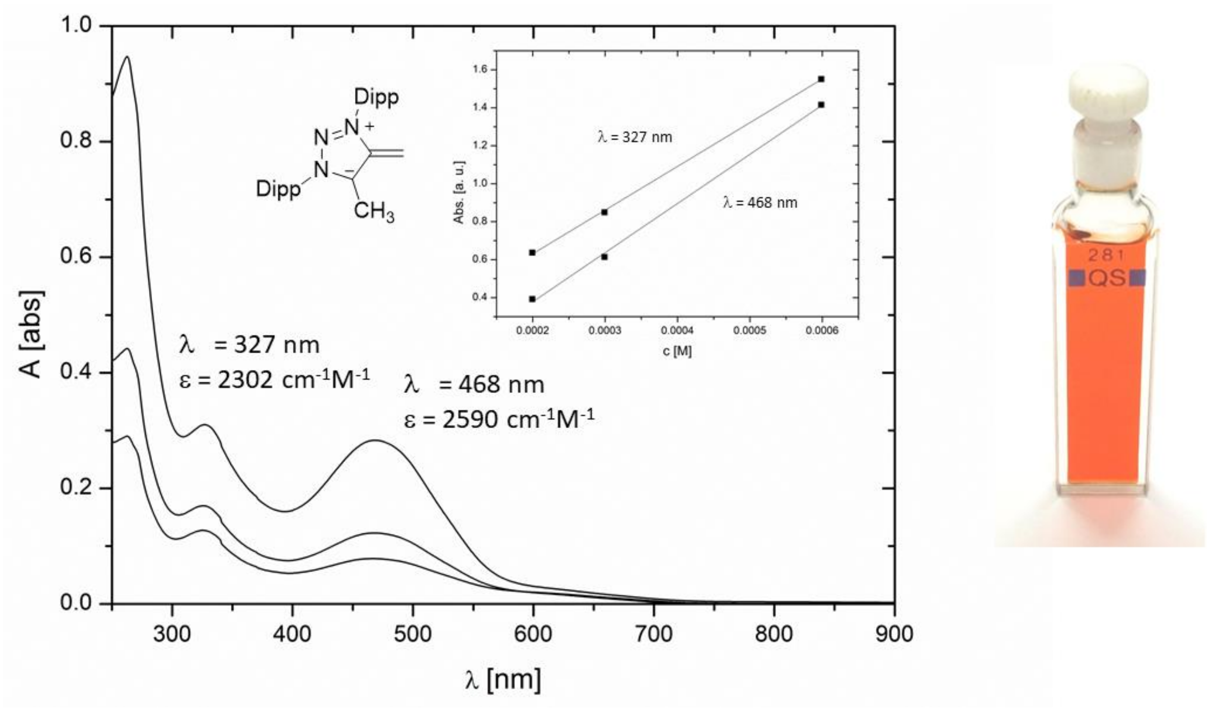

Figure S45. UV-VIS spectra of compound **4**.

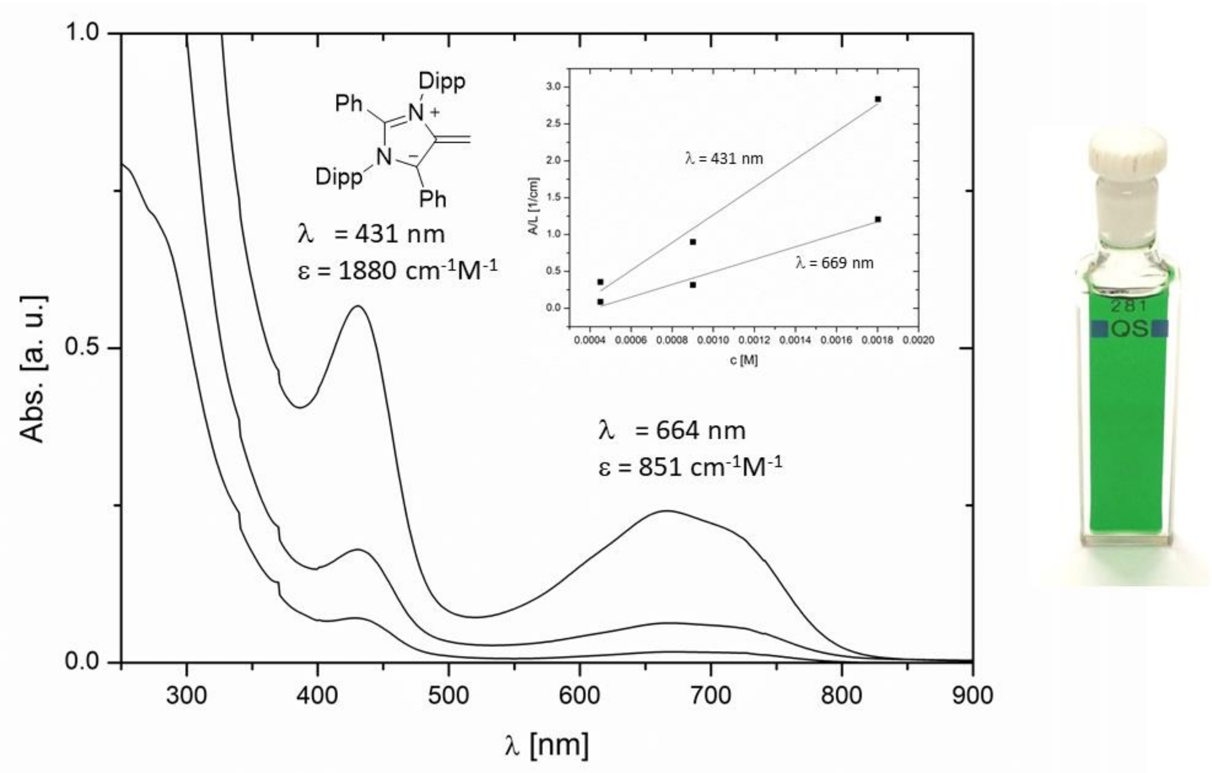

Figure S46. UV-VIS spectra of compound **6**.

11. Computational details

General part

All calculations were performed with the Gaussian16 program package[25] (version g16, rev.a03). The theoretical approach is based on the framework of density functional theory (DFT).[26] All calculations were performed with either the B3LYP or the BLYP functionals and Ahlrichs redefined triple-ζ double valence polarized basis set [def2-TZVP(P)].[27] Ground states were fully optimized without constraints at the corresponding level of theory and verified by a frequency calculation. Gibbs free reaction energies and enthalpies were calculated for standard conditions (p = 1 atm, T = 298 K) and are unscaled. NRT calculations were performed with NBO 6.0[28] implemented into the Gaussian16 program suite. Proton affinities (PAs) were calculated according to: $E(PA) = E_{(NHO/mNHO)}(\text{protonated}) - E_{(NHO/mNHO)}$. The BLYP/def2-TZVPP level of theory was chosen to be consistent and comparable with the recent theoretical analysis of NHOs by Naumann et al. (see a benchmarking there).[29] For the visualization of of molecular orbitals IBOView[30] or GausView 6[31] were used and for the visualization of structures CYLview.[32]

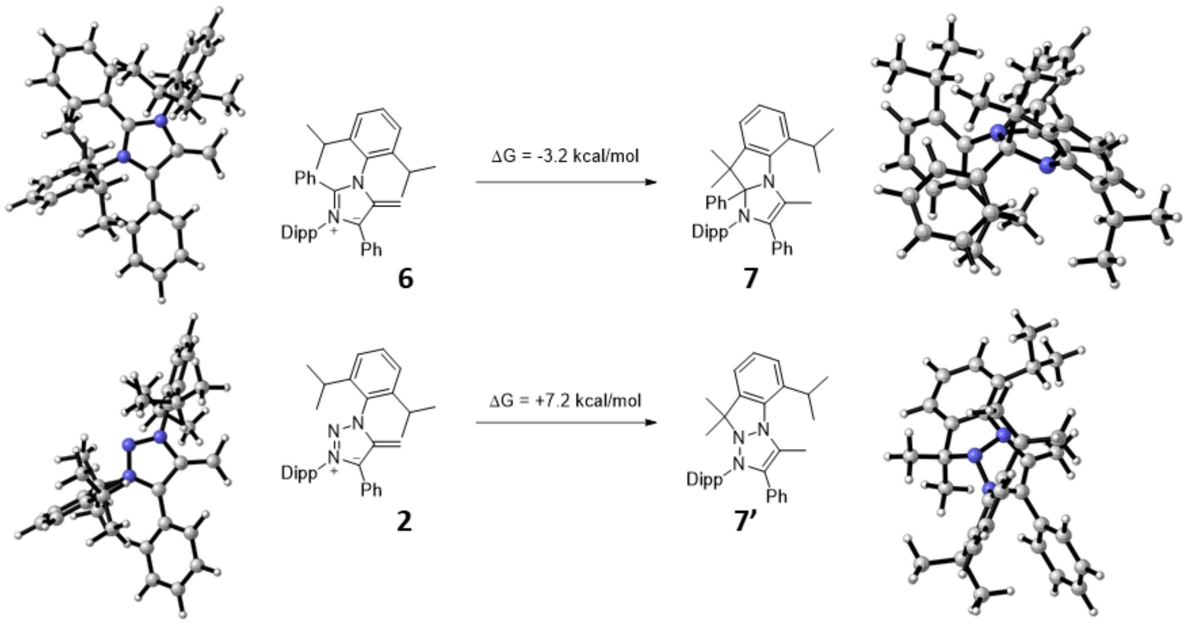

Figure S47. Calculation of the thermodynamics for the exergonic rearrangement of **6** to **7** and the hypothetical endergonic reaction of **2** to **7’** at the B3LYP-D3(BJ)/def2-TZVP level of theory. Without D3(BJ)[33] correction the energies amount to + 2.4 kcal/mol (top) and +11.7 kcal/mol (bottom).

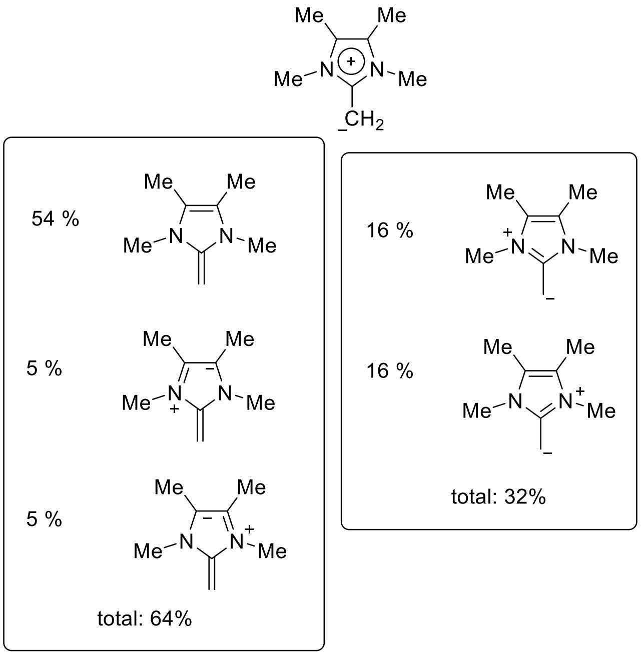

Figure S48. Summary of resonance structures based on NRT calculations at the B3LYP/def2-TZVPP level of theory for traditional NHO **A**.

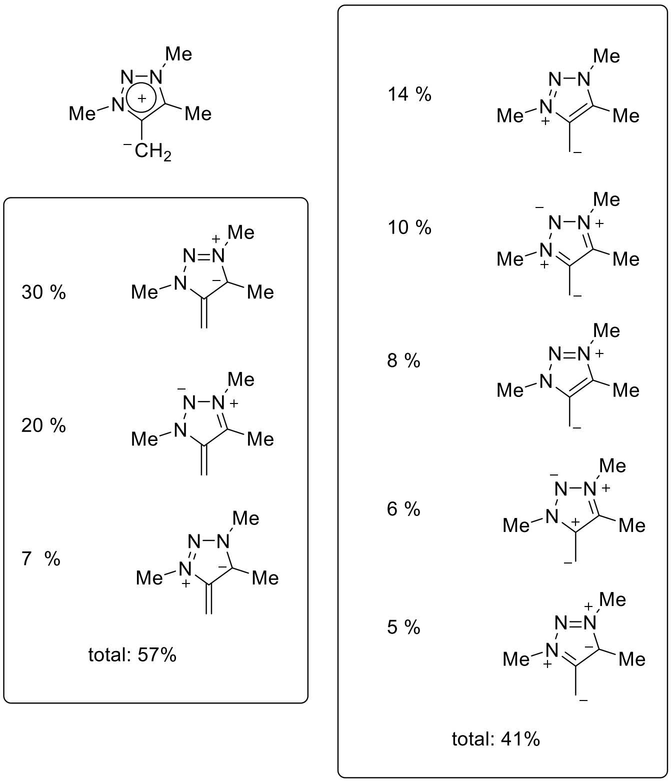

Figure S49. Summary of resonance structures based on NRT calculations at the B3LYP/def2-TZVPP level of theory for mNHO **B**.

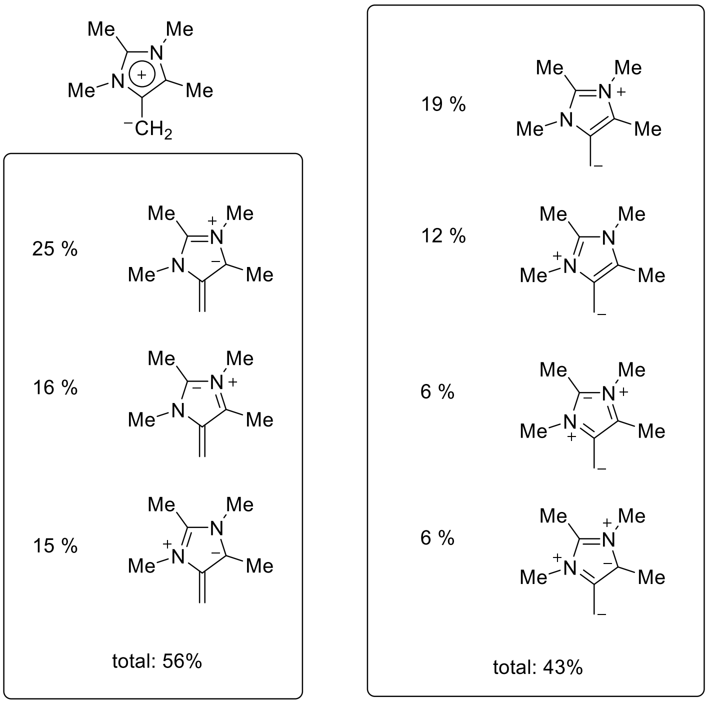

Figure S50. Summary of resonance structures based on NRT calculations at the B3LYP/def2-TZVPP level of theory for mNHO **C**.

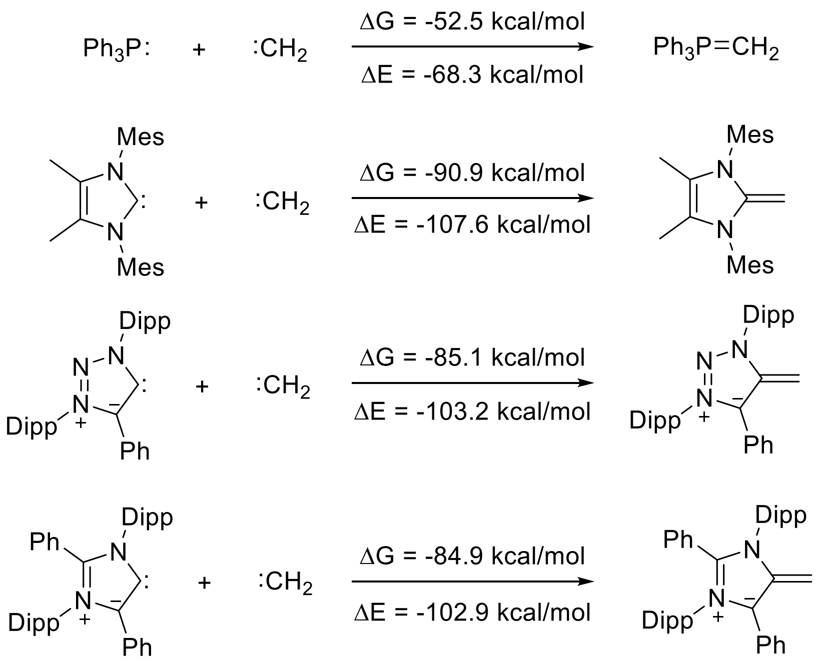

Figure S51. Formal fragmentation of the double bond into carbene and triplet methylene. Energies are calculated at the B3LYP/def2-TZVPP level of theory.

TD-DFT calculation of UV-Vis spectra

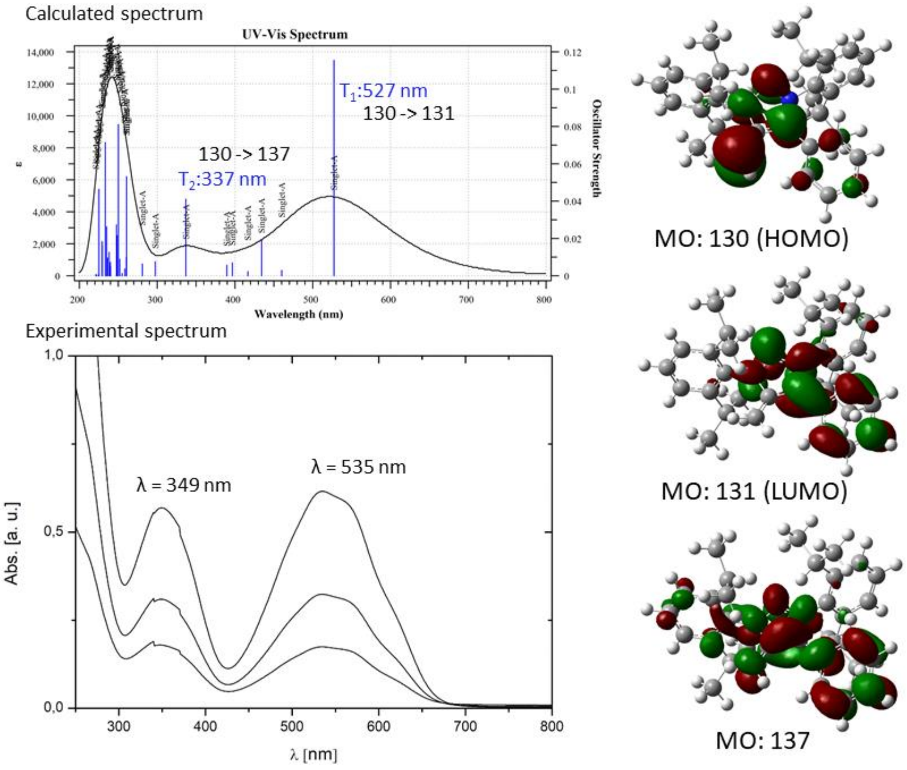

Figure S52. Comparison between calculated (top) [TD-DFT B3LYP-D3BJ/def2TZVPP] and experimental (bottom) UV-Vis spectrum for **2**. Visualization of selected molecular orbitals (right); isovalue 0.03.

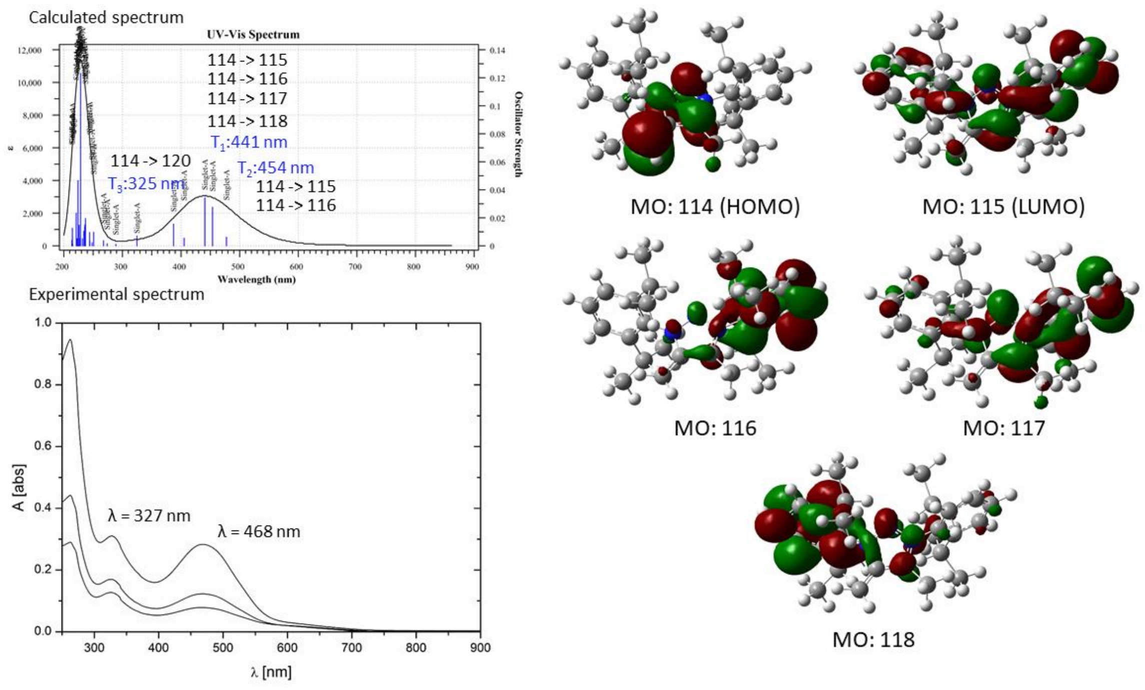

Figure S53. Comparison between calculated (top) [TD-DFT B3LYP-D3BJ/def2TZVPP] and experimental (bottom) UV-Vis spectrum for **4**. Visualization of selected molecular orbitals (right); isovalue 0.03.

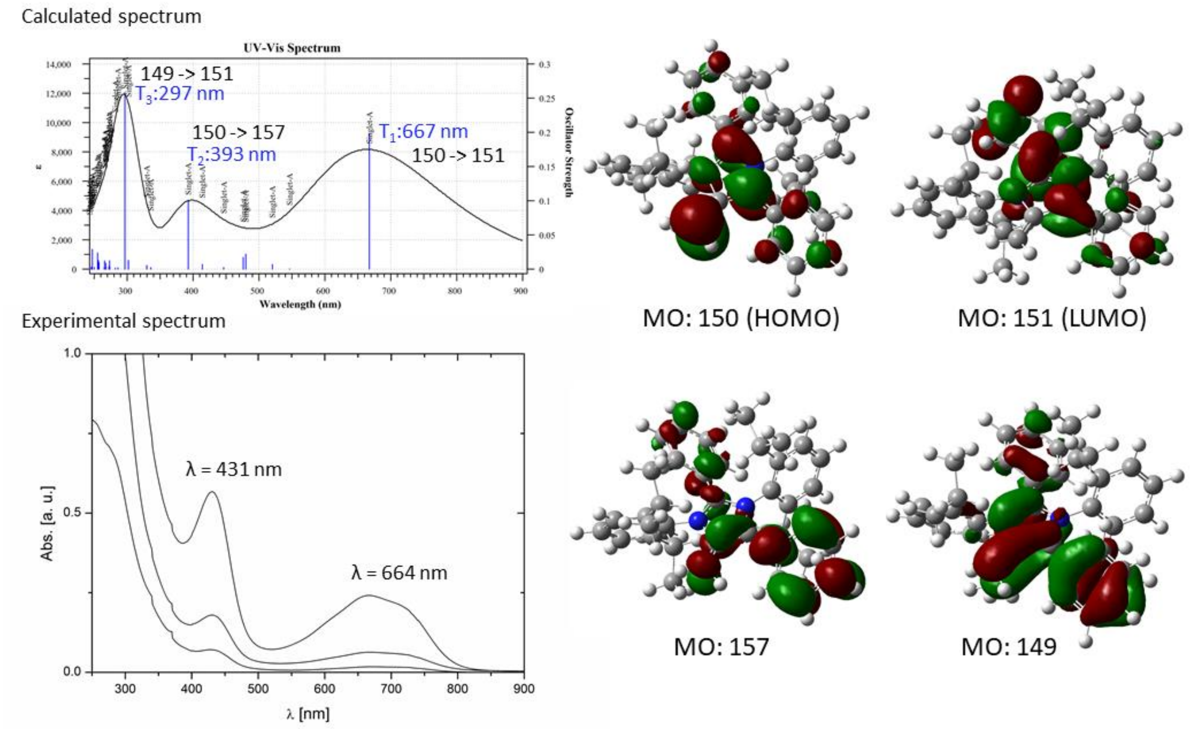

Figure S54. Comparison between calculated (top) [TD-DFT B3LYP-D3BJ/def2TZVPP] and experimental (bottom) UV-Vis spectrum for **6**. Visualization of selected molecular orbitals (right); isovalue 0.03.

Optimized x,y,z-coordinates

Simple systems

Structure A

24

scf done: -422.711436

N	-0.726339	-0.916990	-0.024238
C	-1.165382	0.410897	-0.018364
N	0.006888	1.173038	0.013201
C	1.140671	0.324003	0.025090
C	0.689174	-0.962984	0.001836
C	-2.456285	0.864133	-0.039369
C	-0.016069	2.622250	0.027483
C	2.535475	0.861552	0.058418
C	1.442297	-2.254640	0.002463
C	-1.649547	-2.033937	-0.056965
H	-1.093491	-2.974236	-0.056280
H	-2.316073	-2.018597	0.819375
H	-2.278679	-1.997651	-0.959985
H	1.201410	-2.878282	0.877263
H	1.235178	-2.859484	-0.893893
H	2.519656	-2.063574	0.025016
H	3.257240	0.038896	0.060774
H	2.760769	1.492926	-0.814967
H	2.727792	1.469276	0.956156
H	1.005435	3.008644	0.051951
H	-0.520149	3.016290	-0.868722
H	-0.555706	2.998005	0.910708
H	-3.289431	0.173267	-0.063344
H	-2.675166	1.924338	-0.031891

Structure A (protonated)

25

scf done: -423.148942

N	-0.026668	1.163047	-0.043537
C	1.109256	0.340675	0.058343
C	0.661604	-0.958539	0.014762
N	-0.735496	-0.897777	-0.109593
C	-1.141368	0.395610	-0.138678
C	2.493338	0.893198	0.184732
C	1.405056	-2.254519	0.075561
C	-1.612030	-2.079664	-0.199680
C	-2.556686	0.860360	-0.255902
C	0.028767	2.637052	-0.026098
H	-1.316769	-2.693585	-1.055906
H	-1.533232	-2.672897	0.716717
H	-2.645964	-1.761162	-0.332643
H	1.087937	-2.866178	0.930451
H	1.258410	-2.851157	-0.834573
H	2.477375	-2.070903	0.180192
H	3.216105	0.078787	0.279589
H	2.778503	1.486825	-0.694054
H	2.597769	1.533996	1.069891
H	0.804814	2.976352	-0.716913
H	-0.926872	3.050130	-0.347638
H	0.258846	2.994049	0.983184
H	-3.194857	0.361866	0.483147
H	-2.636310	1.934834	-0.080984
H	-2.965684	0.650472	-1.253347

Structure B

24

scf done: -422.679133

C	1.163103	-0.357336	-0.114413

C	0.778402	1.018448	-0.082179
N	-0.648711	0.970886	-0.015315
C	-1.099594	-0.330436	0.090093
N	0.001885	-1.119307	0.022898
C	1.525132	2.177199	-0.070706
C	-1.454464	2.173992	0.070839
C	-2.531802	-0.737752	0.007786
C	-0.001631	-2.582142	0.033285
C	2.553952	-0.890874	-0.036667
H	-2.517025	1.921806	0.101867
H	-1.190810	2.748204	0.972752
H	-1.263986	2.815173	-0.801269
H	-2.650724	-1.811458	0.174497
H	-3.142216	-0.228958	0.767661
H	-2.981552	-0.505600	-0.974871
H	0.655496	-2.952572	0.827694
H	-1.009906	-2.954884	0.215373
H	0.354749	-2.970803	-0.928807
H	2.609378	-1.965361	-0.247087
H	3.193773	-0.384373	-0.774574
H	3.025392	-0.718828	0.950039
H	1.069612	3.160204	-0.067101
H	2.607103	2.123221	-0.108383

Structure B (protonated)

25

scf done: -423.148942

C	0.188430	1.141261	-0.019801
N	-1.021457	0.527443	-0.021668
C	-0.846729	-0.867475	0.003740
C	0.509030	-1.095721	0.012967
N	1.128306	0.164126	-0.004044
C	-2.342189	1.184449	-0.029286
C	-1.998041	-1.822090	0.015216
C	1.288598	-2.371569	0.034443
C	2.587986	0.370516	-0.009694
C	0.461596	2.610282	-0.033960
H	2.808215	1.436562	-0.066233
H	3.025927	-0.039329	0.905811
H	3.027961	-0.129443	-0.877825
H	-0.451934	3.184432	0.131060
H	1.166747	2.884974	0.759302
H	0.890424	2.928528	-0.993679
H	-2.815079	1.092370	0.954031
H	-2.232917	2.238795	-0.281638
H	-2.976007	0.709988	-0.782796
H	-2.611903	-1.733320	-0.891020
H	-1.632826	-2.851060	0.065085
H	-2.653571	-1.660586	0.880800
H	1.927241	-2.473524	-0.853002
H	0.610787	-3.228745	0.053087
H	1.934194	-2.439432	0.919955

Structure C

20

scf done: -399.418202

C	0.010821	0.968677	-0.019358
C	1.061861	-0.015199	-0.010234
N	0.330431	-1.221419	0.028257
N	-1.035519	-1.050183	0.042768
N	-1.161509	0.278686	0.012957
C	2.427843	0.119117	-0.031902
C	0.888358	-2.554518	0.052399
C	-2.522640	0.819126	0.018712
C	0.154900	2.451119	-0.056160
H	-0.812088	2.965365	-0.055711

H	0.730822	2.812922	0.810482
H	0.707849	2.771309	-0.953575
H	-2.676232	1.450420	0.900592
H	-2.700239	1.408884	-0.887085
H	-3.208497	-0.028943	0.047624
H	0.066683	-3.274091	0.080436
H	1.502593	-2.730298	-0.843229
H	1.526433	-2.688089	0.938605
H	3.089116	-0.739577	-0.020417
H	2.875159	1.105941	-0.061292

Structure C (protonated)

21

scf done: -399.861419

N	-1.139626	0.285044	-0.043521
C	0.049396	0.979566	-0.050150
C	1.037992	-0.001220	-0.049178
N	0.352948	-1.195702	-0.042049
N	-0.968476	-1.035015	-0.038569
C	0.134489	2.471104	-0.056634
C	2.528784	0.095690	-0.054259
C	0.903983	-2.563708	-0.038123
C	-2.511917	0.825285	-0.041310
H	-0.346916	2.903074	0.830101
H	1.179185	2.792549	-0.061059
H	-0.352174	2.895668	-0.944071
H	-2.661412	1.437356	0.853055
H	-2.666734	1.431120	-0.939004
H	-3.199329	-0.021591	-0.036326
H	0.062520	-3.257732	-0.033062
H	1.511659	-2.714610	-0.935238
H	1.516560	-2.707518	0.856827
H	2.963646	-0.381430	0.833372
H	2.841936	1.142898	-0.059372
H	2.958115	-0.388417	-0.940795

Full systems

Structure D

56

scf done: -1042.001608

C	0.000017	-0.001399	-0.443983
N	1.111434	0.001029	0.418279
N	-1.111418	0.001211	0.418214
C	-0.680173	0.005378	1.775189
C	0.680147	0.005249	1.775199
C	-1.657239	0.008374	2.907973
H	-2.313446	-0.874010	2.892037
H	-1.126562	0.010996	3.865263
H	-2.313642	0.890510	2.887341
C	1.657233	0.008536	2.907986
H	1.126599	0.011508	3.865298
H	2.313419	-0.873865	2.892329
H	2.313637	0.890659	2.886989
C	2.476987	-0.000119	-0.030619
C	3.137101	-1.229739	-0.246516
C	3.136977	1.228320	-0.253410
C	4.475709	-1.204981	-0.667184
C	4.475618	1.201323	-0.673921
C	5.163590	-0.002393	-0.885917
H	4.991741	-2.151714	-0.829298
H	4.991542	2.147190	-0.841330
C	-2.476951	-0.000141	-0.030726
C	-3.136692	-1.229820	-0.247350
C	-3.137359	1.228239	-0.252698
C	-4.475336	-1.205214	-0.667956
C	-4.476014	1.201094	-0.673106

C	-5.163602	-0.002717	-0.885893
H	-4.991100	-2.152009	-0.830548
H	-4.992281	2.146901	-0.839807
C	0.000058	-0.005249	-1.809533
H	-0.932635	-0.006711	-2.359758
H	0.932744	-0.006969	-2.359761
C	2.419748	-2.548578	-0.042422
H	1.534723	-2.617043	-0.687824
H	3.081764	-3.389726	-0.275369
H	2.069892	-2.668614	0.991379
C	2.419526	2.548241	-0.056743
H	2.070251	2.674387	0.976533
H	3.081280	3.388091	-0.295048
H	1.534132	2.612723	-0.702040
C	6.600060	-0.003715	-1.371509
H	6.645120	-0.007692	-2.470557
H	7.141192	0.885823	-1.027256
H	7.141744	-0.890410	-1.020834
C	-2.420329	2.548266	-0.055213
H	-1.534746	2.613312	-0.700185
H	-3.082246	3.388033	-0.293362
H	-2.071437	2.674074	0.978234
C	-2.419009	-2.548567	-0.043854
H	-1.534287	-2.616772	-0.689704
H	-2.068635	-2.668678	0.989762
H	-3.080988	-3.389796	-0.276610
C	-6.600050	-0.004125	-1.371541
H	-7.142187	0.883783	-1.024644
H	-6.644997	-0.004774	-2.470599
H	-7.140797	-0.892463	-1.023606

Structure D (protonated)

57

scf done: -1042.451699

C	0.000004	0.038862	-0.367804
N	1.093481	-0.045830	0.429202
N	-1.093479	-0.045886	0.429183
C	-0.687245	-0.188119	1.772192
C	0.687232	-0.188082	1.772201
C	-1.667091	-0.306460	2.896174
H	-2.302833	-1.194370	2.789985
H	-1.138680	-0.384351	3.850104
H	-2.331319	0.565140	2.948084
C	1.667047	-0.306363	2.896215
H	1.138595	-0.384563	3.850098
H	2.303029	-1.194085	2.789908
H	2.331048	0.565400	2.948326
C	2.477750	0.003060	-0.032157
C	3.128300	-1.206936	-0.353228
C	3.118023	1.255808	-0.128727
C	4.460977	-1.129939	-0.783170
C	4.451642	1.268507	-0.563057
C	5.142712	0.092272	-0.892624
H	4.978904	-2.053095	-1.039607
H	4.962599	2.226795	-0.645644
C	-2.477748	0.002997	-0.032196
C	-3.128286	-1.206965	-0.353260
C	-3.118017	1.255786	-0.128800
C	-4.460994	-1.129962	-0.783223
C	-4.451602	1.268488	-0.563108
C	-5.142708	0.092222	-0.892644
H	-4.978895	-2.053109	-1.039720
H	-4.962554	2.226778	-0.645762
C	0.000018	0.213696	-1.848454
H	-0.000001	1.277646	-2.122902

H	0.892376	-0.240877	-2.288082
C	2.432118	-2.550628	-0.258527
H	1.588204	-2.626031	-0.957990
H	3.128203	-3.359730	-0.498085
H	2.033361	-2.741902	0.746019
C	2.412556	2.555149	0.207877
H	3.118726	3.390362	0.186522
H	1.614203	2.784900	-0.511304
H	1.952004	2.533397	1.203602
C	6.591465	0.137353	-1.328038
H	6.830446	-0.682656	-2.014282
H	6.833259	1.084230	-1.823153
H	7.259549	0.041938	-0.460166
C	-2.412497	2.555102	0.207775
H	-1.614058	2.784745	-0.511342
H	-3.118611	3.390360	0.186293
H	-1.952044	2.533395	1.203549
C	-2.432149	-2.550679	-0.258515
H	-1.587767	-2.625887	-0.957426
H	-2.034068	-2.742231	0.746250
H	-3.128077	-3.359718	-0.498745
C	-6.591493	0.137491	-1.327928
H	-7.259463	0.048294	-0.459296
H	-6.831704	1.082073	-1.828206
H	-6.832226	-0.685873	-2.009487
H	-0.892311	-0.240916	-2.288101

Structure 2

77

scf done: -1446.225708

N	0.691792	0.166750	-0.194281
N	-0.516101	0.243102	-0.753271
N	-1.367843	-0.098082	0.267094
C	0.695189	-0.221944	1.131463
C	1.878278	-0.426756	1.963571
C	3.062051	0.345405	1.844332
H	3.118426	1.141756	1.110212
C	4.163448	0.113049	2.669831
H	5.053785	0.729248	2.554343
C	4.125712	-0.888168	3.649501
H	4.983175	-1.059787	4.297053
C	2.963546	-1.657713	3.787383
H	2.916529	-2.444573	4.538752
C	1.858288	-1.432329	2.964905
H	0.975785	-2.055395	3.074462
C	-0.705491	-0.418955	1.484749
C	-1.341167	-0.780300	2.641249
H	-2.423391	-0.822252	2.685343
H	-0.787564	-0.967516	3.550502
C	1.822786	0.425498	-1.079754
C	2.511626	-0.675374	-1.636690
C	3.591076	-0.390498	-2.490152
H	4.148349	-1.211619	-2.935728
C	3.953431	0.923068	-2.787968
H	4.791595	1.117701	-3.454989
C	3.236740	1.988190	-2.241104
H	3.520787	3.007445	-2.493382
C	2.154616	1.768804	-1.372872
C	1.372124	2.962418	-0.815257
H	0.673146	2.586428	-0.060444
C	0.527706	3.630467	-1.930422
H	1.170182	4.043098	-2.719301
H	-0.067638	4.454147	-1.516323
H	-0.156441	2.908870	-2.390303
C	2.282557	4.000974	-0.115366

H	2.869375	3.546629	0.691844
H	1.669924	4.798771	0.323527
H	2.981966	4.471995	-0.817390
C	2.115243	-2.132544	-1.381267
H	1.278386	-2.139392	-0.675232
C	1.620463	-2.810377	-2.684328
H	0.786033	-2.255873	-3.128820
H	1.279063	-3.831920	-2.472831
H	2.421056	-2.874768	-3.432419
C	3.264189	-2.944269	-0.733596
H	4.129501	-3.023188	-1.404345
H	2.924398	-3.963563	-0.509351
H	3.599938	-2.484450	0.202228
C	-2.788340	-0.135125	0.027234
C	-3.564787	1.011316	0.327625
C	-4.949387	0.938789	0.102845
H	-5.572576	1.803971	0.320507
C	-5.541453	-0.221822	-0.396546
H	-6.616892	-0.254867	-0.564083
C	-4.757150	-1.340142	-0.681062
H	-5.231245	-2.239229	-1.069993
C	-3.368156	-1.326839	-0.472941
C	-2.541664	-2.575252	-0.791402
H	-1.497543	-2.355657	-0.546560
C	-2.596726	-2.921232	-2.300208
H	-3.616008	-3.177406	-2.617251
H	-1.953901	-3.784212	-2.517064
H	-2.255137	-2.078007	-2.912192
C	-2.967906	-3.784142	0.078042
H	-4.003543	-4.084989	-0.127255
H	-2.885060	-3.549217	1.145481
H	-2.322975	-4.647604	-0.132044
C	-2.952348	2.304459	0.872471
H	-1.872478	2.148660	0.965651
C	-3.167320	3.488711	-0.102701
H	-4.232468	3.729072	-0.215116
H	-2.766567	3.263449	-1.097835
H	-2.662843	4.387937	0.274599
C	-3.486087	2.644836	2.286292
H	-4.567075	2.836630	2.273690
H	-2.991963	3.547230	2.669797
H	-3.289563	1.826851	2.988339

Structure 2 (B3LYP-D3(BJ)-def2-TZVP)

77

scf done: -1447.113173

N	0.623802	0.217802	-0.173080
N	-0.568347	0.372565	-0.690224
N	-1.388988	-0.190420	0.227662
C	-1.320424	-1.312265	2.405216
H	-0.743728	-1.652870	3.246864
H	-2.395536	-1.382967	2.453464
C	-0.718380	-0.715318	1.345202
C	0.655814	-0.421100	1.027726
C	1.863165	-0.797019	1.738164
C	1.889788	-2.014159	2.437931
H	1.009458	-2.640399	2.437209
C	3.037179	-2.435125	3.091391
H	3.034904	-3.384494	3.611778
C	4.187099	-1.653560	3.073363
H	5.081871	-1.983078	3.584769
C	4.169912	-0.437428	2.398858
H	5.052815	0.188889	2.387276
C	3.024924	-0.008519	1.746694
H	3.034168	0.944757	1.242311

C	-2.796562	-0.167750	0.014994
C	-3.422914	-1.348650	-0.403920
C	-4.803153	-1.317450	-0.583456
H	-5.323386	-2.208600	-0.904354
C	-5.521292	-0.151410	-0.360088
H	-6.593956	-0.144537	-0.507502
C	-4.875994	1.005130	0.050231
H	-5.452958	1.903203	0.219868
C	-3.497337	1.021352	0.252497
C	-2.782608	2.259403	0.760906
H	-1.766252	2.227852	0.371052
C	-2.694254	2.233089	2.293992
H	-3.693472	2.264984	2.734796
H	-2.136504	3.099713	2.656657
H	-2.197658	1.329602	2.648849
C	-3.407080	3.565975	0.269829
H	-4.397606	3.731821	0.697545
H	-3.498164	3.581789	-0.817296
H	-2.782189	4.408747	0.570152
C	-2.599180	-2.589257	-0.693606
H	-1.780279	-2.606082	0.027092
C	-1.991867	-2.505363	-2.102098
H	-2.779464	-2.500051	-2.859246
H	-1.346218	-3.365780	-2.290694
H	-1.400160	-1.598853	-2.226062
C	-3.373708	-3.894350	-0.512275
H	-3.833037	-3.950173	0.475704
H	-2.694911	-4.742283	-0.617461
H	-4.158358	-4.013097	-1.262446
C	1.741085	0.669436	-0.957477
C	2.463483	-0.275429	-1.687072
C	3.547765	0.191566	-2.428260
H	4.136769	-0.508345	-3.005541
C	3.876633	1.537653	-2.441324
H	4.720804	1.879790	-3.026287
C	3.129097	2.450630	-1.711109
H	3.396154	3.498215	-1.733561
C	2.043784	2.034046	-0.945086
C	1.219170	3.027533	-0.149404
H	0.617636	2.464802	0.565267
C	2.084921	3.998205	0.659458
H	2.779376	3.465755	1.311012
H	1.450286	4.627710	1.285395
H	2.666002	4.658766	0.013967
C	0.254973	3.781836	-1.074561
H	0.807953	4.365814	-1.813500
H	-0.368653	4.468054	-0.498717
H	-0.395696	3.087084	-1.604691
C	2.102497	-1.747684	-1.700370
H	1.229053	-1.889475	-1.065347
C	1.718591	-2.204824	-3.112723
H	0.900474	-1.603978	-3.510610
H	1.398810	-3.248239	-3.096872
H	2.563546	-2.124498	-3.799312
C	3.234465	-2.606232	-1.122483
H	4.131871	-2.542543	-1.741268
H	2.929055	-3.653484	-1.084750
H	3.494359	-2.290917	-0.112844

Structure 2 (protonated)

78

scf done: -1446.678608

C	-3.376620	-1.326454	-0.449865
C	-2.796653	-0.129204	0.030532
C	-3.534510	1.042522	0.318931

C	-4.923105	0.974875	0.118061
C	-5.533802	-0.189675	-0.347613
C	-4.769272	-1.321710	-0.630867
N	-1.347770	-0.111432	0.253255
N	-0.526817	0.239709	-0.742207
N	0.701270	0.146560	-0.219375
C	0.681182	-0.264779	1.105419
C	-0.671904	-0.432629	1.408040
C	1.853481	0.430776	-1.083798
C	2.551638	-0.666148	-1.640310
C	3.652983	-0.361067	-2.456989
C	4.025046	0.958292	-2.712434
C	3.296482	2.013699	-2.163314
C	2.187504	1.783114	-1.333438
C	1.859354	-0.480583	1.965934
C	2.885533	0.479615	2.073075
C	3.971297	0.258421	2.923841
C	4.050917	-0.919275	3.677078
C	3.037413	-1.879788	3.577254
C	1.947499	-1.663703	2.730048
C	-1.345715	-0.842632	2.677504
C	2.144852	-2.130199	-1.443691
C	3.294373	-2.985767	-0.856210
C	1.385906	2.972435	-0.792990
C	2.274781	4.032795	-0.097732
C	-2.903842	2.349615	0.810334
C	-3.429450	2.758112	2.209958
C	-2.570435	-2.581672	-0.798148
C	-3.041344	-3.818679	0.006905
C	0.547843	3.618976	-1.927236
C	1.621748	-2.738118	-2.771266
C	-2.608842	-2.860844	-2.322673
C	-3.117993	3.493384	-0.213609
H	2.830271	1.402632	1.504543
H	4.753383	1.010154	3.002234
H	4.897985	-1.087285	4.338353
H	3.096588	-2.799818	4.154301
H	1.176590	-2.426056	2.644275
H	-1.569983	-1.917289	2.682277
H	-0.695746	-0.631348	3.531518
H	4.220214	-1.171333	-2.908149
H	4.881296	1.165808	-3.350842
H	3.590806	3.035975	-2.386549
H	0.681199	2.602086	-0.037585
H	1.198801	4.027579	-2.709721
H	-0.057423	4.443093	-1.530867
H	-0.123862	2.890394	-2.394904
H	2.880101	3.599210	0.707500
H	1.645773	4.817367	0.339851
H	2.958239	4.518509	-0.803633
H	1.316532	-2.169429	-0.725776
H	0.784928	-2.158282	-3.177671
H	1.280015	-3.767520	-2.606978
H	2.410518	-2.766891	-3.532478
H	4.141858	-3.051672	-1.548639
H	2.943414	-4.008119	-0.669311
H	3.662846	-2.572853	0.089507
H	-5.533659	1.850444	0.324522
H	-6.611389	-0.213406	-0.495893
H	-5.260621	-2.217065	-1.003662
H	-1.520597	-2.406147	-0.532327
H	-3.629174	-3.077269	-2.661217
H	-1.985626	-3.730249	-2.564557
H	-2.239191	-2.002866	-2.896065

H	-4.069466	-4.100159	-0.249181
H	-3.006679	-3.637650	1.088409
H	-2.399166	-4.679649	-0.215160
H	-1.820696	2.200366	0.902469
H	-4.181744	3.734988	-0.324565
H	-2.728958	3.225568	-1.202266
H	-2.605525	4.402626	0.123600
H	-4.504462	2.972435	2.187993
H	-2.917685	3.664663	2.555176
H	-3.262680	1.971750	2.956253
H	-2.291878	-0.309385	2.809116

Structure 4

70

scf done: -1254.495927

C	-3.040684	-1.239250	-0.377843
C	-2.400420	-0.000271	-0.126951
C	-3.041412	1.238369	-0.377662
C	-4.346076	1.208057	-0.896869
C	-4.993513	-0.000953	-1.154499
C	-4.345364	-1.209620	-0.897053
N	-1.072606	0.000137	0.425022
N	0.026607	-0.000015	-0.428625
N	1.056644	0.000131	0.427958
C	0.714581	0.000347	1.745629
C	-0.724173	0.000224	1.799860
C	2.401947	0.000279	-0.126633
C	3.024448	-1.241385	-0.386280
C	4.326411	-1.209220	-0.914214
C	4.972036	0.000578	-1.173083
C	4.326002	1.210227	-0.914518
C	3.024036	1.242092	-0.386586
C	1.660209	0.000316	2.896606
C	-1.570460	0.000233	2.879164
C	2.334526	-2.585240	-0.136010
C	3.111702	-3.455976	0.882295
C	2.333644	2.585774	-0.136693
C	3.110628	3.457170	0.881188
C	-2.361889	2.583422	-0.109135
C	-3.118388	3.407473	0.962095
C	-2.360390	-2.583959	-0.109540
C	-3.116553	-3.408787	0.961315
C	2.101114	3.351563	-1.463208
C	2.102460	-3.351607	-1.462269
C	-2.174117	-3.396042	-1.415220
C	-2.176292	3.395970	-1.414607
H	-2.647548	-0.000029	2.760316
H	-1.163072	0.000495	3.883550
H	4.838597	-2.144524	-1.129798
H	5.980698	0.000700	-1.582967
H	4.837876	2.145648	-1.130341
H	1.347809	2.383637	0.294994
H	3.050606	3.615435	-1.946747
H	1.553019	4.283103	-1.272463
H	1.516343	2.749476	-2.167786
H	3.252208	2.931820	1.833388
H	2.558569	4.383733	1.084575
H	4.101582	3.737404	0.501593
H	1.348554	-2.383310	0.295464
H	1.517574	-2.749998	-2.167160
H	1.554671	-4.283271	-1.271250
H	3.052109	-3.615329	-1.945579
H	4.102825	-3.735936	0.502939
H	2.559985	-4.382691	1.085918
H	3.252932	-2.930252	1.834339

H	-4.862335	2.143833	-1.102805
H	-6.005396	-0.001222	-1.556946
H	-4.861067	-2.145670	-1.103137
H	-1.362425	-2.376031	0.289654
H	-3.139339	-3.666221	-1.863076
H	-1.630870	-4.327962	-1.210247
H	-1.605448	-2.825086	-2.158691
H	-4.128405	-3.673756	0.627418
H	-3.200344	-2.849592	1.900261
H	-2.579221	-4.343743	1.168931
H	-1.363730	2.375988	0.289835
H	-3.141713	3.665628	-1.862342
H	-1.607282	2.825593	-2.158269
H	-1.633650	4.328201	-1.209432
H	-4.130411	3.672021	0.628378
H	-2.581529	4.342636	1.169998
H	-3.201783	2.847883	1.900840
H	1.497749	-0.881077	3.536605
H	2.704064	-0.000664	2.568196
H	1.499197	0.882637	3.535702

Structure 4 (protonated)

71

scf done: -1254.949576

C	3.013726	-1.260297	-0.374741
C	2.411526	-0.004552	-0.128102
C	3.040911	1.235550	-0.385140
C	4.343957	1.181971	-0.906057
C	4.976285	-0.036529	-1.155137
C	4.318237	-1.238841	-0.894946
N	1.057715	0.013448	0.433518
N	-0.000012	-0.007278	-0.385855
N	-1.057696	0.013578	0.433557
C	-0.695483	0.045432	1.762606
C	0.695539	0.045288	1.762595
C	-2.411535	-0.004223	-0.128009
C	-3.014086	-1.259896	-0.374187
C	-4.318586	-1.238284	-0.894402
C	-4.976278	-0.035892	-1.155131
C	-4.343598	1.182523	-0.906548
C	-3.040572	1.235956	-0.385564
C	-2.319916	-2.602085	-0.122099
C	-3.091775	-3.471017	0.902768
C	-2.370454	2.593914	-0.153739
C	-3.174154	3.478950	0.831741
C	1.662823	0.089217	2.900910
C	-1.662733	0.089412	2.900945
C	2.371084	2.593571	-0.152856
C	3.175079	3.478312	0.832625
C	2.319235	-2.602380	-0.123018
C	3.090587	-3.471453	0.902123
C	2.129854	3.331970	-1.494505
C	2.095721	-3.372581	-1.449439
C	-2.129388	3.332060	-1.495542
C	-2.096019	-3.372356	-1.448426
H	2.610088	-0.387720	2.633909
H	1.880159	1.124383	3.195981
H	-4.867649	2.109544	-1.125609
H	-5.986270	-0.048376	-1.559450
H	-4.823841	-2.178135	-1.102741
H	-1.328489	-2.406051	0.304951
H	-3.049275	-3.638257	-1.920590
H	-1.549113	-4.303565	-1.257215
H	-1.520049	-2.776446	-2.165661
H	-3.233904	-2.947435	1.856272

H	-2.539259	-4.396841	1.104086
H	-4.082533	-3.752566	0.527163
H	-1.385013	2.422870	0.297763
H	-1.529794	2.726066	-2.184494
H	-1.600414	4.276395	-1.318420
H	-3.076642	3.569840	-1.994143
H	-4.155907	3.747094	0.424147
H	-2.632718	4.412344	1.028137
H	-3.340337	2.973131	1.790872
H	4.823230	-2.178752	-1.103645
H	5.986295	-0.049131	-1.559411
H	4.868297	2.108930	-1.124687
H	1.385719	2.422577	0.298839
H	3.077061	3.569601	-1.993264
H	1.601112	4.276390	-1.317138
H	1.529997	2.726188	-2.183413
H	4.156787	3.746453	0.424915
H	3.341365	2.972288	1.791628
H	2.633782	4.411728	1.029294
H	1.327680	-2.406204	0.303662
H	3.049100	-3.638693	-1.921238
H	1.520152	-2.776552	-2.166899
H	1.548546	-4.303677	-1.258451
H	4.081495	-3.753004	0.526917
H	2.537932	-4.397263	1.103116
H	3.232317	-2.947957	1.855733
H	-1.245443	-0.422528	3.774362
H	-1.880737	1.124613	3.195358
H	-2.609700	-0.388398	2.634411
H	1.246003	-0.423565	3.774059

Structure 6

88

scf done: -1661.218311

C	3.244005	-0.790625	1.364340
C	2.661683	-0.807731	0.072501
C	3.444073	-0.994271	-1.098708
C	4.834756	-1.111822	-0.943239
C	5.431799	-1.061824	0.317335
C	4.641459	-0.908351	1.455634
N	1.223616	-0.705035	-0.057987
C	0.443173	0.441905	0.045700
N	-0.858226	0.030817	-0.064236
C	-0.947853	-1.367675	-0.208211
C	0.402518	-1.881472	-0.156727
C	-2.008767	0.929731	-0.062325
C	-2.826627	0.989057	1.094581
C	-3.926476	1.862299	1.064122
C	-4.206708	2.641897	-0.058067
C	-3.390412	2.557227	-1.185293
C	-2.278352	1.699195	-1.222313
C	-2.179174	-2.140729	-0.346640
C	-3.315562	-1.709136	-1.081785
C	-4.456007	-2.505924	-1.197916
C	-4.514515	-3.769921	-0.596154
C	-3.405451	-4.218856	0.132977
C	-2.263560	-3.426322	0.256136
C	0.914666	-3.153087	-0.194579
C	-2.567930	0.165530	2.365096
C	-3.765287	-0.759483	2.702182
C	-1.434503	1.635195	-2.503856
C	-2.248518	1.102772	-3.711518
C	2.839632	-1.074780	-2.508139
C	3.092221	-2.457929	-3.161115
C	2.415132	-0.683273	2.648593

C	2.465267	-2.006183	3.455589
C	-0.806415	3.009908	-2.847736
C	-2.230022	1.061646	3.584948
C	2.839384	0.520715	3.525020
C	3.356010	0.053570	-3.437782
H	-3.305601	-0.747117	-1.579554
H	-5.302067	-2.138417	-1.777043
H	-5.401528	-4.391929	-0.697515
H	-3.431228	-5.192733	0.620205
H	-1.432699	-3.785920	0.854908
H	1.983252	-3.324244	-0.166354
H	0.270088	-4.012060	-0.311103
H	-4.572709	1.933633	1.936343
H	-5.063925	3.313046	-0.055055
H	-3.622254	3.164920	-2.057395
H	-0.611902	0.932339	-2.335685
H	-1.578408	3.750700	-3.093009
H	-0.149847	2.914692	-3.722114
H	-0.212199	3.402511	-2.016642
H	-2.645311	0.098500	-3.526393
H	-1.606358	1.049822	-4.600179
H	-3.092536	1.762468	-3.950264
H	-1.702081	-0.479174	2.182247
H	-1.339937	1.677037	3.411733
H	-2.036021	0.435272	4.465186
H	-3.061696	1.733658	3.832655
H	-4.657087	-0.177288	2.968447
H	-3.515845	-1.394861	3.561631
H	-4.021257	-1.410522	1.861072
H	5.460305	-1.247421	-1.823399
H	6.512715	-1.152386	0.412223
H	5.115743	-0.888283	2.434766
H	1.370274	-0.523653	2.363527
H	3.866271	0.409575	3.896320
H	2.180333	0.601306	4.399255
H	2.781718	1.462364	2.967312
H	3.485673	-2.229094	3.794011
H	2.113252	-2.849034	2.849807
H	1.826285	-1.931702	4.345592
H	1.754930	-0.956368	-2.414342
H	4.439592	-0.019715	-3.596164
H	3.143110	1.050620	-3.035259
H	2.872643	-0.019905	-4.420701
H	4.164211	-2.637513	-3.316989
H	2.600397	-2.505611	-4.141575
H	2.689808	-3.265324	-2.541177
C	0.932904	1.807579	0.234440
C	2.170097	2.225317	-0.326712
C	2.650486	3.522817	-0.147603
C	1.922850	4.461793	0.594305
C	0.702549	4.070658	1.161537
C	0.218910	2.773180	0.994322
H	2.756311	1.533793	-0.917154
H	3.600470	3.801811	-0.600402
H	2.299980	5.472827	0.732323
H	0.124407	4.777437	1.754469
H	-0.715609	2.505897	1.469353

Structure 6 (B3LYP-D3(BJ)-def2-TZVP)

88

scf done: -1662.267547

N	-0.832225	0.010789	-0.180571
N	1.196177	-0.764945	-0.155002
C	0.850381	-3.181654	-0.310184
H	1.910108	-3.363056	-0.244318

H	0.197875	-4.023436	-0.454822
C	0.362941	-1.912643	-0.283111
C	-0.959303	-1.365352	-0.362424
C	-2.220767	-2.065055	-0.462891
C	-3.350513	-1.542674	-1.121367
H	-3.292964	-0.585876	-1.613546
C	-4.547706	-2.238446	-1.156030
H	-5.395819	-1.804262	-1.670644
C	-4.663859	-3.486152	-0.551728
H	-5.599201	-4.028898	-0.585720
C	-3.553814	-4.027097	0.088346
H	-3.624468	-4.994259	0.570513
C	-2.354956	-3.334391	0.132227
H	-1.516661	-3.754168	0.667606
C	-1.938036	0.923618	-0.116702
C	-2.171293	1.775317	-1.201055
C	-3.235411	2.668290	-1.095479
H	-3.443482	3.345438	-1.913106
C	-4.032368	2.699003	0.037984
H	-4.854702	3.400183	0.100560
C	-3.783095	1.830693	1.090377
H	-4.414354	1.861071	1.968221
C	-2.730044	0.921553	1.037539
C	-2.461773	-0.003145	2.212415
H	-1.679319	-0.701039	1.921540
C	-3.699461	-0.835910	2.566263
H	-4.509002	-0.206507	2.941685
H	-3.451673	-1.557577	3.346464
H	-4.065292	-1.384771	1.700213
C	-1.952154	0.773943	3.433747
H	-1.022066	1.300432	3.216995
H	-1.761754	0.087025	4.260589
H	-2.685776	1.509238	3.770727
C	-1.328263	1.745061	-2.463366
H	-0.547243	0.997589	-2.330768
C	-0.641041	3.093183	-2.715207
H	-1.374429	3.876776	-2.916064
H	0.015677	3.022524	-3.584257
H	-0.042425	3.401333	-1.859636
C	-2.161352	1.320386	-3.679793
H	-2.968566	2.029110	-3.874710
H	-2.604385	0.334660	-3.535952
H	-1.530856	1.278969	-4.569953
C	0.451304	0.381205	-0.039308
C	0.967264	1.720313	0.173661
C	2.219952	2.090853	-0.346985
H	2.798401	1.380794	-0.911328
C	2.728197	3.363961	-0.152714
H	3.693296	3.615939	-0.572841
C	2.009015	4.312408	0.565510
H	2.407737	5.306749	0.715655
C	0.772013	3.960143	1.094563
H	0.201384	4.678922	1.668288
C	0.259831	2.687226	0.910839
H	-0.691463	2.444497	1.350469
C	2.593886	-0.872991	0.104875
C	3.049192	-0.738620	1.422104
C	4.422443	-0.817120	1.644676
H	4.805483	-0.708773	2.650794
C	5.303069	-1.039207	0.597998
H	6.367030	-1.095592	0.790033
C	4.823699	-1.201765	-0.693642
H	5.519441	-1.387520	-1.501282
C	3.460462	-1.128222	-0.967167

C	2.954313	-1.304500	-2.388287
H	1.866334	-1.318312	-2.353073
C	3.405528	-2.643016	-2.984112
H	4.490951	-2.686213	-3.096377
H	2.963824	-2.779890	-3.972923
H	3.088977	-3.473982	-2.355348
C	3.382123	-0.141004	-3.293458
H	2.991645	0.813574	-2.939717
H	3.009004	-0.297298	-4.307614
H	4.469910	-0.060392	-3.346093
C	2.098894	-0.544374	2.588328
H	1.095529	-0.407584	2.189706
C	2.059813	-1.802606	3.464694
H	1.767140	-2.671866	2.874556
H	1.339627	-1.676836	4.276159
H	3.036717	-2.003902	3.909355
C	2.435083	0.707669	3.405013
H	3.407368	0.618158	3.893217
H	1.686549	0.858738	4.185333
H	2.453084	1.596094	2.773531

Structure 6 (protonated)

89

scf done: -1661.685709

C	-0.294904	2.650492	-1.236482
C	-0.959639	1.788412	-0.336828
C	-2.137135	2.254504	0.287046
C	-2.626924	3.535175	0.022415
C	-1.957720	4.378717	-0.871274
C	-0.790782	3.928922	-1.499377
C	-0.444476	0.428887	-0.081039
N	-1.193356	-0.713965	0.019368
C	-0.347612	-1.821091	0.170137
C	0.947785	-1.347400	0.196217
N	0.868985	0.056677	0.039674
C	-2.655472	-0.825941	-0.055002
C	-3.260913	-0.870203	-1.333756
C	-4.659752	-0.997489	-1.375606
C	-5.415753	-1.090805	-0.208209
C	-4.786761	-1.066338	1.036933
C	-3.392992	-0.936912	1.153469
C	-2.476457	-0.807387	-2.649792
C	-2.902577	0.399898	-3.522469
C	-2.762774	-0.935657	2.556876
C	-3.277479	0.232218	3.438895
C	2.019848	0.972203	0.067311
C	2.881256	1.020849	-1.059757
C	3.968622	1.907752	-0.994236
C	4.198184	2.700925	0.128855
C	3.340279	2.621480	1.224248
C	2.231443	1.758700	1.229520
C	2.189229	-2.137596	0.348598
C	3.185004	-1.806289	1.289286
C	4.316428	-2.612488	1.442564
C	4.475017	-3.765518	0.664701
C	3.495083	-4.105714	-0.274805
C	2.364617	-3.299331	-0.433976
C	-0.854123	-3.227008	0.250272
C	2.705493	0.173520	-2.330097
C	2.424645	1.039111	-3.586711
C	1.337307	1.727420	2.478486
C	0.693934	3.111475	2.753693
C	3.942435	-0.726817	-2.585903
C	2.104196	1.242323	3.736282
C	-3.002238	-2.278577	3.297398

C	-2.603563	-2.131950	-3.446097
H	3.074471	-0.924268	1.910595
H	5.071493	-2.341869	2.177439
H	5.355200	-4.392441	0.788774
H	3.612876	-4.994397	-0.891063
H	1.624958	-3.561760	-1.186394
H	-1.816927	-3.278655	0.763390
H	-0.135746	-3.855303	0.784324
H	4.648590	1.975915	-1.839875
H	5.048745	3.379088	0.151639
H	3.534037	3.240965	2.096557
H	0.521766	1.014925	2.307549
H	1.454341	3.856665	3.016334
H	-0.003256	3.041134	3.597684
H	0.142947	3.485657	1.885047
H	2.516886	0.235318	3.607981
H	1.427921	1.216955	4.599623
H	2.933365	1.915090	3.985191
H	1.841626	-0.487150	-2.191834
H	1.509983	1.634938	-3.491039
H	2.307362	0.392600	-4.465133
H	3.253094	1.727682	-3.791147
H	4.824686	-0.125615	-2.836486
H	3.749469	-1.396512	-3.432979
H	4.188322	-1.340377	-1.714286
H	-5.389859	-1.151353	1.937785
H	-6.497604	-1.190259	-0.267774
H	-5.162328	-1.029152	-2.339331
H	-1.413798	-0.675466	-2.414111
H	-3.946148	0.311546	-3.847464
H	-2.279810	0.450755	-4.424046
H	-2.797393	1.346480	-2.980938
H	-3.638790	-2.312870	-3.759278
H	-2.280893	-2.996034	-2.852694
H	-1.984828	-2.090259	-4.350946
H	-1.678160	-0.809811	2.450372
H	-4.357067	0.155604	3.613827
H	-3.080086	1.213376	2.993537
H	-2.782243	0.206052	4.417174
H	-4.069240	-2.437962	3.493500
H	-2.484526	-2.271096	4.264258
H	-2.637335	-3.140644	2.728936
H	-2.668988	1.624831	0.988118
H	-3.532205	3.873928	0.521185
H	-2.341554	5.375556	-1.076429
H	-0.264158	4.571682	-2.201222
H	0.604506	2.322201	-1.741334
H	-0.997679	-3.659519	-0.749043

Structure of MeIMes carbene

53

scf done: -1002.687421

C	-5.118230	0.000680	-0.985197
C	-4.431403	-1.201923	-0.765654
C	-3.096103	-1.228697	-0.333998
C	-2.441932	0.000081	-0.111893
C	-3.096072	1.229169	-0.332408
C	-4.431361	1.202987	-0.764103
N	-1.069147	-0.000226	0.346410
C	0.000014	0.000480	-0.521502
N	1.069159	-0.000191	0.346442
C	0.684521	-0.001296	1.709935
C	-0.684545	-0.001315	1.709924
C	2.441950	0.000125	-0.111843
C	3.096185	-1.228660	-0.333785

C	4.431465	-1.201880	-0.765470
C	5.118226	0.000735	-0.985212
C	4.431306	1.203029	-0.764272
C	3.096018	1.229207	-0.332544
C	-1.663013	-0.002133	2.844234
C	1.662968	-0.002088	2.844267
C	2.379225	-2.549193	-0.141788
C	2.378905	2.549456	-0.139179
C	6.550405	0.001056	-1.483528
C	-2.379083	-2.549226	-0.142187
C	-2.379016	2.549425	-0.138876
C	-6.550421	0.001046	-1.483473
H	-2.317784	-0.884374	2.822673
H	-1.135026	-0.002992	3.804034
H	-2.317574	0.880302	2.824126
H	1.134968	-0.002417	3.804059
H	2.317428	-0.884571	2.823028
H	2.317838	0.880107	2.823847
H	4.944432	-2.148397	-0.937688
H	4.944157	2.149783	-0.935524
H	-4.944328	-2.148439	-0.937987
H	-4.944265	2.149740	-0.935204
H	1.504413	-2.615884	-0.800983
H	3.047109	-3.388157	-0.365150
H	2.011182	-2.672949	0.885335
H	2.009934	2.671633	0.887793
H	3.046989	3.388751	-0.360689
H	1.504675	2.617144	-0.799049
H	6.585980	0.000558	-2.582914
H	7.094642	0.889437	-1.141050
H	7.095322	-0.886608	-1.140255
H	-1.504700	2.617179	-0.798625
H	-3.047093	3.388715	-0.360425
H	-2.010190	2.671554	0.888155
H	-1.504321	-2.615828	-0.801455
H	-2.010959	-2.673060	0.884897
H	-3.046957	-3.388190	-0.365575
H	-7.095272	0.888341	-1.139140
H	-6.586016	0.002848	-2.582857
H	-7.094713	-0.887702	-1.142036

Structure of ^{Me}IMes carbene (protonated)

54

scf done: -1003.139521

C	-5.110632	-0.000928	-1.031978
C	-4.430683	-1.205172	-0.793050
C	-3.107555	-1.237978	-0.328733
C	-2.470949	-0.000474	-0.103263
C	-3.108499	1.237196	-0.327998
C	-4.431152	1.203979	-0.792149
N	-1.095934	-0.000147	0.393264
C	0.000003	0.000036	-0.387167
N	1.095936	0.000286	0.393273
C	0.690165	0.000202	1.741175
C	-0.690171	-0.000121	1.741170
H	0.000009	-0.000009	-1.467684
C	2.470951	0.000493	-0.103261
C	3.108430	-1.237239	-0.327869
C	4.431086	-1.204138	-0.792030
C	5.110635	0.000705	-1.031974
C	4.430754	1.205013	-0.793164
C	3.107629	1.237936	-0.328855
C	-1.669612	-0.000185	2.871160
C	1.669603	0.000693	2.871167
C	2.411705	-2.563465	-0.094585

C	2.410605	2.564249	-0.096994
C	6.529564	-0.000684	-1.558084
C	-2.410505	-2.564228	-0.096600
C	-2.411806	2.563484	-0.094965
C	-6.529562	0.000346	-1.558087
H	-2.321683	-0.881885	2.840628
H	-1.142115	-0.003948	3.828671
H	-2.316853	0.885255	2.845036
H	1.142102	-0.001704	3.828682
H	2.321124	-0.881455	2.841607
H	2.317396	0.885692	2.844072
H	4.943809	-2.148725	-0.968130
H	4.943029	2.149609	-0.970089
H	-4.942901	-2.149812	-0.969896
H	-4.943932	2.148522	-0.968330
H	1.563307	-2.703924	-0.778262
H	3.104970	-3.393280	-0.259572
H	2.021032	-2.655392	0.927092
H	2.016618	2.655749	0.923425
H	3.104631	3.393950	-0.259339
H	1.564506	2.705338	-0.783412
H	6.534994	-0.058622	-2.655916
H	7.064021	0.912957	-1.276209
H	7.095265	-0.861606	-1.184597
H	-1.564252	2.704389	-0.779609
H	-3.105438	3.393215	-0.258830
H	-2.019937	2.655099	0.926272
H	-1.563455	-2.704815	-0.781934
H	-2.017864	-2.656090	0.924315
H	-3.104125	-3.394018	-0.260215
H	-7.095498	0.860913	-1.184129
H	-6.535014	0.058895	-2.655885
H	-7.063770	-0.913590	-1.276702

Structure of MIC

74

scf done: -1406.915992

C	-3.407785	-1.327406	-0.196710
C	-2.795247	-0.087462	0.088847
C	-3.522353	1.120092	0.187326
C	-4.910913	1.054915	-0.014266
C	-5.544599	-0.154835	-0.302080
C	-4.799317	-1.330836	-0.390897
N	-1.359002	-0.056489	0.328939
N	-0.545967	0.064660	-0.762091
N	0.673843	0.066556	-0.207030
C	0.611039	-0.049199	1.186005
C	-0.752519	-0.135556	1.553924
C	1.801046	0.152987	-1.127890
C	2.444293	-1.041960	-1.524594
C	3.534961	-0.923238	-2.402103
C	3.956968	0.321479	-2.870272
C	3.289814	1.481855	-2.473557
C	2.196904	1.429409	-1.593530
C	1.756541	-0.085681	2.115169
C	3.065884	0.343345	1.805887
C	4.084043	0.288932	2.764496
C	3.824775	-0.190444	4.052597
C	2.528380	-0.613865	4.376209
C	1.509374	-0.562606	3.425037
C	1.984632	-2.429451	-1.066527
C	3.102702	-3.200397	-0.322499
C	1.467731	2.718147	-1.200438
C	2.424648	3.794998	-0.633695
C	-2.860327	2.453467	0.546210

C	-3.154601	2.832710	2.020272
C	-2.626145	-2.642750	-0.254823
C	-2.957392	-3.537594	0.966501
C	0.648956	3.271461	-2.394693
C	1.435634	-3.254109	-2.258428
C	-2.848736	-3.400245	-1.585917
C	-3.257182	3.595818	-0.418866
H	3.300576	0.729421	0.820154
H	5.083734	0.629254	2.499521
H	4.619698	-0.230863	4.794998
H	2.309898	-0.986803	5.375511
H	0.500348	-0.880301	3.673193
H	4.055676	-1.820743	-2.729178
H	4.803607	0.387523	-3.551461
H	3.621255	2.444683	-2.856297
H	0.755041	2.474557	-0.405150
H	1.305618	3.553918	-3.227966
H	0.087685	4.164399	-2.090884
H	-0.065150	2.525730	-2.761731
H	2.988668	3.418134	0.227767
H	1.849820	4.669608	-0.303527
H	3.145118	4.139836	-1.386135
H	1.159857	-2.298145	-0.358191
H	0.618473	-2.726586	-2.763881
H	1.053263	-4.220891	-1.906491
H	2.218911	-3.454830	-3.000737
H	3.954635	-3.412736	-0.981199
H	2.718167	-4.161419	0.042420
H	3.472775	-2.633644	0.539311
H	-5.505126	1.963218	0.058326
H	-6.622384	-0.181143	-0.454538
H	-5.306302	-2.268478	-0.609164
H	-1.559458	-2.404017	-0.196647
H	-3.891017	-3.723364	-1.702669
H	-2.219973	-4.299448	-1.618918
H	-2.591959	-2.772703	-2.447834
H	-4.015390	-3.830951	0.970415
H	-2.745465	-3.012211	1.904757
H	-2.354275	-4.455048	0.942613
H	-1.776328	2.323494	0.460733
H	-4.323614	3.843992	-0.346052
H	-3.043053	3.331472	-1.461541
H	-2.693580	4.505699	-0.174683
H	-4.230598	2.980010	2.182870
H	-2.641994	3.767610	2.283654
H	-2.806529	2.046781	2.699741

Structure of MIC (protonated)

75

scf done: -1407.368851

C	-3.403256	-1.387959	-0.238950
C	-2.829423	-0.151297	0.134031
C	-3.570332	1.030748	0.363523
C	-4.962906	0.933459	0.210182
C	-5.570504	-0.268223	-0.155561
C	-4.800644	-1.410112	-0.378583
N	-1.375491	-0.097294	0.318241
N	-0.561823	0.089437	-0.721212
N	0.674644	0.089732	-0.194493
C	0.661708	-0.094899	1.182649
C	-0.688087	-0.215066	1.489308
C	1.811687	0.248102	-1.108144
C	2.520758	-0.912334	-1.494705
C	3.603563	-0.722065	-2.369168
C	3.945318	0.546500	-2.837220

C	3.207284	1.664810	-2.447026
C	2.116760	1.550704	-1.569961
C	1.812856	-0.168706	2.094618
C	2.974175	0.611968	1.921042
C	4.017727	0.533548	2.846494
C	3.922228	-0.319670	3.952365
C	2.773104	-1.099603	4.133416
C	1.725353	-1.026011	3.214393
H	-1.188939	-0.348736	2.436494
C	2.149315	-2.330838	-1.050373
C	3.314696	-3.035664	-0.312374
C	1.308032	2.797190	-1.192991
C	2.197352	3.952778	-0.671926
C	-2.938558	2.367991	0.763860
C	-3.328121	2.766625	2.210496
C	-2.592310	-2.663048	-0.488820
C	-2.989416	-3.794887	0.492340
C	0.437741	3.264622	-2.388980
C	1.658951	-3.177373	-2.253625
C	-2.713735	-3.128099	-1.962086
C	-3.293019	3.494342	-0.238908
H	3.060636	1.287755	1.076754
H	4.905536	1.145587	2.704995
H	4.738481	-0.377210	4.668878
H	2.695042	-1.769755	4.986358
H	0.847033	-1.653109	3.353070
H	4.180037	-1.583945	-2.696008
H	4.786781	0.664028	-3.516865
H	3.480052	2.643761	-2.832800
H	0.623272	2.532978	-0.377193
H	1.065374	3.566287	-3.236372
H	-0.172189	4.129035	-2.099574
H	-0.232400	2.468621	-2.732938
H	2.811409	3.643272	0.182447
H	1.567655	4.789847	-0.347231
H	2.870366	4.332814	-1.449136
H	1.313416	-2.267976	-0.342868
H	0.813412	-2.701218	-2.763673
H	1.340300	-4.169702	-1.911536
H	2.457521	-3.320972	-2.991262
H	4.175179	-3.189179	-0.974338
H	2.992209	-4.022018	0.042777
H	3.654577	-2.455715	0.553139
H	-5.579181	1.813672	0.375437
H	-6.651530	-0.314401	-0.269452
H	-5.291640	-2.336551	-0.665982
H	-1.532605	-2.442805	-0.311154
H	-3.745594	-3.403593	-2.210573
H	-2.084215	-4.009737	-2.132858
H	-2.398628	-2.341351	-2.657389
H	-4.029622	-4.108833	0.345833
H	-2.880196	-3.480939	1.537888
H	-2.352730	-4.673486	0.332000
H	-1.847155	2.257744	0.741274
H	-4.367317	3.712693	-0.237915
H	-3.004680	3.226272	-1.261811
H	-2.769414	4.418429	0.034753
H	-4.409067	2.927038	2.301494
H	-2.826536	3.699463	2.495445
H	-3.045737	1.993613	2.936246

Structure of aNHC

85

scf done: -1621.908587

C	0.443608	2.793905	0.768108

C	0.995531	1.771529	-0.036285
C	2.100442	2.102197	-0.854710
C	2.630246	3.394296	-0.861610
C	2.070728	4.395533	-0.058626
C	0.973164	4.086771	0.753355
C	0.467412	0.395362	-0.023773
N	1.198190	-0.772014	0.010517
C	0.411466	-1.930666	0.001204
C	-0.887983	-1.423041	-0.065678
N	-0.846120	0.007813	-0.062713
C	2.646964	-0.865439	0.141889
C	3.234971	-0.679548	1.414511
C	4.629834	-0.820332	1.514844
C	5.406627	-1.149092	0.404621
C	4.798040	-1.356018	-0.834333
C	3.409605	-1.223447	-0.996955
C	2.411433	-0.416002	2.679814
C	2.929548	0.792011	3.495785
C	2.769234	-1.517739	-2.358705
C	3.510204	-0.849086	-3.541475
C	-1.978849	0.925630	-0.086383
C	-2.732528	1.110837	1.102332
C	-3.839516	1.973217	1.041139
C	-4.189412	2.627174	-0.140193
C	-3.433583	2.426816	-1.295301
C	-2.317944	1.573474	-1.302308
C	-2.104266	-2.255814	-0.147203
C	-3.329541	-1.865461	-0.731829
C	-4.413032	-2.749929	-0.800437
C	-4.306935	-4.048673	-0.293806
C	-3.095437	-4.455812	0.283372
C	-2.014968	-3.577255	0.357672
C	-2.405066	0.414730	2.432129
C	-2.104634	1.426425	3.567942
C	-1.546252	1.368573	-2.613177
C	-0.986155	2.702038	-3.168864
C	-3.539673	-0.547478	2.869447
C	-2.410230	0.656111	-3.685730
C	2.641183	-3.047645	-2.578482
C	2.338440	-1.695405	3.553294
H	-3.449001	-0.871212	-1.145990
H	-5.342504	-2.417377	-1.260282
H	-5.151184	-4.733504	-0.348944
H	-2.993304	-5.464059	0.682172
H	-1.072753	-3.893480	0.796590
H	-4.438247	2.133874	1.935312
H	-5.052133	3.290841	-0.161272
H	-3.717817	2.938318	-2.212582
H	-0.691920	0.715684	-2.406679
H	-1.793702	3.384635	-3.463153
H	-0.374611	2.510686	-4.060054
H	-0.360806	3.215863	-2.431234
H	-2.759764	-0.322962	-3.340022
H	-1.821195	0.499495	-4.598670
H	-3.289630	1.255381	-3.954742
H	-1.504884	-0.191743	2.286727
H	-1.269322	2.091794	3.320752
H	-1.840229	0.888108	4.487092
H	-2.977255	2.053385	3.790998
H	-4.465421	0.002148	3.084540
H	-3.247920	-1.079863	3.783829
H	-3.756862	-1.291231	2.097111
H	5.412527	-1.631649	-1.688494
H	6.485411	-1.256024	0.506384

H	5.112109	-0.684707	2.480572
H	1.386171	-0.181911	2.375796
H	3.934096	0.612691	3.899430
H	2.264653	0.983672	4.348115
H	2.969022	1.700965	2.883922
H	3.334569	-1.988136	3.910410
H	1.920039	-2.533085	2.983750
H	1.702407	-1.522993	4.431939
H	1.749724	-1.115872	-2.344130
H	4.514020	-1.266842	-3.688712
H	3.617894	0.234101	-3.399883
H	2.950821	-1.013183	-4.471499
H	3.630386	-3.524696	-2.596060
H	2.147509	-3.254384	-3.537693
H	2.045453	-3.497717	-1.777149
H	2.540464	1.346610	-1.496140
H	3.479488	3.619389	-1.504013
H	2.483991	5.402128	-0.066067
H	0.528407	4.852691	1.386091
H	-0.399242	2.577104	1.413742

Structure of aNHC (protonated)

86

scf done: -1622.381645

C	0.408245	2.699987	1.025428
C	0.992335	1.767745	0.142119
C	2.107156	2.169401	-0.625557
C	2.619880	3.462862	-0.508983
C	2.034369	4.378038	0.373749
C	0.927815	3.991327	1.138783
C	0.466617	0.395931	0.024537
N	1.217970	-0.743932	-0.021485
C	0.374680	-1.837444	-0.131660
C	-0.925035	-1.396994	-0.160879
N	-0.851055	0.012041	-0.060268
C	2.674676	-0.887962	0.094795
C	3.256805	-0.823978	1.382700
C	4.647562	-1.009581	1.460144
C	5.411749	-1.263287	0.321897
C	4.800704	-1.345222	-0.929801
C	3.416579	-1.162081	-1.080931
C	2.454055	-0.615325	2.671620
C	2.961783	0.597146	3.490083
C	2.782582	-1.298143	-2.472308
C	3.485862	-0.425069	-3.541292
C	-2.003213	0.924579	-0.074392
C	-2.801800	1.024559	1.093815
C	-3.905912	1.891296	1.037674
C	-4.204924	2.618220	-0.113937
C	-3.401541	2.493879	-1.246585
C	-2.281683	1.645637	-1.263648
C	-2.133538	-2.237646	-0.258479
C	-3.262523	-1.889940	-1.027302
C	-4.346808	-2.766218	-1.131115
C	-4.326176	-4.002440	-0.475579
C	-3.211064	-4.359508	0.292341
C	-2.127164	-3.486421	0.403278
H	0.768498	-2.839684	-0.197959
C	-2.538888	0.248989	2.394093
C	-2.266802	1.190195	3.596590
C	-1.449642	1.549652	-2.551030
C	-0.868208	2.927434	-2.960058
C	-3.711011	-0.708661	2.733629
C	-2.261179	0.941993	-3.724819
C	2.751400	-2.781525	-2.926517

C	2.447499	-1.904328	3.534500
H	-3.296583	-0.942826	-1.553555
H	-5.207544	-2.482180	-1.732605
H	-5.172293	-4.680701	-0.559835
H	-3.188448	-5.313868	0.813787
H	-1.278904	-3.763944	1.025377
H	-4.542786	1.996713	1.912609
H	-5.067047	3.281845	-0.129292
H	-3.648714	3.064451	-2.138554
H	-0.601376	0.879139	-2.369665
H	-1.663562	3.628461	-3.240006
H	-0.209257	2.813685	-3.829596
H	-0.289254	3.381791	-2.149382
H	-2.622186	-0.067510	-3.497762
H	-1.631661	0.877073	-4.620811
H	-3.130049	1.562612	-3.974334
H	-1.642779	-0.367934	2.256917
H	-1.405890	1.847240	3.427824
H	-2.060097	0.596226	4.495234
H	-3.133739	1.825515	3.813061
H	-4.626440	-0.148489	2.959376
H	-3.462078	-1.306007	3.619288
H	-3.929523	-1.394337	1.909469
H	5.409181	-1.556997	-1.805477
H	6.486590	-1.406833	0.410922
H	5.136420	-0.965224	2.430308
H	1.412104	-0.406480	2.402343
H	3.983790	0.439282	3.854417
H	2.321484	0.753253	4.366906
H	2.954711	1.516681	2.894002
H	3.458511	-2.163507	3.871187
H	2.051818	-2.762129	2.976870
H	1.824726	-1.760792	4.426129
H	1.742152	-0.954680	-2.412886
H	4.514755	-0.755597	-3.724500
H	3.522508	0.631824	-3.251143
H	2.945803	-0.493955	-4.493303
H	3.767256	-3.180474	-3.035037
H	2.248384	-2.872420	-3.897000
H	2.220710	-3.419288	-2.209474
H	2.564677	1.479272	-1.325292
H	3.474883	3.756452	-1.113699
H	2.436401	5.384726	0.463671
H	0.468369	4.693965	1.830268
H	-0.443381	2.416302	1.632065

Structure of 7

88

scf done: -1662.267547

N	-0.832225	0.010789	-0.180571
N	1.196177	-0.764945	-0.155002
C	0.850381	-3.181654	-0.310184
H	1.910108	-3.363056	-0.244318
H	0.197875	-4.023436	-0.454822
C	0.362941	-1.912643	-0.283111
C	-0.959303	-1.365352	-0.362424
C	-2.220767	-2.065055	-0.462891
C	-3.350513	-1.542674	-1.121367
H	-3.292964	-0.585876	-1.613546
C	-4.547706	-2.238446	-1.156030
H	-5.395819	-1.804262	-1.670644
C	-4.663859	-3.486152	-0.551728
H	-5.599201	-4.028898	-0.585720
C	-3.553814	-4.027097	0.088346
H	-3.624468	-4.994259	0.570513

C	-2.354956	-3.334391	0.132227
H	-1.516661	-3.754168	0.667606
C	-1.938036	0.923618	-0.116702
C	-2.171293	1.775317	-1.201055
C	-3.235411	2.668290	-1.095479
H	-3.443482	3.345438	-1.913106
C	-4.032368	2.699003	0.037984
H	-4.854702	3.400183	0.100560
C	-3.783095	1.830693	1.090377
H	-4.414354	1.861071	1.968221
C	-2.730044	0.921553	1.037539
C	-2.461773	-0.003145	2.212415
H	-1.679319	-0.701039	1.921540
C	-3.699461	-0.835910	2.566263
H	-4.509002	-0.206507	2.941685
H	-3.451673	-1.557577	3.346464
H	-4.065292	-1.384771	1.700213
C	-1.952154	0.773943	3.433747
H	-1.022066	1.300432	3.216995
H	-1.761754	0.087025	4.260589
H	-2.685776	1.509238	3.770727
C	-1.328263	1.745061	-2.463366
H	-0.547243	0.997589	-2.330768
C	-0.641041	3.093183	-2.715207
H	-1.374429	3.876776	-2.916064
H	0.015677	3.022524	-3.584257
H	-0.042425	3.401333	-1.859636
C	-2.161352	1.320386	-3.679793
H	-2.968566	2.029110	-3.874710
H	-2.604385	0.334660	-3.535952
H	-1.530856	1.278969	-4.569953
C	0.451304	0.381205	-0.039308

C	0.967264	1.720313	0.173661
C	2.219952	2.090853	-0.346985
H	2.798401	1.380794	-0.911328
C	2.728197	3.363961	-0.152714
H	3.693296	3.615939	-0.572841
C	2.009015	4.312408	0.565510
H	2.407737	5.306749	0.715655
C	0.772013	3.960143	1.094563
H	0.201384	4.678922	1.668288
C	0.259831	2.687226	0.910839
H	-0.691463	2.444497	1.350469
C	2.593886	-0.872991	0.104875
C	3.049192	-0.738620	1.422104
C	4.422443	-0.817120	1.644676
H	4.805483	-0.708773	2.650794
C	5.303069	-1.039207	0.597998
H	6.367030	-1.095592	0.790033
C	4.823699	-1.201765	-0.693642
H	5.519441	-1.387520	-1.501282
C	3.460462	-1.128222	-0.967167
C	2.954313	-1.304500	-2.388287
H	1.866334	-1.318312	-2.353073
C	3.405528	-2.643016	-2.984112
H	4.490951	-2.686213	-3.096377
H	2.963824	-2.779890	-3.972923
H	3.088977	-3.473982	-2.355348
C	3.382123	-0.141004	-3.293458
H	2.991645	0.813574	-2.939717
H	3.009004	-0.297298	-4.307614
H	4.469910	-0.060392	-3.346093
C	2.098894	-0.544374	2.588328
H	1.095529	-0.407584	2.189706

C	2.059813	-1.802606	3.464694
H	1.767140	-2.671866	2.874556
H	1.339627	-1.676836	4.276159
H	3.036717	-2.003902	3.909355
C	2.435083	0.707669	3.405013
H	3.407368	0.618158	3.893217
H	1.686549	0.858738	4.185333
H	2.453084	1.596094	2.773531

Structure of 7’

77

scf done: -1447.103078

C	-3.570972	0.683323	2.674258
C	-2.515213	0.506999	1.802098
C	-2.725879	-0.036917	0.536476
C	-3.998309	-0.350267	0.066261
C	-5.050853	-0.181001	0.973041
C	-4.848641	0.315076	2.254273
N	-1.513410	-0.095929	-0.193870
N	-0.595395	0.806090	0.539185
C	-1.050768	0.812629	1.990353
N	0.702607	0.255132	0.348816
C	0.518742	-1.081823	-0.124359
C	-0.773457	-1.315451	-0.363053
C	1.674285	1.114345	-0.281906
C	2.908381	1.258860	0.375460
C	3.864747	2.109302	-0.175902
C	3.611555	2.805708	-1.344679
C	2.401100	2.632738	-1.996591
C	1.421996	1.774891	-1.499080
C	-1.423581	-2.565378	-0.823280
C	1.670544	-1.984671	-0.285335
C	2.530606	-1.850991	-1.377536
C	3.619422	-2.699650	-1.526145
C	3.864783	-3.692518	-0.584389
C	3.017629	-3.831302	0.508349
C	1.929842	-2.980140	0.657890
C	3.234376	0.523712	1.662663
C	4.488450	-0.346188	1.524128
C	0.138483	1.597358	-2.287986
C	0.404469	1.289819	-3.766444
C	-0.780617	2.212438	2.527875
C	-0.398467	-0.246440	2.889003
C	-4.258403	-0.738932	-1.374262
C	-5.021442	-2.059692	-1.506340
C	-0.778395	2.816729	-2.133813
C	3.369538	1.497873	2.838770
C	-4.989302	0.396914	-2.103826
H	-2.236954	-2.861672	-0.156325
H	-0.690199	-3.369064	-0.851650
H	-1.846592	-2.453711	-1.824090
H	2.343423	-1.074317	-2.106113
H	4.277815	-2.584202	-2.377366
H	4.714633	-4.352648	-0.700069
H	3.205784	-4.599494	1.247329
H	1.273803	-3.078926	1.512755
H	4.817318	2.231602	0.322990
H	4.358739	3.473996	-1.754008
H	2.216783	3.166211	-2.919705
H	-0.400864	0.748229	-1.885164
H	-1.007972	2.985149	-1.082731
H	-1.716850	2.656832	-2.669802
H	-0.307454	3.716659	-2.536768
H	0.862171	2.131414	-4.289339
H	-0.536500	1.063489	-4.272379

H	1.064318	0.427470	-3.879180	H	-5.974631	0.574071	-1.668120
H	2.400781	-0.139924	1.873868	H	-5.126086	0.149046	-3.158664
H	4.202779	2.187175	2.686874	H	-4.421643	1.326118	-2.040162
H	3.550277	0.953719	3.768585	H	-6.021582	-1.983781	-1.075322
H	2.465503	2.094920	2.964280	H	-4.502144	-2.873190	-0.998796
H	4.391153	-1.046910	0.696456	H	-5.135458	-2.331424	-2.557723
H	4.650510	-0.920900	2.438501	H	0.287977	2.425546	2.485408
H	5.380266	0.260011	1.351764	H	-1.104588	2.299747	3.566478
H	-6.058874	-0.416773	0.656424	H	-1.303490	2.956887	1.929609
H	-5.693094	0.441880	2.919086	H	-0.531695	-1.247417	2.483545
H	-3.415394	1.104073	3.659714	H	-0.871456	-0.212893	3.871830
H	-3.289861	-0.853717	-1.858821	H	0.664835	-0.054462	3.012035

12. References

(1) K. Powers, C. Hering-Junghans, R. McDonald, M. J. Ferguson, E. Rivard, *Polyhedron* **2016**, 8-14.

(2) A. Füstner, M. Alcarazo, R. Goddard, C. W. Lehmann, *Angew. Chem. Int. Ed.* **2008**, *47*, 3210-3214.

(3) N. Kuhn, H. Bohnen, J. Kreutzberg, D. Bläser, R. Boese, *J. Chem. Soc. Chem. Commun.* **1993**, 1136-1137.

(4) S. Naumann, A. W. Thomas, A. P. Dove, *Angew. Chem. Int. Ed.* **2015**, *54*, 9550-9554.

(5) W.-C- Chen, J.-S. Shen, T. Jurca, C.-J. Peng, Y.-H. Lin, Y.-P. Wang. W.-C. Shih, G. P. A. Yap, T.-G. Ong, *Angew. Chem. Int. Ed.* **2015**, *54*, 15207-15212.

(6) I. C. Watson, A. Schumann, H. Yu, E. C. Davy, R. McDonald, M. J. Ferguson, C. Hering-Junghans, E. Rivard, *Chem. Eur. J.* **2019**, *25*, 9678-9690.

(7) A. J. Arduengo, F. Davidson, H. V. R. Dias, J. R. Goerlich, D. Khasnis, W. J. Marshall, T. K. Prakasha, *J. Am. Chem. Soc.* **1997**, *119*, 12742-12749.

(8) S. M. I. Al-Rafia, A. C. Malcolm, S. K. Liew, M. J. Ferguson, R. McDonald, E. Rivard, *Chem. Commun.* **2011**, *47*, 6987-6989.

(9) D. T. Chase, J. P. Moerdyk, C. W. Bielawski, *Org. Lett.* **2014**, *16*, 812-815.

(10) K. Schwedtmann, R. Schoemaker, F. Hennersdorf, A. Bauza, A. Frontera, R. Weiss, J. J. Weigand, *Dalton. Trans.* **2016**, *45*, 11384-11396.

(11) P. Walther, W. Frey, S. Naumann, *Poly. Chem.* **2018**, *9*, 3674-3683.

(12) T. X. Gentner, G. Ballmann, J. Pahl, H. Elsen, S. Harder, *Organometallics* **2018**, *37*, 4473-4480

(13) J. Bouffard, B. K. Keitz, R. Tonner, G. Guisado-Barrios, G. Frenking, R. H. Grubbs, G. Bertrand, *Organometallics* **2011**, *30*, 2617-2627.

(14) a) N. Nimitsiriwat, V. C. Gibson, E. L. Marshall, P. Takolpockdee, A. K. Tomov, A. J. P. White, D. J. Williams, M. R. J. Elsegood, S. H. Dale, *Inorg. Chem.* **2007**, *46*, 9988–9997; b) A. G. Barrett, M. R. Crimmin, M. S. Hill, P. B. Hitchcock, G. Kociok-Köhn, P. A. Procopiou, *Inorg. Chem.* **2008**, *47*, 7366–7376. Note: **Caution**: "The reaction of $DippNH_2$ with iAmONO should **NOT** be scaled up beyond amounts specified in these refs. as a violent delayed-onset runaway reaction can occur." from ref. 13a. We did not encounter problems with the synthesis but also did not scale up beyond these amounts. For more details see SI in ref. 13a.

(15) H. M. Teeter, E. W. Bell *Org. Synth.* **1952**, *32*, 20.

(16) E. Aldeco-Perez, A. J. Rosenthal, B. Donnadieu, P. Parameswaran, G. Frenking, G. Bertrand, *Science* **2009**, *326*, 556–559

(17) Sheldrick, G. M. *Acta Cryst*. **2008**, *A64*, 112.

(18) Dolomanov, O.V.; Bourhis, L. J.; Gildea, R. J.; Howard, J. A. K.; Puschmann, H. *J. Appl. Cryst.* **2009,** *42*, 339.

(19) Kottke, T.; Stalke, D. *J. Appl. Cryst*. **1993**, *26*, 615.

(20) S. Kronig, P. G. Jones, M. Tamm, *Eur. J. Inorg. Chem.* **2013**, 2301–2314;

(21) T. Dröge, F. Glorius, *Angew. Chem. Int. Ed.* **2010**, *49*, 6940-6952.

(22) R. A. Kelly III, H. Clavier, S. Giudice, N. M. Scott, E. D. Stevens, J. Bordner, I. Samardjiev, C. D. Hoff, L. Cavallo, S. P. Nolan, *Organometallics* **2008**, 27, 202−210.

(23) G. Ung, G. Bertrand, *Chem. Eur. J.* **2011**, *17*, 8269–8272.

(24) H. V. Huynh, *Chem. Rev.* **2018**, *118*, 9457–9492.

(25) Gaussian 16, Revision A.03, M. J. Frisch, G. W. Trucks, H. B. Schlegel, G. E. Scuseria, M. A. Robb, J. R. Cheeseman, G. Scalmani, V. Barone, G. A. Petersson, H. Nakatsuji, X. Li, M. Caricato, A. V. Marenich, J. Bloino, B. G. Janesko, R. Gomperts, B. Mennucci, H. P. Hratchian, J. V. Ortiz, A. F. Izmaylov, J. L. Sonnenberg, D. Williams-Young, F. Ding, F. Lipparini, F. Egidi, J. Goings, B. Peng, A. Petrone, T. Henderson, D. Ranasinghe, V. G. Zakrzewski, J. Gao, N. Rega, G. Zheng, W. Liang, M. Hada, M. Ehara, K. Toyota, R. Fukuda, J. Hasegawa, M. Ishida, T. Nakajima, Y. Honda, O. Kitao, H. Nakai, T. Vreven, K. Throssell, J. A. Montgomery, Jr., J. E. Peralta, F. Ogliaro, M. J. Bearpark, J. J. Heyd, E. N. Brothers, K. N. Kudin, V. N. Staroverov, T. A. Keith, R. Kobayashi, J. Normand, K. Raghavachari, A. P. Rendell, J. C. Burant, S. S. Iyengar, J. Tomasi, M. Cossi, J. M. Millam, M. Klene, C. Adamo, R. Cammi, J. W. Ochterski, R. L. Martin, K. Morokuma, O. Farkas, J. B. Foresman, and D. J. Fox, Gaussian, Inc., Wallingford CT, 2016.

(26) a) P. Hohenberg, W. Kohn, *Phys. Rev. B* **1964**, 136, B864; b) W. Kohn, L. J. Sham, *Phys. Rev.* **1965**, 140, A1133.

(27) a) F. Weigend, R. Ahlrichs, *Phys. Chem. Chem. Phys.* **2005**, *7*, 3297; b) F. Weigend, *Phys. Chem. Chem. Phys.* **2006**, *8*, 1057.

(28) NBO 6.0. E. D. Glendening, J. K. Badenhoop, A. E. Reed, J. E. Carpenter, J. A. Bohmann, C. M. Morales, C. R. Landis, F. Weinhold (Theoretical Chemistry Institute, University of Wisconsin, Madison, WI, 2013); http://nbo6.chem.wisc.edu/

(29) R. Schuldt, J. Kästner, S. Naumann, *J. Org. Chem.* **2019**, 2209-2218.
(30) IBOView: G. Knizia, *J. Chem. Theory Comput.* **2013**, *9*, 4834.
(31) R. Dennington, T. A. Keith, J. M. Millam **2016** GaussView, Version 6.
(32) CYLview v1.0.561 2009-2012 C. Y. Legault, University of Sherbrooke, Canada.
(33) a) S. Grimme, S. Ehrlich, L. Goerigk, *J. Comp. Chem*. **2011**, **32**, 1456; b) S. Grimme, J. Antony, S. Ehrlich, H. Krieg, *J.Chem.Phys.* **2010**, *132*, 154104.
